# Supplementary material for: Marine plankton show threshold extinction response to Neogene climate change
Source: Nat Commun. 2020 Oct 22;11:5069. doi: 10.1038/s41467-020-18879-7 (PMC7582175; doi:10.1038/s41467-020-18879-7)
Supplement: Supplementary file 1 — Supplementary Information [file 41467_2020_18879_MOESM1_ESM.pdf]

Supplementary Information

Marine plankton show threshold extinction response to Neogene climate change

Trubovitz *et al.*

This file contains:

Supplementary Figure 1
Supplementary Figure 2
Supplementary References

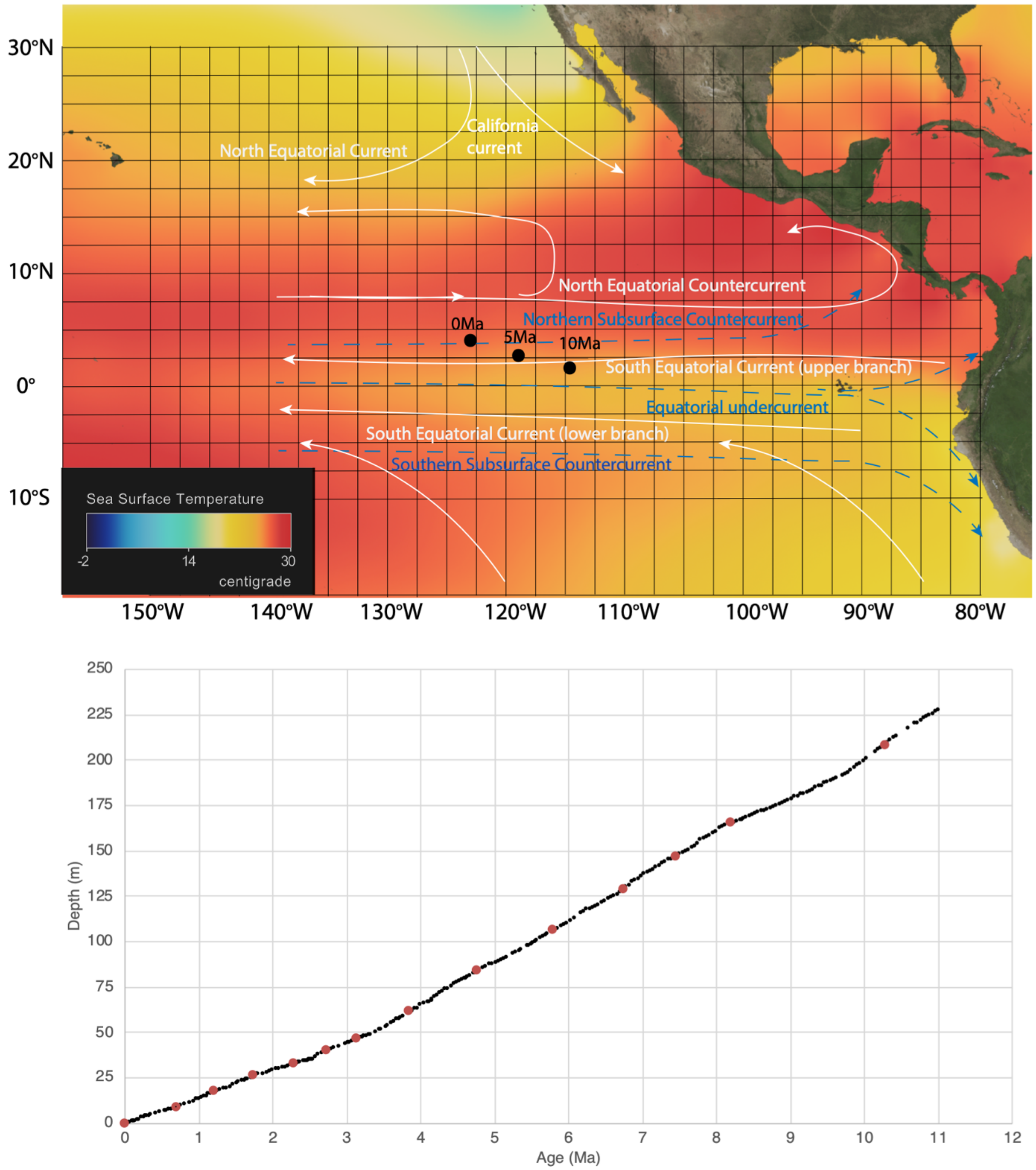

Supplementary Figure 1. Geographic position of IODP Site U1337 over the last 10 Ma (top), and age-depth model of sediment core with sampling intervals marked in red (bottom). Modern SST background and temperature scale from NASA/Goddard Space Flight Center Scientific Visualization Studio. Backtracked paleoposition of IODP Site U1337 is indicated with black points labelled 10 Ma, 5 Ma, and 0 Ma; data from [1] and [2]. Generalized eastern equatorial Pacific circulation patterns are adapted from [3]. Surface currents are shown in white and subsurface currents are shown in dashed light blue. Sediment age-depth model is replotted from [4].

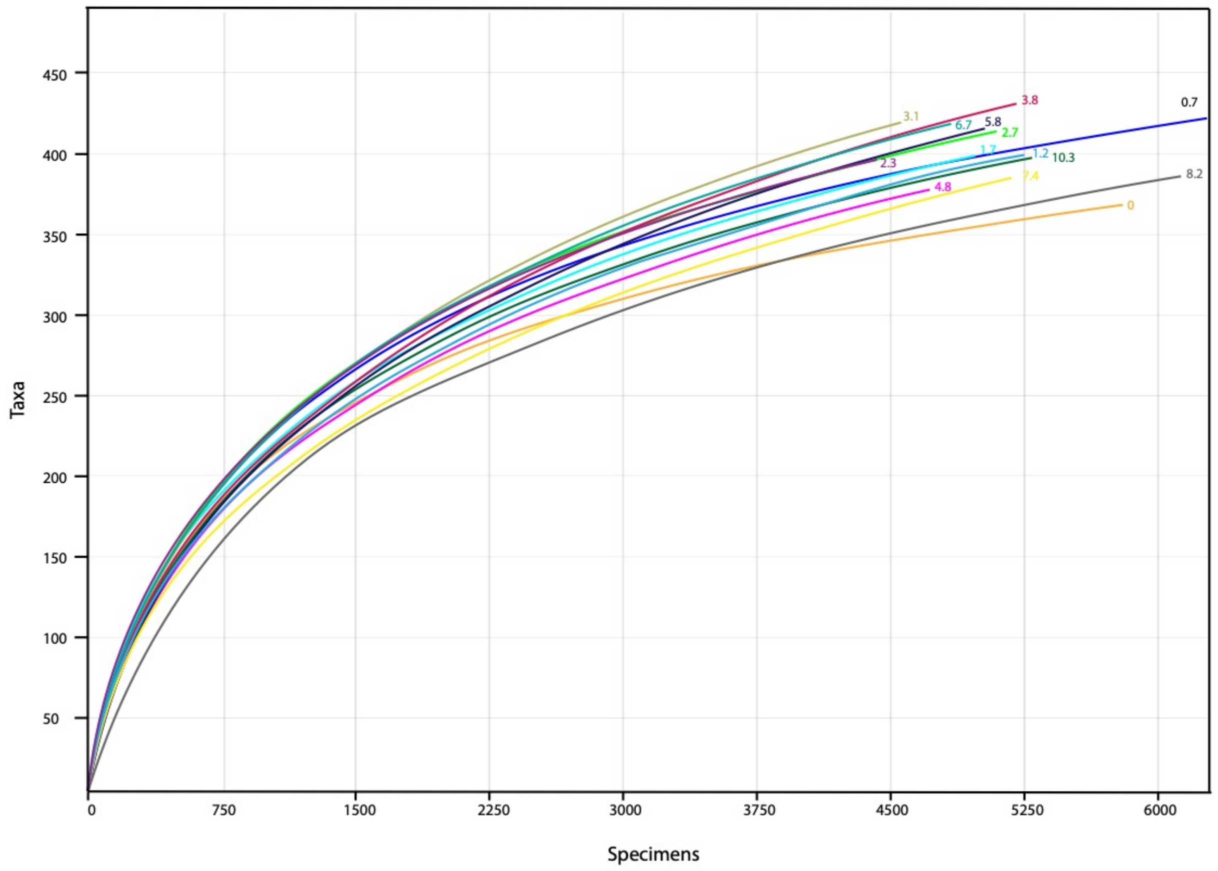

Supplementary Figure 2. Collection curves for eastern equatorial Pacific (EEP) samples. Curves illustrate the rate at which new taxa were observed with increasing sampling effort for each EEP sample. Flattening of curve shape is how adequate sample coverage was initially assessed during data collection, and later confirmed using coverage metrics (see Methods). All curves began to flatten after approximately 4000 specimens had been counted, indicating that few new species would be encountered with continued sampling. The consistent curvature across samples suggests reasonably full coverage and similar biodiversity levels over time. Colors denote individual EEP samples and matching numbers indicate sample ages (Ma).

Supplementary References

1. Pälike, H. *et al.* Expedition 320/321 Summary. *Proc. Integr. Ocean Drill. Progr.* **320/321**, 1–340 (2010).
2. Liu, J. *et al.* Eastern equatorial Pacific cold tongue evolution since the late Miocene linked to extratropical climate. *Sci. Adv.* **5**, eaau6060 (2019).
3. Kessler, W. S. The circulation of the eastern tropical Pacific: A review. *Prog. Oceanogr.* **69**, 181–217 (2006).
4. Tian, J. *et al.* Paleoceanography of the east equatorial Pacific over the past 16 Myr and Pacific–Atlantic comparison: High resolution benthic foraminiferal δ18O and δ13C records at IODP Site U1337. *Earth Planet. Sci. Lett.* **499**, 185–196 (2018).
