# Supplementary material for: Marine plankton show threshold extinction response to Neogene climate change
Source: Nat Commun. 2020 Oct 22;11:5069. doi: 10.1038/s41467-020-18879-7 (PMC7582175; doi:10.1038/s41467-020-18879-7)
Supplement: Supplementary file 3 — Reporting Summary [file 41467_2020_18879_MOESM3_ESM.pdf]

nature research

Corresponding author(s): Sarah Trubovitz

Last updated by author(s): Sep 13, 2020

Reporting Summary

Nature Research wishes to improve the reproducibility of the work that we publish. This form provides structure for consistency and transparency in reporting. For further information on Nature Research policies, see our Editorial Policies and the Editorial Policy Checklist.

Statistics

For all statistical analyses, confirm that the following items are present in the figure legend, table legend, main text, or Methods section.

n/a	Confirmed	
☐	☒	The exact sample size (*n*) for each experimental group/condition, given as a discrete number and unit of measurement
☐	☒	A statement on whether measurements were taken from distinct samples or whether the same sample was measured repeatedly
☐	☒	The statistical test(s) used AND whether they are one- or two-sided<br>*Only common tests should be described solely by name; describe more complex techniques in the Methods section.*
☒	☐	A description of all covariates tested
☒	☐	A description of any assumptions or corrections, such as tests of normality and adjustment for multiple comparisons
☐	☒	A full description of the statistical parameters including central tendency (e.g. means) or other basic estimates (e.g. regression coefficient) AND variation (e.g. standard deviation) or associated estimates of uncertainty (e.g. confidence intervals)
☐	☒	For null hypothesis testing, the test statistic (e.g. *F*, *t*, *r*) with confidence intervals, effect sizes, degrees of freedom and *P* value noted<br>*Give P values as exact values whenever suitable.*
☒	☐	For Bayesian analysis, information on the choice of priors and Markov chain Monte Carlo settings
☒	☐	For hierarchical and complex designs, identification of the appropriate level for tests and full reporting of outcomes
☒	☐	Estimates of effect sizes (e.g. Cohen's *d*, Pearson's *r*), indicating how they were calculated

Our web collection on statistics for biologists contains articles on many of the points above.

Software and code

Policy information about availability of computer code

Data collection	Raritas (version 0.7) software was used for specimen enumeration and observation of collection curve growth during data collection. ToupLite (version 1.0) camera software was used for photographing specimens. Graphic Converter (version 10) was used for organizing specimen images.
Data analysis	R (version 3.6.0) was used for extinction rate calculations and statistical analyses, with the addition of the open-source packages iNEXT (version 2.0.20), divDyn (version 0.8.0), and nlme (version 3.1-137). Details on specific functions and packages used for each analysis are given in Methods. Past (version 4.03) was used to calculate Pielou equitability ("diversity indices" function).

For manuscripts utilizing custom algorithms or software that are central to the research but not yet described in published literature, software must be made available to editors and reviewers. We strongly encourage code deposition in a community repository (e.g. GitHub). See the Nature Research guidelines for submitting code & software for further information.

Data

Policy information about availability of data

All manuscripts must include a data availability statement. This statement should provide the following information, where applicable:

- Accession codes, unique identifiers, or web links for publicly available datasets
- A list of figures that have associated raw data
- A description of any restrictions on data availability

All data generated or analyzed during this study are available in the Zenodo repository (https://doi.org/10.5281/zenodo.4014322).

Field-specific reporting

Please select the one below that is the best fit for your research. If you are not sure, read the appropriate sections before making your selection.

☐ Life sciences ☐ Behavioural & social sciences ☒ Ecological, evolutionary & environmental sciences

For a reference copy of the document with all sections, see nature.com/documents/nr-reporting-summary-flat.pdf

Ecological, evolutionary & environmental sciences study design

All studies must disclose on these points even when the disclosure is negative.

Study description	This study is based on a new biodiversity census of late Neogene and Quaternary fossil polycystine radiolaria from the eastern equatorial Pacific. A sequence of 14 sediment samples ranging in age from 10.3 to 0.0 million years old were analyzed from a single location (IODP Site U1337) representative of the low to mid latitude radiolarian bioprovinces. Approximately 5000 specimens were enumerated from each sample, which were used to establish the number of species present and their relative abundances in the assemblage. Biodiversity and ecologic metrics were calculated for each sample and visually examined for a trend through time. The tropical time series data collected for this study were compared to the radiolarian diversity trend in the Neptune Sandbox Berlin (NSB) database, and also compared to a published high latitude radiolarian diversity dataset from the Southern Ocean (Renaudie and Lazarus, 2013). Both low and high latitude diversity time series were interpreted in the context of previously-published regional temperature reconstructions.
Research sample	All samples were comprised of polycystine radiolaria species. This is a carbon cycle important group of heterotrophic marine plankton (Rhizaria). Samples are well preserved (based on prior publications, the large majority of original diversity is expected to be present as fossils) and were processed to include the >45 μm size fraction, which accounts for the minimum size of polycystine radiolarian species known from previous research.
Sampling strategy	During data collection, adequate sample size was determined by visually examining collection curve flattening (see Supplementary Figure 2). To verify completeness of >90%, established coverage metrics were calculated using iNEXT v. 2.0.20 (details in Methods). Standardized coverage accounts for variations in community evenness that are not possible when samples are standardized by size alone. Sampling was concluded to be sufficient when completeness values were >90% (exact sample sizes and completeness values are given in Supplementary Data 1).
Data collection	Species occurrence and abundance data were collected by counting specimens observed in transmitted light microscopy using Raritas enumeration software. All new data presented in this study were collected by Trubovitz. The Southern Ocean dataset was collected by Renaudie, and has been published by Renaudie and Lazarus (2013). The same software and species identification concepts were employed in both data collection efforts. Data was also obtained from the NSB microfossil database for comparison to our study.
Timing and spatial scale	The eastern equatorial Pacific samples range in age from 10.3 to 0.0 million years old, and are between 0.4 and 2.1 million years apart. Unequal spacing is in part due to an update to the original age model, which was published after samples had been processed. It is also due to intended high-frequency sampling around the Pliocene-Pleistocene transition, as we wanted to ensure sufficient documentation across this interval of established environmental change. All new tropical samples are from IODP Site U1337, and were enumerated by Trubovitz from 2018-2020. Comparison was made to tropical Pacific radiolarian diversity in NSB (all longitudes, ≤ 20° N/S), and to a previously-published dataset from the Southern Ocean (Renaudie and Lazarus, 2013).
Data exclusions	For extinction rate analysis of Southern Ocean data, the youngest time bin (0-1 Ma) was removed according to pre-established criteria to account for an edge effect bias (see Methods).
Reproducibility	We did not reproduce this study. It was not considered critical to our study due to the robust sample standardization technique used to verify our results (Methods; Supplementary Data 1 and Supplementary Data 5).
Randomization	Specimens were randomly-settled from solution onto glass cover slips during slide-making. This procedure ensures that each slide displays a random subset and even distribution of specimens from the sample.
Blinding	Blinding was not relevant to this study, since the fossil specimens were not subjected to experimental treatments.

Did the study involve field work? ☐ Yes ☒ No

Reporting for specific materials, systems and methods

We require information from authors about some types of materials, experimental systems and methods used in many studies. Here, indicate whether each material, system or method listed is relevant to your study. If you are not sure if a list item applies to your research, read the appropriate section before selecting a response.

Materials & experimental systems

n/a	Involved in the study
☒	☐ Antibodies
☒	☐ Eukaryotic cell lines
☐	☒ Palaeontology and archaeology
☒	☐ Animals and other organisms
☒	☐ Human research participants
☒	☐ Clinical data
☒	☐ Dual use research of concern

Methods

n/a	Involved in the study
☒	☐ ChIP-seq
☒	☐ Flow cytometry
☒	☐ MRI-based neuroimaging

Palaeontology and Archaeology

Specimen provenance	Eastern equatorial Pacific sediment samples were obtained from the International Ocean Discovery Program (IODP) Gulf Coast Repository, in April 2017. All of these samples originated from IODP Site U1337 (international waters at 4°N, 123°W). No permits were necessary to obtain samples from the repository. Sediments were processed for siliceous microfossils using standard procedures at the Museum für Naturkunde in Berlin, during May and June 2017. No new Southern Ocean specimens were obtained for this study.
Specimen deposition	Specimens from the eastern equatorial Pacific are subject to ongoing research at the University of Nevada, Reno, but will be deposited at the Museum für Naturkunde in Berlin once research is completed (likely in early 2021). The Southern Ocean microfossil specimens referred to in this study have been deposited by Renaudie and Lazarus at the Museum für Naturkunde.
Dating methods	No new dates are provided. Ages are from published age models, cited in the main text.

☒ Tick this box to confirm that the raw and calibrated dates are available in the paper or in Supplementary Information.

Ethics oversight	No ethical approval or guidance was required as our study did not involve human or other living subjects.

Note that full information on the approval of the study protocol must also be provided in the manuscript.
