# Supplementary material for: Marine plankton show threshold extinction response to Neogene climate change
Source: Nat Commun. 2020 Oct 22;11:5069. doi: 10.1038/s41467-020-18879-7 (PMC7582175; doi:10.1038/s41467-020-18879-7)
Supplement: Supplementary file 4 — Description of Additional Supplementary Files [file 41467_2020_18879_MOESM4_ESM.pdf]

# Description of Additional Supplementary Files

**Supplementary Data 1.**
This table includes the eastern equatorial Pacific (EEP) sample ages, within-sample species richness, range-through species richness, extrapolated species richness (including standard error on extrapolation calculations), Pielou equitability (evenness), sample coverage, total specimens counted at all taxonomic levels, and specimens counted at the species level.

**Supplementary Data 2.**
This table provides estimates of radiolarian diversity reported in the literature; it specifies the data source, time interval, geographic coverage, data type, and citation.

**Supplementary Data 3.**
This table summarizes the results of the Southern Ocean (SO) extinction versus extirpation analysis. Part A gives the absolute number of extinctions and extirpations per 1 million-year time bin. Part B shows extinction and extirpations as a percent of SO species richness within each time bin. Part C gives lists of last occurrences datums for SO species recorded in each 1 million-year time bin.

**Supplementary Data 4.**
This table lists the name of each EEP species included in analyses, along with its mean, maximum, minimum, and standard deviation in abundance throughout the 14 samples in the time series.

**Supplementary Data 5.**
This table provides the SO sample ages, within-sample species richness, range-through species richness, extrapolated species richness (including standard error on extrapolation calculations), sample coverage, specimens counted at the species level, and Pielou equitability per 0.5 million-year time bin.

**Supplementary Data 6.**
This table lists the name of each SO species included in analyses, along with its mean, maximum, minimum, and standard deviation in abundance throughout the time series.

**Supplementary Data 7.** Photo documentation of all species-level taxa observed in the EEP. Genus and species names, higher taxon, original author (if applicable), and number of photographic records in our digital image library are given. If a species is undescribed, an informal working description is given. Scale bars are 50 μm.
