# Supplementary material for: Marine plankton show threshold extinction response to Neogene climate change
Source: Nat Commun. 2020 Oct 22;11:5069. doi: 10.1038/s41467-020-18879-7 (PMC7582175; doi:10.1038/s41467-020-18879-7)
Supplement: Supplementary file 11 — Supplementary Data 7 [file 41467_2020_18879_MOESM11_ESM.pdf]

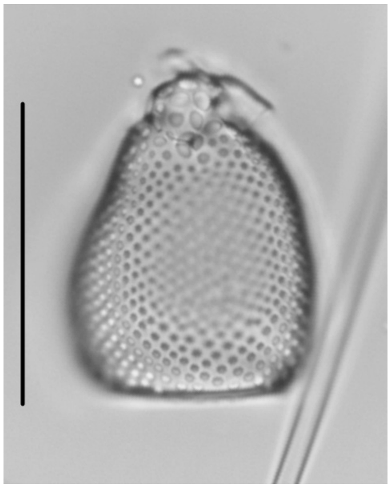

Artobotrys borealis
Artostrobiidae
Cleve, 1899
Records: 21

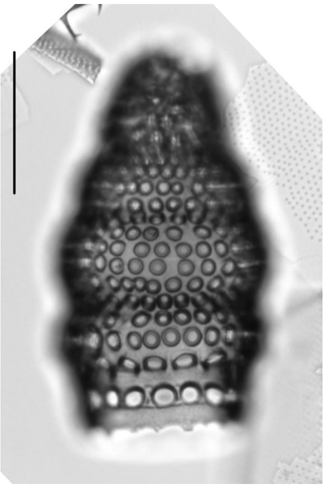

Botryostrobus aquilonaris
Artostrobiidae
Bailey, 1856
Records: 115

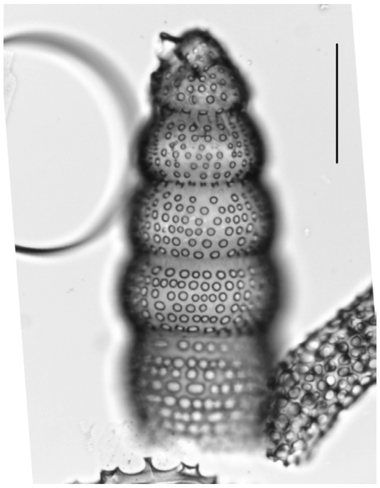

Botryostrobus auritus australis
Artostrobiidae
(Ehrenberg) Nigrini, 1977
Records: 115

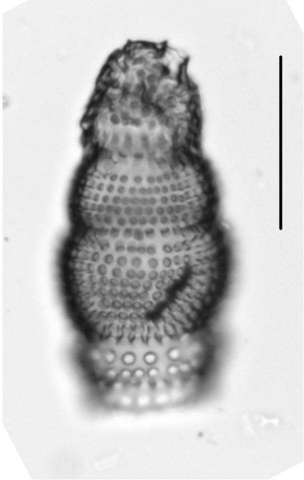

Botryostrobus bramlettei
Artostrobiidae
Campbell & Clark, 1944
Records: 146

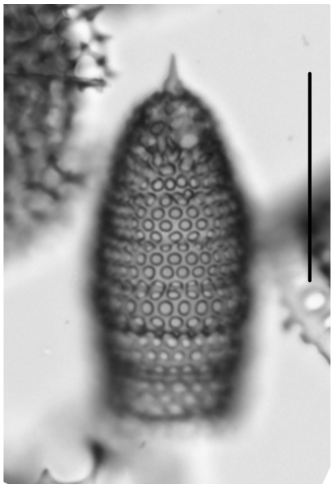

Botryostrobus joides
Artostrobiidae
Petrushevskaya, 1975
Records: 15

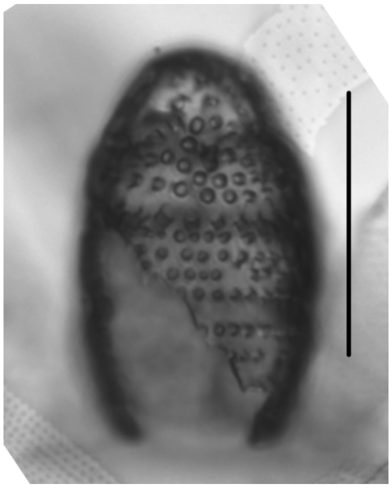

Phormostichoartus ashbyi
Artostrobiidae
Renaudie & Lazarus, 2015
Records: 5

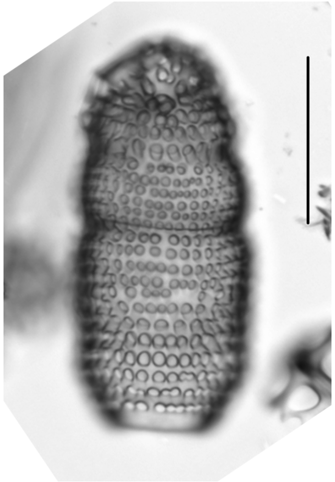

Phormostichoartus corbula
Artostrobiidae
(Harting) Nigrini, 1977
Records: 59

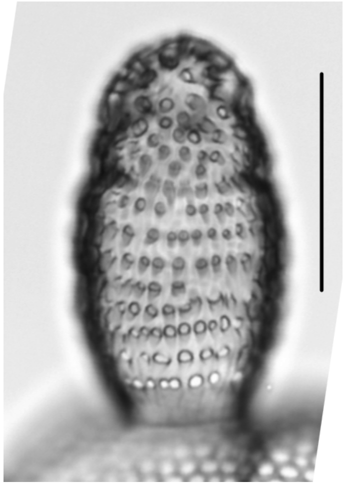

Phormostichoartus crustula
Artostrobiidae
(Caulet) Nigrini & Caulet, 1992
Records: 48

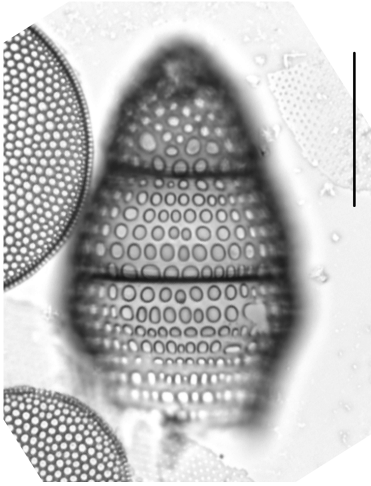

Phormostichoartus doliolum
Artostrobiidae
Riedel & Sanfilippo, 1971
Records: 78

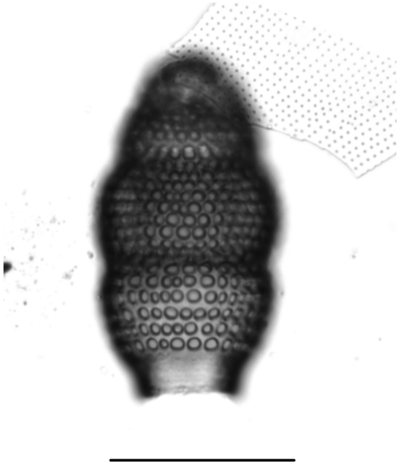

Phormostichoartus fistula
Artostrobiidae
Nigrini, 1977
Records: 130

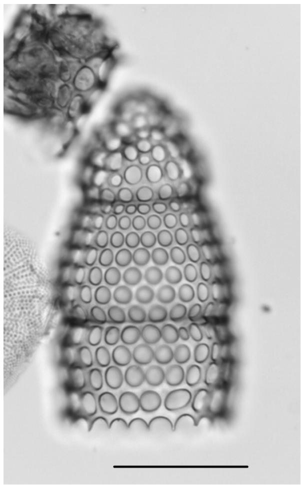

Phormostichoartus fistula cf
Artostrobiidae
Undescribed
Records: 23

Like P. fistula, except pores are larger, segments tend to be more elongated, and thorax is somewhat tapered in shape.

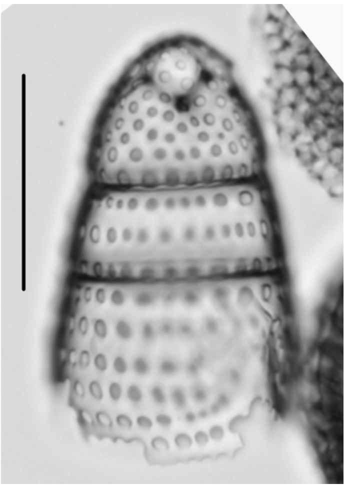

Phormostichoartus marylandicus
Artostrobiidae
Martin, 1904
Records: 40

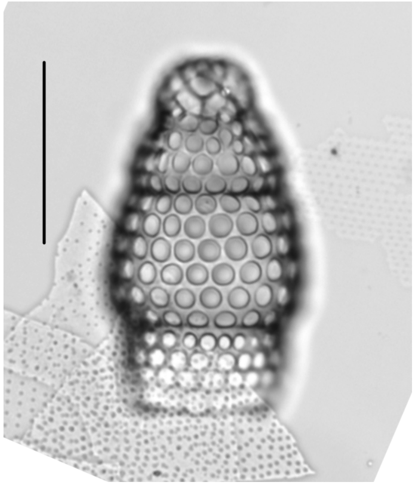

Phormostichoartus pitomorphus
Artostrobiidae
Caulet, 1986
Records: 79

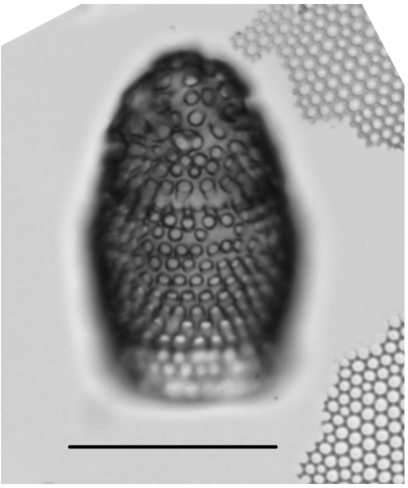

Phormostichoartus platycephala
Artostrobiidae
(Ehrenberg) Caulet, 1986
Records: 59

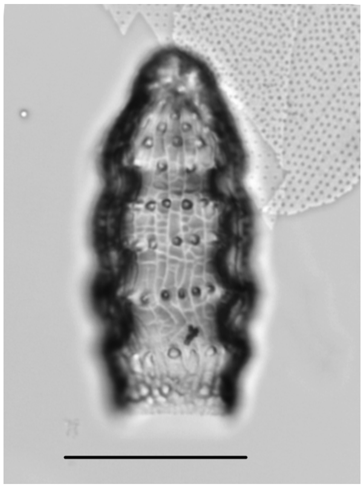

Siphocampe arachnea
Artostrobiidae
(Ehrenberg) Nigrini, 1977
Records: 85

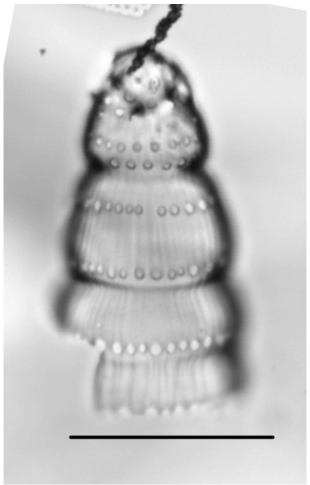

Siphocampe grantmackiei
Artostrobiidae
O'Connor, 1997
Records: 28

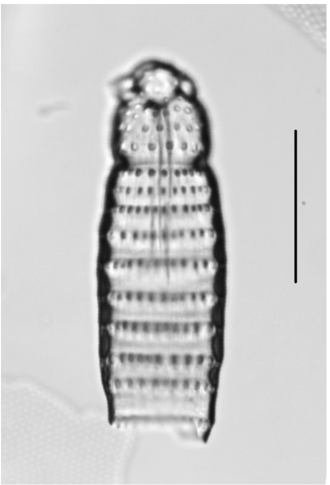

Siphocampe lineata
Artostrobiidae
(Ehrenberg) Nigrini, 1977
Records: 46

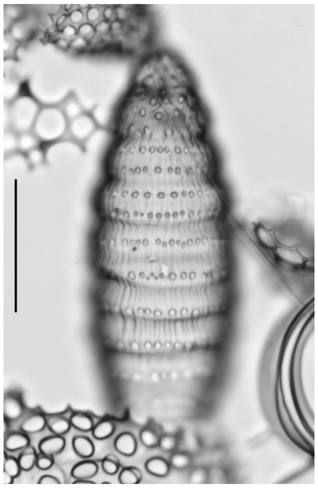

Siphocampe lineata cf
Artostrobiidae
Undescribed
Records: 93

Parallel lineations are like those of S. lineata, except that this species has several post-thoracic segments, with 1-2 rows of pores each. It differs from S. grantmackiei in that there is very little hyaline space between rows of pores, and the segments are not as thick.

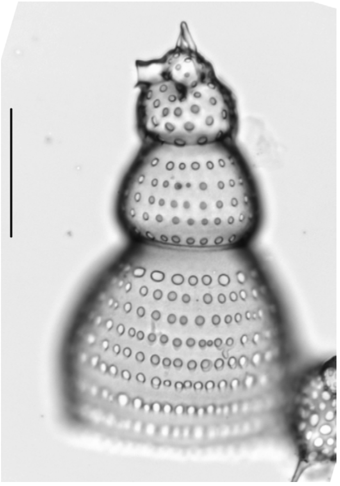

Siphostichartus corona
Artostrobiidae
(Haeckel) Nigrini, 1977
Records: 40

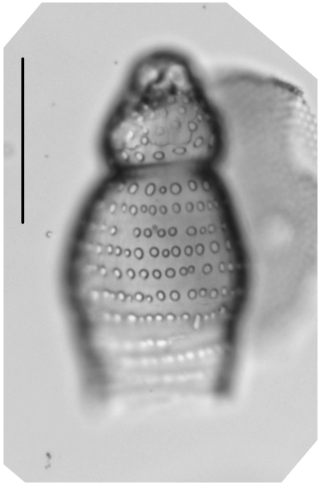

Siphostichoartus corona cf
Artostrobiidae
Undescribed
Records: 46

Similar to S. corona, except with only two thoracic segments instead of three; the middle segment is missing, but the flared final segment is present. Distinguished from S. lineata by flared final segment, and from P. crustula by structure of cephalis and relatively thin shell.

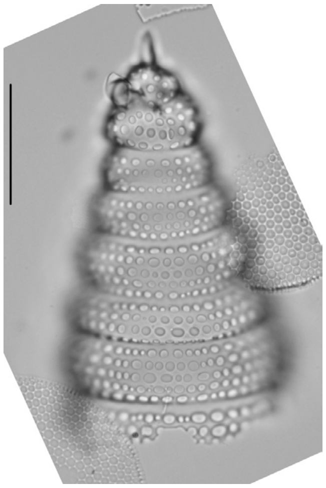

Spirocyrtis gyroscalaris
Artostrobiidae
Nigrini, 1977
Records: 26

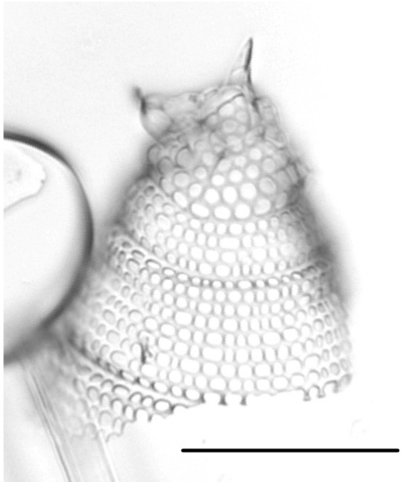

Spirocyrtis scalaris
Artostrobiidae
Haeckel, 1887
Records: 27

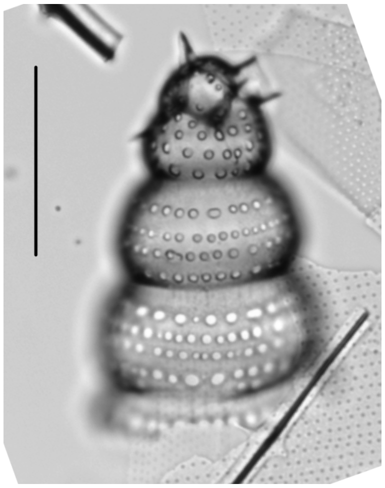

Spirocyrtis subscalaris
Artostrobiidae
Nigrini, 1977
Records: 86

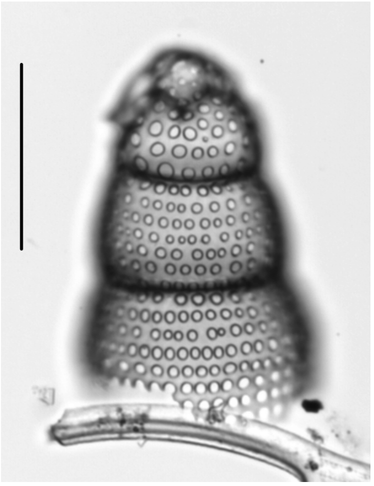

Spirocyrtis subscalaris cf
Artostrobiidae
Undescribed
Records: 77

Different from S. subscalaris in that cephalic tube is pointed downwards rather than upwards; there is no hyaline space between segments; segments do not flare out at their centers; and abdominal segment is consistently quadrate/barrel-shaped. Actually has very little in common with S. subscalaris, except for general shell thickness, pore size, and number of pores per segment.

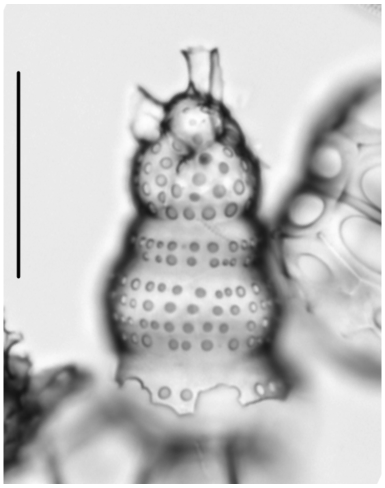

Spirocyrtis subtilis

Artostrobiidae

Petrushevskaya, 1972

Records: 8

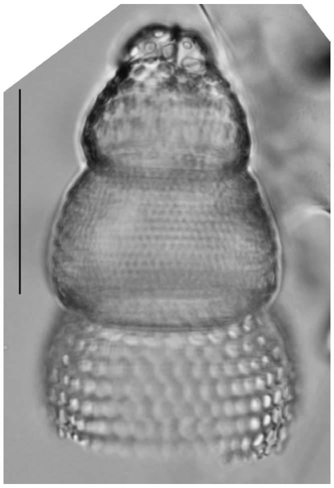

unknown artostrobiid sp 1

Artostrobiidae

Undescribed

Records: 10

Abdominal segment is tapered, distinguishing this species from S. subscalaris cf.

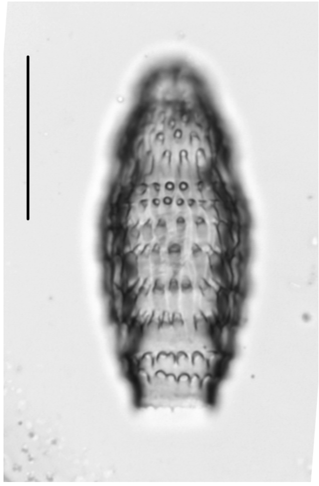

unknown artostrobiid sp 2

Artostrobiidae

Undescribed

Records: 52

Superficially similar to S. lineata, except that shell is significantly thicker, and pores tend to be irregularly distributed.

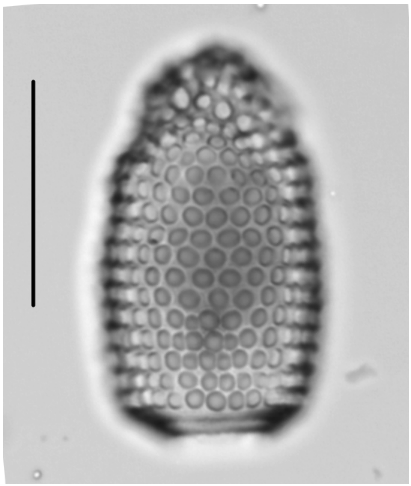

unknown artostrobiid sp 3

Artostrobiidae

Undescribed

Records: 21

This species only has one segment after the cephalis, making it unique from all other artostrobiids identified here, and distinguishing it from the superficially similar, P. crustula, which has a similar shape, but always exhibits two segments after the cephalis.

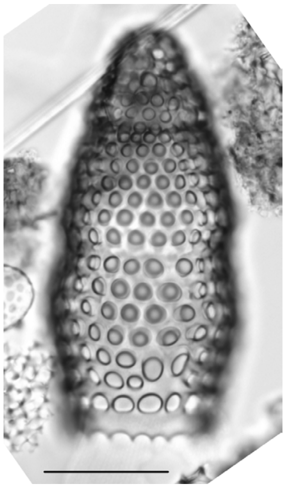

unknown artostrobiid sp 4

Artostrobiidae

Undescribed

Records: 10

This species is thick-walled and has three segments that get progressively longer with distance from the cephalis. Pores are large.

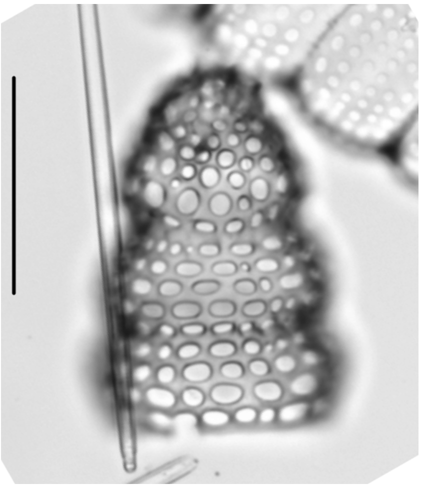

unknown artostrobiid sp 5

Artostrobiidae

Undescribed

Records: 20

Resembles S. gyroscalaris in that pores are somewhat rectangular or stretched in shape. But it differs by being generally thicker-walled, more narrow in outline, and having larger pores.

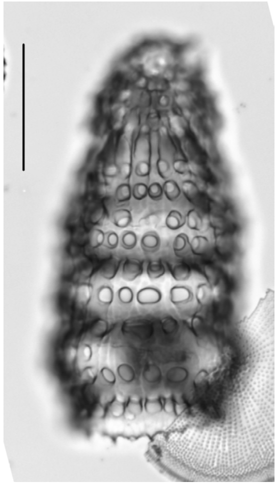

unknown artostrobiid sp 6

Artostrobiidae

Undescribed

Records: 31

This species has very large pores and very thick walls. Only ~2 rows of pores per segment. Extended area without pores between first and second thoracic segments.

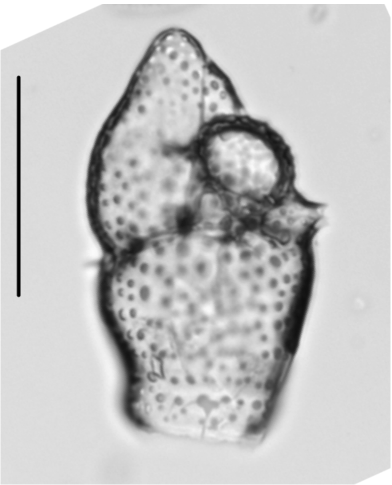

Acrobotrissa cribrosa

Cannobotryidae

Popofsky, 1913

Records: 70

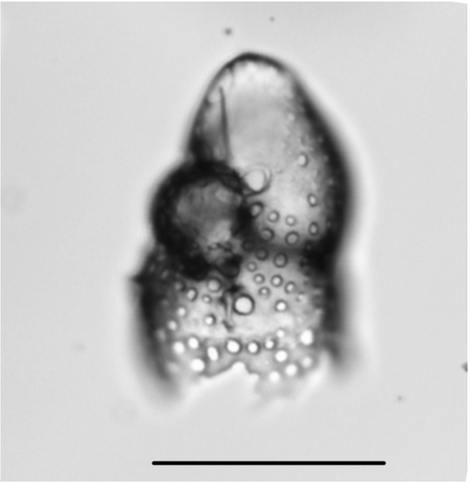

Acrobotrissa cribrosa cf

Cannobotryidae

Undescribed

Records: 49

Lacking a tube near the base of cephalis, but otherwise identical to A. cribrosa

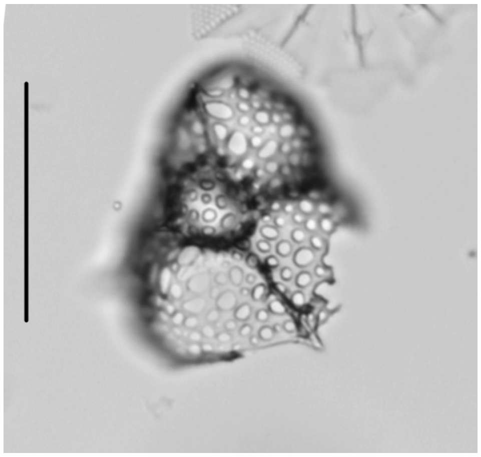

Acrobotrys chelinobotrys

Cannobotryidae

Takahashi, 1991

Records: 47

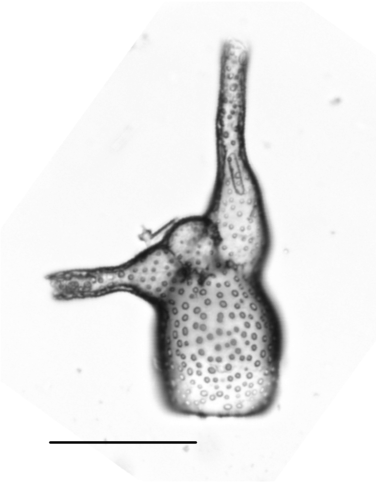

Acrobotrys disolenia

Cannobotryidae

Haeckel, 1887

Records: 57

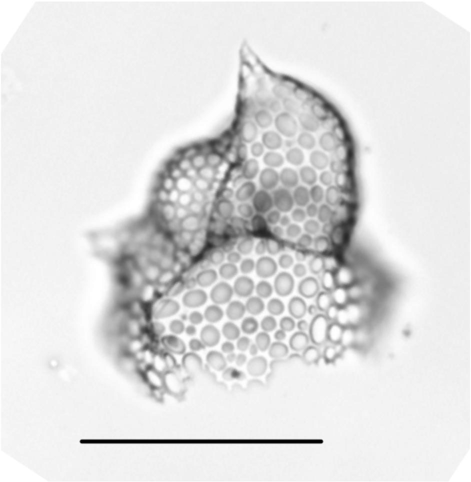

Acrobotrys teralans

Cannobotryidae

Renz, 1976

Records: 56

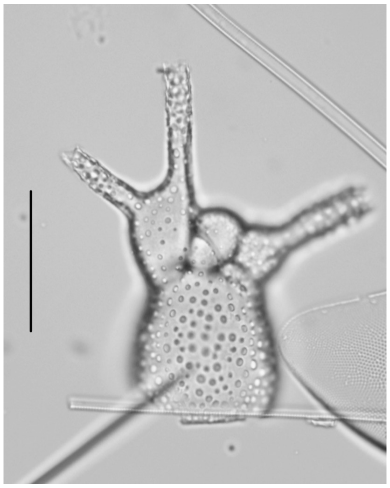

Acrobotrys tritubus
Cannobotryidae
Riedel, 1957
Records: 16

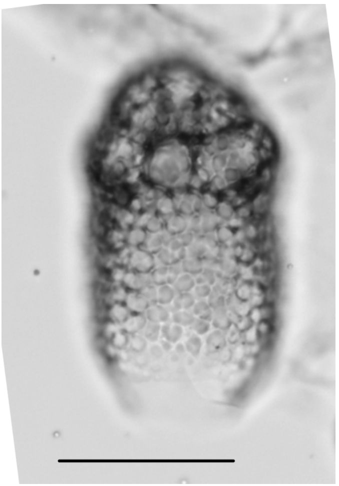

Amphimelissa setosa cf
Cannobotryidae
(Cleve) Bernstein, 1934
Records: 77

This species differs from Amphimelissa setosa in that it has a greater number of cephalic lobes. It is distinguished from Botryostrobus scutum in that it lacks abdominal segments.

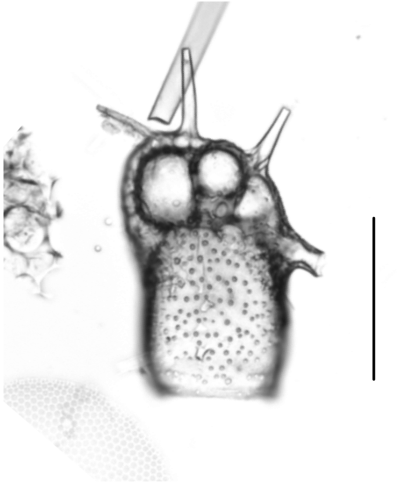

Botryocyrtis elongatum
Cannobotryidae
Takahashi, 1991
Records: 58

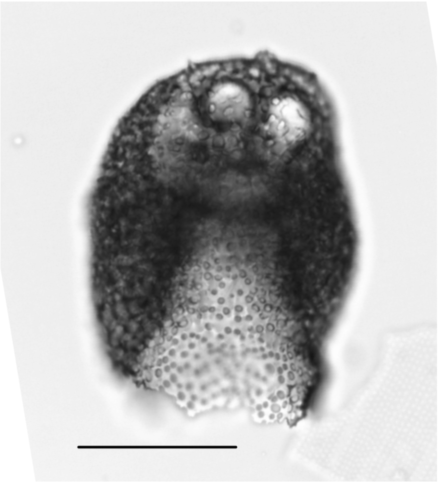

Botryocyrtis scutum
Cannobotryidae
(Harting) Nigrini, 1967
Records: 60

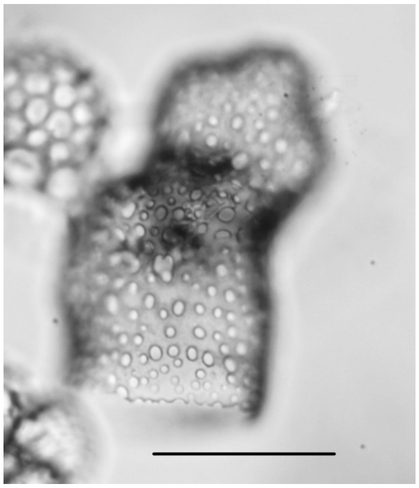

Centrobotrys petrushevskayae
Cannobotryidae
Sanfilippo & Riedel, 1973
Records: 5

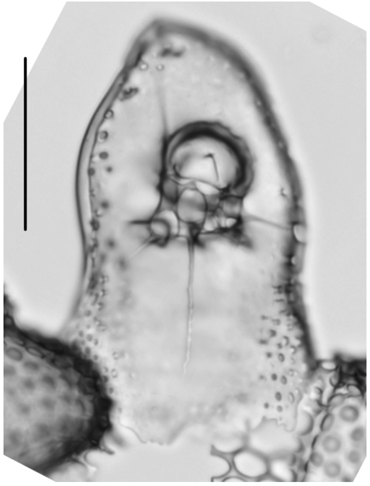

Centrobotrys thermophila
Cannobotryidae
Petrushevskaya, 1965
Records: 41

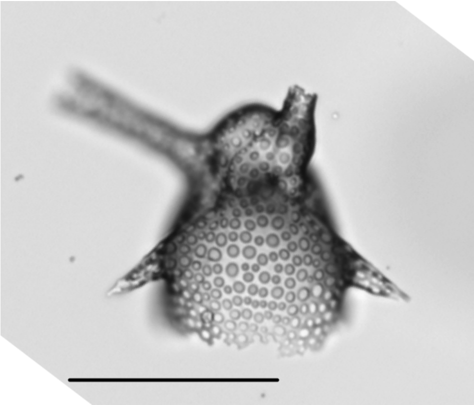

Neobotrys quadrituberosa

Cannobotryidae

Popofsky, 1913

Records: 20

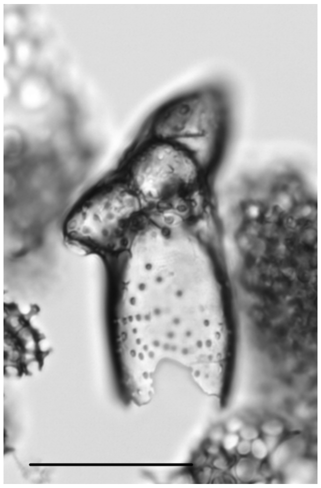

unknown cannobotryid sp 10

Cannobotryidae

Undescribed

Records: 8

This species has two sub-horizontally oriented cephalic lobes, pointed ~180 degrees from one another off the central lobe.

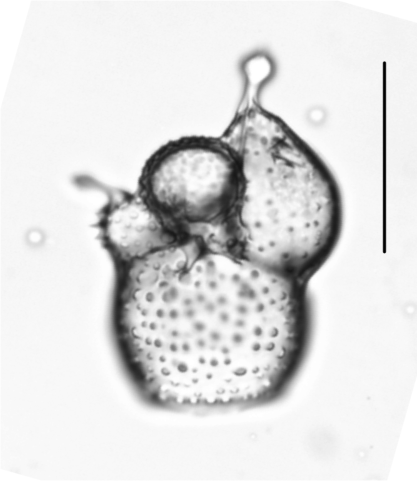

unknown cannobotryid sp 11

Cannobotryidae

Undescribed

Records: 13

Similar to A. cribrosa, except that instead of a lower cephalic tube, this species has a lobe with a protruding spine. The upper lobe also has a strong spine, often with a distinct rounded tip.

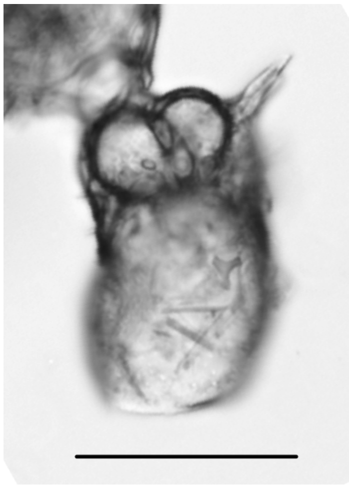

unknown cannobotryid sp 12

Cannobotryidae

Undescribed

Records: 8

This species likely belongs to the genus Saccospyris, as it has only two cephalic lobes. Otherwise, its shape is overall similar to B. elongatum, with at least two spines coming out of the cephalis, and one tube pointing slightly downward of horizontal.

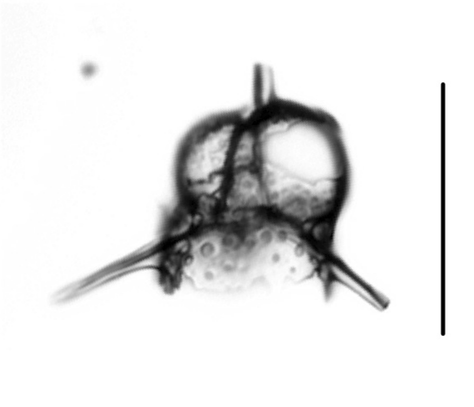

unknown cannobotryid sp 13

Cannobotryidae

Undescribed

Records: 4

This species differs from unknown cannobotryid sp 12 in that it lacks a long tube on the side of the cephalis, and instead has two downward-pointing bladed spines. This species likely belongs to the Saccospyris genus.

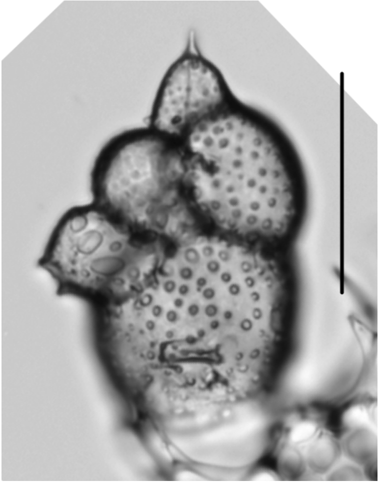

unknown cannobotryid sp 2

Cannobotryidae

Undescribed

Records: 51

This species resembles A. cribrosa, except that the large/main cephalic lobe is subdivided into two inflated parts. This species may be synonymous with Acrobotrys acuminata (Haeckel).

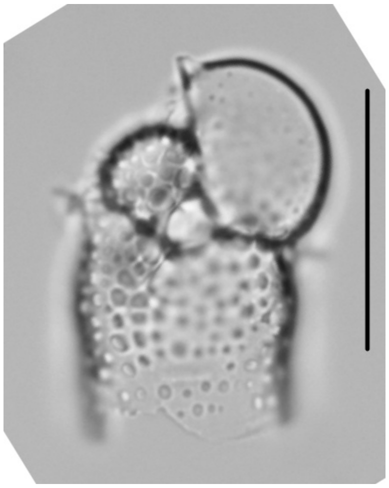

unknown cannobotryid sp 4

Cannobotryidae

Undescribed

Records: 53

This species has a relatively small, often hyaline main cephalic lobe, which is half-moon-shaped and has spines protruding vertically and horizontally out of it.

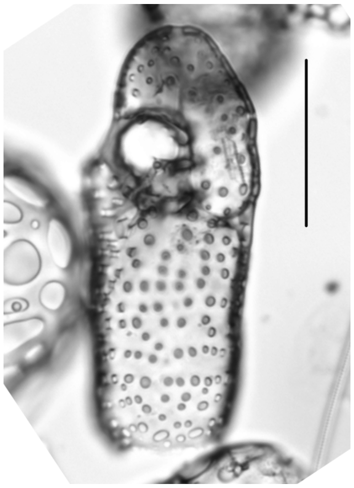

unknown cannobotryid sp 5

Cannobotryidae

Undescribed

Records: 39

This species has an elongated thorax, and a wide, compressed cephalic lobe, vaguely resembling C. thermophila, except that the cephalis is not hyaline.

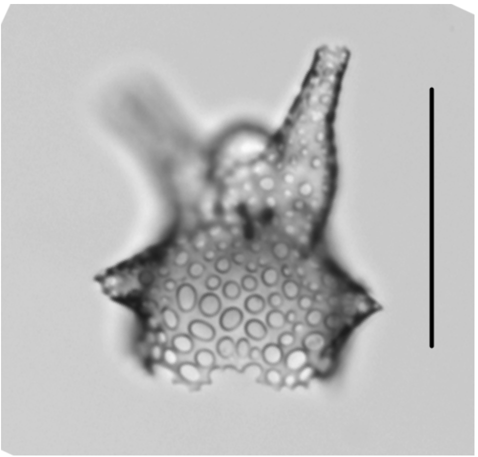

unknown cannobotryid sp 7

Cannobotryidae

Undescribed

Records: 19

This species resembles N. quadrituberosa, except that the upper two tubes are oriented more directly upward, and the bottom two protrusions are spines rather than tubes.

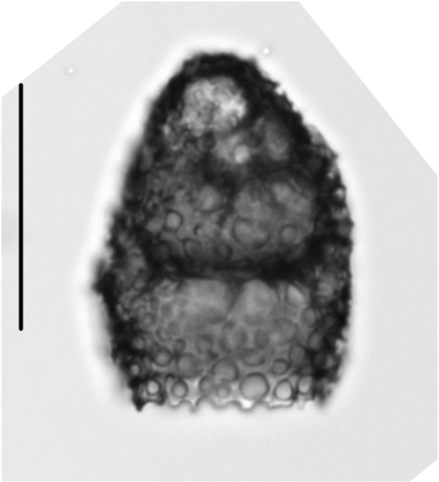

unknown cannobotryid sp 8

Cannobotryidae

Undescribed

Records: 12

This species has a reduced cephalis, with only one small lobe. Thorax and abdomen otherwise resemble B. scutum.

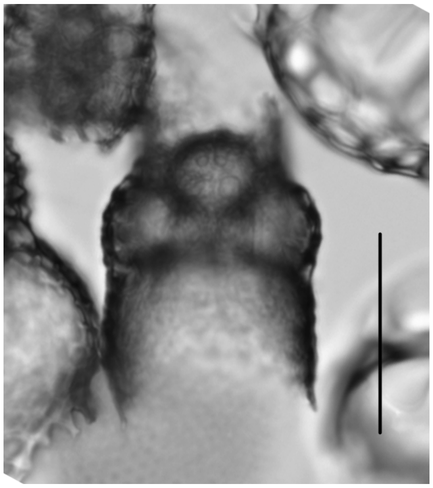

unknown cannobotryid sp 9

Cannobotryidae

Undescribed

Records: 9

This species has a distinct tri-lobate cephalis and no tubes. However, no complete specimens have been found.

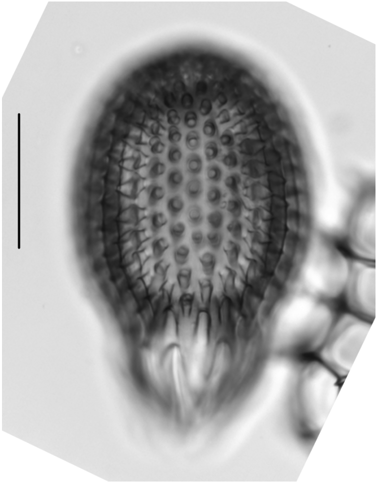

Carpocanistrum acutidentatum

Carpocaniidae

Takahashi, 1991

Records: 92

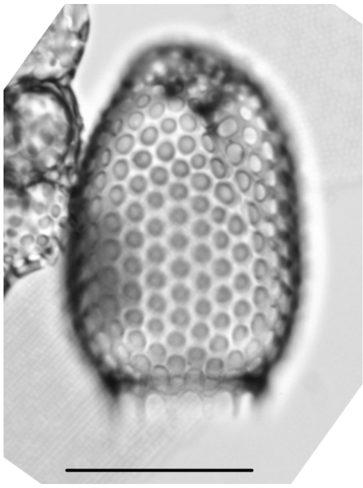

Carpocanistrum cephalum
Carpocaniidae
Haeckel, 1887
Records: 93

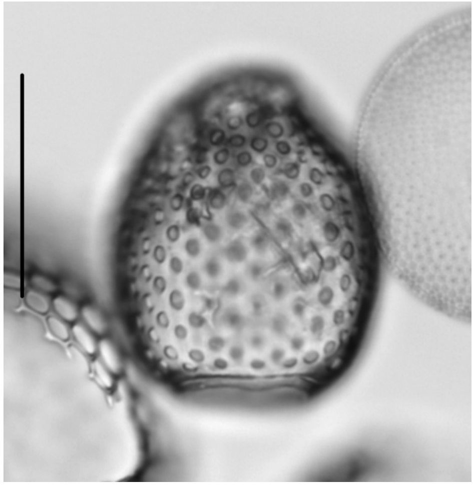

Carpocanistrum coronatum
Carpocaniidae
(Ehrenberg) Takahashi, 1991
Records: 58

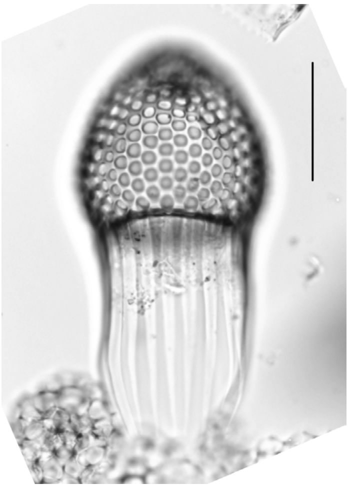

Carpocanistrum favosum
Carpocaniidae
(Haeckel) Takahashi, 1991
Records: 116

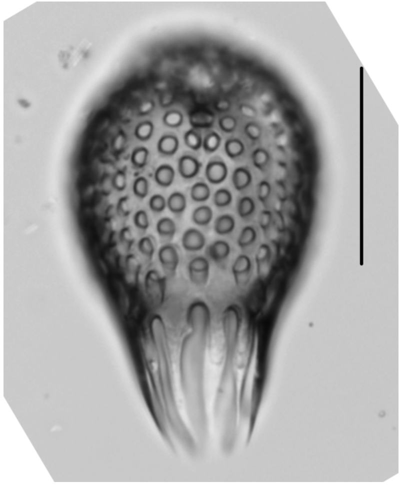

Carpocanistrum flosculum
Carpocaniidae
Haeckel, 1887
Records: 81

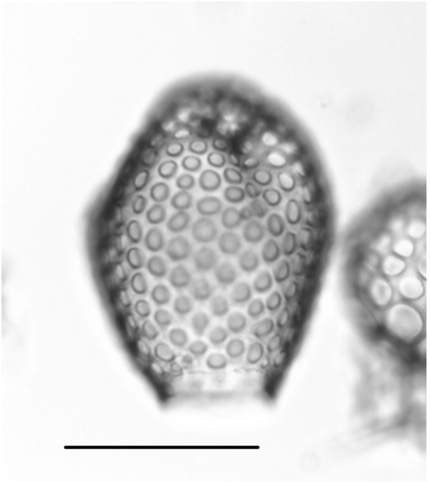

Carpocanistrum flosculum cf
Carpocaniidae
Undescribed
Records: 75

Like C. flosculum, except lacking any indication of spines/teeth on the bottom of the shell. Differentiated from C. coronatum by more elongated thorax.

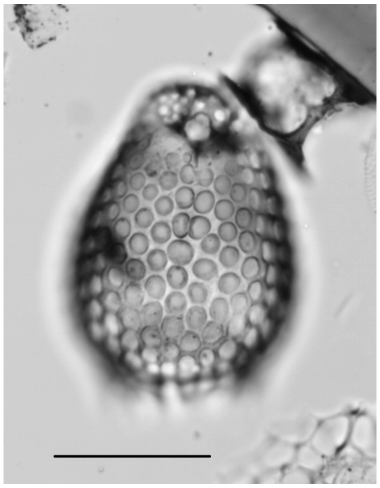

Carpocanistrum sp. A
Carpocaniidae
Nigrini, 1970
Records: 24

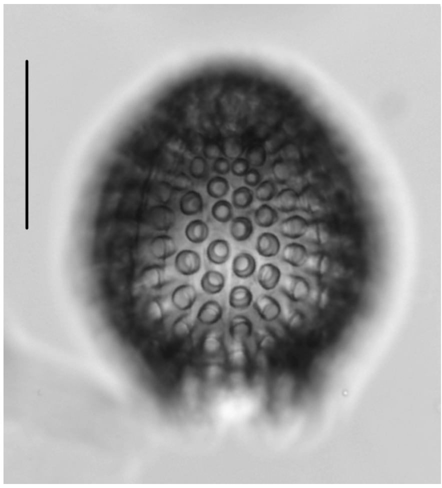

Carpocanium kinugasense
Carpocaniidae
Nishimura, 1990
Records: 15

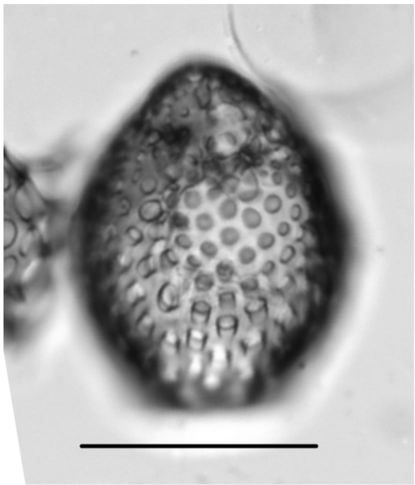

Carpocanium? uberex
Carpocaniidae
Renaudie & Lazarus, 2012
Records: 6

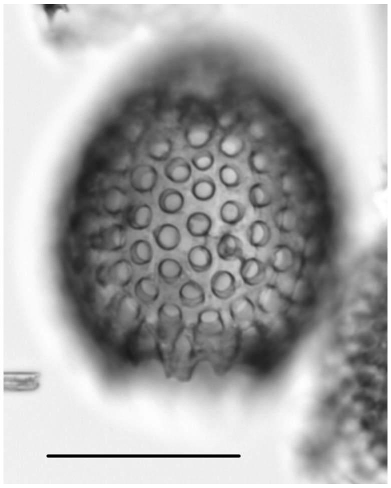

Carpocanopsis cristatum
Carpocaniidae
Carnevale, 1908
Records: 56

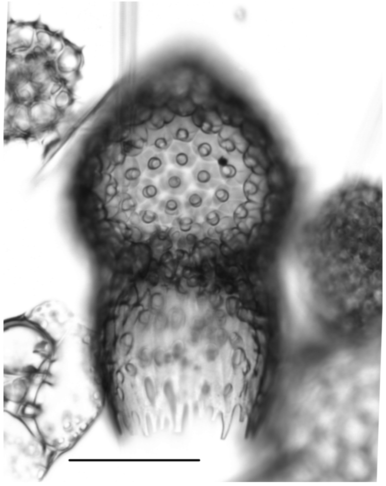

Carpocanopsis sp 1
Carpocaniidae
Undescribed
Records: 39

Distinct wrinkly framing around thoracic pores. Abdomen with "stretched" pores and often patch-es of hyaline area. Teeth at the end of abdomen.

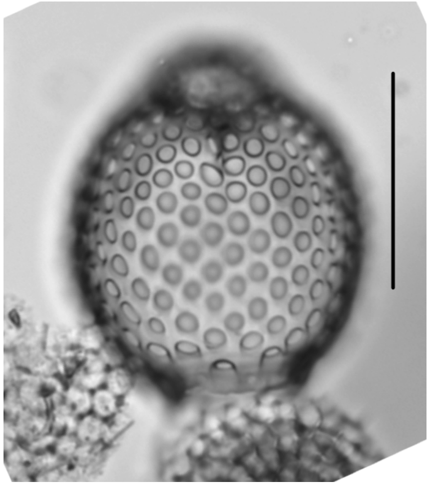

Plannapus hornibrooki
Carpocaniidae
O'Connor, 1999
Records: 27

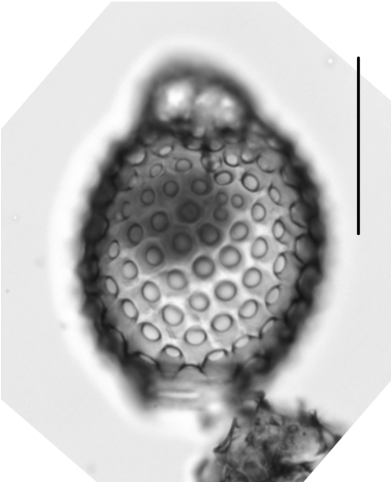

Plannapus maurisi
Carpocaniidae
O'Connor, 1999
Records: 16

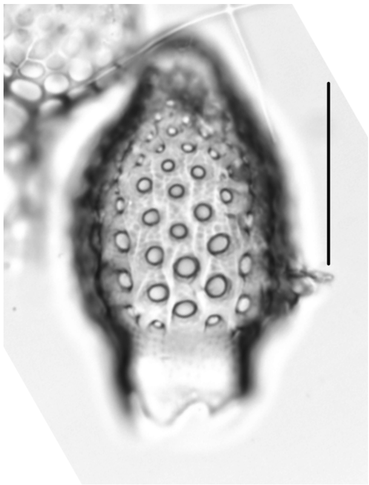

Plannapus papillosus

Carpocaniidae

(Ehrenberg) O'Connor, 1999

Records: 87

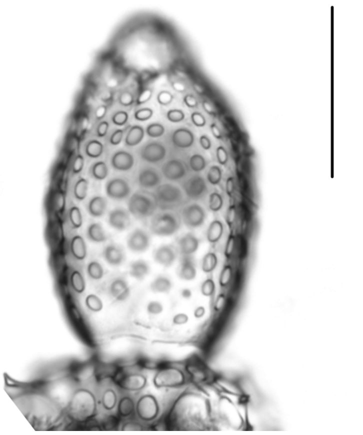

Plannapus papillosus cf sp 1

Carpocaniidae

Undescribed

Records: 76

Similar to P. papillosus, except that thorax is elongated, tends to have a smoother exterior, and usually has smaller pores than the originally-described species. x

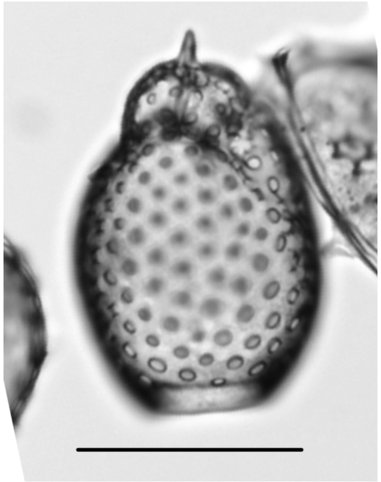

Plannapus papillosus cf sp 3

Carpocaniidae

Undescribed

Records: 104

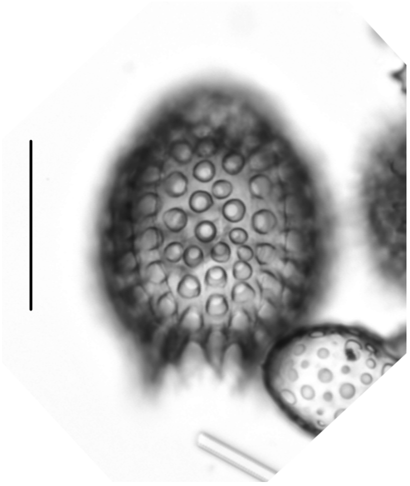

unknown carpocaniid sp 1

Carpocaniidae

Undescribed

Records: 57

Small, with irregular pore distribution, constricted base of thorax, teeth at base

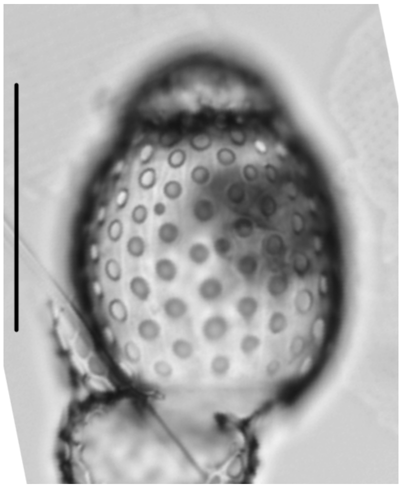

unknown carpocaniid sp 6

Carpocaniidae

Undescribed

Records: 86

Flat cephalis, thin-walled, linear arrangement of pores

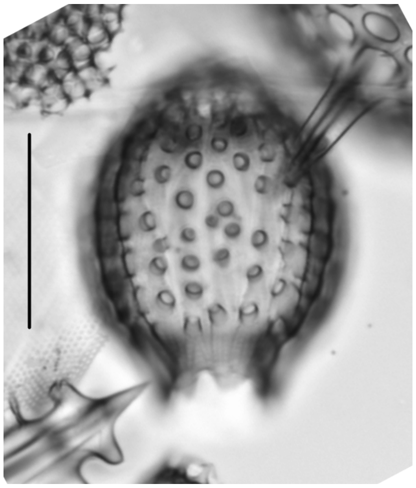

unknown carpocaniid sp 7

Carpocaniidae

Undescribed

Records: 9

Irregular pores, irregular ridges on thorax

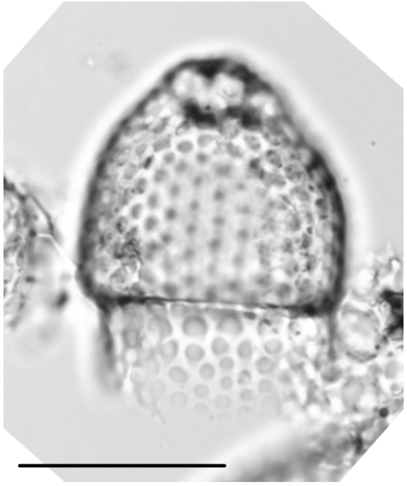

unknown carpocaniid sp 8

Carpocaniidae

Undescribed

Records: 21

Shape similar to C. favosum, except instead of terminal spines/teeth, this species has a clear latticed abdomen with similar pore structure to thorax.

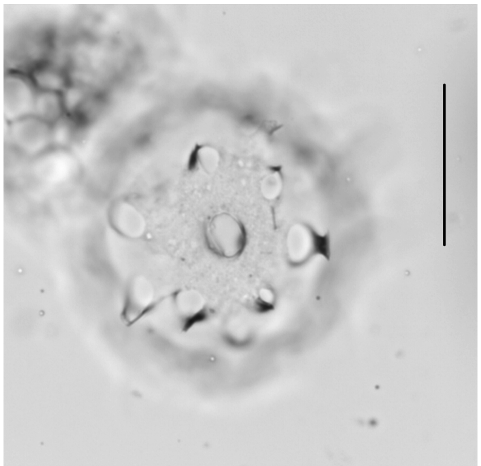

Acrosphaera arktios

Collosphaeridae

Nigrini, 1970

Records: 12

Sensu Motoyama and Nishimura 2005

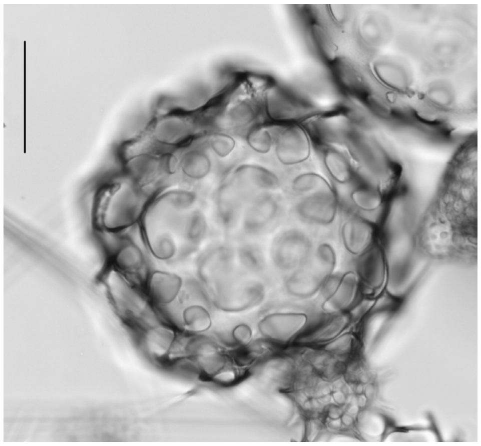

Acrosphaera circumtexta

Collosphaeridae

Strelkov & Reshetnyak, 1971

Records: 52

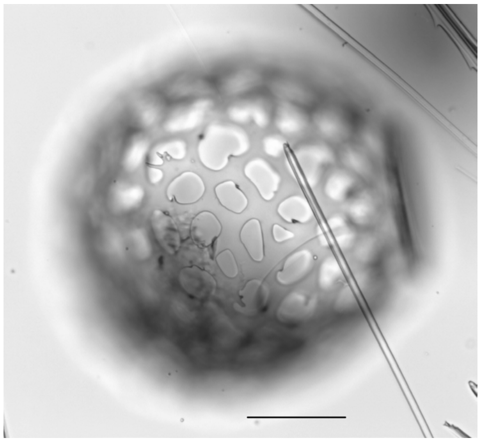

Acrosphaera cuniculiauris

Collosphaeridae

Renaudie & Lazarus, 2012

Records: 11

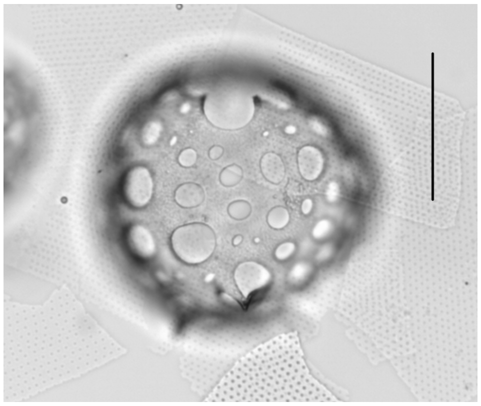

Acrosphaera cyrtodon

Collosphaeridae

(Haeckel) Strelkov & Reshetnyak, 1971

Records: 20

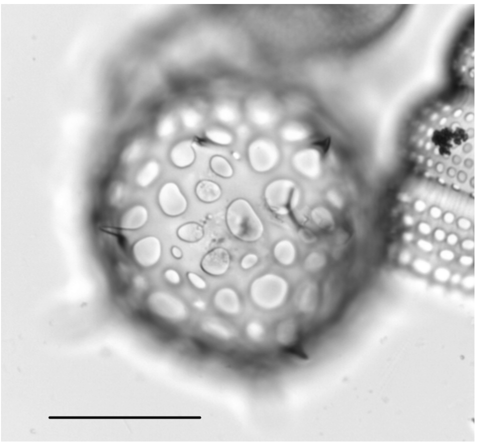

Acrosphaera cyrtodon cf

Collosphaeridae

Undescribed

Records: 114

Like A. cyrtodon, except with significantly more spines and slightly smaller pores.

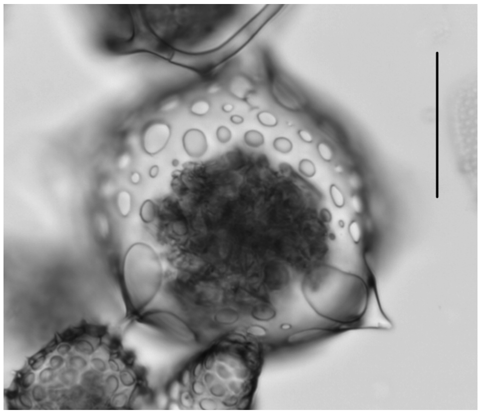

Acrosphaera cyrtodon cf sp 2

Collosphaeridae

Undescribed

Records: 43

Similar to A. cyrtodon, except that spines are surrounded by a set of enlarged pores.

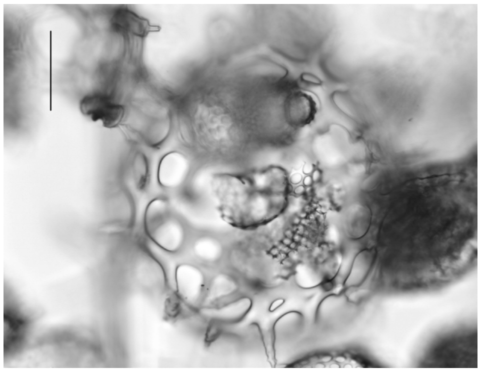

Acrosphaera inflata

Collosphaeridae

Haeckel, 1887

Records: 14

Pl. 5, fig. 7

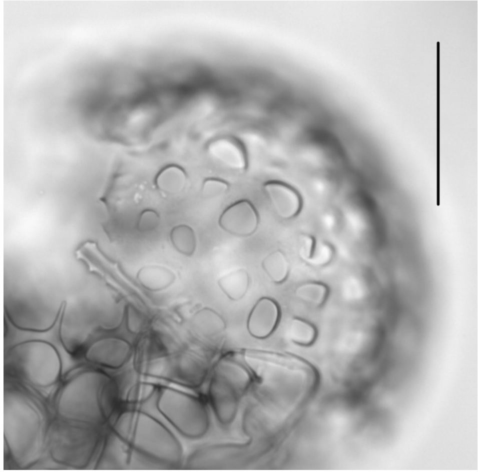

Acrosphaera labrata

Collosphaeridae

Lazarus, 1992

Records: 5

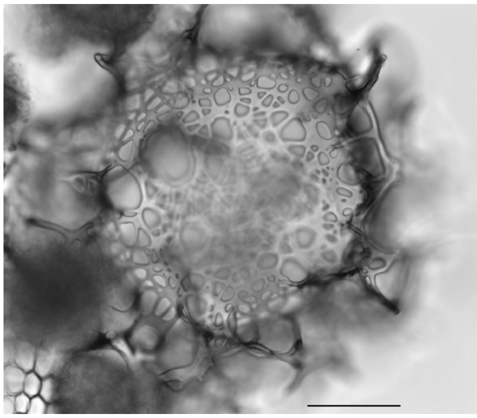

Acrosphaera lappacea

Collosphaeridae

(Haeckel) Johnson & Nigrini, 1980

Records: 22

sensu Strelkov and Reshetnyak

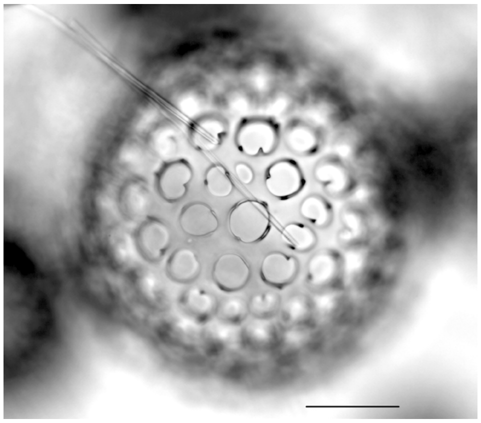

Acrosphaera murrayana

Collosphaeridae

(Haeckel) Hilmers, 1906

Records: 88

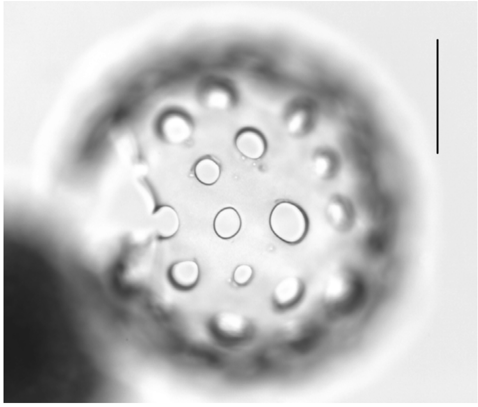

Acrosphaera murrayana smooth form

Collosphaeridae

Undescribed

Records: 60

Like A. murrayanna, except that the raised pores lack teeth

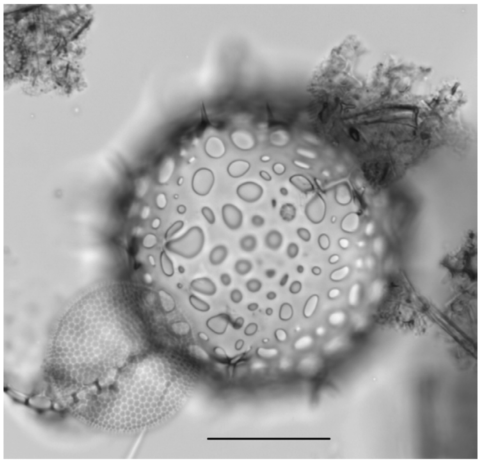

Acrosphaera spinosa
Collosphaeridae
Haeckel, 1861
Records: 76

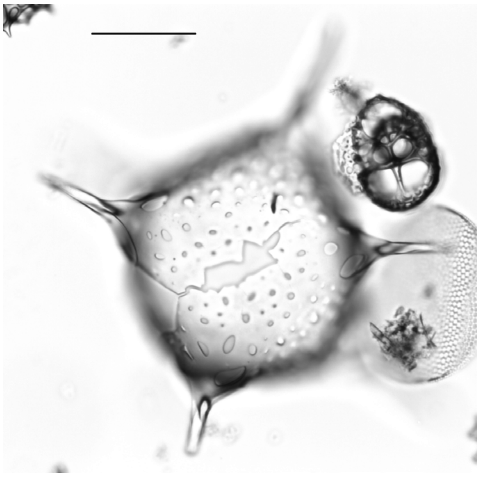

Acrosphaera spinosa fasciculopora
Collosphaeridae
Caulet, 1986
Records: 41

Acrosphaera spinosa with 10-15 spines, strong, long, arising from a cluster of pores that are larger than the irregular small pores of the shell and that form small protuberances on the shell surface.

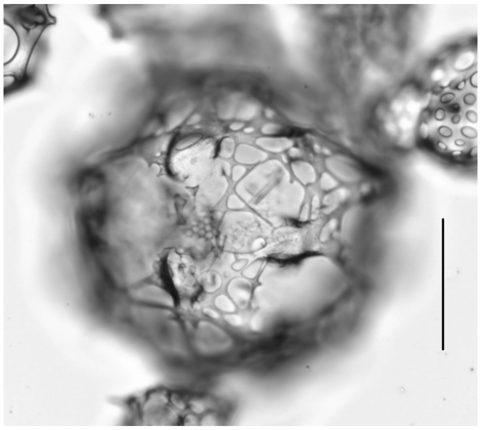

Clathrosphaera arachnoides
Collosphaeridae
Haeckel, 1887
Records: 18

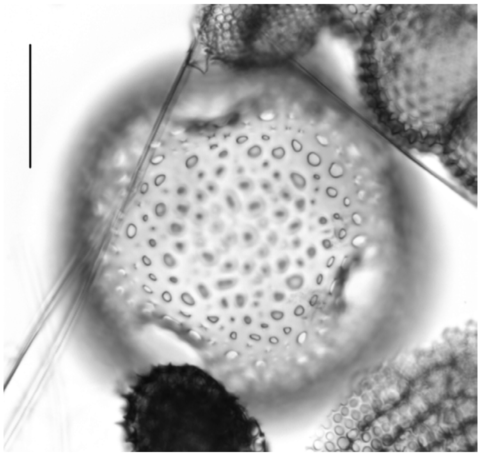

Collosphaera brattstroemi
Collosphaeridae
Bjørklund & Goll, 1979
Records: 42

sensu Kamikuri 2019

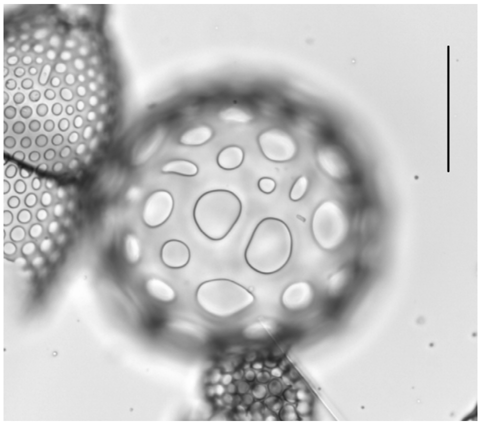

Collosphaera hux/mac
Collosphaeridae
Undescribed
Records: 36

Intermediate between C. huxleyi and C. macropora in terms of pore size.

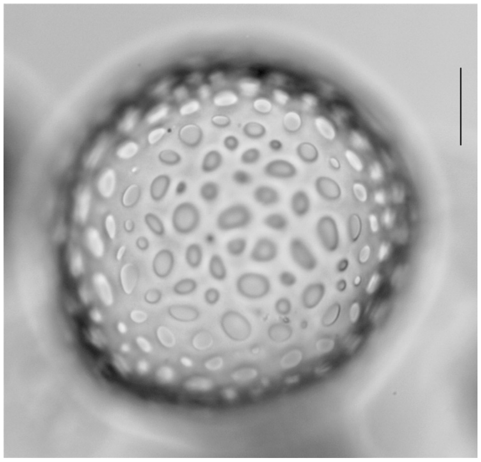

Collosphaera huxleyi
Collosphaeridae
Mueller, 1855
Records: 22

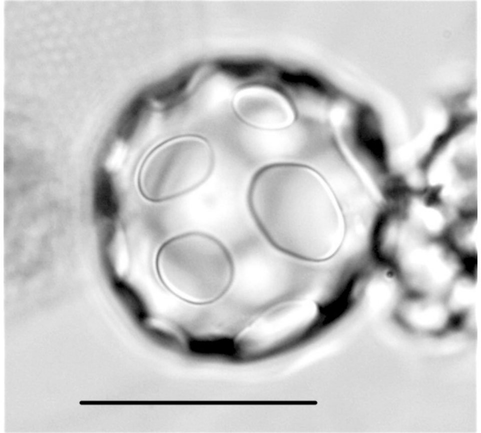

Collosphaera macropora
Collosphaeridae
Popofsky, 1917
Records: 29

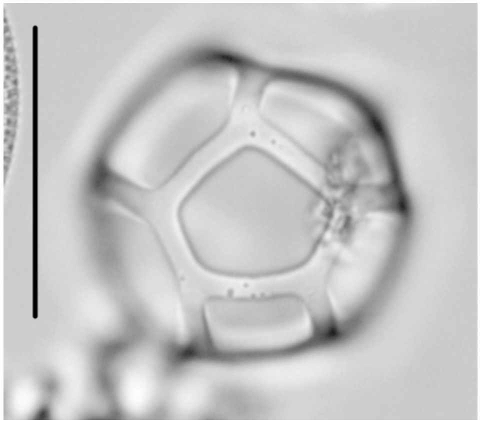

Collosphaera macropora cf sp 2
Collosphaeridae
Undescribed
Records: 13

Like C. macropora, except with very angular pores, which are often pentagonal in shape.

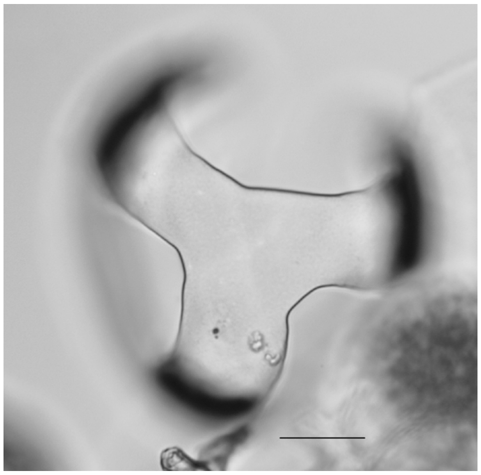

Collosphaera planca
Collosphaeridae
Su, 1982
Records: 43

Similar to C. macropora, except that this species has a reduced number of pores, typically only 3-5, and is much larger in overall size.

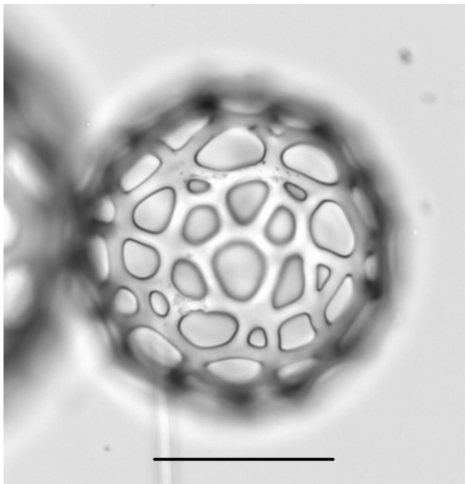

Collosphaera polygona
Collosphaeridae
Haeckel, 1887
Records: 70

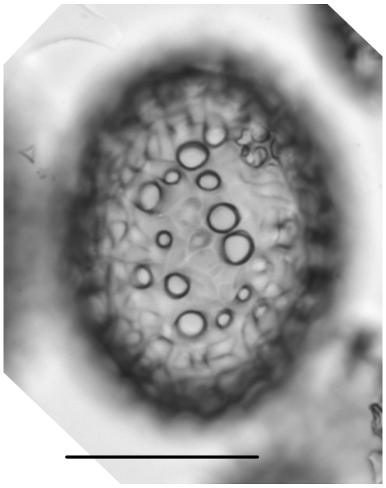

Collosphaera reynoldsi
Collosphaeridae
Kamikuri, 2010
Records: 35

Pl. 3, figs. 18–25

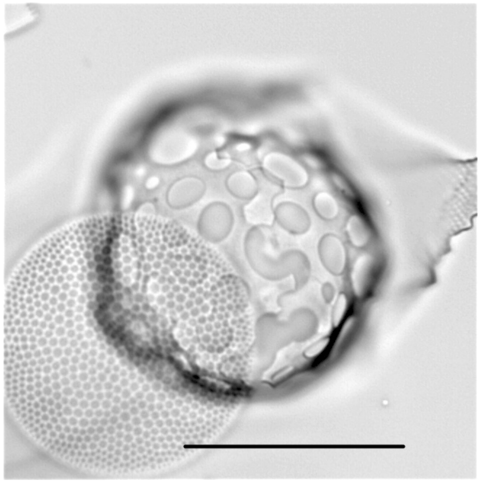

Collosphaera tuberosa cf
Collosphaeridae
Undescribed
Records: 26

This species resembles the species described by Haeckel, 1887, but differs in that it has larger, more regular pores.

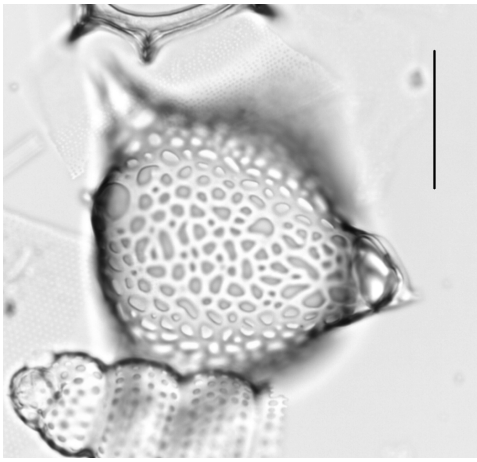

Disolenia collina
Collosphaeridae
Haeckel, 1887
Records: 21
sensu Takahashi 1991

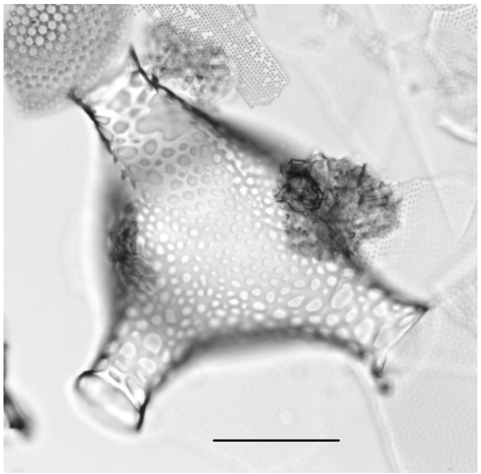

Disolenia quadrata
Collosphaeridae
(Ehrenberg) Nigrini, 1967
Records: 77

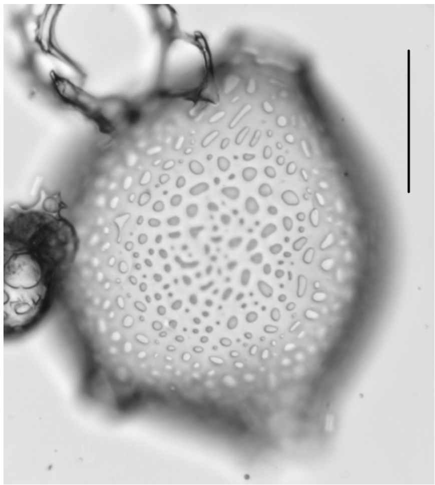

Otosphaera auriculata
Collosphaeridae
Haeckel, 1887
Records: 118

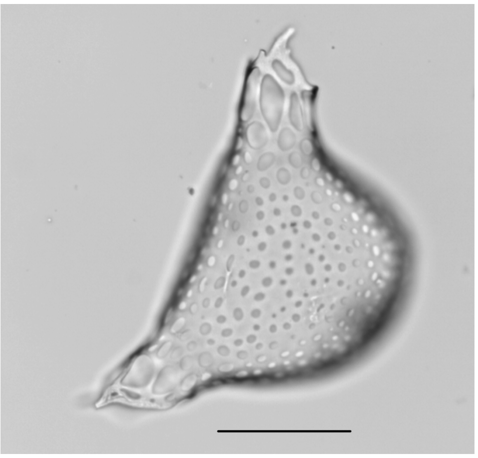

Otosphaera polymorpha
Collosphaeridae
Haeckel, 1887
Records: 174
sensu Takahashi 1991

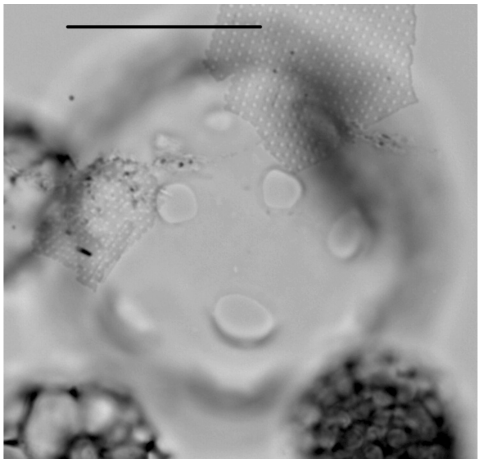

Siphonosphaera abelmannae
Collosphaeridae
Renaudie & Lazarus, 2012
Records: 12

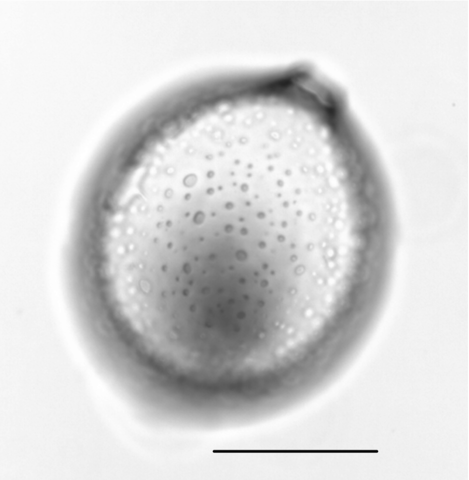

Siphonosphaera hyalina
Collosphaeridae
Caulet, 1986
Records: 21

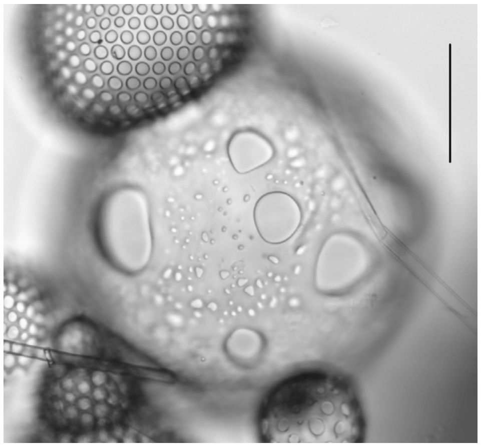

Siphonosphaera magnisphaera
Collosphaeridae
Takahashi 1991
Records: 35

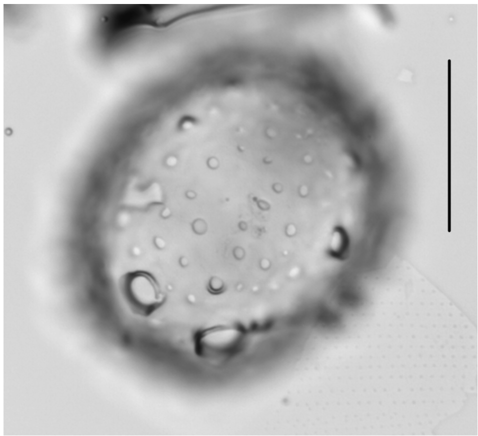

Siphonosphaera martensi
Collosphaeridae
Brandt, 1905
Records: 15

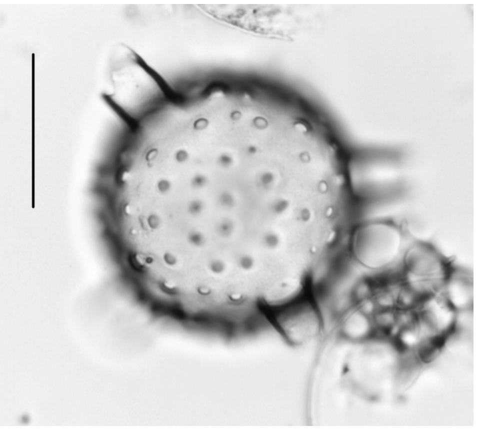

Siphonosphaera socialis
Collosphaeridae
Haeckel, 1887
Records: 28

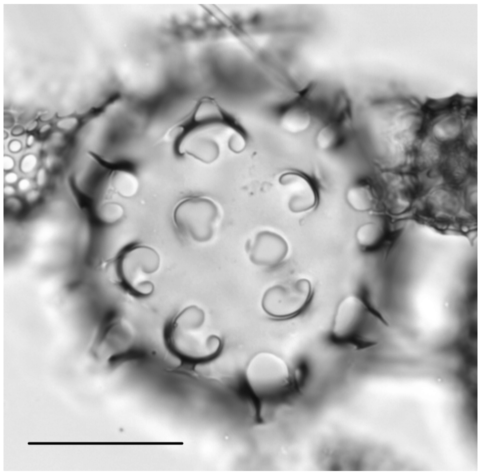

Siphonosphaera sp. C Kamikuri
Collosphaeridae
Kamikuri, 2017
Records: 34

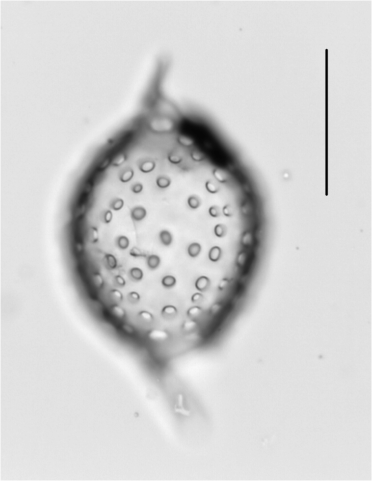

Solenosphaera chierchiae
Collosphaeridae
Strelkov & Reshetnyak, 1971
Records: 53

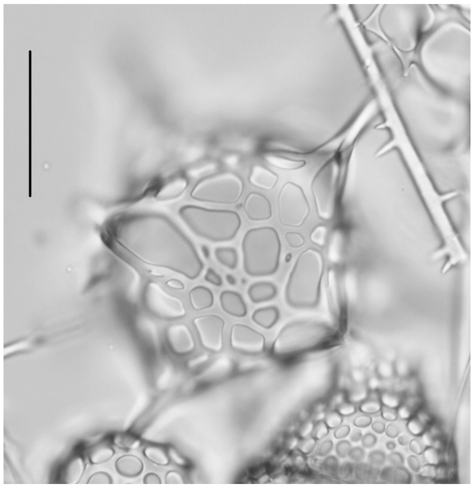

Solenosphaera inflata
Collosphaeridae
Strelkov & Reshetnyak, 1971
Records: 31

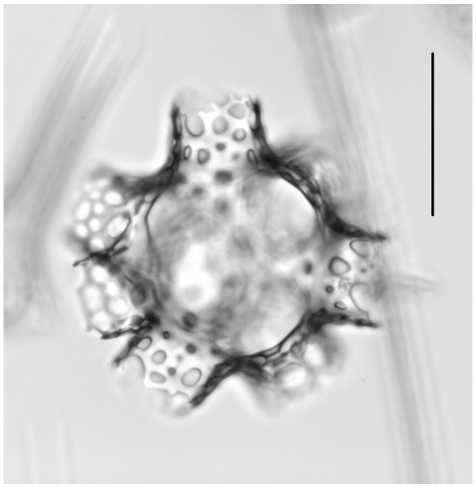

Solenosphaera omnitubus
Collosphaeridae
Riedel & Sanfilippo, 1971
Records: 37

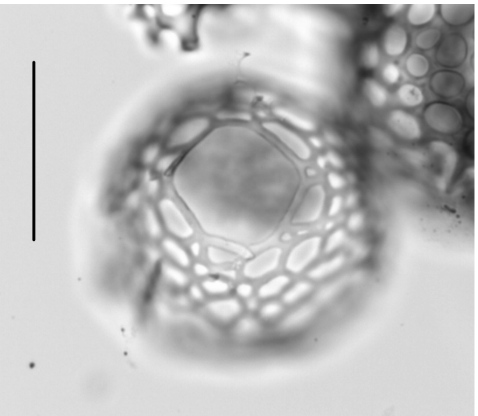

Solenosphaera zanguebarica
Collosphaeridae
Brandt, 1905
Records: 76

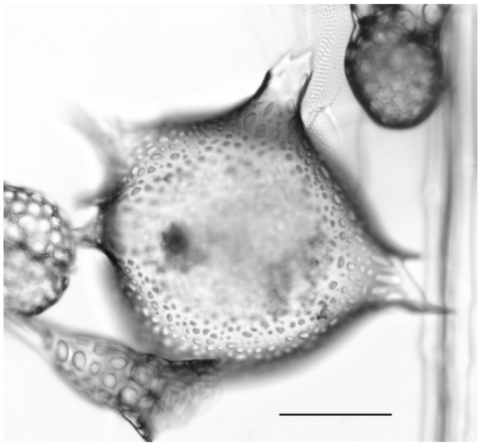

Trisolenia combinata
Collosphaeridae
Bjørklund & Goll, 1979
Records: 12

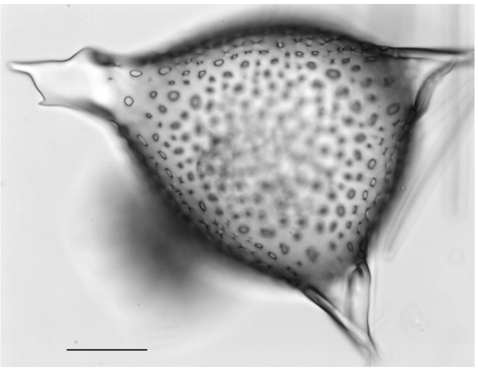

Trisolenia megalactis
Collosphaeridae
Ehrenberg, 1873
Records: 12

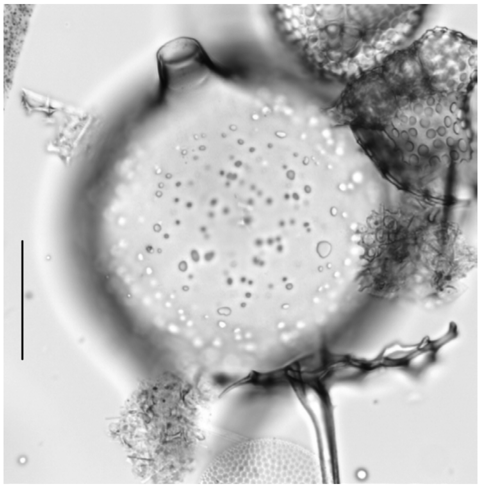

unknown collosphaerid sp 10
Collosphaeridae
Undescribed
Records: 23

This species has small, scattered pores and ~5-6 robust, hyaline tubes.

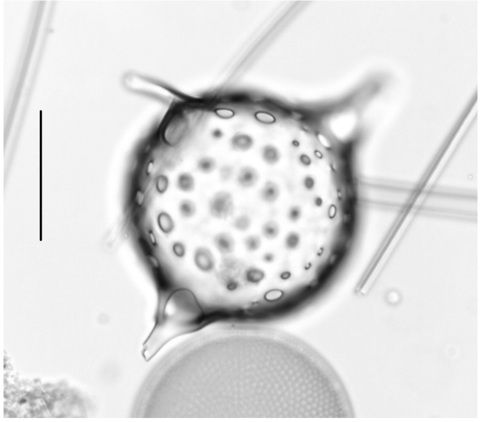

unknown collosphaerid sp 11
Collosphaeridae
Undescribed
Records: 32

This species is relatively small, with regular, medium-sized pores and ~6 curved spines originating from sets of raised and enlarged pores.

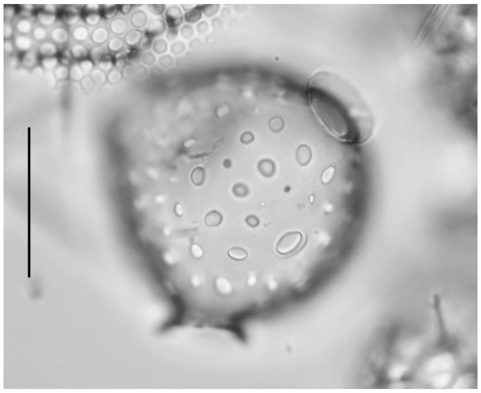

unknown collosphaerid sp 2

Collosphaeridae

Undescribed

Records: 38

This species has distinctive sucker-shaped tubes.

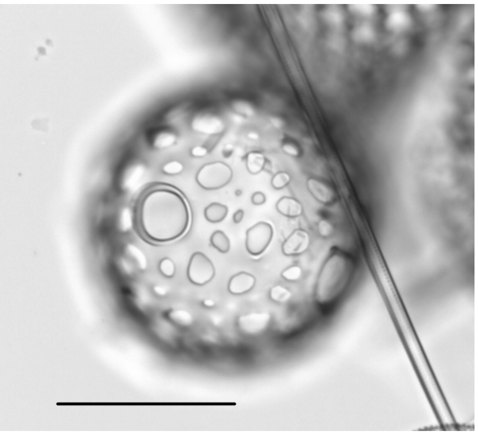

unknown collosphaerid sp 4

Collosphaeridae

Undescribed

Records: 66

This species is defined by sporadic large pores that have rings of extra silica, forming a distinct "lip" structure.

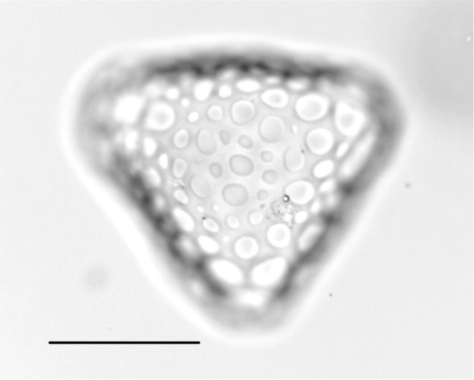

unknown collosphaerid sp 5

Collosphaeridae

Undescribed

Records: 54

This species is consistently triangular in shape, with no tubes or spines, but a tendency to have slightly larger pores at the tips of the triangle, that sometimes suggest an opening.

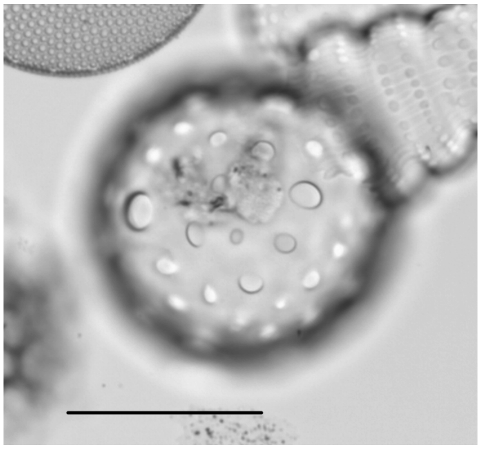

unknown collosphaerid sp 6

Collosphaeridae

Undescribed

Records: 27

This species is small, with many small, slightly raised pores.

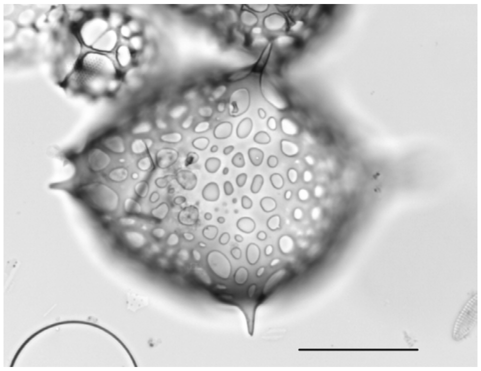

unknown collosphaerid sp 8

Collosphaeridae

Undescribed

Records: 29

This species is irregularly shaped, with spines rising from enlarged pores. Differs from A. cyrtodon q in that it has a more polygonal overall shape, rather than being round. Differs from D. collina in that pores are more round.

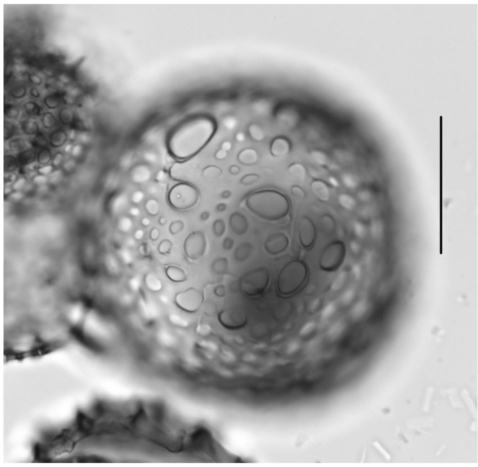

unknown collosphaerid sp 9

Collosphaeridae

Undescribed

Records: 44

This species is relatively large, with many oval-shaped raised pores that suggest tube bases

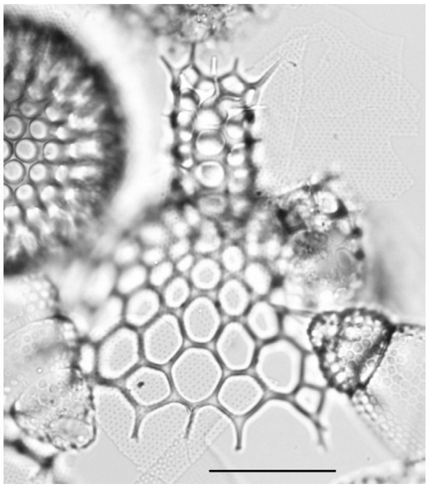

Amphiplecta acrostoma
Plagiacanthidae
Haeckel, 1887
Records: 26

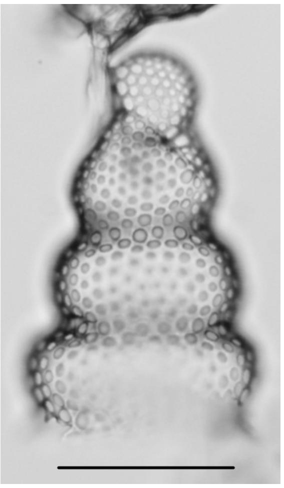

Artopilium undulatum
Plagiacanthidae
Popofsky, 1913
Records: 74

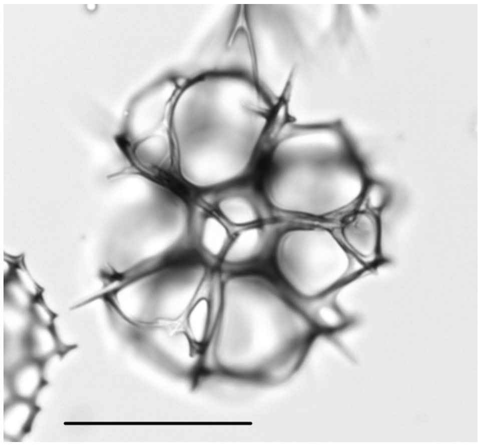

Helotholus vema
Plagiacanthidae
Hays, 1965
Records: 40

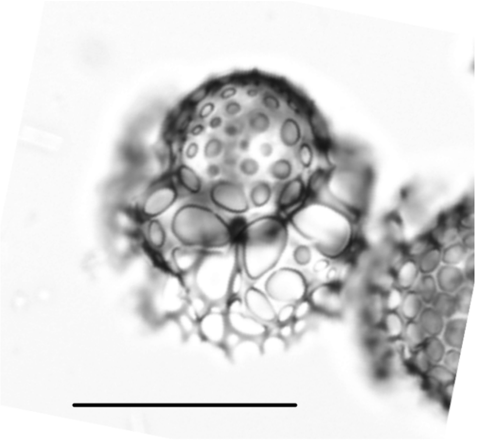

Helotholus warreni
Plagiacanthidae
Goll, 1980
Records: 48

Pl. 3, figs. 5-6

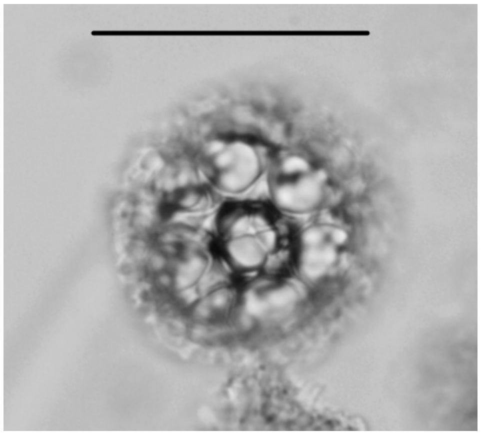

Steganocubus incrassatus
Plagiacanthidae
Funakawa, 1995
Records: 26

Pl. 9, figs. 1-7

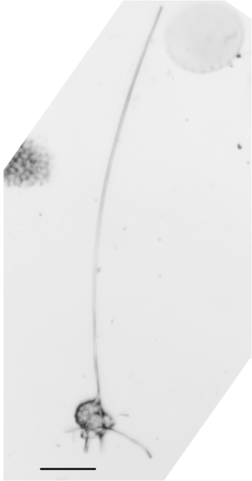

Tetracorethra tetracorethra
Plagiacanthidae
Haeckel, 1887
Records: 10

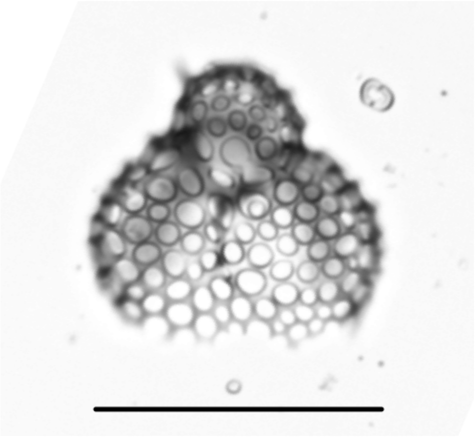

Trisulcus sp 1
Plagiacanthidae
Undescribed
Records: 9

Differs from T. triacanthus in that lobes are not as pronounced, and have the same sized pores throughout thorax.

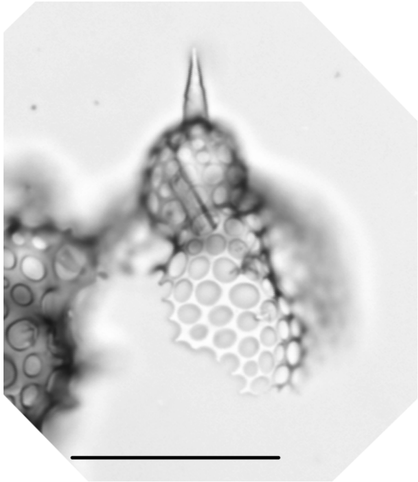

Trisulcus sp 2
Plagiacanthidae
Undescribed
Records: 12

This species differs from T. triacanthus in that cephalis is more elongated, and pores more regular in size. Differs from Trisulcus sp 1 in that lobes extend further down the thorax.

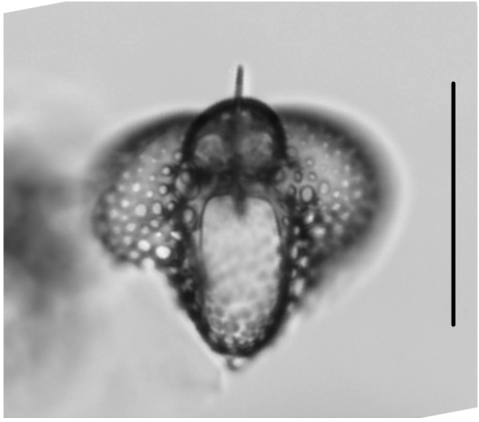

Trisulcus triacanthus
Plagiacanthidae
Popofsky, 1913
Records: 89

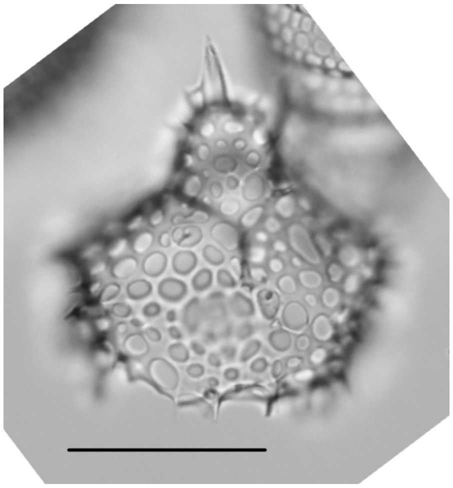

unknown plagonid group D sp 2
Plagiacanthidae
Undescribed
Records: 7

This species has a small, thumb-like cephalis, and a very broad, thorny thorax.

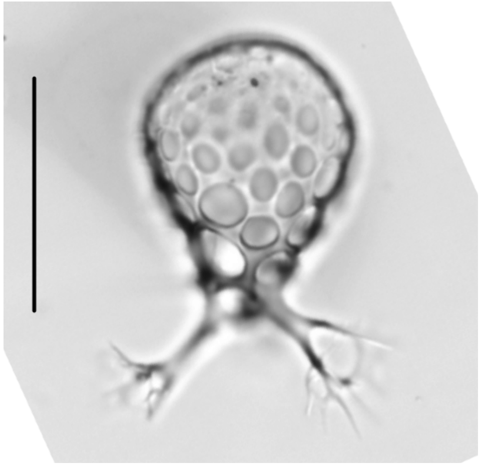

Arachnocorallium calvata
Plagiacanthidae (lophophaenid)
Petrushevskaya, 1971
Records: 115

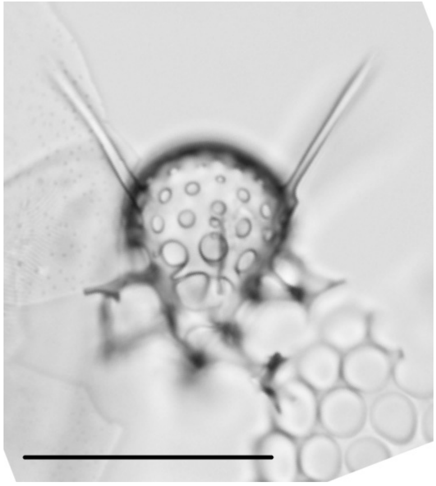

Arachnocorallium calvata cf
Plagiacanthidae (lophophaenid)
Undescribed
Records: 22

Like A. calvata, except that it has two long, conical spines on either side of the cephalis.

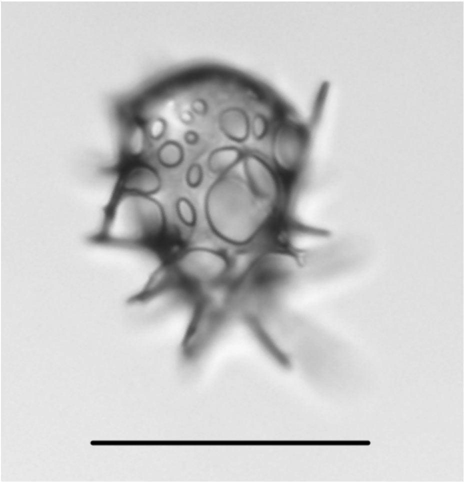

Arachnocorallium stilla

Plagiacanthidae (lophophaenid)

Renaudie & Lazarus, 2015

Records: 8

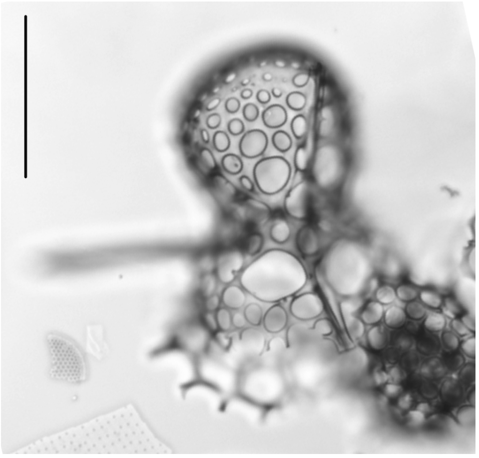

Arachnocorys pentacantha cf sp 3

Plagiacanthidae (lophophaenid)

Undescribed

Records: 96

This species resembles A. pentacantha cf sp 1, but differs in that cephalis is relatively much larger.

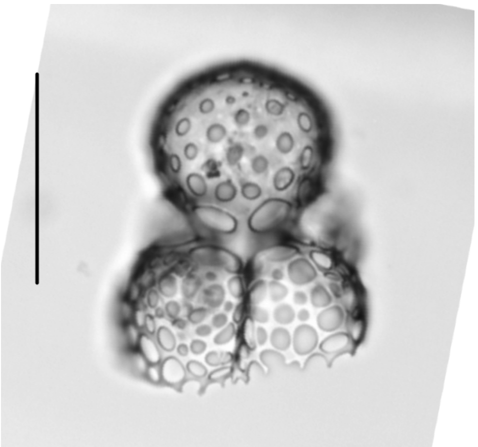

Arachnocorys? sp 2/3

Plagiacanthidae (lophophaenid)

Undescribed

Records: 32

This species has a heavy, round cephalis with regular round pores, large pores at base of cephalis and top of thorax, and wide thorax with dimples associated with spines.

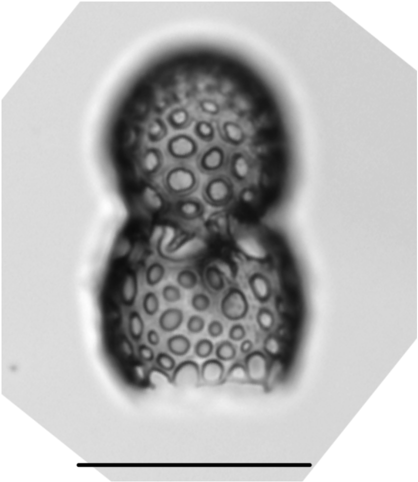

Arachnocorys? sp 4

Plagiacanthidae (lophophaenid)

Undescribed

Records: 27

This species has a narrow thorax, with large pores at the top of thorax.

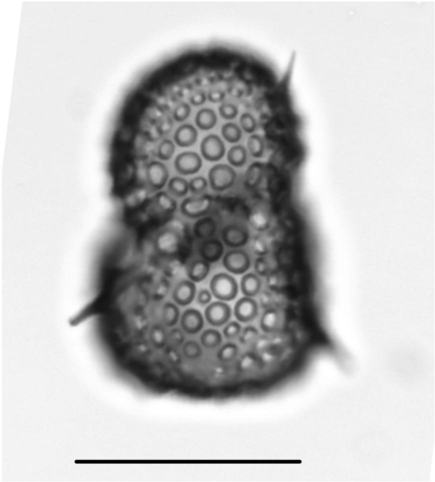

Lithomelissa brevispicula

Plagiacanthidae (lophophaenid)

Popofsky, 1908

Records: 47

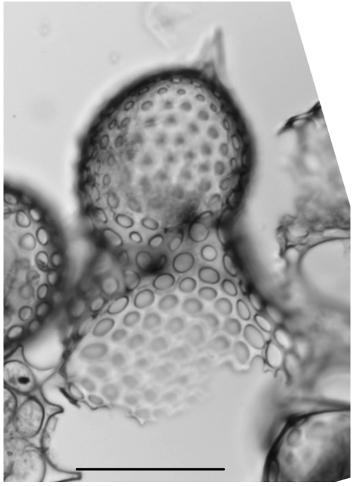

Lithomelissa celsagula

Plagiacanthidae (lophophaenid)

Renaudie & Lazarus, 2015

Records: 22

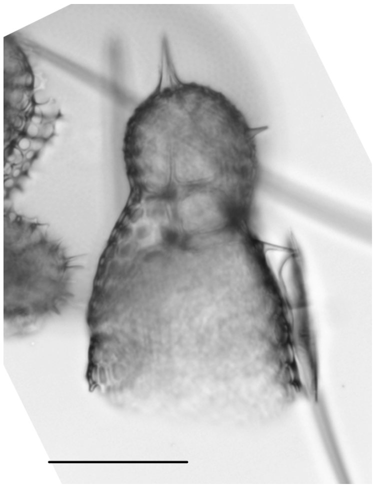

Lithomelissa cheni
Plagiacanthidae (lophophaenid)
Caulet, 1991
Records: 36

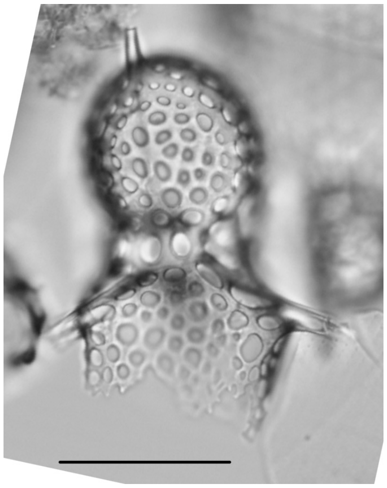

Lithomelissa ehrenbergii
Plagiacanthidae (lophophaenid)
Butschli, 1882
Records: 97

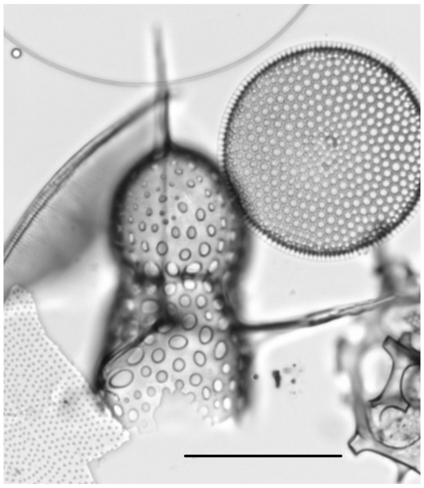

Lithomelissa ehrenbergii cf
Plagiacanthidae (lophophaenid)
Undescribed
Records: 57

Very similar to L. ehrenbergii, except that cephalis is very thin and smooth, rather than thick and bumpy. Cephalis is often so thin that apical spine is clearly visible within.

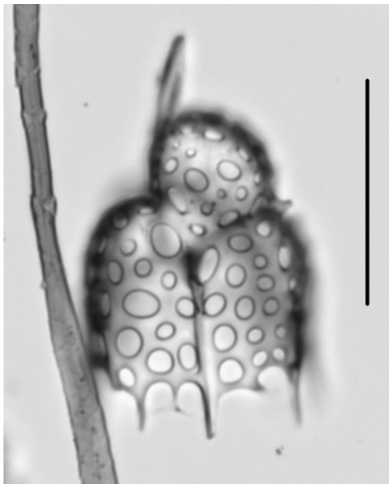

Lithomelissa kozoi
Plagiacanthidae (lophophaenid)
Renaudie & Lazarus, 2013
Records: 63

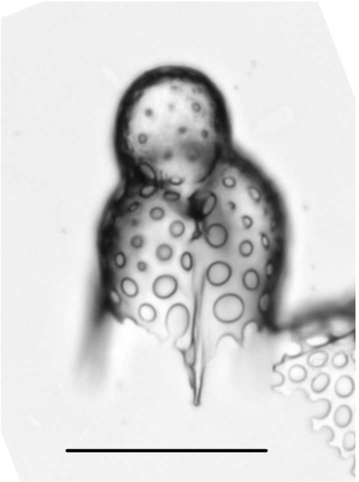

Lithomelissa kozoi cf sp 1
Plagiacanthidae (lophophaenid)
Undescribed
Records: 221

This species is different from L. kozoi in that it lacks horizontally-oriented thoracic spines, and tends to have muted cephalic spines. Cephalis can be hyaline.

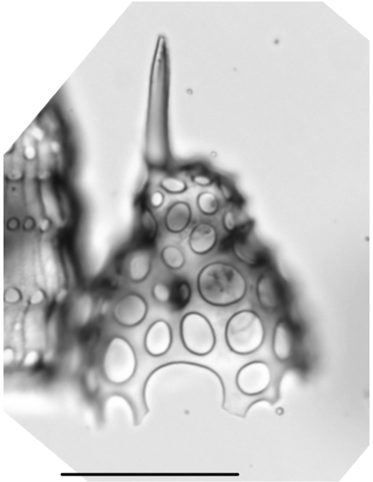

Lithomelissa kozoi cf sp 2
Plagiacanthidae (lophophaenid)
Undescribed
Records: 22

This species is distinct for its small and tilted cephalis, and relatively large pores on thorax.

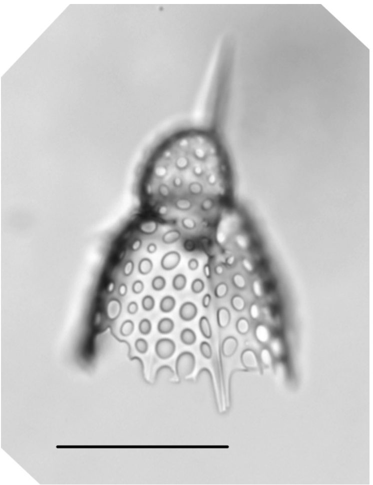

Lithomelissa kozoi cf sp 4

Plagiacanthidae (lophophaenid)

Undescribed

Records: 42

This species is distinct for its relatively long and narrow thorax compared to the other Lithomelissa species.

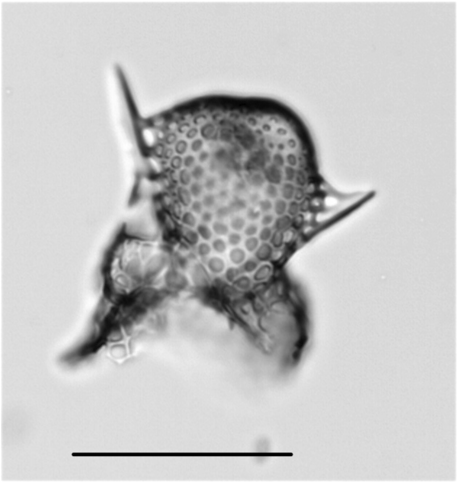

Lithomelissa laticeps

Plagiacanthidae (lophophaenid)

Jørgensen, 1905

Records: 20

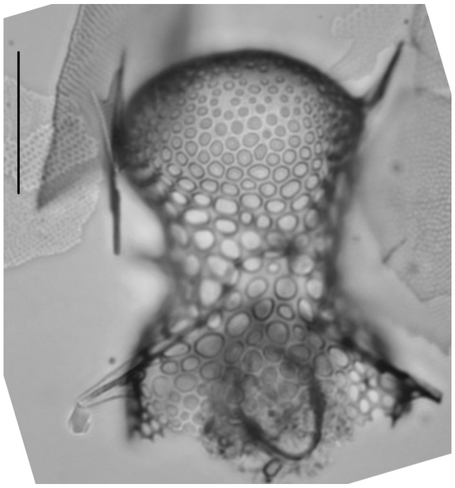

Lithomelissa laticeps cf

Plagiacanthidae (lophophaenid)

Undescribed

Records: 29

Cephalis shape and pore structure is similar to L. laticeps, but this species is different because it only has one large spine protruding from cephalis, rather than two. In addition, the spine runs directly along the side of the cephalis, and does not extend outwards with a meshwork of pores, like L. laticeps.

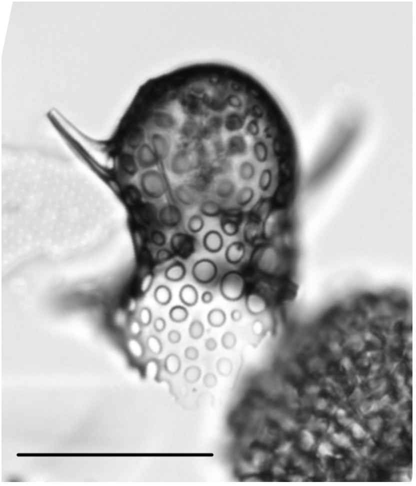

Lithomelissa laticeps cf sp 2

Plagiacanthidae (lophophaenid)

Undescribed

Records: 13

Somewhat similar to L. laticeps, with two strong apical spines. However, this species does not have any meshwork connecting those spines to the cephalis, like L. laticeps does.

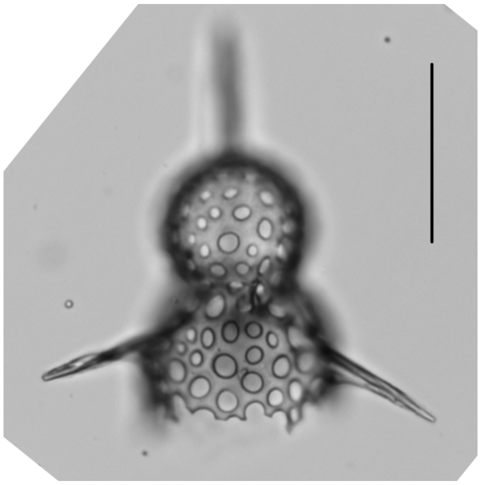

Lithomelissa mitra

Plagiacanthidae (lophophaenid)

Butschli, 1882

Records: 83

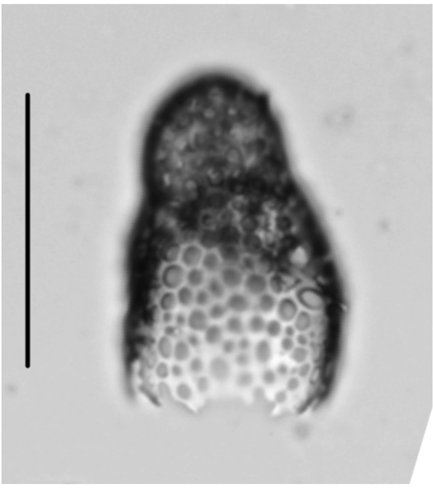

Lithomelissa setosa

Plagiacanthidae (lophophaenid)

Jørgensen, 1905

Records: 65

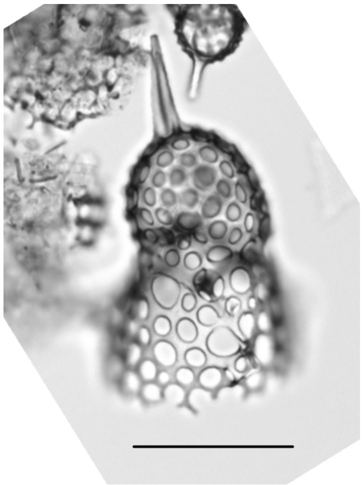

Lithomelissa sp 1

Plagiacanthidae (lophophaenid)

Undescribed

Records: 28

Compressed, asymmetrical cephalis, longer than it is wide. Differs from L. ehrenbergii in that it does not have an extended neck, and differs from L. mitra in that cephalis is not round.

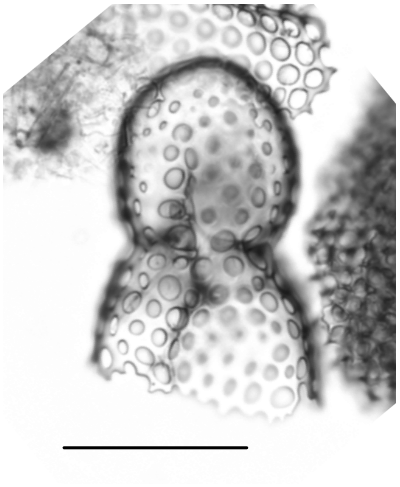

Lithomelissa sp 2

Plagiacanthidae (lophophaenid)

Undescribed

Records: 16

Thinly-silicified, with a muted spine that runs along the cephalis, forming an indented suture lineation. Differs from L. thoracites in that it does not have strong thoracic spines, or an apical spine that breaches cephalis.

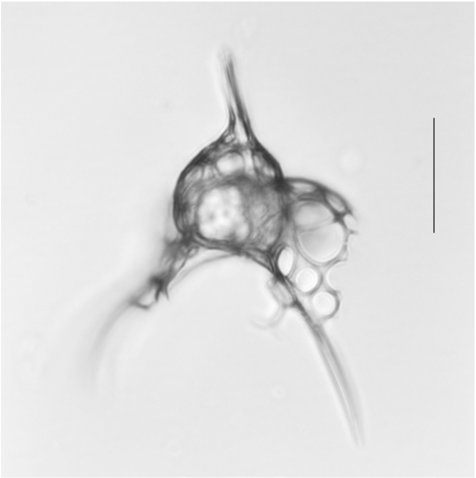

Lithomelissa spinosissima

Plagiacanthidae (lophophaenid)

Tan & Tchang, 1976

Records: 21

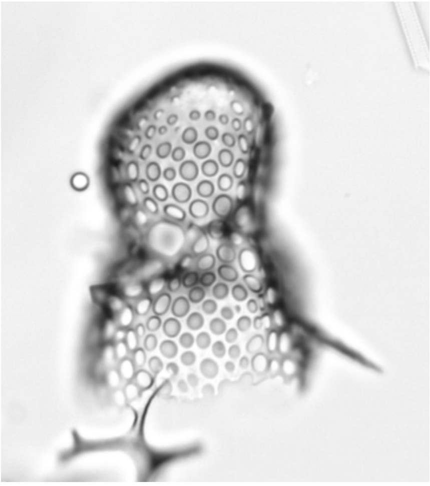

Lithomelissa thoracites

Plagiacanthidae (lophophaenid)

Haeckel, 1860

Records: 117

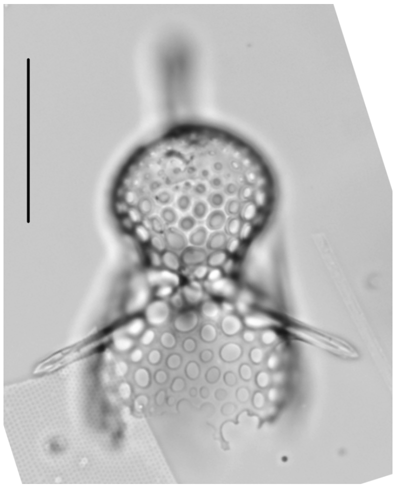

Lithomelissa thoracites cf

Plagiacanthidae (lophophaenid)

Undescribed

Records: 123

Similar to L. thoracites, except that thoracic spines are symmetrical, in contrast to the spines protruding at different angles in L. thoracites. Differs from L. mitra in that apical spine runs along the side of cephalis, rather than through its center.

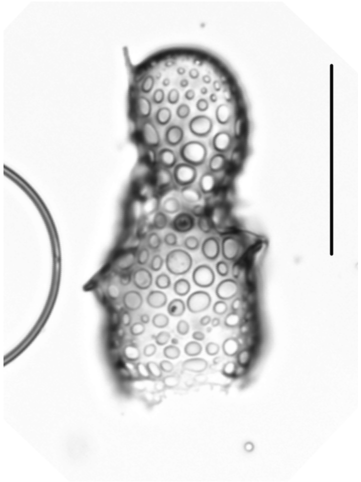

Lophophaena buetschlii

Plagiacanthidae (lophophaenid)

(Haeckel) Petrushevskaya, 1971

Records: 132

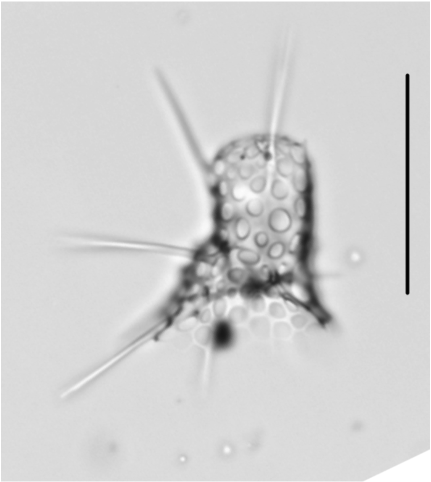

Lophophaena buetschlii cf

Plagiacanthidae (lophophaenid)

Undescribed

Records: 33

Like L. buetschlii, this species has an elongated, sometimes open, cephalis. But it differs in that it has a more broad thorax with a different array of thoracic spines.

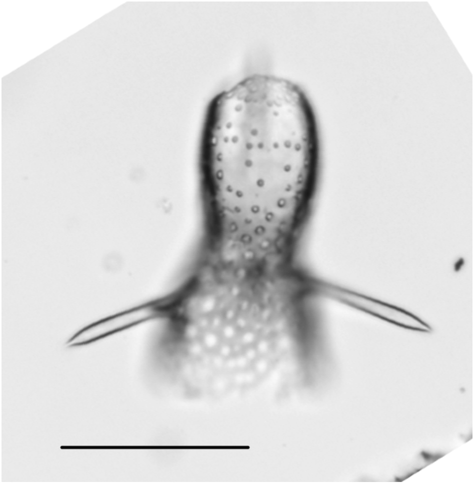

Lophophaena buetschlii cf sp 2

Plagiacanthidae (lophophaenid)

Undescribed

Records: 179

Similar to L. buetschlii, except that this species has long, conical thoracic spines, usually with pointed tips.

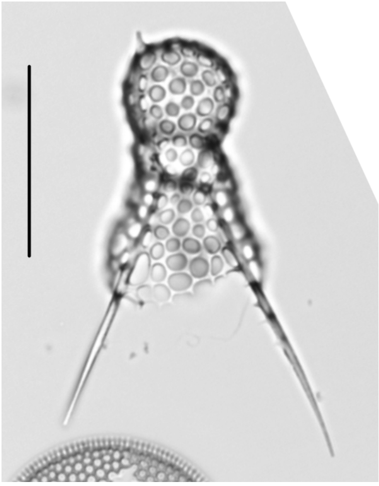

Lophophaena clevei

Plagiacanthidae (lophophaenid)

Petrushevskaya, 1971

Records: 31

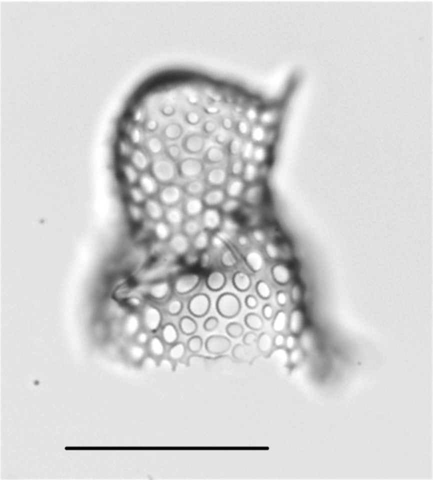

Lophophaena decacantha

Plagiacanthidae (lophophaenid)

Takahashi, 1991

Records: 77

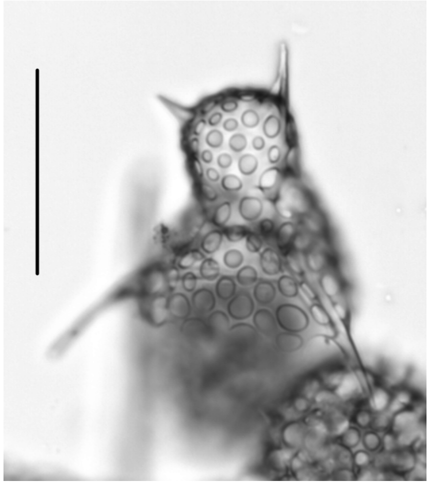

Lophophaena decacantha cf

Plagiacanthidae (lophophaenid)

Undescribed

Records: 4

Cephalis is similar to L. decacantha, but thorax has strong spines that protrude and resemble feet.

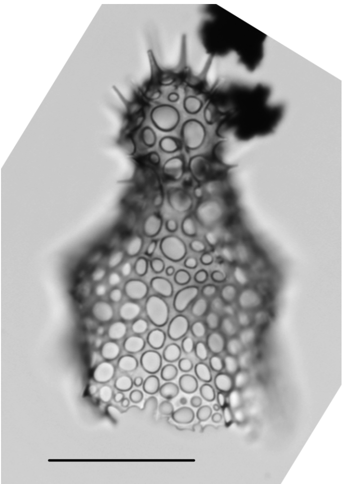

Lophophaena hispida

Plagiacanthidae (lophophaenid)

(Ehrenberg) Petrushevskaya, 1971

Records: 141

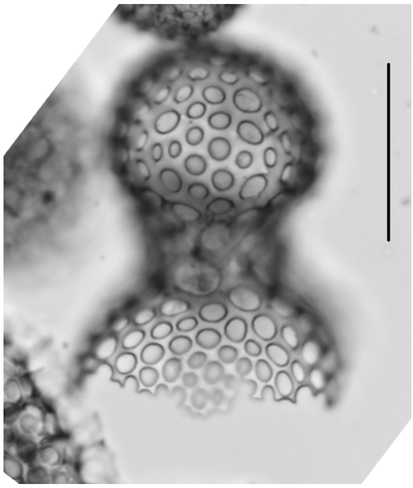

Lophophaena macrencephala
Plagiacanthidae (lophophaenid)
Clark & Campbell, 1945
Records: 47

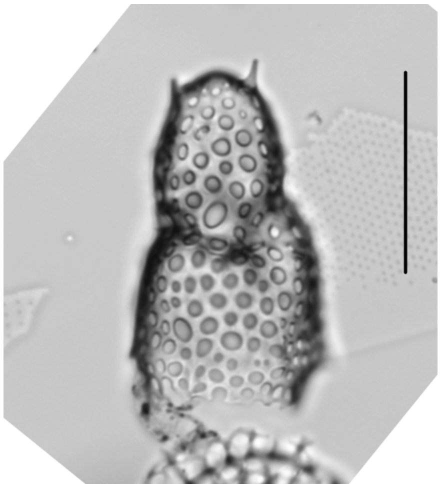

Lophophaena nadezdae
Plagiacanthidae (lophophaenid)
Petrushevskaya, 1971
Records: 178

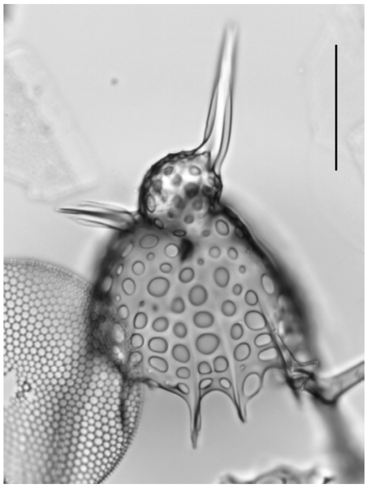

Lophophaena tekopua
Plagiacanthidae (lophophaenid)
O'Connor, 1997
Records: 30

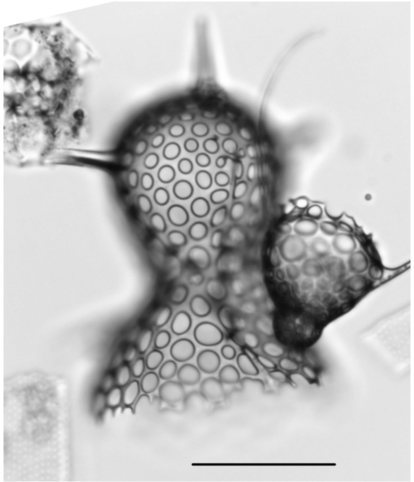

Lophophaena witjazii
Plagiacanthidae (lophophaenid)
(Petrushevskaya) Van de Paverd, 1995
Records: 103

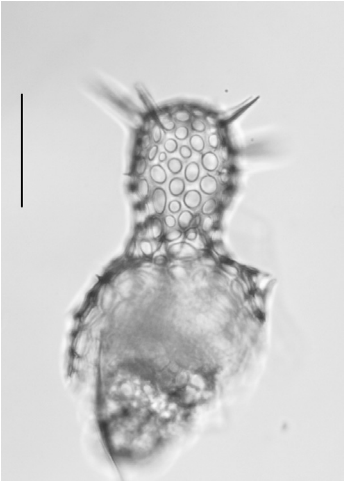

Lophophaena witjazii cf sp 1
Plagiacanthidae (lophophaenid)
Undescribed
Records: 10

This species differs from L. witjazii in that its cephalis is highly elongated.

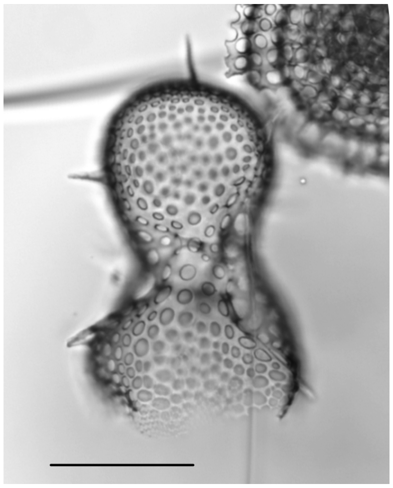

Lophophaena witjazii cf sp 2
Plagiacanthidae (lophophaenid)
Undescribed
Records: 60

Similar to L. witjazii, except that cephalis is more elongated, with distinct neck, less robust spines, and better-defined thorax shape.

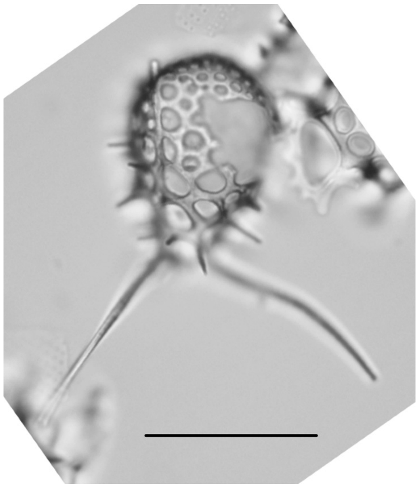

Peridium longispinum
Plagiacanthidae (lophophaenid)
Jørgensen, 1900
Records: 48

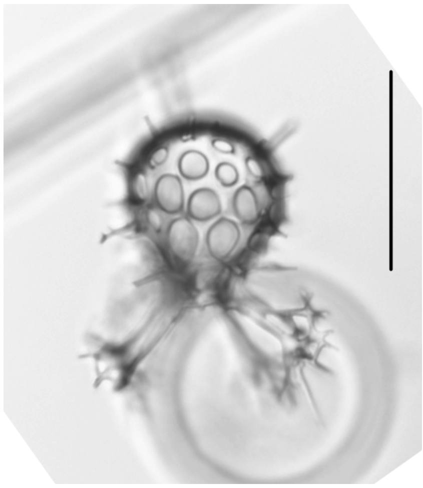

Peridium spinipes
Plagiacanthidae (lophophaenid)
Haeckel, 1887
Records: 81

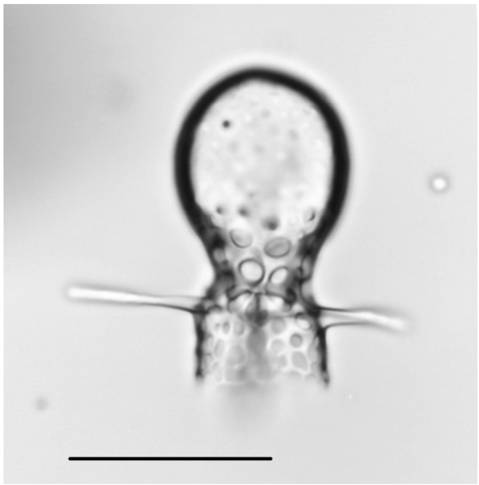

Peromelissa phalacra
Plagiacanthidae (lophophaenid)
Haeckel, 1887
Records: 57

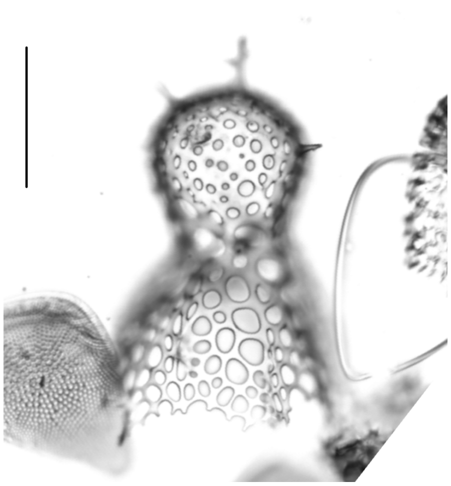

unknown plagonid group C sp 1
Plagiacanthidae (lophophaenid)
Undescribed
Records: 436

This species often has forked cephalic spines, and spines that produce ridges on both cephalis and thorax. These prominent ridges on thorax and relatively large cephalis distinguish this species from unknown plagonid group C sp 12 cf.

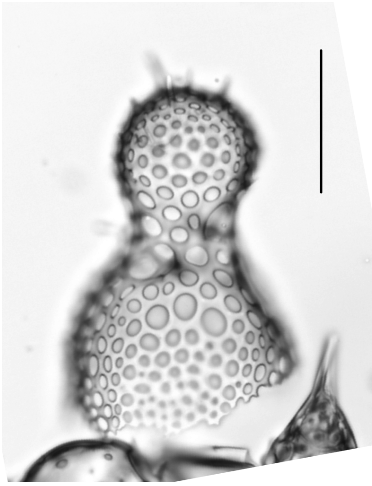

unknown plagonid group C sp 1 cf
Plagiacanthidae (lophophaenid)
Undescribed
Records: 29

This species is like unknown plagonid group E sp 1, except that the thorax lacks prominent ribs.

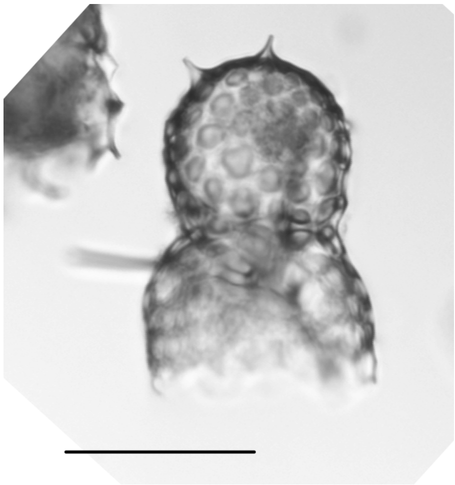

unknown plagonid group C sp 12
Plagiacanthidae (lophophaenid)
Undescribed
Records: 55

Cephalis covered in short, sharp, conical spines, and is rounded.

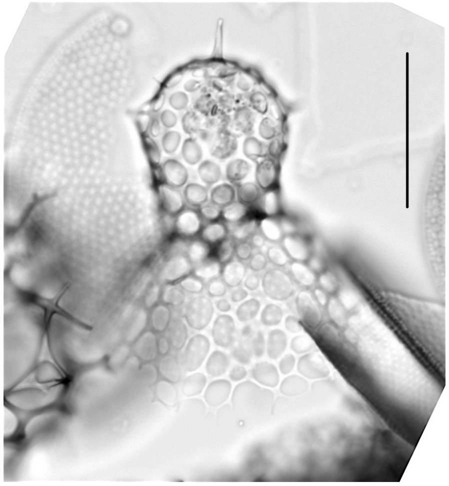

unknown plagonid group C sp 12 cf

Plagiacanthidae (lophophaenid)

Undescribed

Records: 142

Like unknown plagonid group C sp 12, this species has many short cephalic spines, but differs int hat the thorax is much more broad and has many spiny lineations. It also typically has a cephalis that is slightly flat on top.

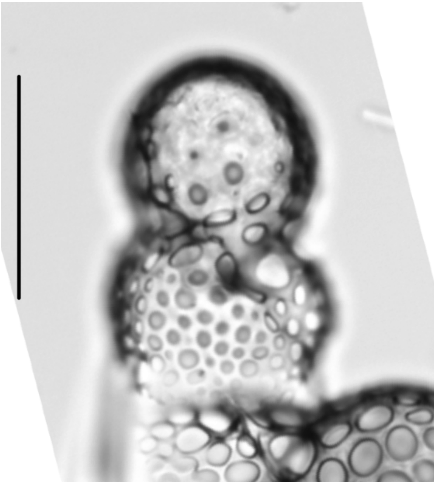

unknown plagonid group C sp 13

Plagiacanthidae (lophophaenid)

Undescribed

Records: 36

This species has a heavily-silicified spherical cephalis, and a narrow, rounded thorax.

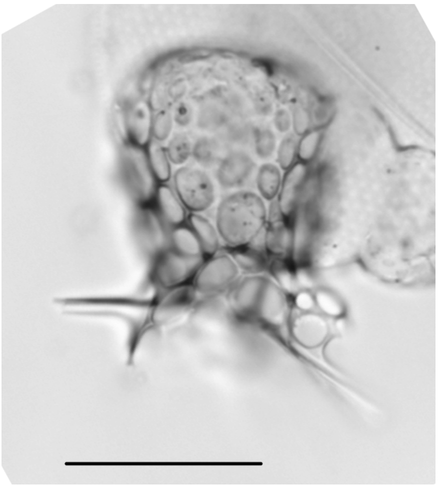

unknown plagonid group C sp 14

Plagiacanthidae (lophophaenid)

Undescribed

Records: 24

This species has a triangular-shaped cephalis, with pores becoming smaller toward the top.

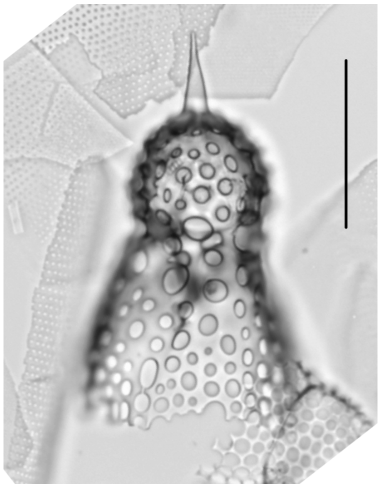

unknown plagonid group C sp 16

Plagiacanthidae (lophophaenid)

Undescribed

Records: 148

This species has a thickly-silicified cephalis with a strong conical apical spine. Thorax is long and narrow. The area between cephalis and thorax tends to have large, irregular pores and hyaline spaces.

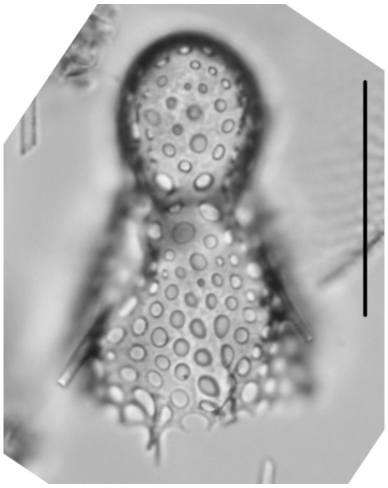

unknown plagonid group C sp 17

Plagiacanthidae (lophophaenid)

Undescribed

Records: 203

This species is distinct for its thorax; an array of spines run down it and typically separate from the outer edge.

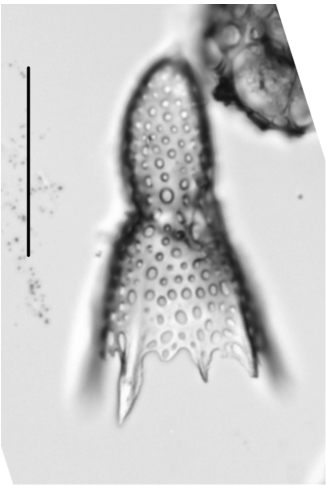

unknown plagonid group C sp 17 cf

Plagiacanthidae (lophophaenid)

Undescribed

Records: 6

This species has similar strong thoracic spines to unknown plagonid group C sp 17, but differs in that the cephalis is more narrow, resembling that of L. buetschlii.

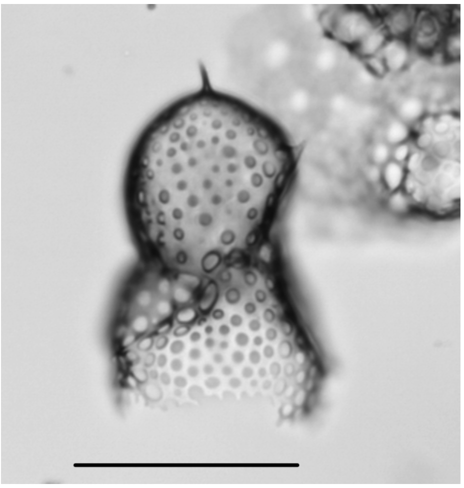

unknown plagonid group C sp 20

Plagiacanthidae (lophophaenid)

Undescribed

Records: 201

This species has two short cephalic spines, strong indentations on thorax where spines have formed, and smaller and/or fewer pores on cephalis than on thorax.

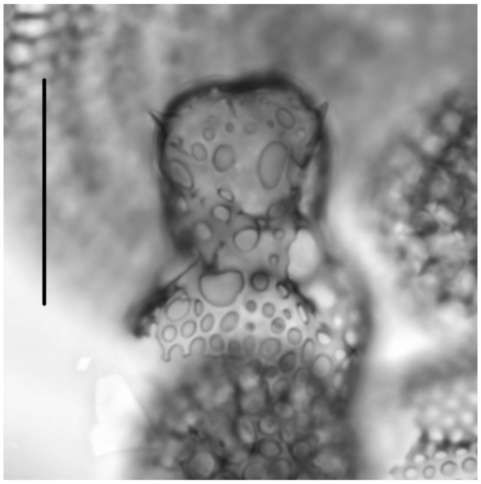

unknown plagonid group C sp 21

Plagiacanthidae (lophophaenid)

Undescribed

Records: 13

This species is distinct for its very irregular pore shapes and sizes on the cephalis, contrasted by small, regular pores on the thorax.

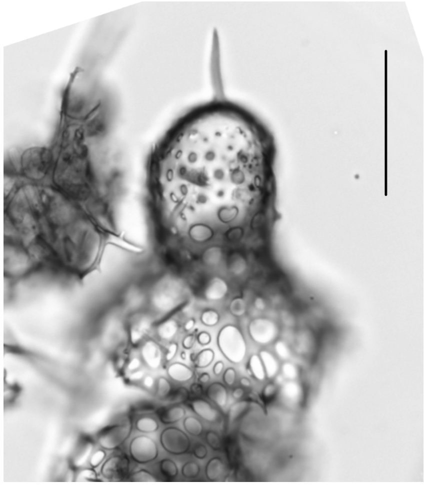

unknown plagonid group C sp 22

Plagiacanthidae (lophophaenid)

Undescribed

Records: 9

This species has a narrow, thumb-shaped cephalis, with very small pores and one conical spine. Thorax is broad, with wide-set spines and very irregular pores, some of them relatively large.

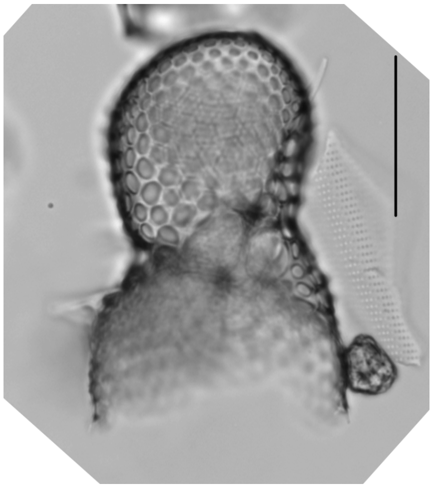

unknown plagonid group C sp 23

Plagiacanthidae (lophophaenid)

Undescribed

Records: 69

This species has a large cephalis with fine, regular pores. There is a strong apical spine, with two distinct “pouches” at its base. There is also a short, strong spine protruding from the base of neck, on the opposite side to the apical spine.

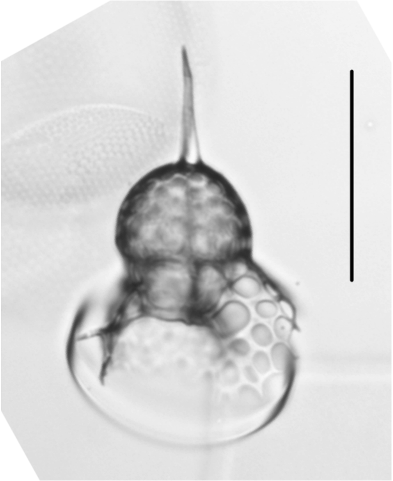

unknown plagonid group C sp 24

Plagiacanthidae (lophophaenid)

Undescribed

Records: 33

This species has a small, narrow cephalis, with a central apical spine that pierces the cephalis. Thorax is wider than cephalis. Two thoracic spines are short and relatively thin.

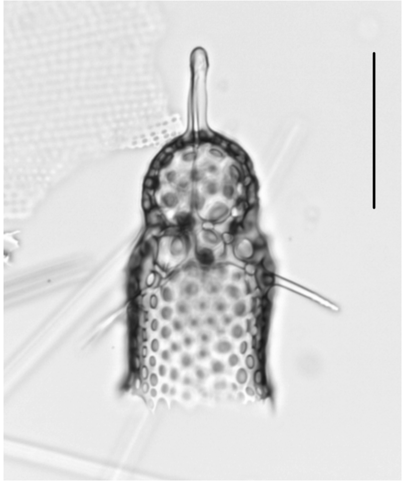

unknown plagonid group C sp 26

Plagiacanthidae (lophophaenid)

Undescribed

Records: 811

This species has a lot of variability, but differs from L. mitra and L. ehrenbergii with a smaller cephalis and no defined neck region. Other characteristics between these species are similar. This species has two thoracic spines, and an apical spine that pierces cephalis through its center. This apical spine often has a rounded tip. Cephalis varies from being as porous as the thorax to almost totally hyaline.

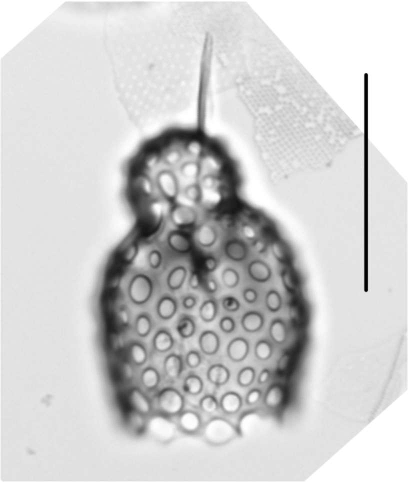

unknown plagonid group C sp 26 cf

Plagiacanthidae (lophophaenid)

Undescribed

Records: 73

This species is similar to unknown plagonid group C sp 26 cf, except that the cephalis is significantly more narrow than the thorax. It also often lacks thoracic spines.

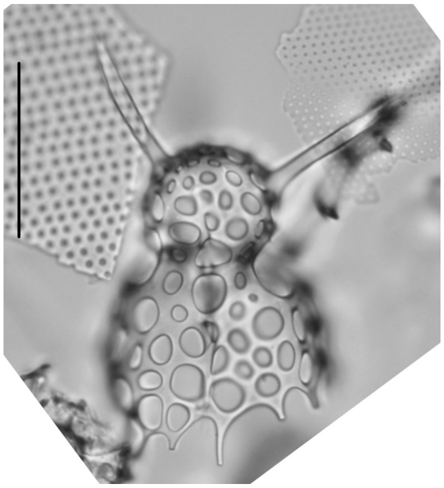

unknown plagonid group C sp 27

Plagiacanthidae (lophophaenid)

Undescribed

Records: 262

This species has at least two long, conical spines on the cephalis, often more than three. It is distinct for its spherical cephalis and enlarged pores near the base of the cephalis.

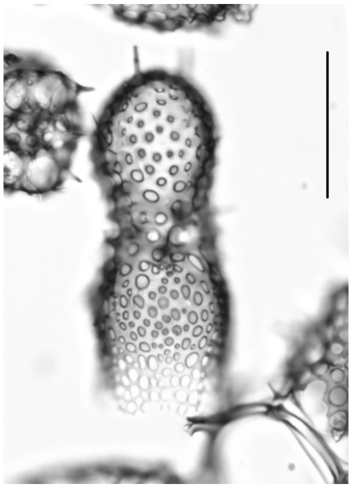

unknown plagonid group C sp 28

Plagiacanthidae (lophophaenid)

Undescribed

Records: 395

This species has an elongated cephalis, neck, and thorax. Cephalis is wider at the top than at the base, and has many thin spines around the top half.

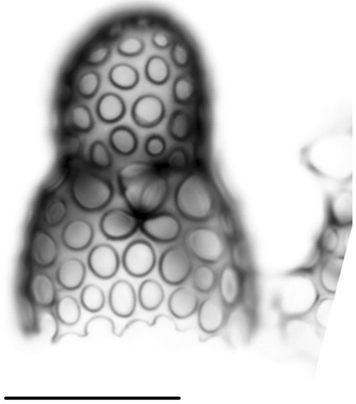

unknown plagonid group C sp 29

Plagiacanthidae (lophophaenid)

Undescribed

Records: 125

This species is notable for its smooth, thumb-shaped cephalis, with only a single muted spine occasionally present. Thorax is wider than cephalis. No thoracic spines are present.

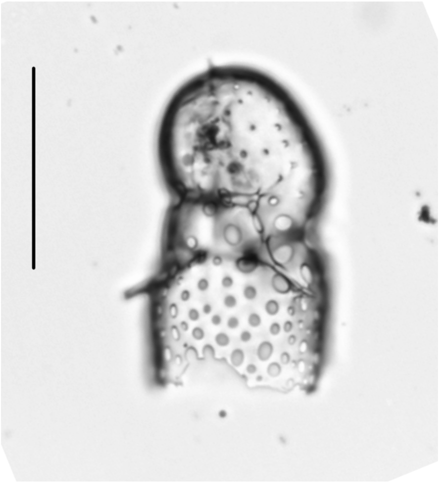

unknown plagonid group C sp 3

Plagiacanthidae (lophophaenid)

Undescribed

Records: 393

This species is identifiable for its long, lumpy, irregular-shaped cephalis, that typically has smaller pores than the thorax. It has an elongated neck area as well. Apical spine cuts through the center of the cephalis.

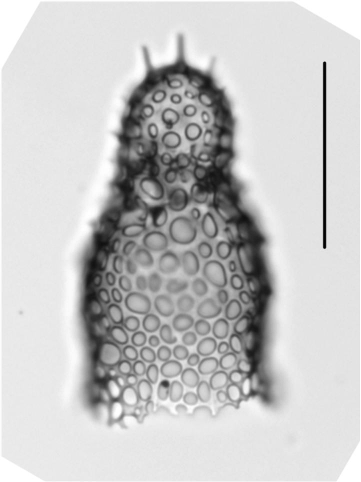

unknown plagonid group C sp 30

Plagiacanthidae (lophophaenid)

Undescribed

Records: 277

This species is similar to L. hispida in that the cephalis is covered in small needle-like spines, but differs in that the cephalis has small pores, matching those of the thorax.

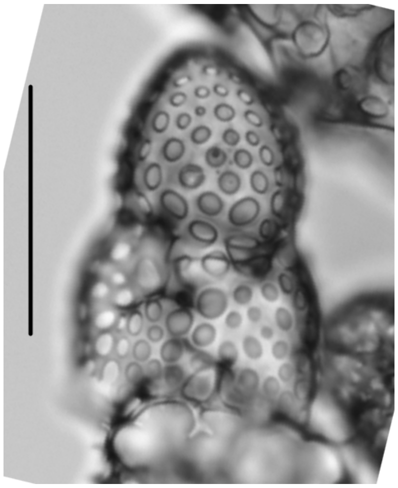

unknown plagonid group C sp 30 cf

Plagiacanthidae (lophophaenid)

Undescribed

Records: 57

This species has a long cephalis with a pointed tip, differentiating it from unknown plagonid group C sp 29 and unknown plagonid group C sp 28. There are no visible spines.

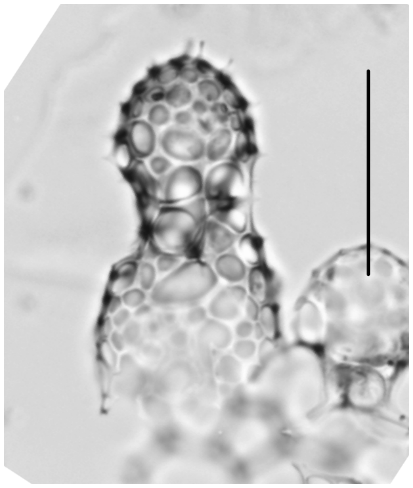

unknown plagonid group C sp 31

Plagiacanthidae (lophophaenid)

Undescribed

Records: 67

This species resembles unknown plagonid group C sp 30, except that it has distinct large pores that form an extra layer of silica around the neck area.

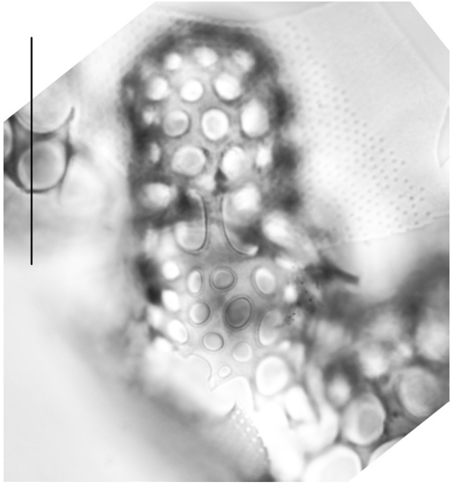

unknown plagonid group C sp 31 cf

Plagiacanthidae (lophophaenid)

Undescribed

Records: 8

This species is similar to unknown plagonid group C sp 31, except that it has a slightly wider neck, and has the additional character of two short thoracic spines.

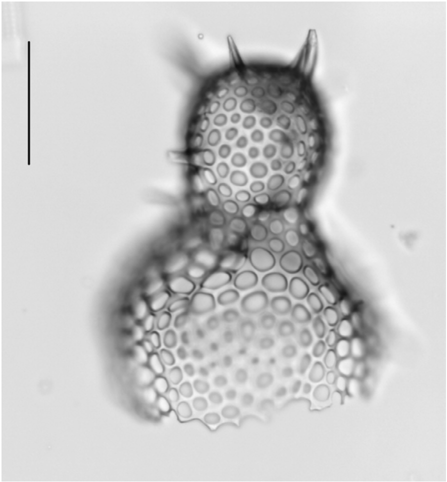

unknown plagonid group C sp 32

Plagiacanthidae (lophophaenid)

Undescribed

Records: 100

This species is similar to L. witjazii with its crown of cephalic spines, but differs in that it has a more asymmetrical cephalis shape, and has a single spine that is most prominent.

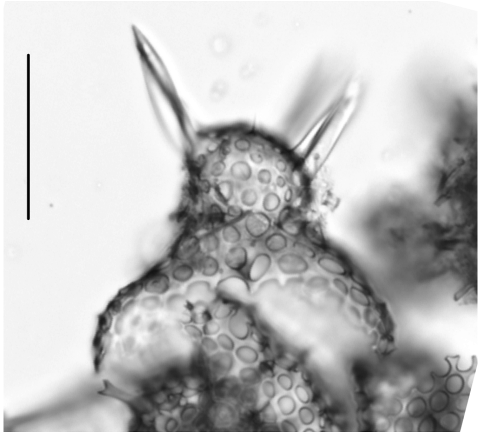

unknown plagonid group C sp 32 cf

Plagiacanthidae (lophophaenid)

Undescribed

Records: 62

This species has strong cephalic spines like unknown plagonid group C sp 32, but differs in that it has two equally prominent spines and has a significantly smaller cephalis.

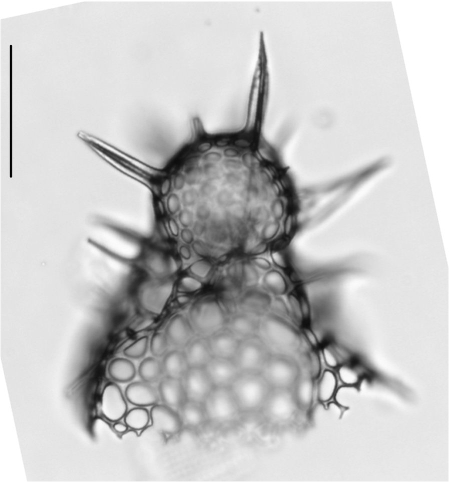

unknown plagonid group C sp 32 cf sp 2

Plagiacanthidae (lophophaenid)

Undescribed

Records: 56

This species is very similar to unknown plagonid group C sp 32, except that the neck area is not constricted, and blends into the thorax. Cephalis is also more irregular.

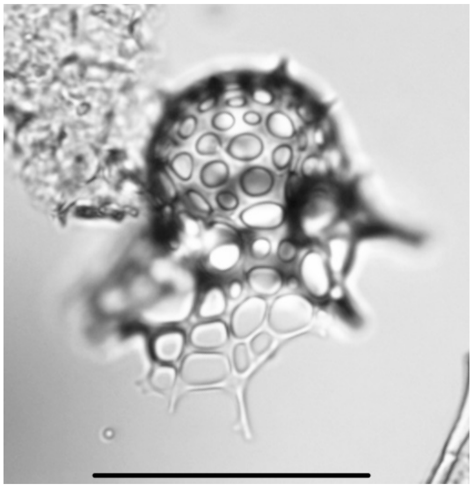

unknown plagonid group C sp 35

Plagiacanthidae (lophophaenid)

Undescribed

Records: 8

This species has a round, thorny cephalis, and a thorax with rectangular pores.

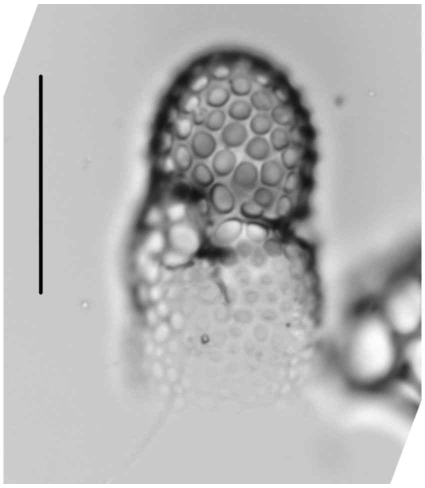

unknown plagonid group C sp 36

Plagiacanthidae (lophophaenid)

Undescribed

Records: 163

This species is distinctive for its lightly-silicified thorax, through which branching spines are visible. Its cephalis is typically more heavily silicified than the thorax. Small thoracic spines are sometimes preserved.

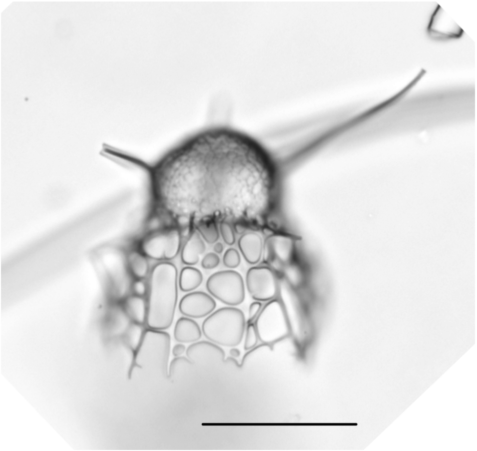

unknown plagonid group C sp 38

Plagiacanthidae (lophophaenid)

Undescribed

Records: 140

This species has very large, angular pores on the thorax, that contrast strongly with the nearly-hyaline cephalis. Cephalis is broad, round, and has many bladed spines. This species has a great deal of variability, and occasionally expresses feet protruding from the sides of the thorax.

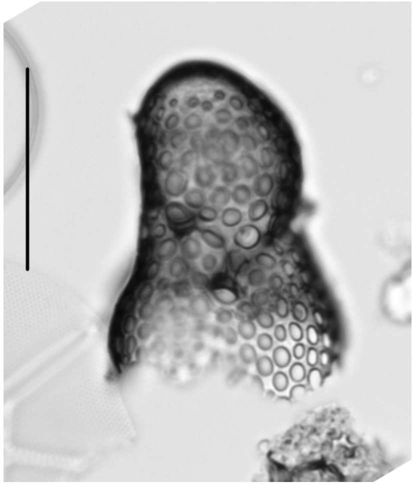

unknown plagonid group C sp 41

Plagiacanthidae (lophophaenid)

Undescribed

Records: 104

This species is distinctive for having one flat side to its cephalis, corresponding to the apical spine.

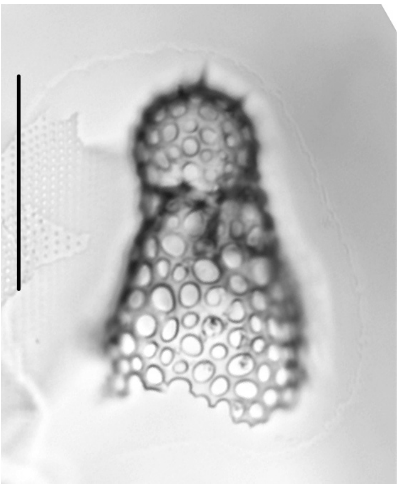

unknown plagonid group C sp 42

Plagiacanthidae (lophophaenid)

Undescribed

Records: 27

This species resembles unknown plagonid group C sp 30, except that cephalis is broader and more hemispherical in shape. Cephalis is approximately the same width as the top of the thorax.

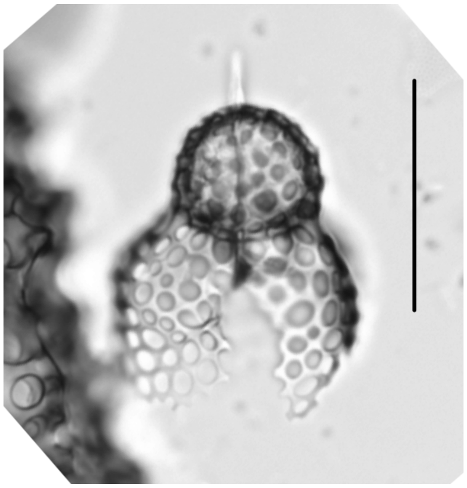

unknown plagonid group C sp 43

Plagiacanthidae (lophophaenid)

Undescribed

Records: 83

This species has a broad, round cephalis and an apical spine. Distinctive for its compressed neck area, with structures extending into thorax. Differs from unknown plagonid group C sp 24 in that its cephalis is more broad. Differs from unknown plagonid group C sp 26 in that thorax is significantly wider than cephalis.

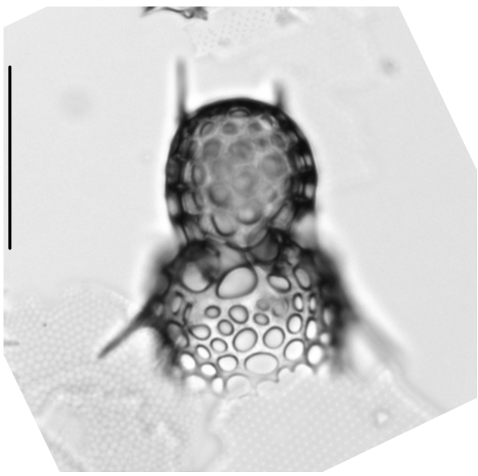

unknown plagonid group C sp 44

Plagiacanthidae (lophophaenid)

Undescribed

Records: 49

This species has a thumb-shaped cephalis, with two strong spines. Pores around the base of cephalis are larger than any of its other pores.

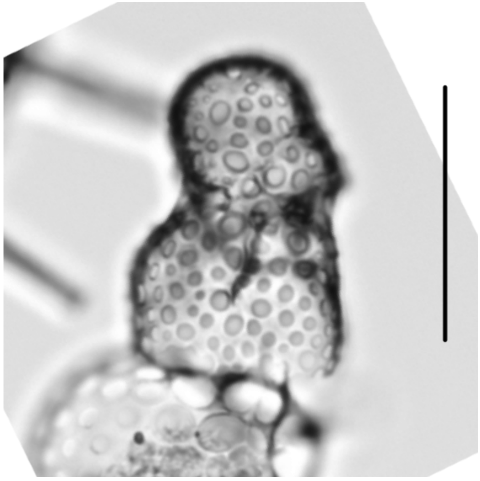

unknown plagonid group C sp 46

Plagiacanthidae (lophophaenid)

Undescribed

Records: 39

This species has a unique, small lump near the outside middle part of the cephalis.

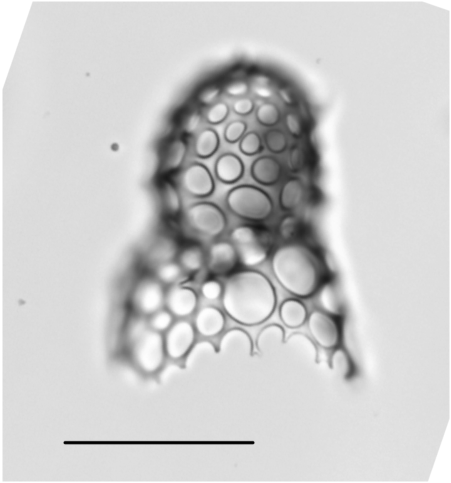

unknown plagonid group C sp 47

Plagiacanthidae (lophophaenid)

Undescribed

Records: 62

Cephalis tends to have one flat side, but pores are larger than those of the similar species, unknown plagonid group C sp 41. Largest pores originate at the neck, and become smaller in either direction.

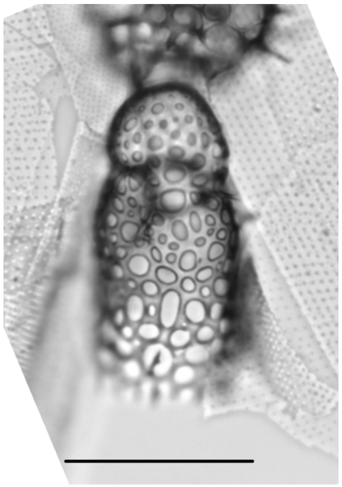

unknown plagonid group C sp 48

Plagiacanthidae (lophophaenid)

Undescribed

Records: 15

This species has a short, wide, irregularly-shaped cephalis, and an unusually-long thorax with teeth at the termination.

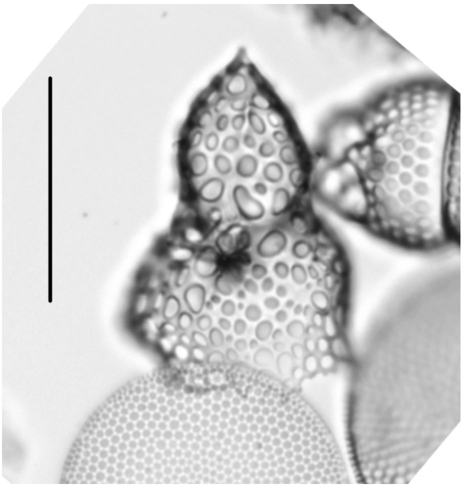

unknown plagonid group C sp 5

Plagiacanthidae (lophophaenid)

Undescribed

Records: 30

This species has a pointed cephalis with a spine protruding from the top. Other, smaller spines are occasionally preserved on sides of cephalis.

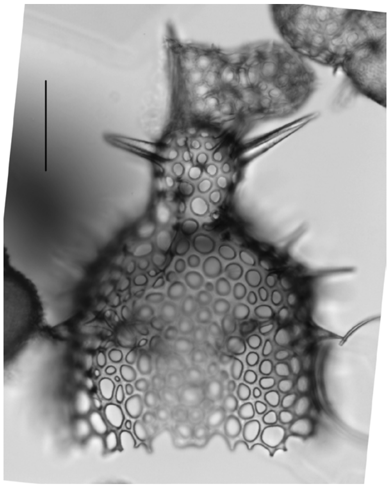

unknown plagonid group C sp 50

Plagiacanthidae (lophophaenid)

Undescribed

Records: 20

This species is large, and have many strong cephalic spines, differentiating it from unknown plagonid group C sp 32 cf. The thorax is also very thorny and broad.

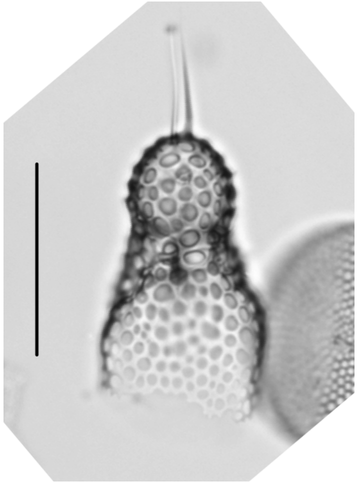

unknown plagonid group C sp 51

Plagiacanthidae (lophophaenid)

Undescribed

Records: 66

This species has a thinly-silicified thorax, and somewhat resembles unknown plagonid group C sp 36. It is differentiated by the conical apical spine on top of the cephalis, and tendency to have a longer neck area.

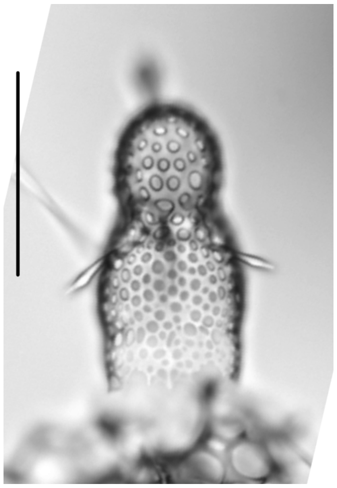

unknown plagonid group C sp 51 cf

Plagiacanthidae (lophophaenid)

Undescribed

Records: 90

This species is similar to unknown plagonid group C sp 51, but it differs with two downward-pointing thoracic spines

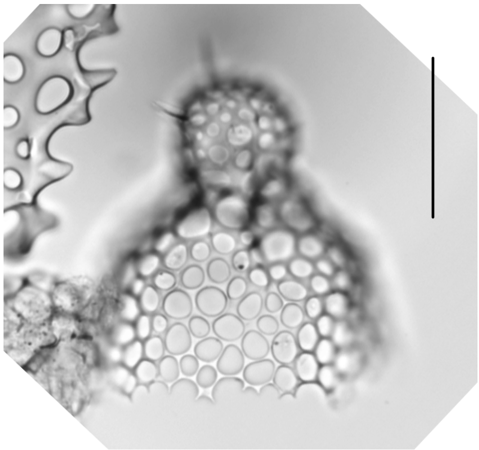

unknown plagonid group C sp 52

Plagiacanthidae (lophophaenid)

Undescribed

Records: 13

This species is similar to unknown plagonid group C sp 50, except that it is smaller, and has fewer, weaker spines on both cephalis and thorax.

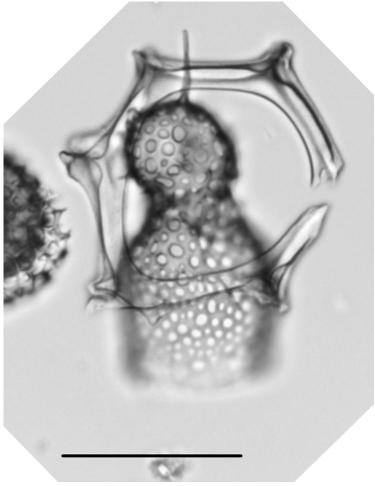

unknown plagonid group C sp 53

Plagiacanthidae (lophophaenid)

Undescribed

Records: 25

This species has a constricted neck area, and many thin spines on cephalis.

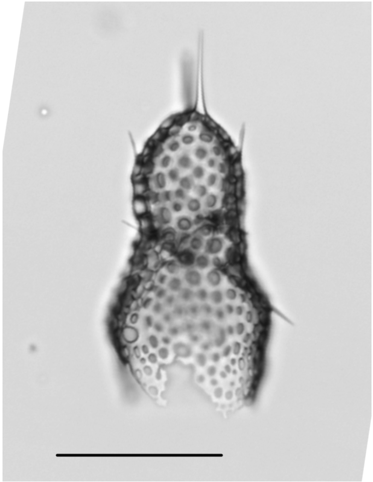

unknown plagonid group C sp 53 cf

Plagiacanthidae (lophophaenid)

Undescribed

Records: 139

This species resembles unknown plagonid group C sp 51 cf, except that it has many cephalic spines, two of which are consistently longer than the others.

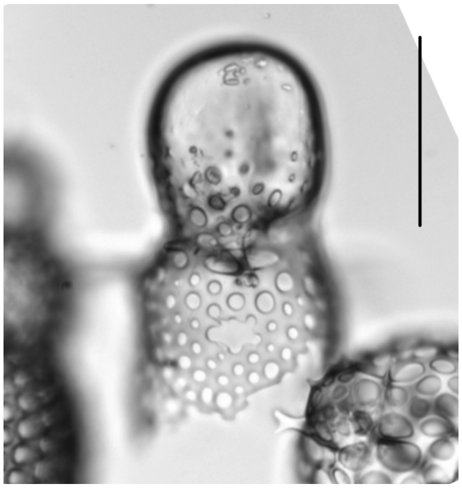

unknown plagonid group C sp 54

Plagiacanthidae (lophophaenid)

Undescribed

Records: 292

This species is similar to Lithomelissa sp 2, except that the top of the cephalis tends to be more flat, and is usually hyaline, or has very small pores in that area.

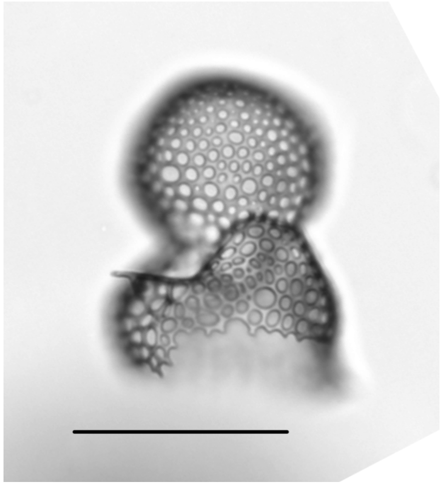

unknown plagonid group C sp 55

Plagiacanthidae (lophophaenid)

Undescribed

Records: 104

This species has a large, bulbous cephalis, and a distinctive thorax with two small spines a flattened area between them. Thorax also sometimes has a large lump feature associated with the two spines. No visible spines on cephalis.

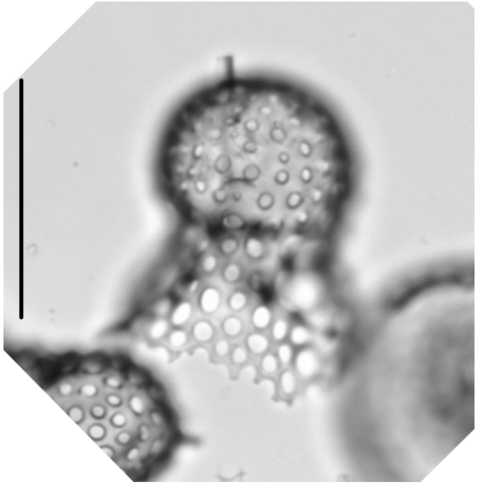

unknown plagonid group C sp 57

Plagiacanthidae (lophophaenid)

Undescribed

Records: 10

This species resembles unknown plagonid group C sp 3, except that the cephalis is more compressed. However, it has a very similar neck and apical spine construction. This species differs from unknown plagonid group C sp 26 in that its cephalis is relatively wider compared to the thorax.

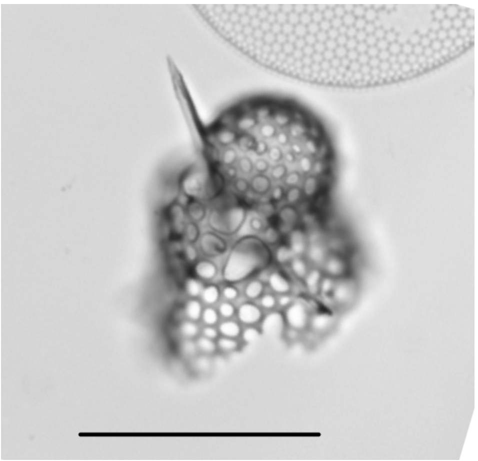

unknown plagonid group C sp 58

Plagiacanthidae (lophophaenid)

Undescribed

Records: 66

This species has a distinctive lobe near the base of the cephalis, that connects the strong apical spine to the top of the thorax, and is associated with two thoracic spines.

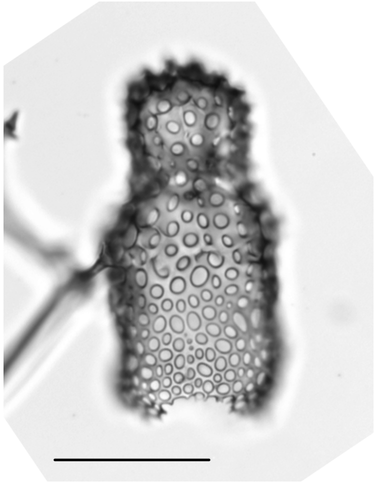

unknown plagonid group C sp 59

Plagiacanthidae (lophophaenid)

Undescribed

Records: 38

This species has a heavily-silicified, rough-textured cephalis, and a rectangular thorax.

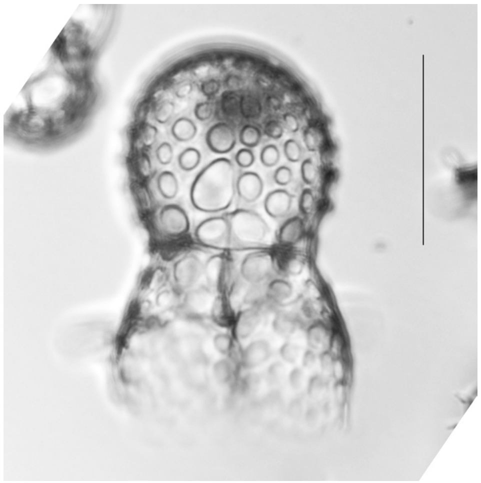

unknown plagonid group C sp 6

Plagiacanthidae (lophophaenid)

Undescribed

Records: 118

This species has a large collar structure, which is sunken into the thorax. Otherwise, it resembles L. thoracites cf, with its attached apical spine and round cephalis.

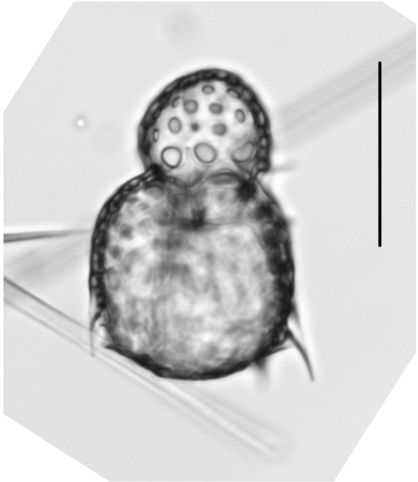

unknown plagonid group C sp 60

Plagiacanthidae (lophophaenid)

Undescribed

Records: 82

This species has a small, rounded thorax that is enclosed on the bottom. Muted feet protrude from the thorax, just above its base.

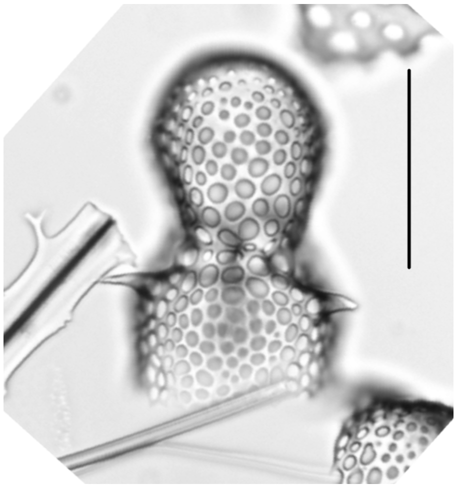

unknown plagonid group C sp 61

Plagiacanthidae (lophophaenid)

Undescribed

Records: 57

This species has a long cephalis, that is widest near the top and narrowest at the bottom. It has two short and thick conical thoracic spines

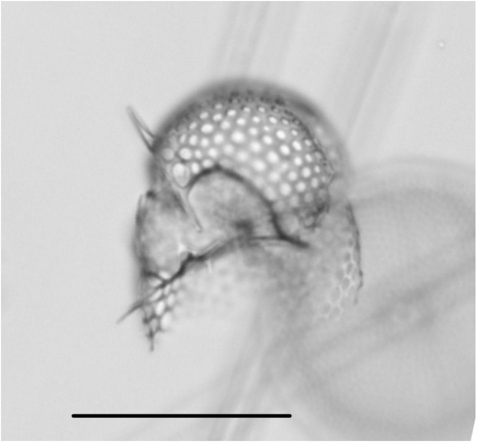

unknown plagonid group C sp 62

Plagiacanthidae (lophophaenid)

Undescribed

Records: 6

This species has a uniquely-shaped cephalis, that is wider than it is long. It also has two very large pouches at the base of the apical spine, and another strong cephalic spine opposite the apical spine. Pores are fine and regular.

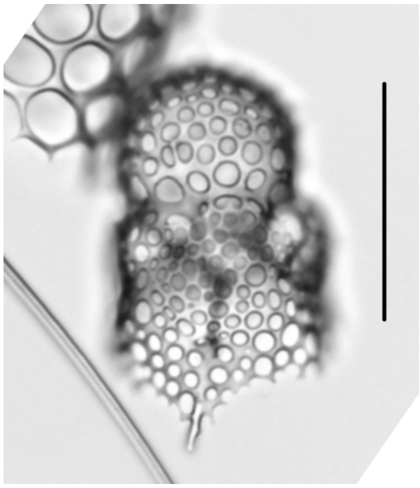

unknown plagonid group C sp 63

Plagiacanthidae (lophophaenid)

Undescribed

Records: 32

This species is unique for its thorax, which produces a ring of small lobes all around the base and lower part of the cephalis. Thorax is thinly-silicified compared to the cephalis.

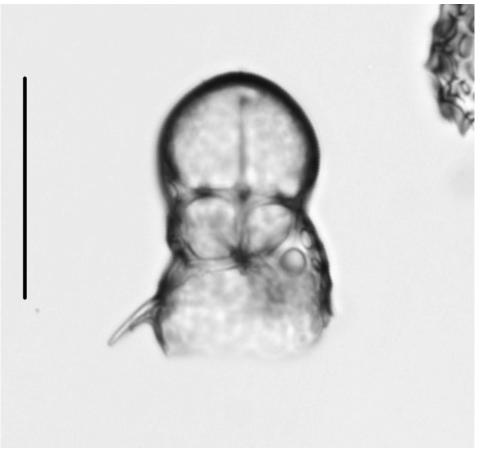

unknown plagonid group C sp 64

Plagiacanthidae (lophophaenid)

Undescribed

Records: 38

This species is small, with a distinct collar structure that rises up ~1/3 of the cephalis. Cephalis is usually almost hyaline, apical spine bisects cephalic interior, and thorax is only slightly wider than cephalis.

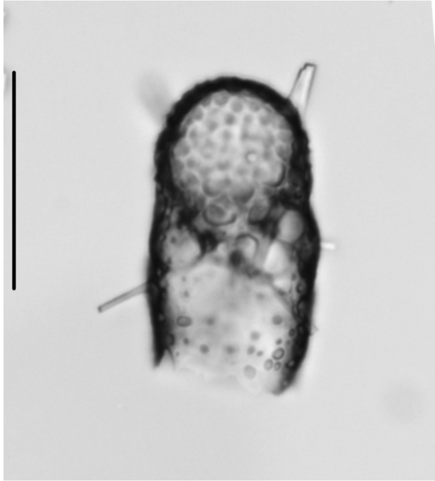

unknown plagonid group C sp 65

Plagiacanthidae (lophophaenid)

Undescribed

Records: 20

This species resembles unknown plagonid group C sp 63, except that it has a smaller cephalis, and relatively narrower thorax.

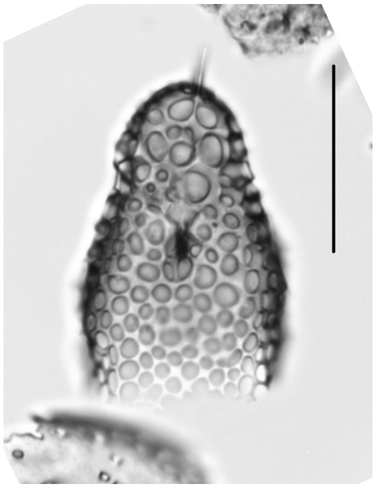

unknown plagonid group C sp 66

Plagiacanthidae (lophophaenid)

Undescribed

Records: 15

This species has a cephalis with large pores, compared to the thorax, and a smooth transition between cephalis and thorax. Muted apical spine.

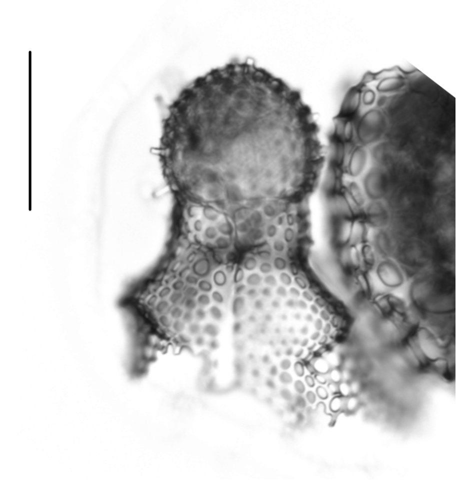

unknown plagonid group C sp 67

Plagiacanthidae (lophophaenid)

Undescribed

Records: 10

This species has strong shoulders on its thorax, and very thick conical thoracic spines. Cephalis is round, with many small spines.

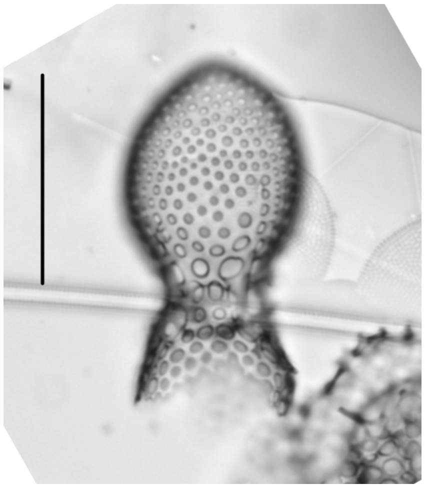

unknown plagonid group C sp 68

Plagiacanthidae (lophophaenid)

Undescribed

Records: 13

This species has a delicate, fine-pored cephalis, which is long , relatively narrow and very symmetrical. Thorax appears to be narrow, but no specimens have been observed with thorax fully preserved.

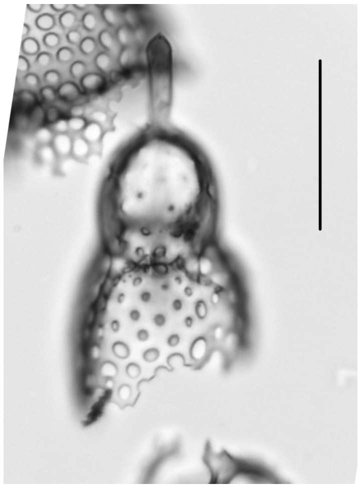

unknown plagonid group C sp 70

Plagiacanthidae (lophophaenid)

Undescribed

Records: 13

This species resembles unknown plagonid group C sp 26, except that the cephalis is significantly narrower, sometimes longer, and generally less round.

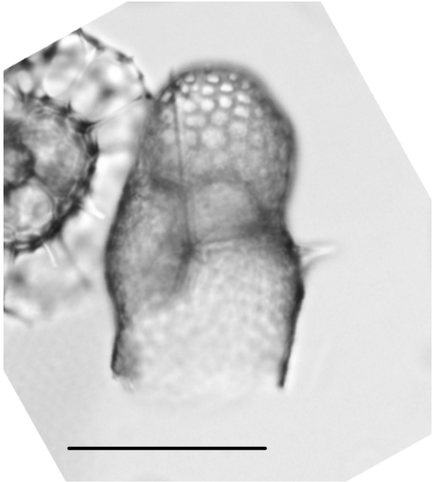

unknown plagonid group C sp 71

Plagiacanthidae (lophophaenid)

Undescribed

Records: 25

This species has a large hump on the upper part of the thorax, and relatively large cephalic arches beneath the internal apical spine. Cephalis is usually sub-quadrate in shape.

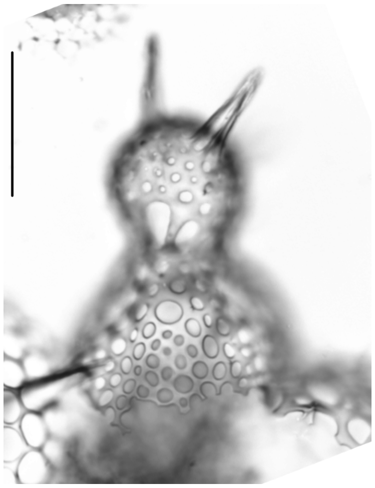

unknown plagonid group C sp 72

Plagiacanthidae (lophophaenid)

Undescribed

Records: 17

This species is distinct for its round cephalis, which is either hyaline or with very small pores. Cephalis has three robust, bladed spines. Thorax is shouldered.

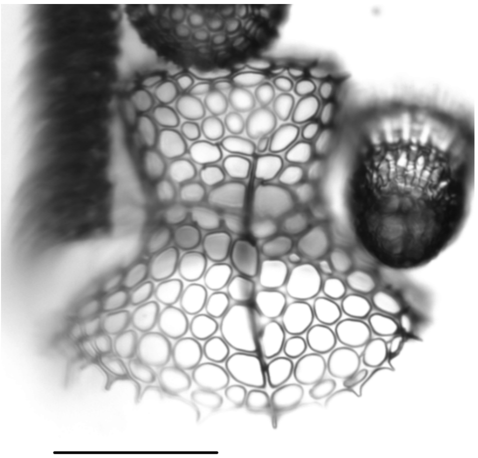

unknown plagonid group C sp 73

Plagiacanthidae (lophophaenid)

Undescribed

Records: 60

This species differs from Lampromitra schultzei in that its cephalis is significantly larger and more angular. It differs from Amphiplecta acrostoma in that its cephalis is wider and fully enclosed. It also has a clear termination at the base of the thorax.

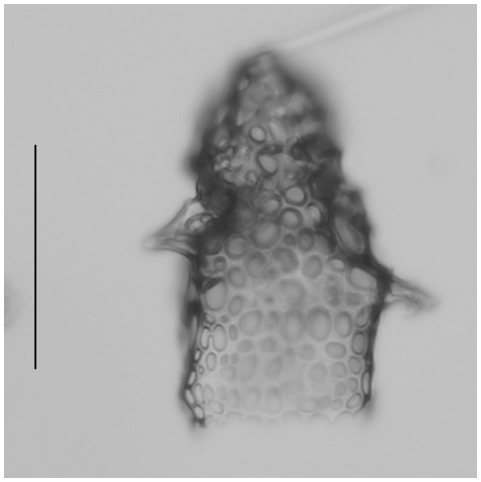

unknown plagonid group C sp 74

Plagiacanthidae (lophophaenid)

Undescribed

Records: 25

Thorasic spine orientation and elongated cephalis resaembles Lophophaena buetschlii, but this species has highly irregular cephalic shape and pore size.

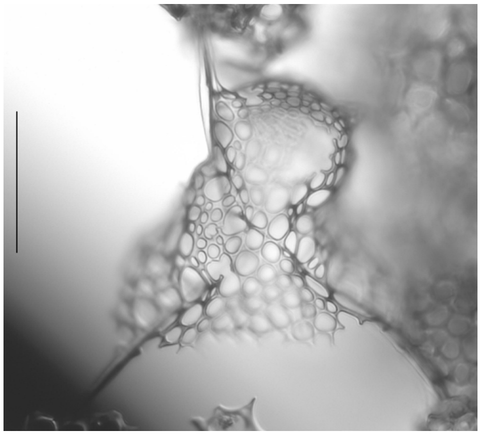

unknown plagonid group C sp 75

Plagiacanthidae (lophophaenid)

Undescribed

Records: 29

This species is defined by its flattened cephalis with two dominant bladed spines, and three strong bladed feet originating from the thorax.

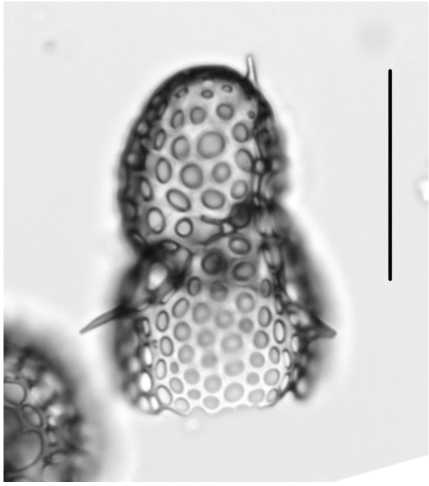

unknown plagonid group C sp 8

Plagiacanthidae (lophophaenid)

Undescribed

Records: 124

This species resembles L. thoracites with its asymmetrical thoracic spines, except that cephalis and upper thorax are significantly more heavily silicified, such that the apical spine is often expressed as a ridge along the cephalis, rather than a dimple.

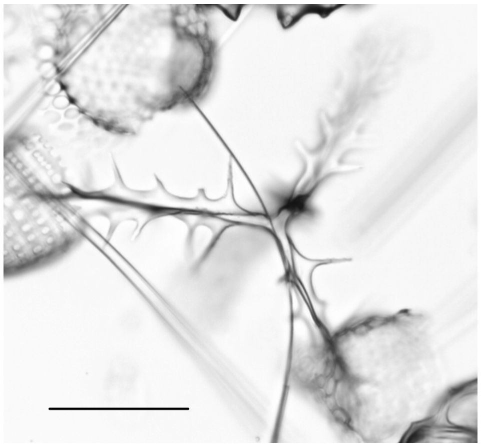

Neosemantis bjoerklundi

Plagiacanthidae (plagonid)

Goll, 1979

Records: 8

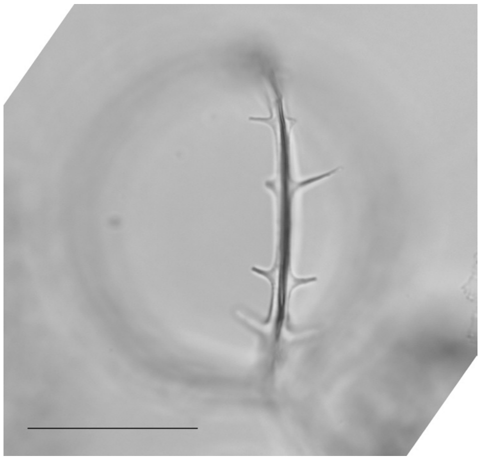

Neosemantis distephanus

Plagiacanthidae (plagonid)

Goll, 1979

Records: 15

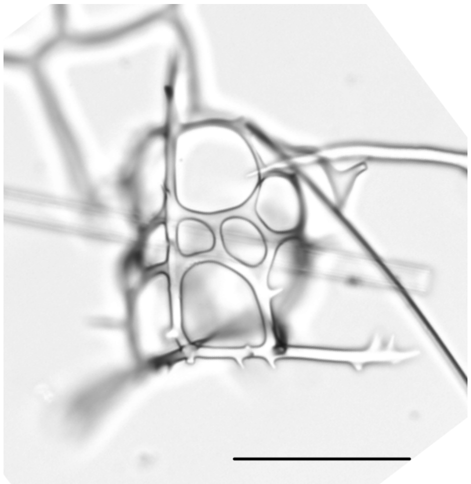

Phormacantha hystrix

Plagiacanthidae (plagonid)

Jørgensen, 1905

Records: 74

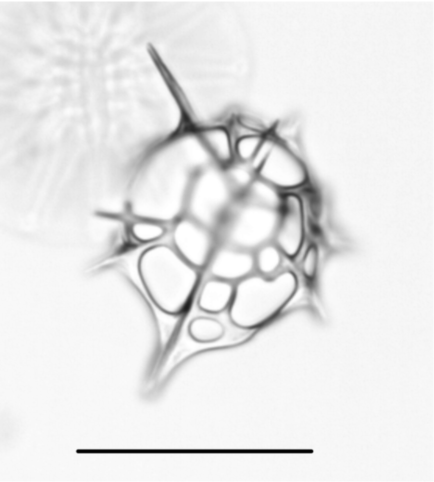

Plectacantha cresmatoplegma

Plagiacanthidae (plagonid)

Nigrini, 1968

Records: 33

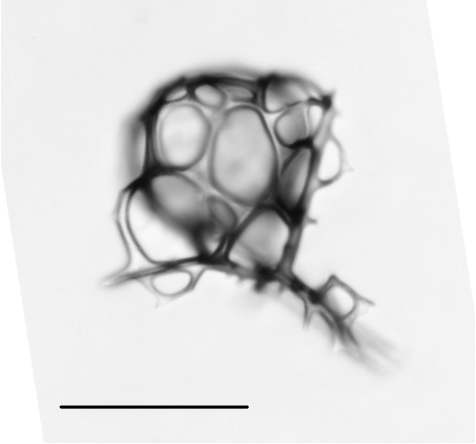

Plectacantha oikiskos

Plagiacanthidae (plagonid)

Jørgensen, 1905

Records: 13

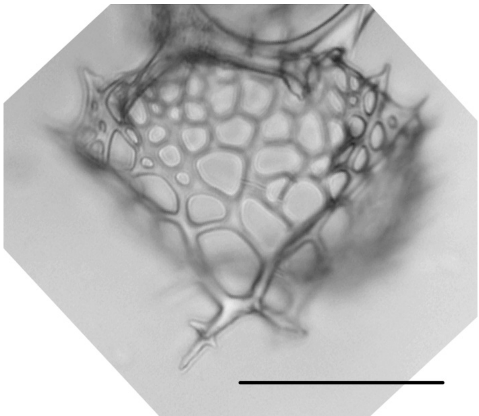

Plectacantha? sp 1

Plagiacanthidae (plagonid)

Undescribed

Records: 28

This species resembles P. cremastoplegma, but it has much smaller pores

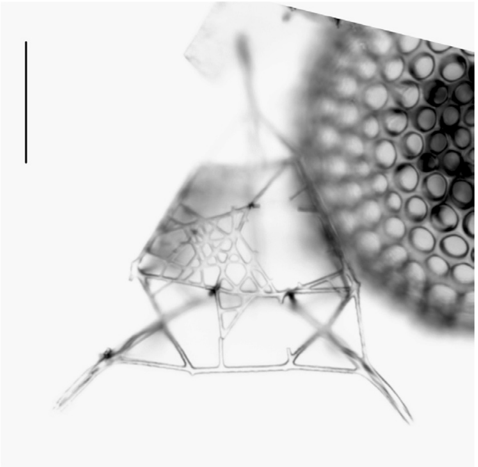

Plectaniscus cortiniscus

Plagiacanthidae (plagonid)

Haeckel, 1887

Records: 20

sensu Muhong et al. 2017

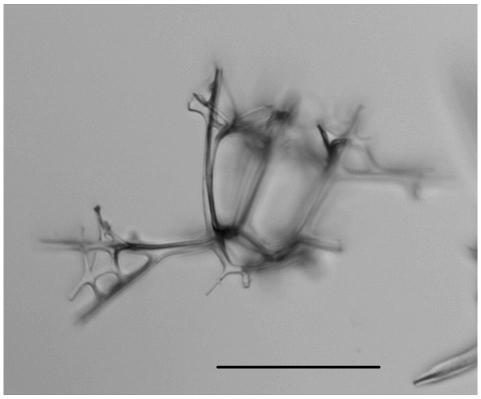

Pseudocubus obeliscus

Plagiacanthidae (plagonid)

Haeckel, 1887

Records: 49

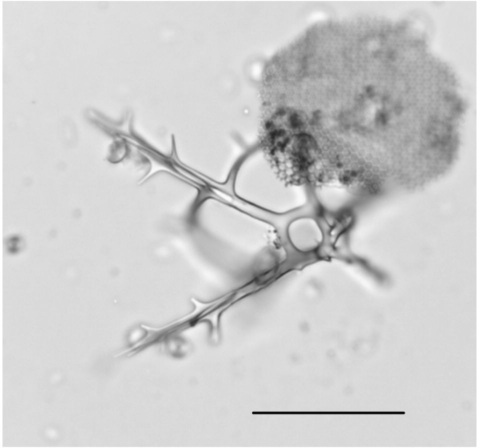

Pseudocubus? sp 1

Plagiacanthidae (plagonid)

Undescribed

Records: 33

The shape of this species is similar to P. obeliscus, but it has three long, thorny spines that extend past the top of the shell.

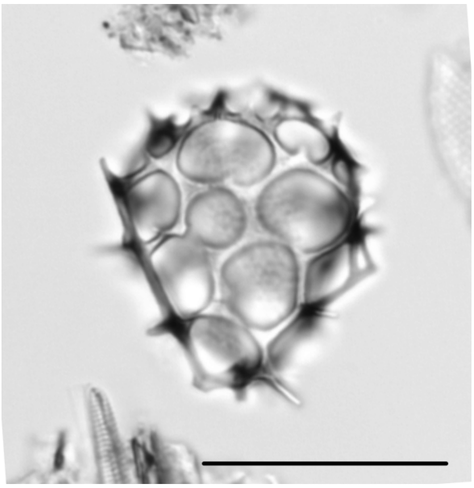

unknown plagonid group A sp 10

Plagiacanthidae (plagonid)

Undescribed

Records: 34

This species has a clear enclosed cephalis, and an apical spine with mirrored pores, suggesting the genus Peridium. However, it differs from described species with its rounded, barbed pores.

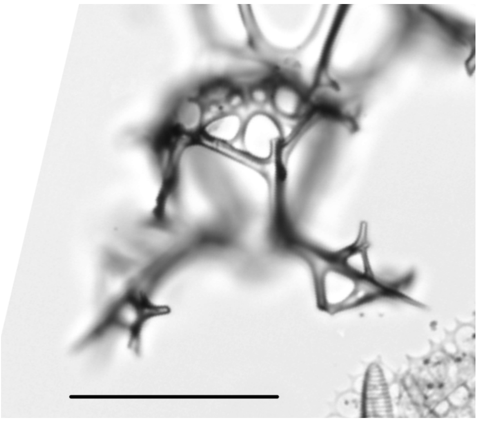

unknown plagonid group A sp 11

Plagiacanthidae (plagonid)

Undescribed

Records: 15

This species is similar to P. obeliscus except that it has a pore lattice on top of the cephalis.

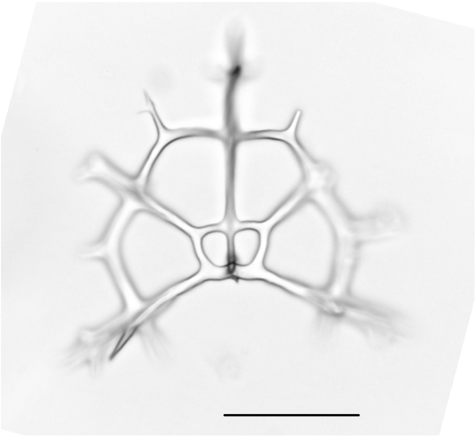

unknown plagonid group A sp 12

Plagiacanthidae (plagonid)

Undescribed

Records: 17

This species has an angular pore structure resembling that of the Pseudocubus genus, but differs in that it lies flat.

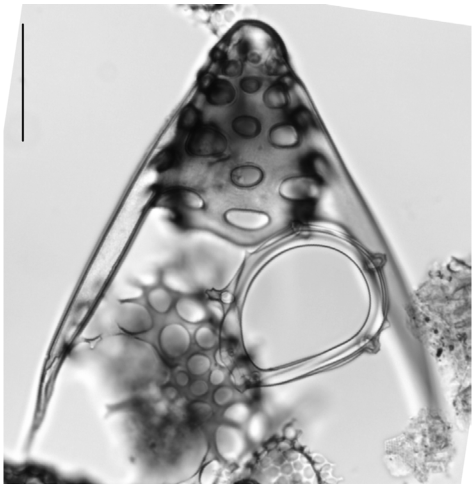

Archipilium macropus

Plagiacanthidae (sethoperid)

(Haeckel) Petrushevskaya & Kozlova, 1972

Records: 17

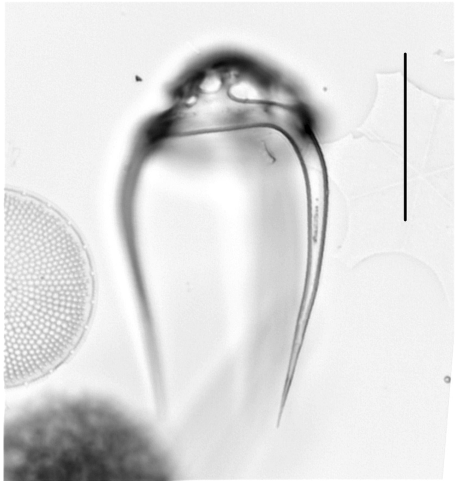

Archipilium orthopterum

Plagiacanthidae (sethoperid)

Haeckel, 1887

Records: 29

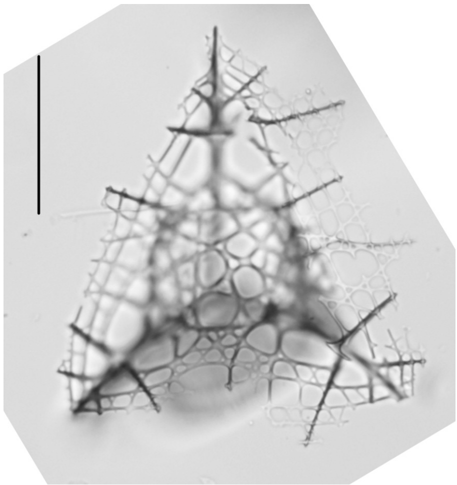

Callimitra carolotae

Plagiacanthidae (sethoperid)

Haeckel, 1887

Records: 50

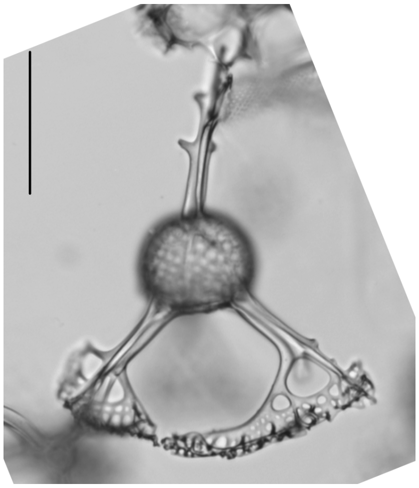

Callimitra sharonae

Plagiacanthidae (sethoperid)

Goll, 1979

Records: 50

Pl. 5, figs. 2-3

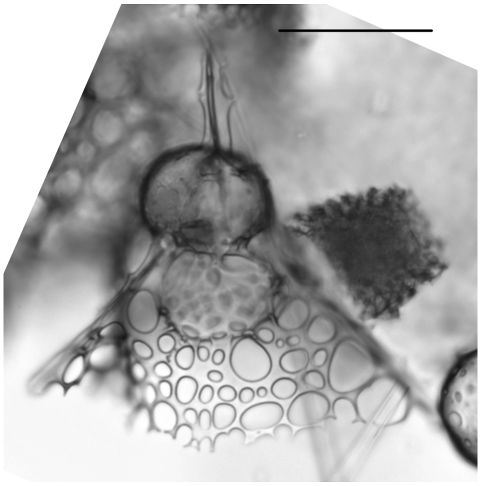

Callimitra sp 1

Plagiacanthidae (sethoperid)

Undescribed

Records: 30

This species has a barbed apical spine and pyramidal lattice structure between the feet.

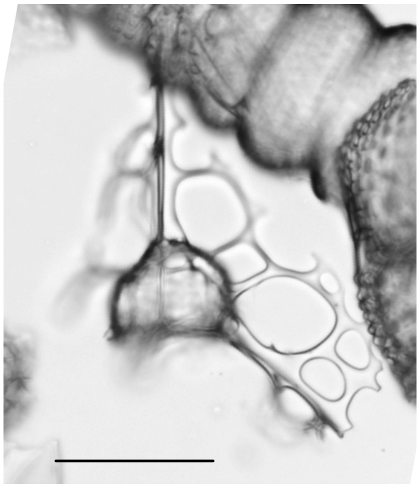

Callimitra sp 2

Plagiacanthidae (sethoperid)

Undescribed

Records: 7

This species differs from the other Callimitra species in that it has very large pores between the feet and apical spines.

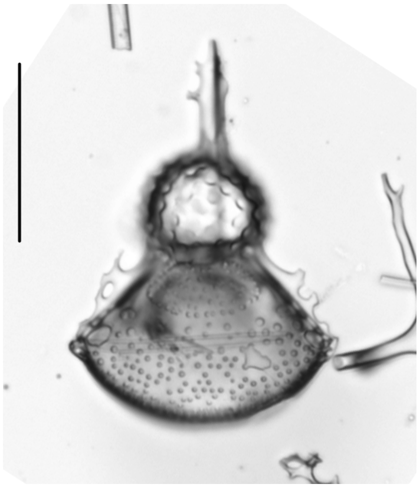

Callimitra sp 3

Plagiacanthidae (sethoperid)

Undescribed

Records: 6

This species has a thickly-silicified cephalis, and an enclosed base with extremely small pores, bordering on hyaline.

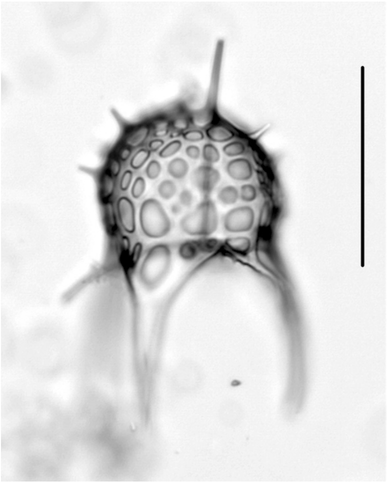

Chitascenium cranites

Plagiacanthidae (sethoperid)

Sugiyama, 1994

Records: 78

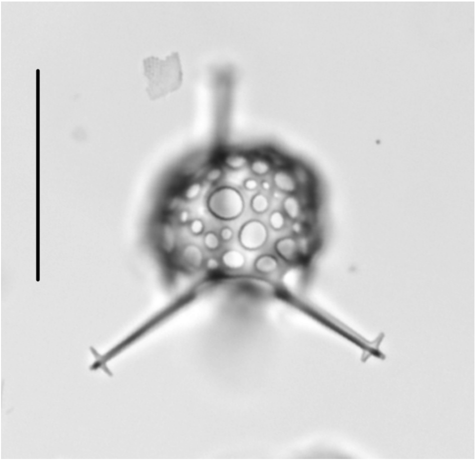

Cladoscenium ancoratum

Plagiacanthidae (sethoperid)

Haeckel, 1887

Records: 54

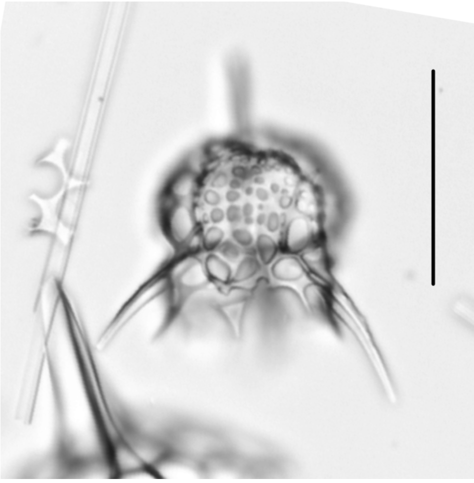

Cladoscenium tricolpium
Plagiacanthidae (sethoperid)
(Haeckel) Jørgensen, 1900
Records: 26

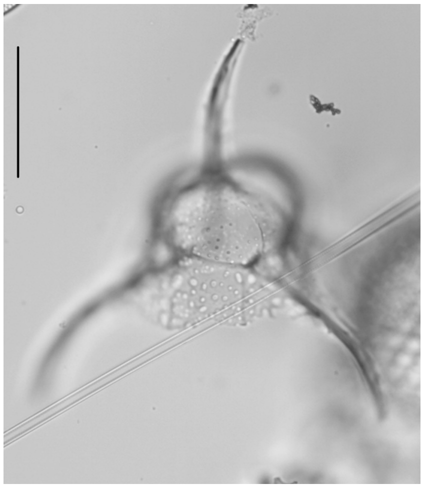

Cladoscenium tricolpium cf
Plagiacanthidae (sethoperid)
Undescribed
Records: 71

This species is similar to C. tricolpium, except that the shell is mostly hyaline.

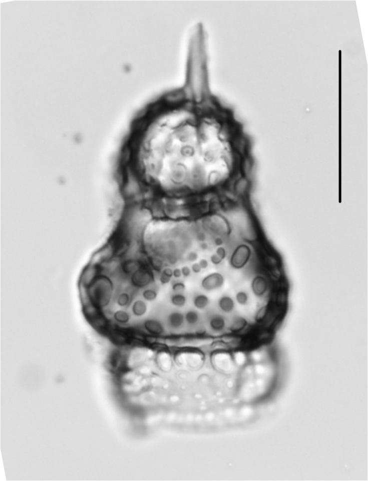

Clathrocanium coarctatum
Plagiacanthidae (sethoperid)
Ehrenberg, 1872
Records: 75

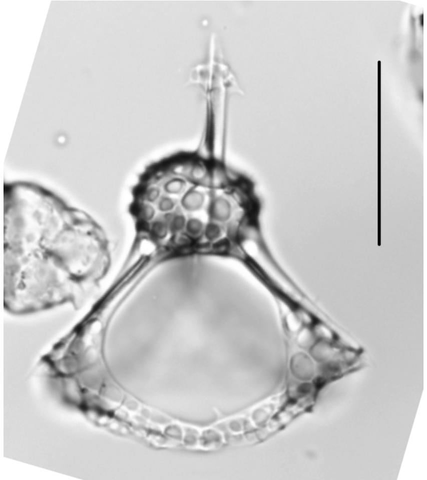

Clathrocanium coarctatum cf
Plagiacanthidae (sethoperid)
Undescribed
Records: 36

This species has a distinctively small cephalis, and the base is latticed rather than thickly silicified, which differentiates it from C. coarctatum

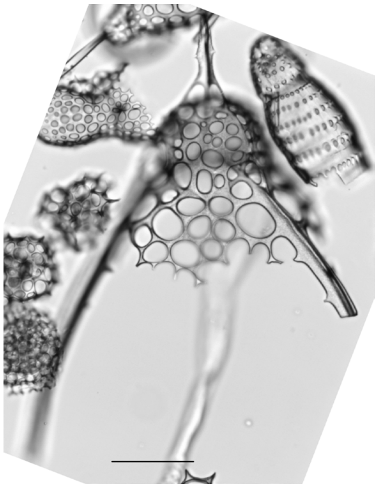

Clathrocorys sugiyamai
Plagiacanthidae (sethoperid)
Renaudie & Lazarus, 2013
Records: 17

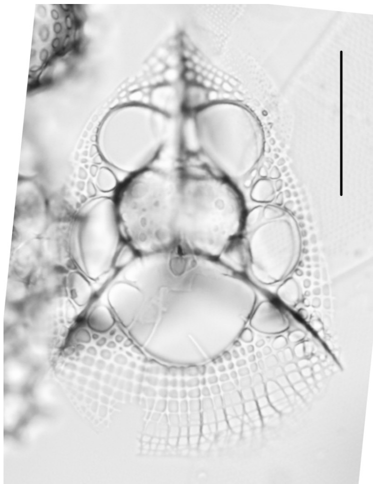

Clathrocorys teuscheri
Plagiacanthidae (sethoperid)
Haeckel, 1887
Records: 74

sensu Goll 1979

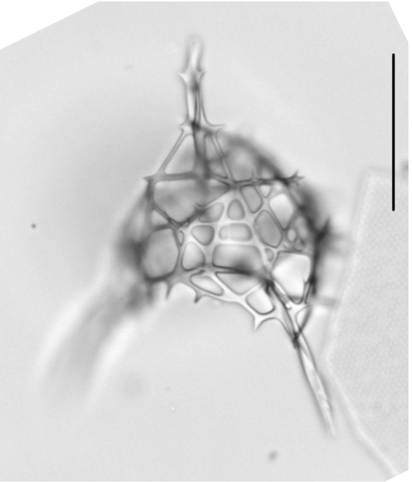

Clathromitra lemi

Plagiacanthidae (sethoperid)

Renaudie & Lazarus, 2013

Records: 6

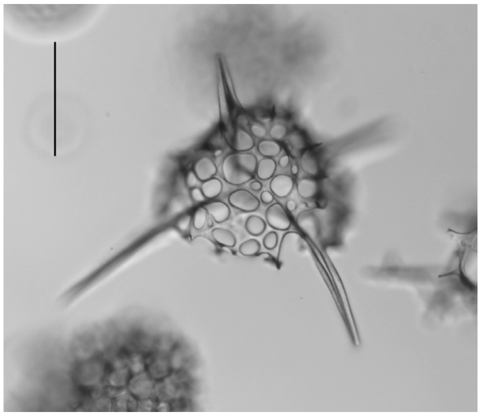

Corythomelissa horrida

Plagiacanthidae (sethoperid)

Petrushevskaya, 1975

Records: 41

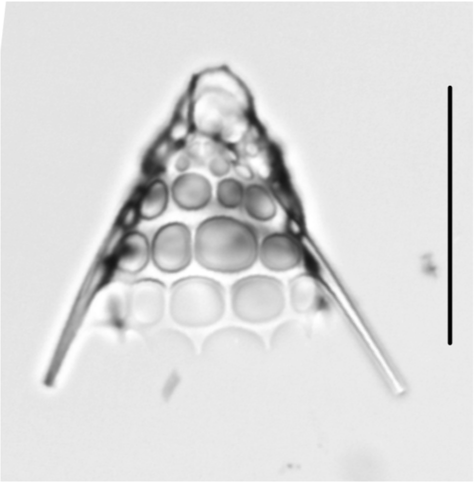

Dictyophimus archipilium

Plagiacanthidae (sethoperid)

Petrushevskaya, 1975

Records: 17

This species has many characteristics in common with A. orthopterum, such as large pores and conical feet. However, it differs in that it has a small, hyaline cephalis that is distinctly separate from the thorax.

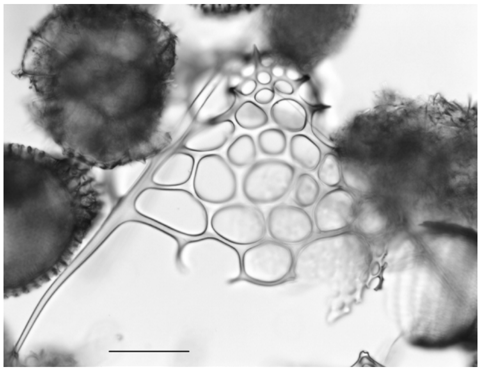

Dictyophimus platycephalis sensu Haeckel

Plagiacanthidae (sethoperid)

Haeckel, 1887

Records: 16

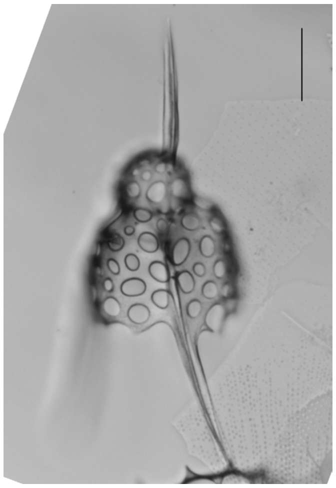

Pseudodictyophimus gracilipes

Plagiacanthidae (sethoperid)

Caulet, 1979

Records: 109

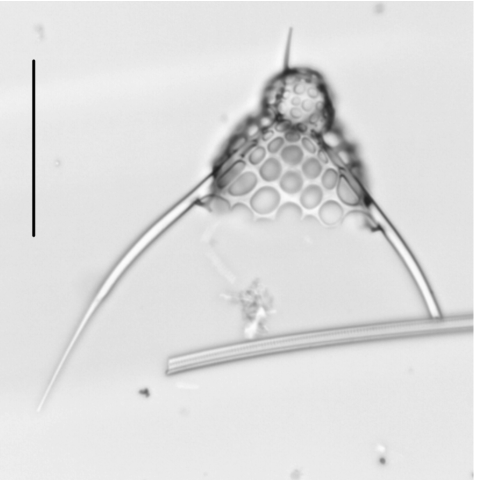

Pseudodictyophimus gracilipes cf

Plagiacanthidae (sethoperid)

Undescribed

Records: 28

Similar to P. gracilipes except that legs are conical rather than bladed, and tend to be more straight than P. gracilipes.

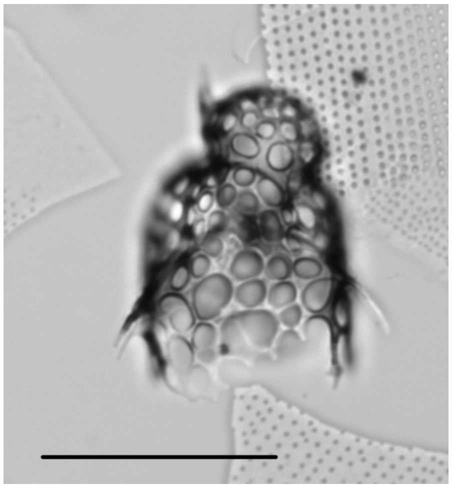

Pseudodictyophimus gracilipes forma bicornis

Plagiacanthidae (sethoperid)

Petrushevskaya, 1971

Records: 33

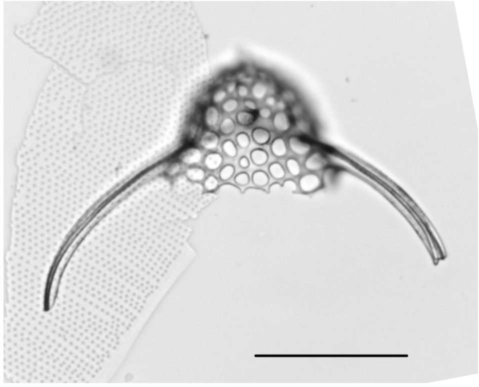

Pseudodictyophimus platycephalis

Plagiacanthidae (sethoperid)

(Haeckel) 1887

Records: 77

sensu Benson 1966

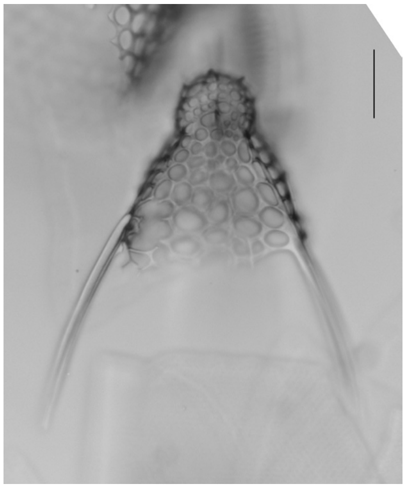

Pseudodictyophimus? sp 2

Plagiacanthidae (sethoperid)

Undescribed

Records: 35

Long thorax, hemispherical, thorny cephalis, bladed feet, with thorax extending below feet

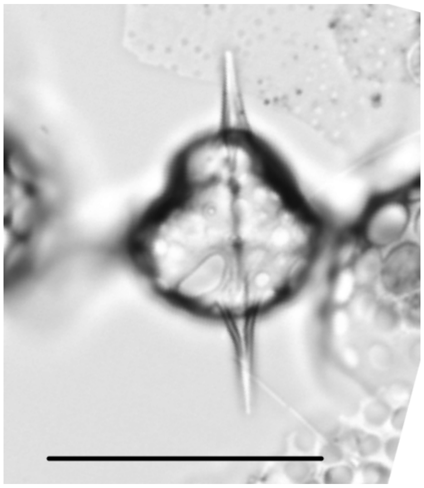

Pseudodictyophimus? sp 3

Plagiacanthidae (sethoperid)

Undescribed

Records: 24

Very small species, with an enclosed thorax.

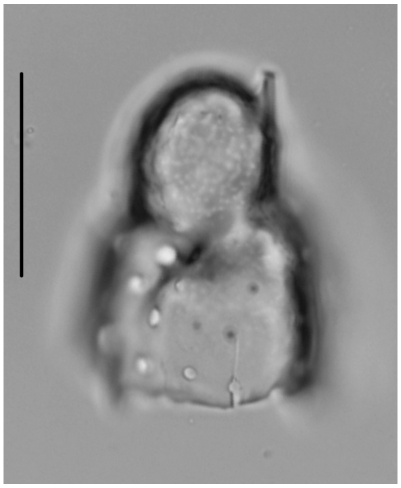

Pseudodictyophimus? sp 6

Plagiacanthidae (sethoperid)

Undescribed

Records: 7

Small species with a completely hyaline cephalis.

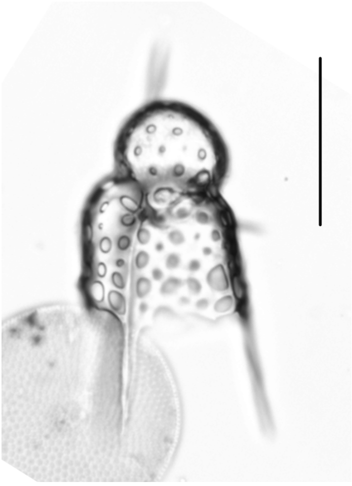

Pseudodictyophimus? sp 7

Plagiacanthidae (sethoperid)

Undescribed

Records: 15

Relatively long, lobed thorax, otherwise similar to P. gracilipes.

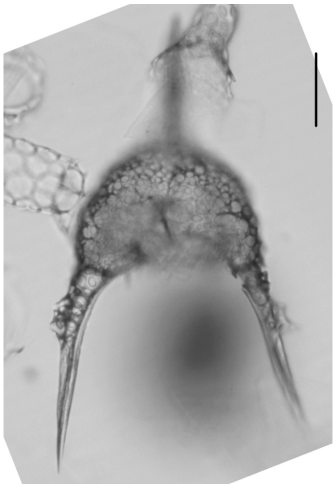

Pteroscenium pinnatum
Plagiacanthidae (sethoperid)
Haeckel, 1887
Records: 39

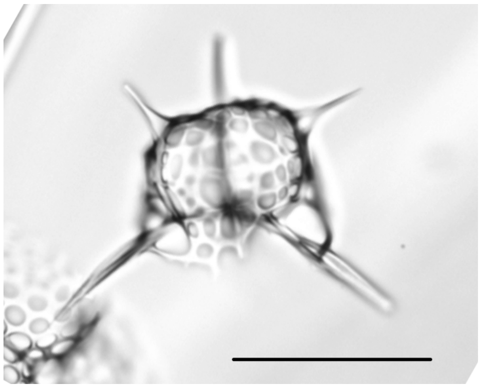

unknown plagonid cf horrida
Plagiacanthidae (sethoperid)
Undescribed
Records: 75

Bi-lobed cephalis with strong apical spine and feet. Can have two additional spines on either side of the cephalis.

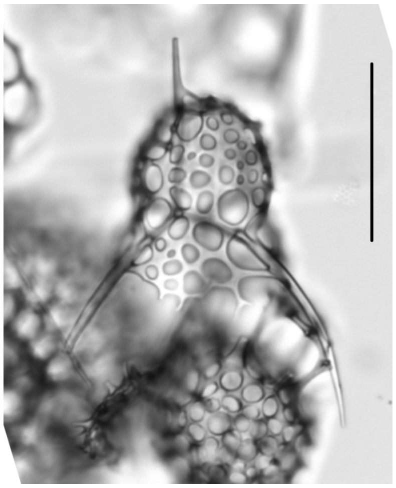

unknown plagonid cf horrida sp 2
Plagiacanthidae (sethoperid)
Undescribed
Records: 18

Elongated bi-lobed cephalis with conical apical spines and feet.

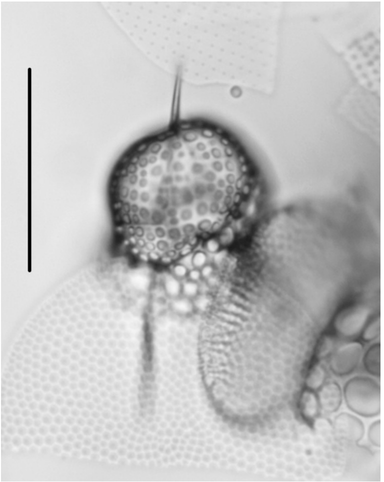

unknown plagonid group B sp 1
Plagiacanthidae (sethoperid)
Undescribed
Records: 23

Large, bulbous cephalis with small pores, three feet, and conical apical spine.

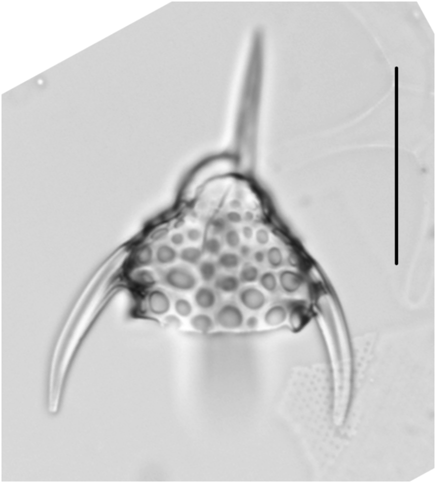

unknown plagonid group B sp 10
Plagiacanthidae (sethoperid)
Undescribed
Records: 178

Relatively large, hyaline cephalis with one large lobe and one small lobe, and a strong apical spine coming from the center. Three curved feet coming from latticed thorax.

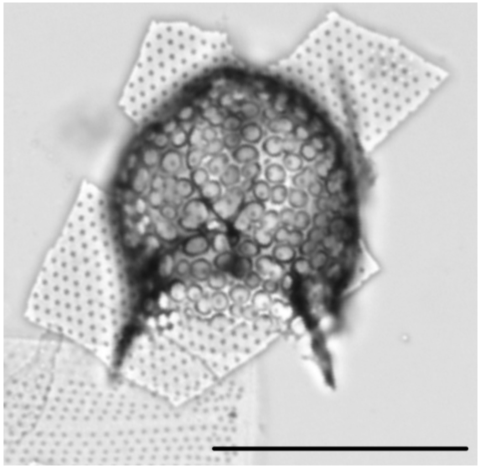

unknown plagonid group B sp 13
Plagiacanthidae (sethoperid)
Undescribed
Records: 31

small, irregular pores have a distinct “hole-punch” texture. This species has a very large cephalis with no distinguishable lobes, 3 feet, and an apical spine that cuts through the center of the cephalis.

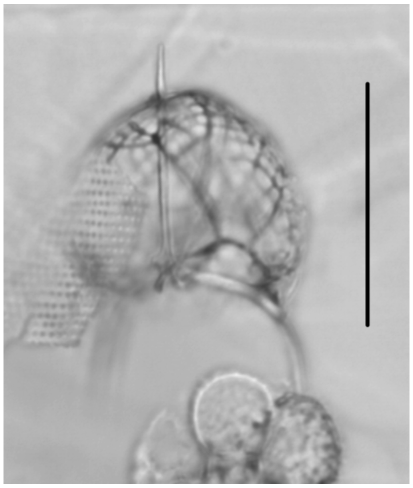

unknown plagonid group B sp 13 cf

Plagiacanthidae (sethoperid)

Undescribed

Records: 15

Similar to unknown plagonid group B sp 13, except that the shell exterior is covered with angular lineations, crossing over the fine pore structure.

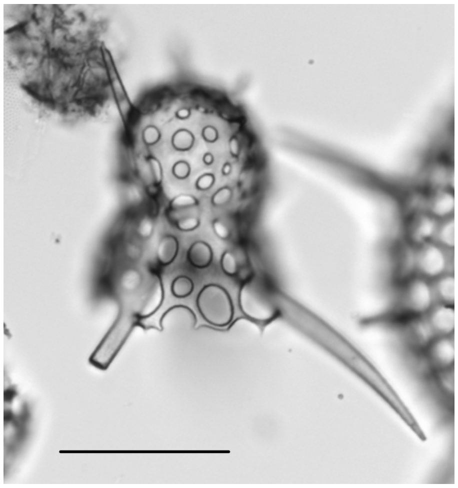

unknown plagonid group B sp 14

Plagiacanthidae (sethoperid)

Undescribed

Records: 51

Hemispherical cephalis, often with several spines, evidence of a thorax, and three conical feet.

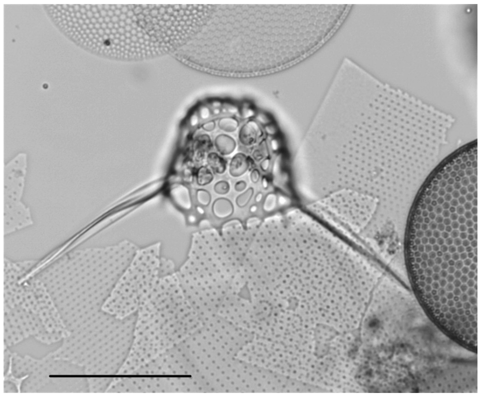

unknown plagonid group B sp 15

Plagiacanthidae (sethoperid)

Undescribed

Records: 69

No clear distinction between cephalis and thorax, this species has a rounded, flat cephalis and three bladed feet.

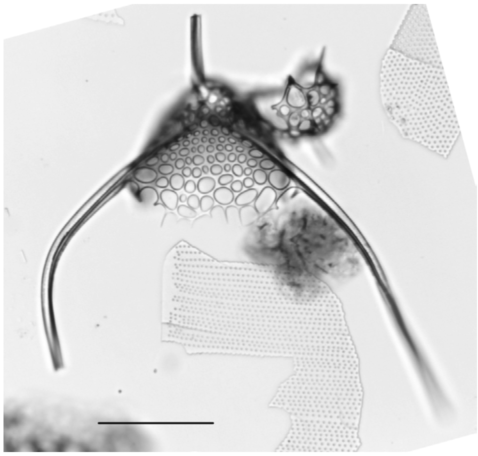

unknown plagonid group B sp 16

Plagiacanthidae (sethoperid)

Undescribed

Records: 54

This species resembles a larger version of unknown plagonid group B sp 10, except that in addition to its significantly larger size, it also has a much broader thorax and very broad, curved feet.

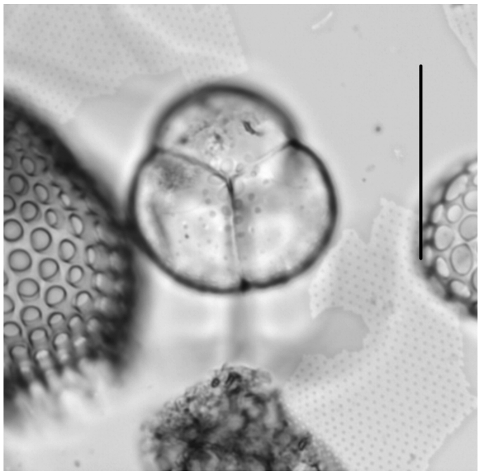

unknown plagonid group B sp 17

Plagiacanthidae (sethoperid)

Undescribed

Records: 35

The cephalis of this species has three identical lobes, almost entirely hyaline, but with a few scattered pores around the suture lines. This species has three feet that bifurcate at their bases.

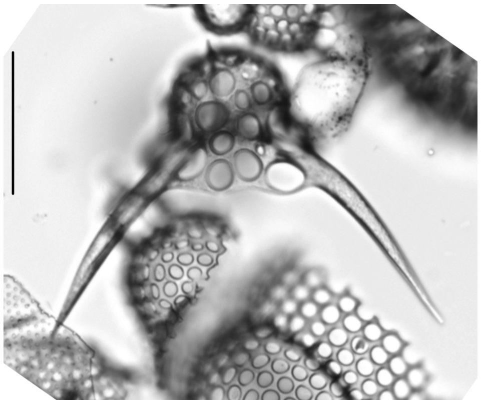

unknown plagonid group B sp 19

Plagiacanthidae (sethoperid)

Undescribed

Records: 31

This is a large and heavily silicified species; it differs from A. orthopterum in that it has a relatively larger cephalis, the cephalis has an apical spine, and both thorax and cephalis exhibit the same size and distribution of pores.

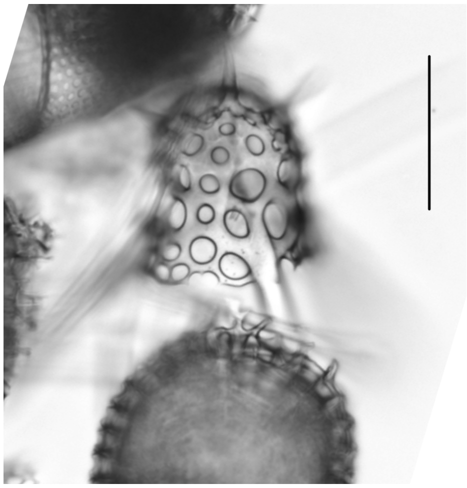

unknown plagonid group B sp 19 cf

Plagiacanthidae (sethoperid)

Undescribed

Records: 10

This species is similar to unknown plagonid group B sp 19, except that it has multiple short, strong spines protruding from the cephalis.

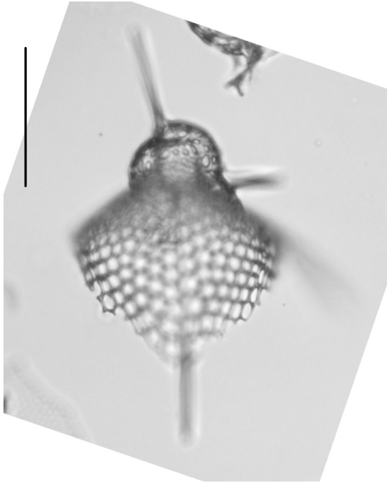

unknown plagonid group B sp 2

Plagiacanthidae (sethoperid)

Undescribed

Records: 8

This species has very fine, regular pores throughout pyramid-shaped thorax. Cephalis has two strong spines protruding from the apex and horizontally the base.

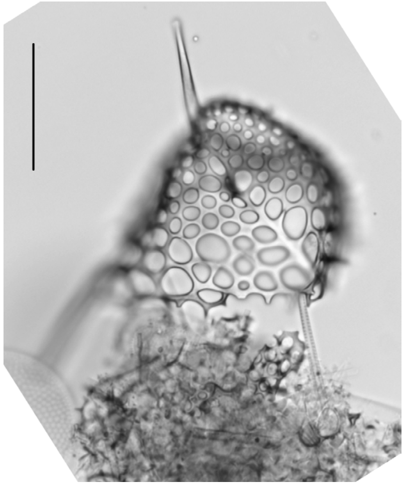

unknown plagonid group B sp 22

Plagiacanthidae (sethoperid)

Undescribed

Records: 11

This species has a boxy, irregular-shaped thorax, unlike the other species described here. Indistinct cephalis blends into thorax, with a conical apical spine and three feet.

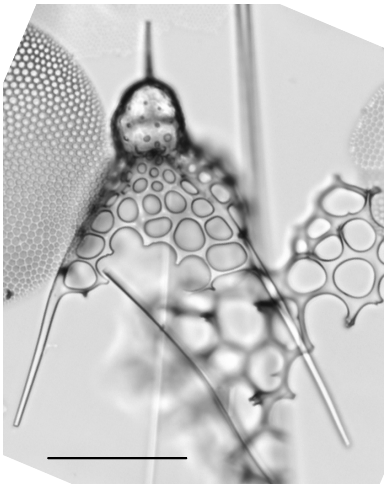

unknown plagonid group B sp 24

Plagiacanthidae (sethoperid)

Undescribed

Records: 18

This species has a small, thumb-shaped cephalis, with much smaller pores than those present on the thorax. Thin apical spine and feet.

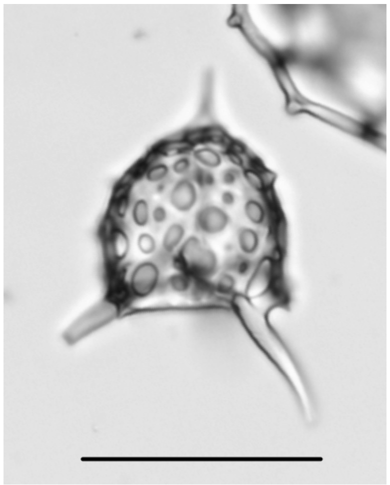

unknown plagonid group B sp 25

Plagiacanthidae (sethoperid)

Undescribed

Records: 9

This species somewhat resembles unknown plagonid group B sp 19 and sp 19 cf, except that the shape of its cephalis is more elongated, and the overall size of the species is smaller.

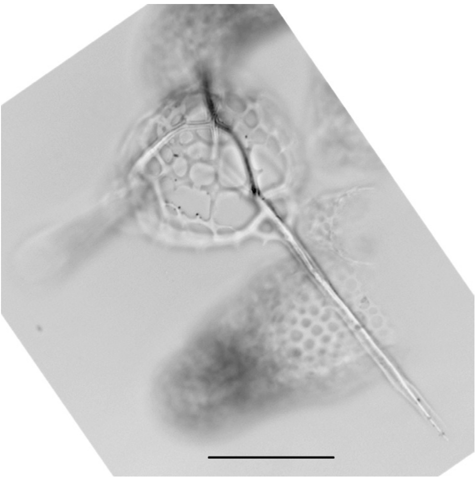

unknown plagonid group B sp 3

Plagiacanthidae (sethoperid)

Undescribed

Records: 11

This species has a distinctively flat cephalis, differing from P. platycephalis with its thorny feet and set of two large pores at the base of each foot.

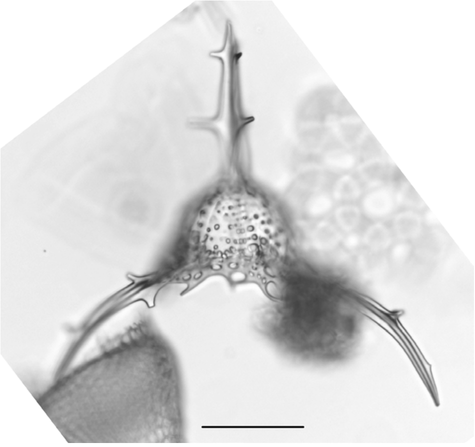

unknown plagonid group B sp 9

Plagiacanthidae (sethoperid)

Undescribed

Records: 70

This species has three broadly curving feet with strong thorns on both feet and apical spine. Thorax is usually poorly developed, with jagged termination. Differs from C. sugiyami in more compressed shape, and significantly smaller, more irregular pores.

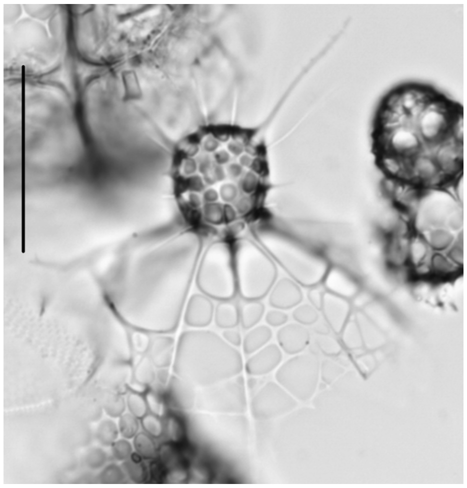

Arachnocorys circumtexta

Plagiacanthidae (sethophormid)

Haeckel, 1860

Records: 60

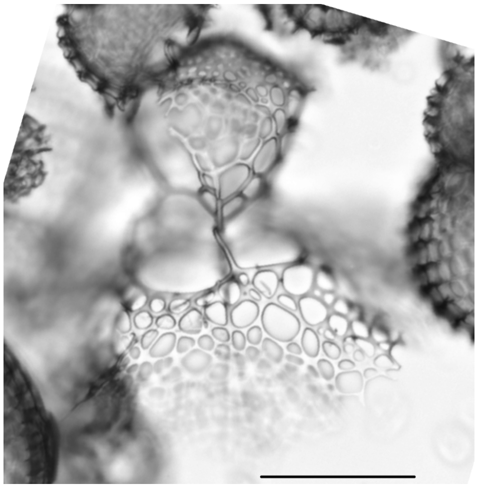

Arachnocorys circumtexta cf

Plagiacanthidae (sethophormid)

Undescribed

Records: 17

Similar to A. circumtexta, except that cephalis is relatively larger, and lower part of thorax is more finely latticed with smaller, less angular pores.

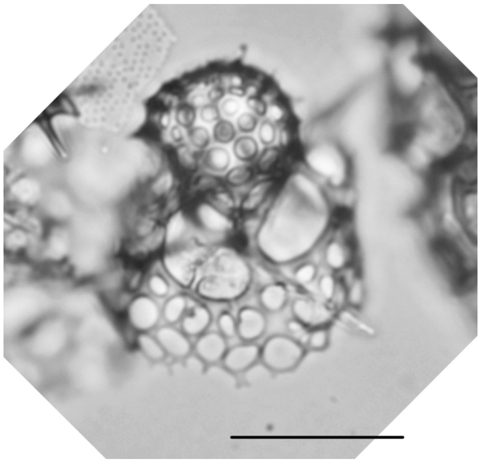

Arachnocorys pentacantha cf sp 1

Plagiacanthidae (sethophormid)

Undescribed

Records: 70

This species has a small cephalis, large openings at the base of cephalis, three strong thoracic spines, and strong, bladed apical spine.

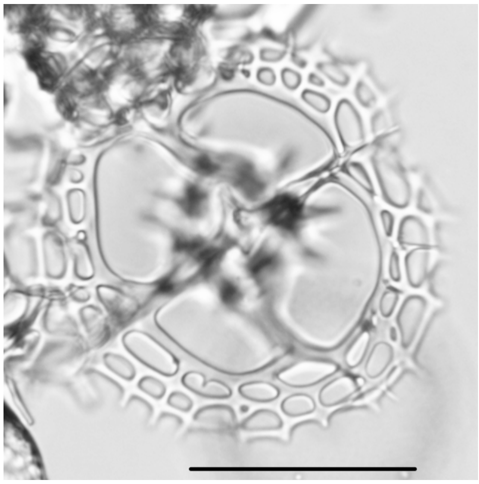

Arachnocorys? sp 1

Plagiacanthidae (sethophormid)

Undescribed

Records: 21

This species has a tiny, thorny cephalis, surrounded by 4 large openings between cephalis and thorax. Thorax is wide, with rectangular pores and spines.

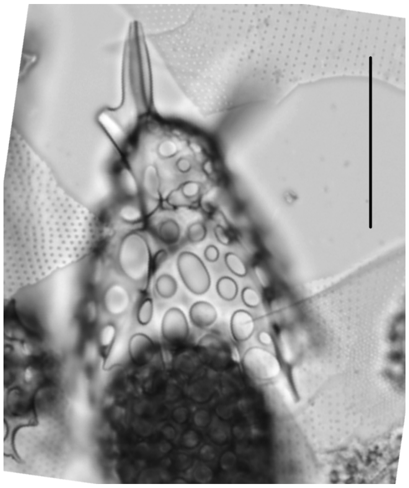

Ceratocyrtis cantharoides

Plagiacanthidae (sethophormid)

Sugiyama & Furutani, 1992

Records: 14

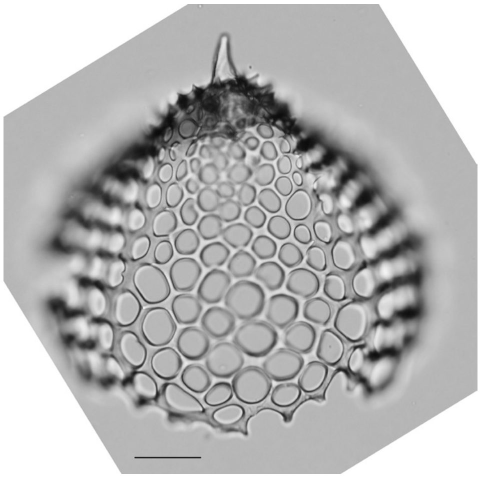

Ceratocyrtis cucullaris

Plagiacanthidae (sethophormid)

(Ehrenberg) Butschli, 1882

Records: 15

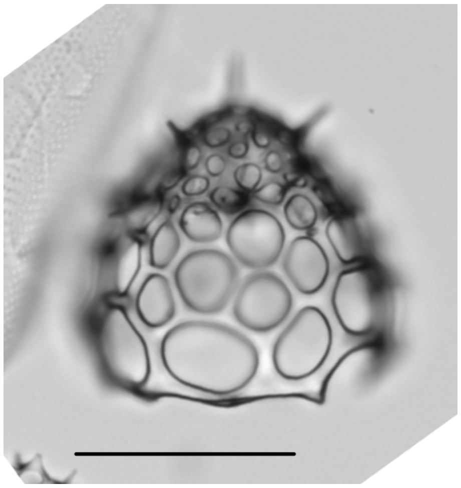

Ceratocyrtis histricosa

Plagiacanthidae (sethophormid)

(Jørgensen) Petrushevskaya, 1971

Records: 61

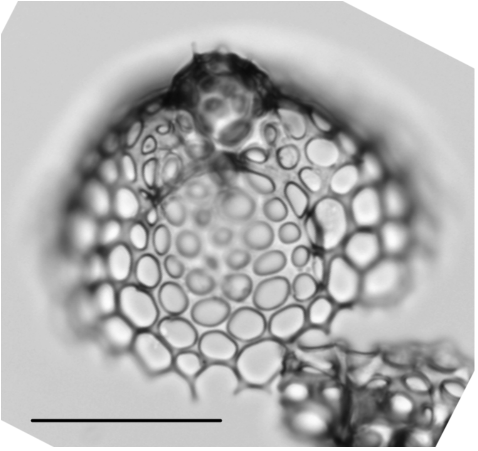

Ceratocyrtis sp 1

Plagiacanthidae (sethophormid)

Undescribed

Records: 15

This species has a smooth surface and three large lobes on thorax, separated by internal spines.

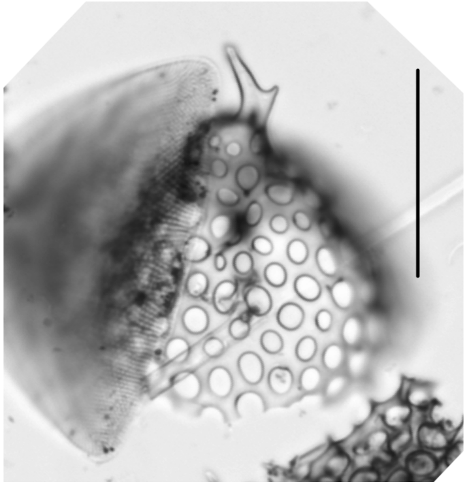

Ceratocyrtis sp 10

Plagiacanthidae (sethophormid)

Undescribed

Records: 65

This species typically has a slightly thorny cephalis and thorax, with small, regular pores, and one slight dimple on thorax that does not appear to be associated with any spines. Cephalus has one forked spine.

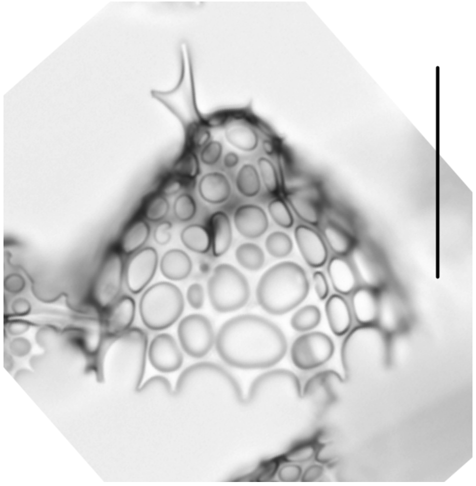

Ceratocyrtis sp 10 cf

Plagiacanthidae (sethophormid)

Undescribed

Records: 15

This species is similar to Ceratocyrtis sp 10, except that pores on thorax are significantly larger and more irregular in shape.

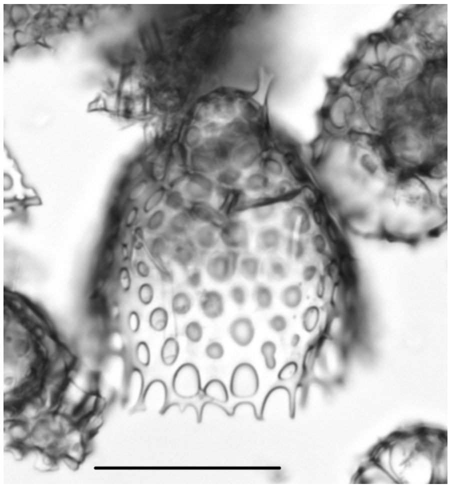

Ceratocyrtis sp 11

Plagiacanthidae (sethophormid)

Undescribed

Records: 28

This species resembles Ceratocyrtis sp 10, except that the thorax is more long and narrow, often with teeth present at the base.

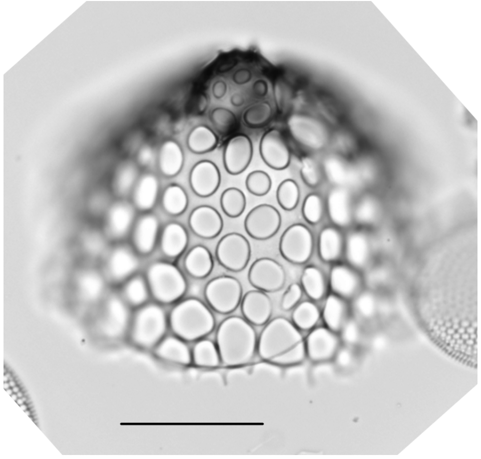

Ceratocyrtis sp 2

Plagiacanthidae (sethophormid)

Undescribed

Records: 106

This species resembles Ceratocyrtis sp 1, except that it does not have a pronounced lobe on the thorax.

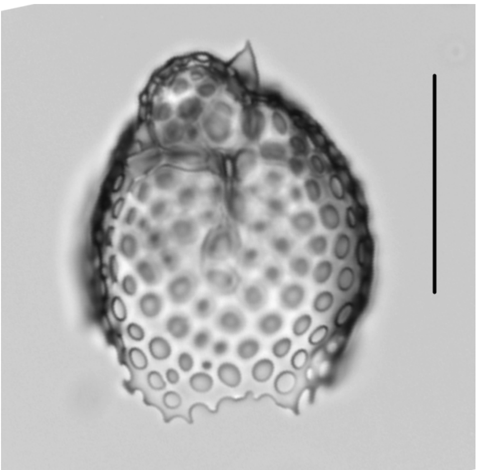

Ceratocyrtis sp 5

Plagiacanthidae (sethophormid)

Undescribed

Records: 19

This species has a strong conical apical spine that protrudes out the side of the cephalis, and is unique for its tapering thorax. Unlike the other Ceratocyrtis species, this one has its widest point approximately 1/3 down the thorax.

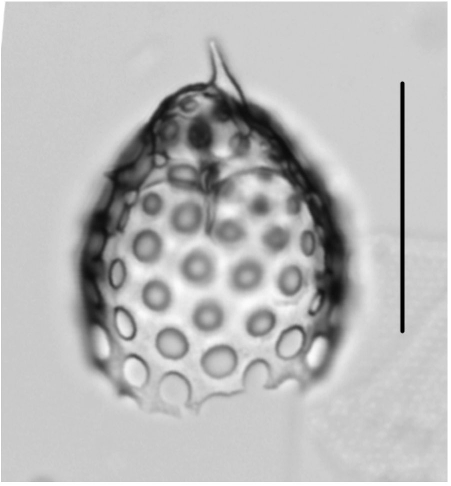

Ceratocyrtis sp 5 cf

Plagiacanthidae (sethophormid)

Undescribed

Records: 31

This species resembles a petite version of Ceratocyrtis sp 5, about 1/2 its size, and is sometimes also more hyaline.

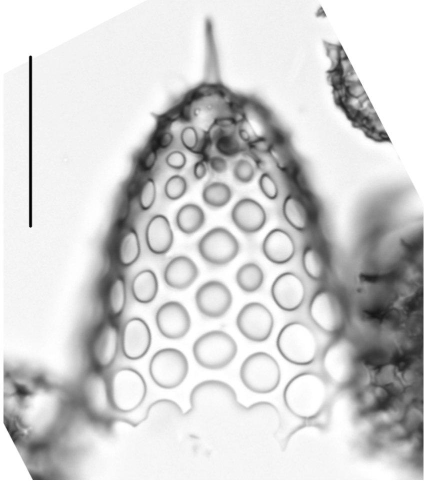

Ceratocyrtis sp 6

Plagiacanthidae (sethophormid)

Undescribed

Records: 53

This species has a long, conical thorax, with pores increasing in size toward the base.

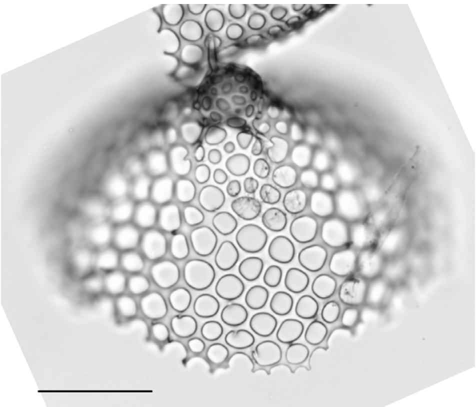

Ceratocyrtis sp 7 cf

Plagiacanthidae (sethophormid)

Undescribed

Records: 41

This species is unique for its relatively heavily silicified cephalis, and very large, wide, thorax with large, semi-irregular pores.

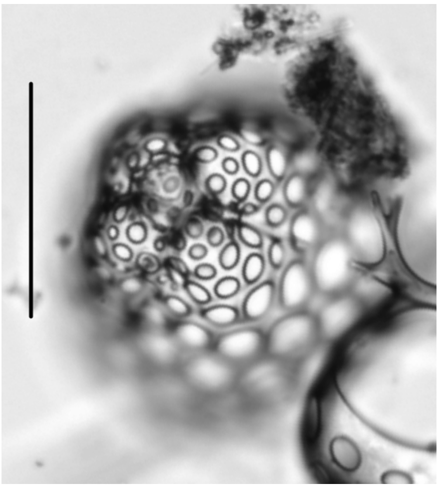

Ceratocyrtis sp 8

Plagiacanthidae (sethophormid)

Undescribed

Records: 40

This species is similar to Ceratocyrtis sp 6, except differs with its four prominent lumps on the uppermost part of the thorax.

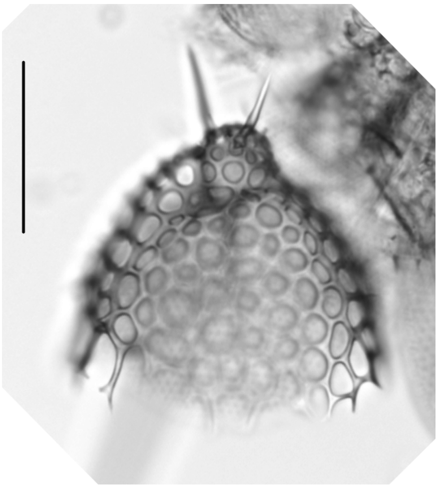

Ceratocyrtis sp 9
Plagiacanthidae (sethophormid)
Undescribed
Records: 43

This species has two long, thin conical spines on its cephalis, with large, semi-irregular pores on the thorax.

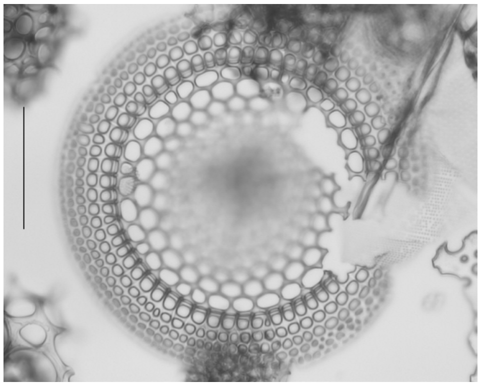

Eucecryphalaus sestrodiscus
Plagiacanthidae (sethophormid)
Haeckel, 1887
Records: 25

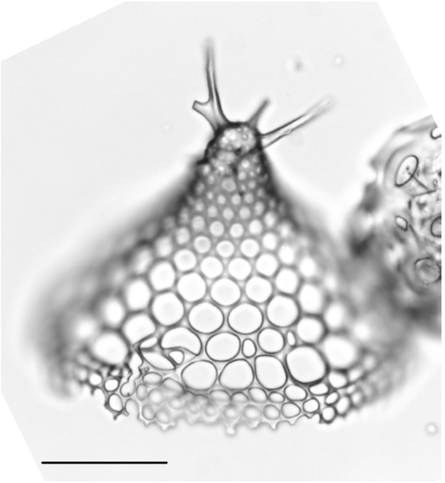

Eucecryphalus cervus
Plagiacanthidae (sethophormid)
(Ehrenberg) Petrushevskaya, 1971
Records: 148

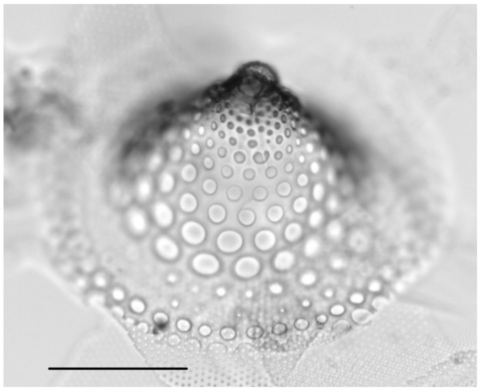

Eucecryphalus cervus cf
Plagiacanthidae (sethophormid)
Undescribed
Records: 36

Like E. cervus, except that pores at base of skirt are round, and rather than a suture at the base, there is a smooth hyaline area.

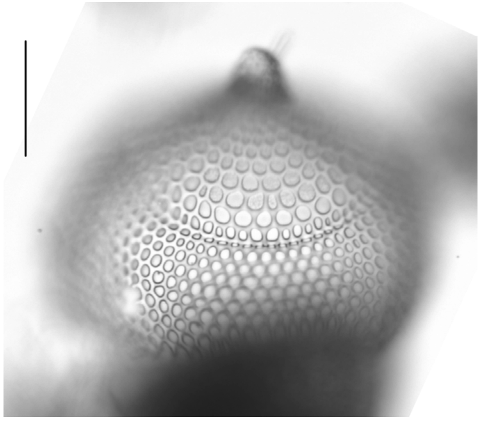

Eucecryphalus clinatus
Plagiacanthidae (sethophormid)
Takahashi, 1991
Records: 43

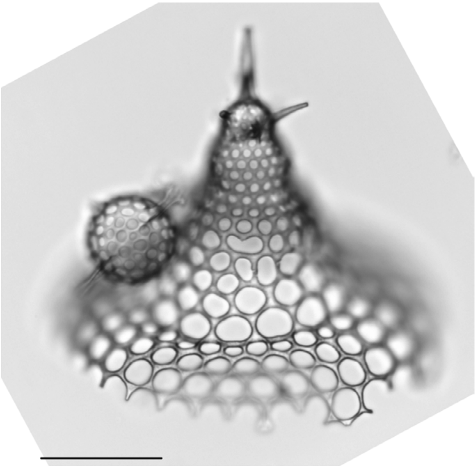

Eucecryphalus clinatus cf
Plagiacanthidae (sethophormid)
Undescribed
Records: 65

This species is relatively large, with a two-segmented thorax, and large round pores around the base of the skirt. This feature differentiates it from E. europae, which has square pores at the base of the skirt.

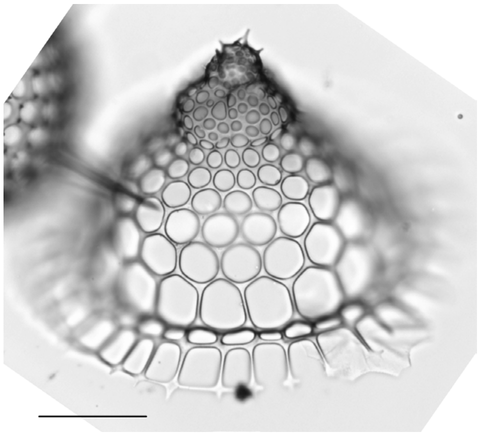

Eucecryphalus europae
Plagiacanthidae (sethophormid)
(Haeckel) Takahashi, 1991
Records: 131

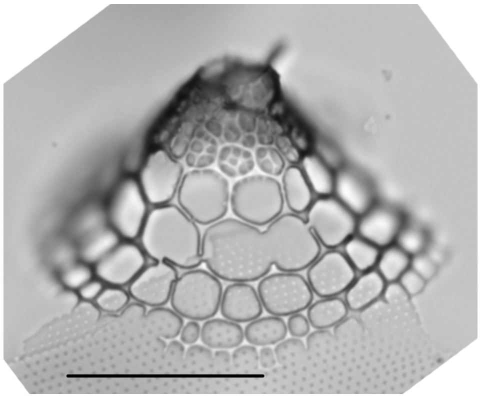

Eucecryphalus gegenbauri
Plagiacanthidae (sethophormid)
Haeckel, 1860
Records: 26

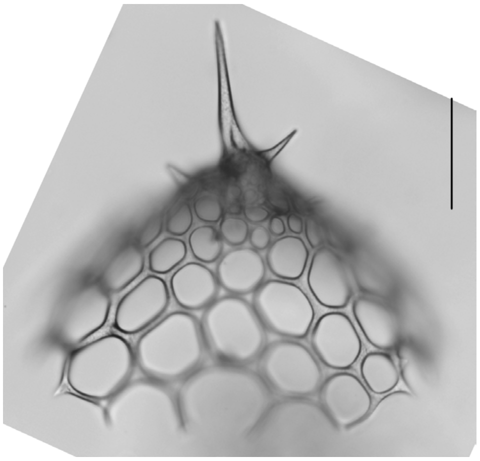

Lampromitra huxleyi
Plagiacanthidae (sethophormid)
Haeckel, 1879
Records: 20

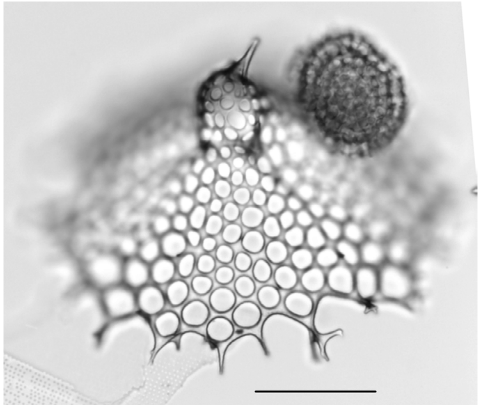

Lampromitra quadricuspis
Plagiacanthidae (sethophormid)
Haeckel, 1887
Records: 86

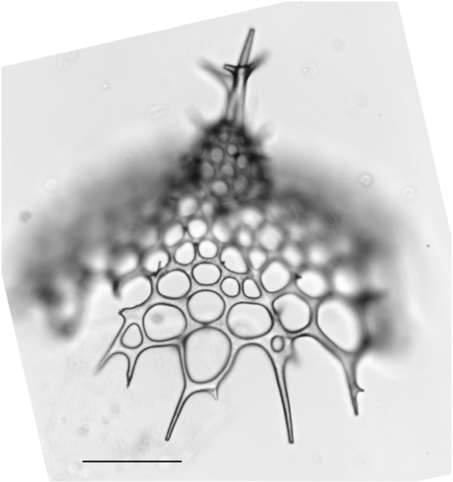

Lampromitra quadricuspis cf
Plagiacanthidae (sethophormid)
Undescribed
Records: 47

This species is similar to L. quadricuspis, but differs in that thoracic pores become larger toward the base, whereas in L. quadricuspis, pores remain approximately the same size throughout the thorax.

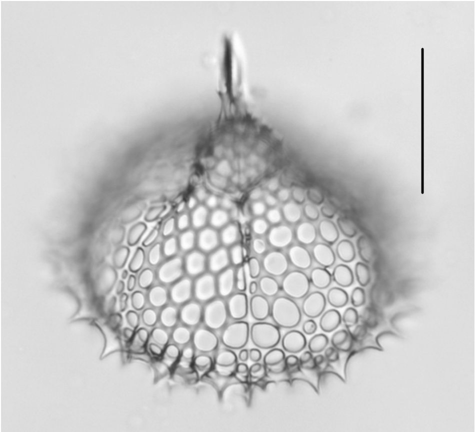

Lampromitra schultzei
Plagiacanthidae (sethophormid)
(Haeckel) Takahashi, 1991
Records: 75

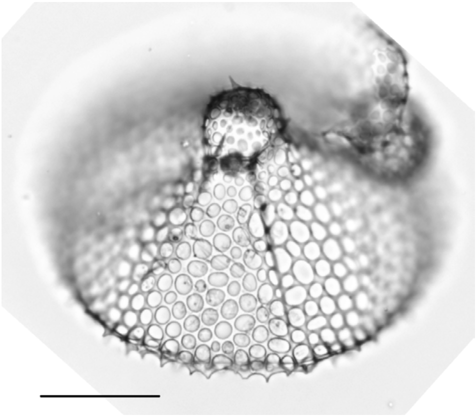

Lampromitra schultzei cf

Plagiacanthidae (sethophormid)

Undescribed

Records: 65

Similar to L. schultzei, except for its distinct, large, and irregular cephalis.

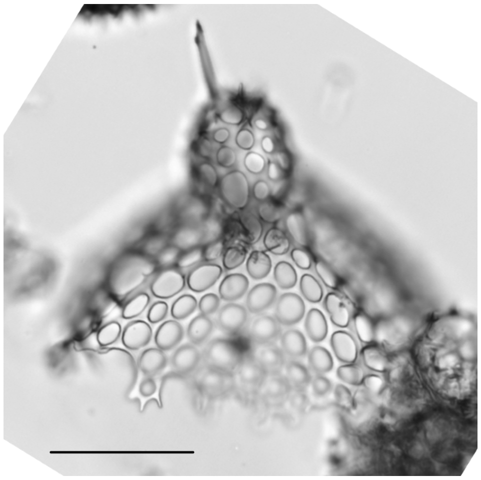

Lampromitra sp 1

Plagiacanthidae (sethophormid)

Undescribed

Records: 45

This species has a long, narrow cephalis, and a wavy, lobed thorax with ribs.

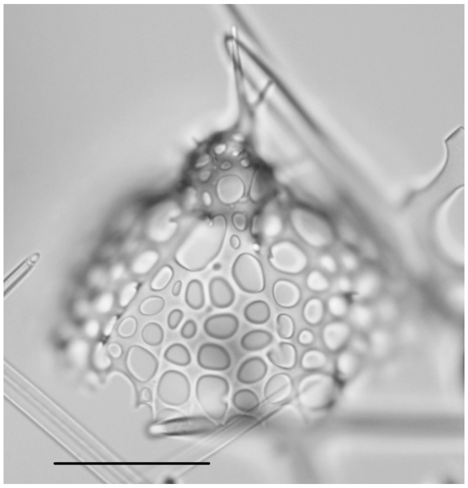

Lampromitra spinosiretis

Plagiacanthidae (sethophormid)

Takahashi, 1991

Records: 64

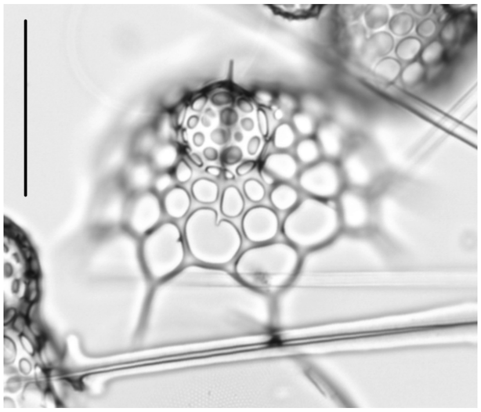

Tetraphormis dodecaster

Plagiacanthidae (sethophormid)

(Haeckel) Takahashi, 1991

Records: 61

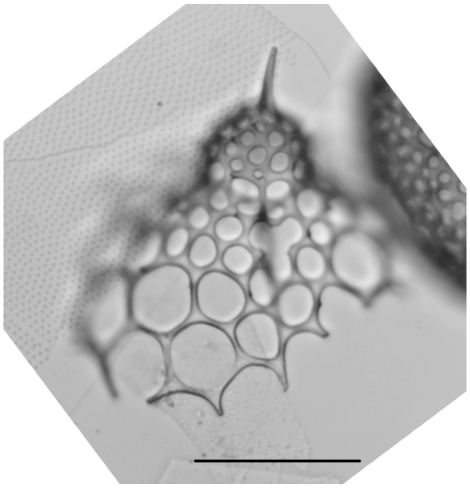

unknown plagonid group E sp 11

Plagiacanthidae (sethophormid)

Undescribed

Records: 34

This species has a small, round cephalis, and a thorax with increasingly large pores nearing the base.

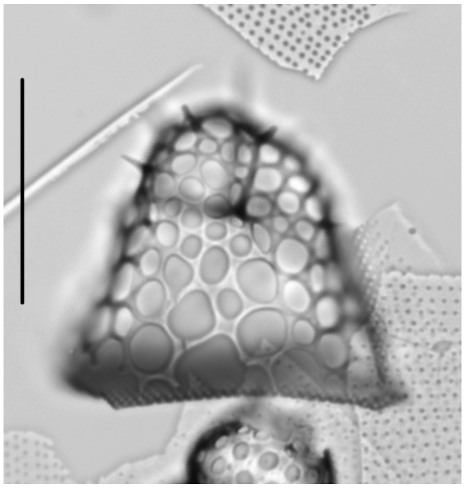

unknown plagonid group E sp 12

Plagiacanthidae (sethophormid)

Undescribed

Records: 7

This species is distinct for its wide, lobed cephalis, with many small spines. Irregular pores on thorax.

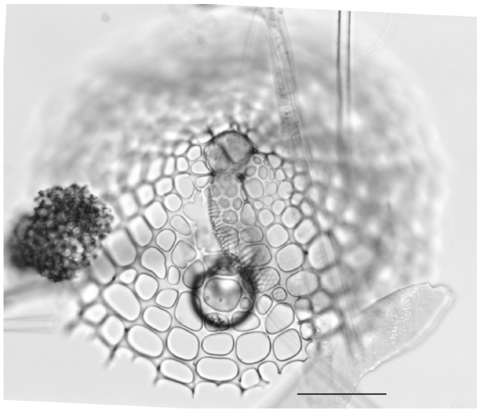

unknown plagonid group E sp 13

Plagiacanthidae (sethophormid)

Undescribed

Records: 107

This species is distinct for its long thorax with rectangular pores.

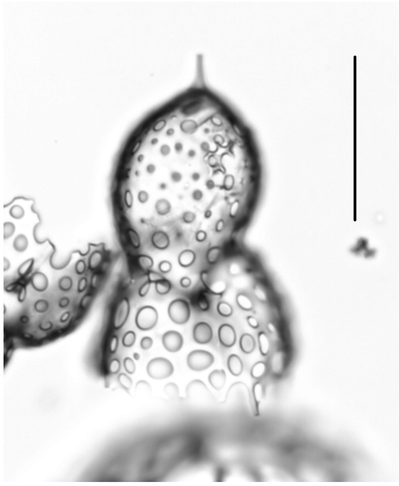

unknown plagonid group E sp 14

Plagiacanthidae (sethophormid)

Undescribed

Records: 72

This species has a long, narrow cephalis with a slightly pointed top, and a thin, conical spine at its apex. No apical spine is visible on the cephalis interior. Cephalis tends to be more lightly silicified than thorax.

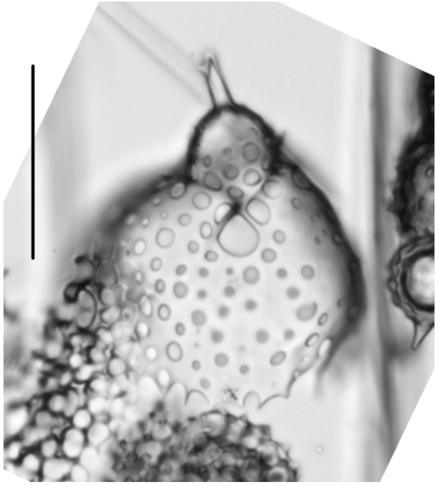

unknown plagonid group E sp 15

Plagiacanthidae (sethophormid)

Undescribed

Records: 201

This species has a small cephalis with a single large conical spine. Thorax has distinctive shoulders, and tend to be hyaline, and are separated by a single teardrop-shaped pore near the connection between cephalis and thorax.

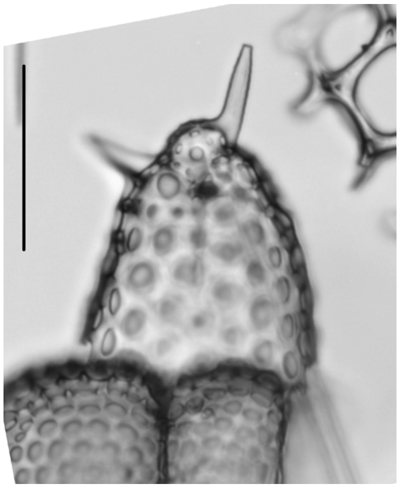

unknown plagonid group E sp 16

Plagiacanthidae (sethophormid)

Undescribed

Records: 10

This species resembles Ceratocyrtis sp 6, but differs in that it has two large conical spines at 90 degrees from each other protruding from the top and bottom of the small cephalis.

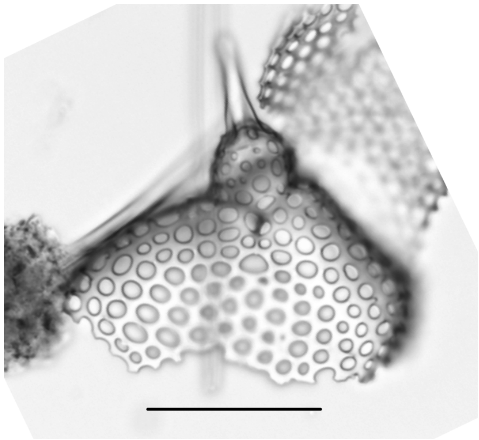

unknown plagonid group E sp 17

Plagiacanthidae (sethophormid)

Undescribed

Records: 8

This species is distinct for its very broad thorax.

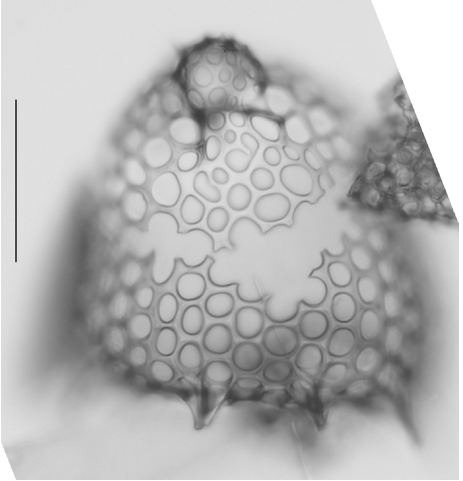

unknown plagonid group E sp 18

Plagiacanthidae (sethophormid)

Undescribed

Records: 15

This species resembles those in the Ceratocyrtis genus, but differs from all other observed species in that it has short, strong, and bladed spines at the base of the thorax

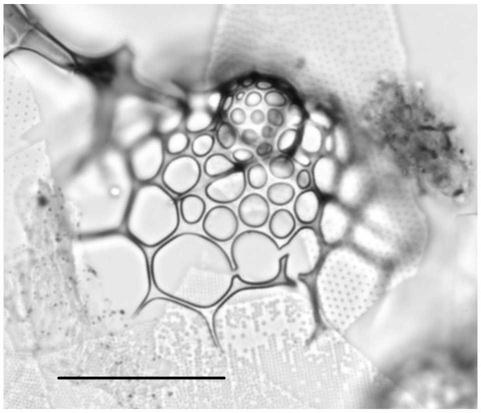

unknown plagonid group E sp 3

Plagiacanthidae (sethophormid)

Undescribed

Records: 38

This species is distinct for its thoracic pores that more than double in size from top to base. It has three ribs on thorax.

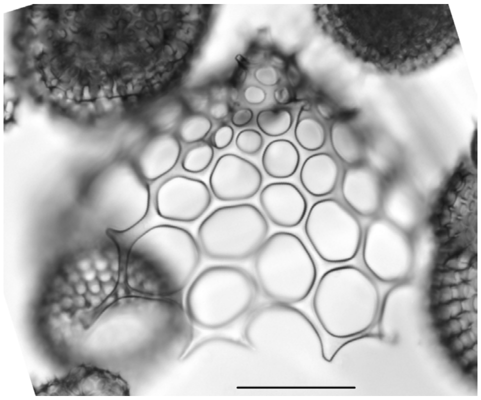

unknown plagonid group E sp 3 cf

Plagiacanthidae (sethophormid)

Undescribed

Records: 32

This species is like unknown plagonid group E sp 3, but is significantly larger and more robust.

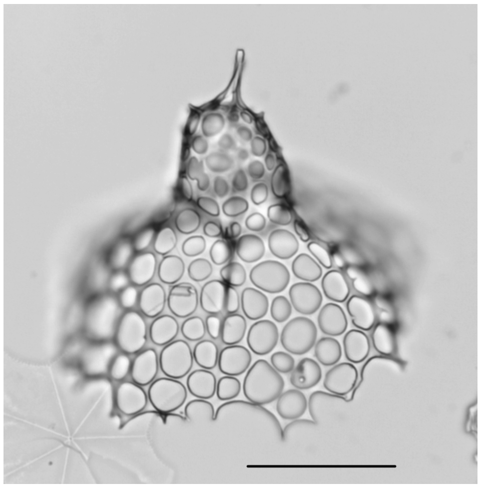

unknown plagonid group E sp 5

Plagiacanthidae (sethophormid)

Undescribed

Records: 46

This species has small, regular pores throughout cephalis and thorax, a thumb-shaped cephalis, and three ribs on thorax.

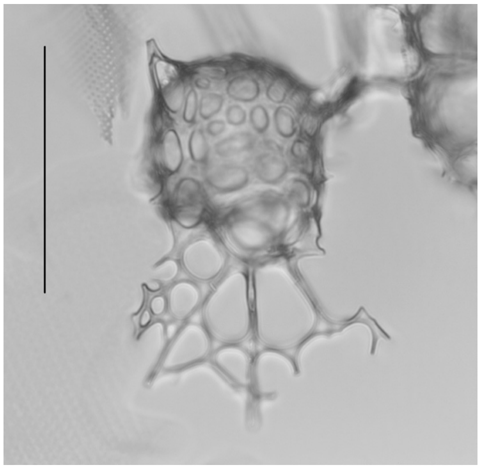

unknown plagonid group E sp 8

Plagiacanthidae (sethophormid)

Undescribed

Records: 19

This species has a cephalis with large horns, and a lightly-silicified thorax with large, angular pores.

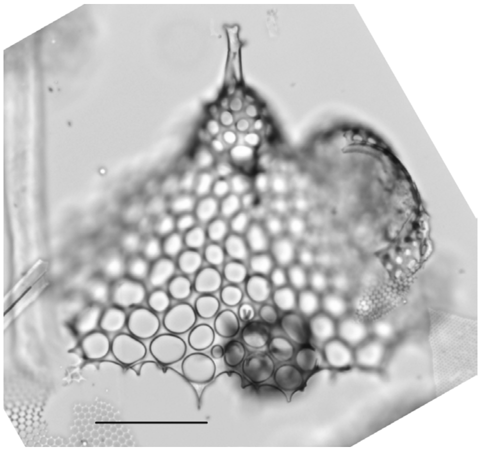

unknown plagonid group E sp 9

Plagiacanthidae (sethophormid)

Undescribed

Records: 39

This species is similar to unknown plagonid group E sp 5, except that it has a thorny spine on top of the cephalis, and tends to have a few enlarged pores near the base of the cephalis.

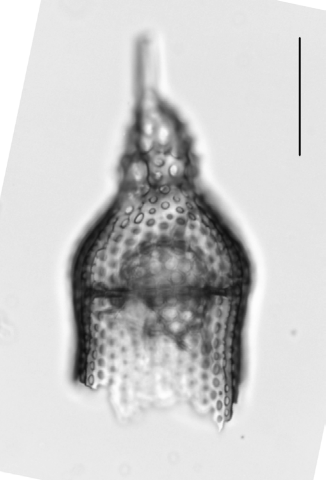

Albatrossidium minzok

Pterocorythidae

Sanfilippo & Riedel, 1992

Records: 6

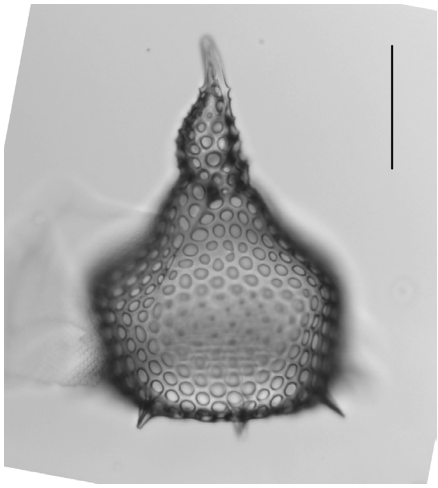

Anthocyrtidium angulare
Pterocorythidae
Nigrini, 1971
Records: 41

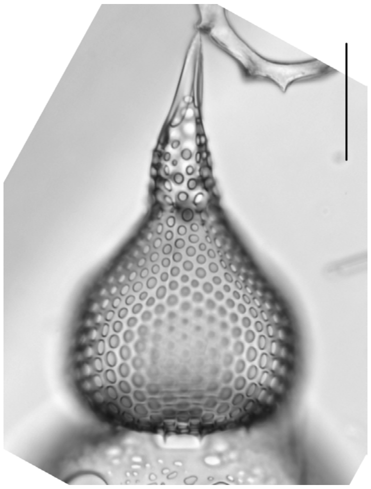

Anthocyrtidium ehrenbergii
Pterocorythidae
(Stohr) Haeckel, 1887
Records: 121

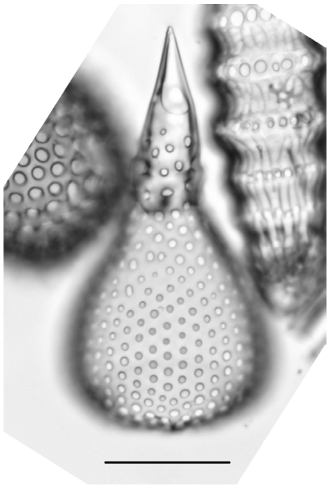

Anthocyrtidium euryclathrum
Pterocorythidae
Nigrini & Caulet, 1988
Records: 39

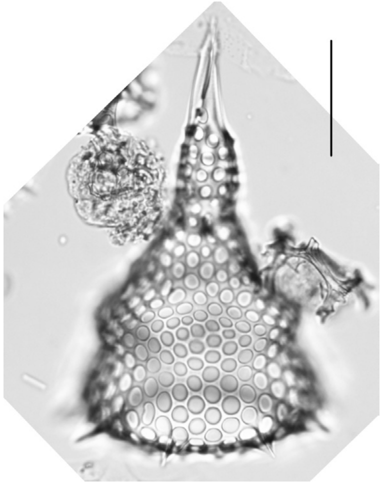

Anthocyrtidium jenghisi
Pterocorythidae
Streeter, 1988
Records: 3

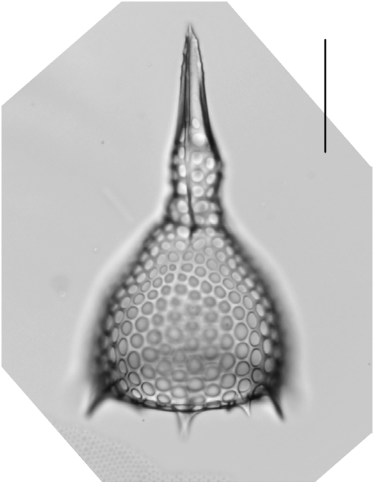

Anthocyrtidium ophirense
Pterocorythidae
(Ehrenberg) Petrushevskaya, 1968
Records: 138

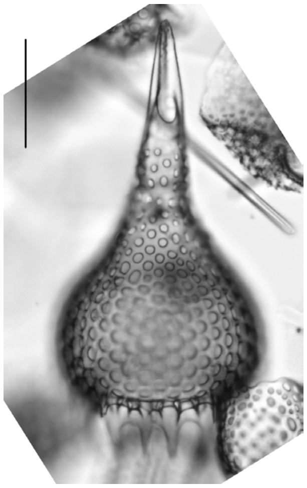

Anthocyrtidium pliocenica
Pterocorythidae
(Seguenza) Nigrini & Caulet, 1988
Records: 106

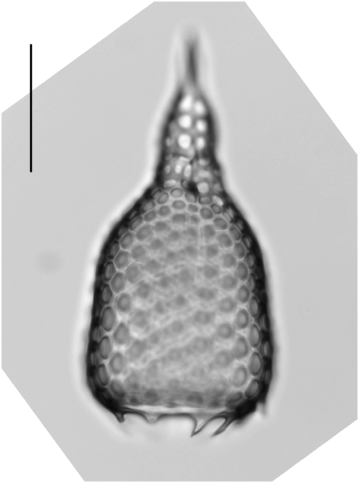

Anthocyrtidium pliocenica cf

Pterocorythidae

Undescribed

Records: 41

This species has a long, almost quadrate thorax, but does not have a well-developed set of double teeth. Horn on cephalis is also less pronounced than A. pliocenica.

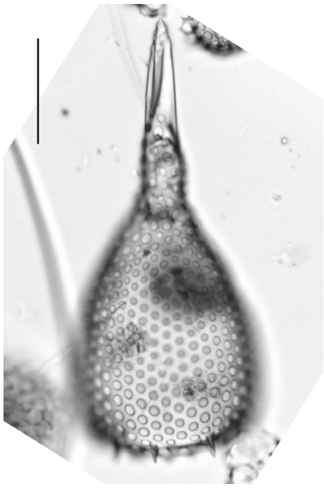

Anthocyrtidium prolatum

Pterocorythidae

Nigrini & Caulet, 1988

Records: 36

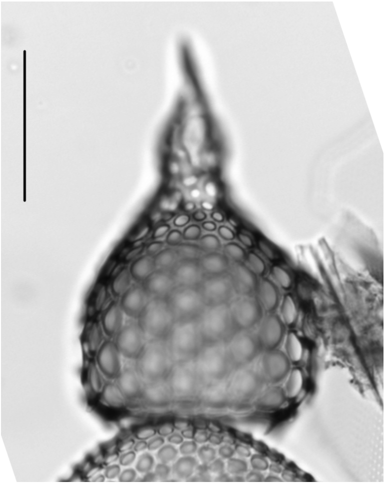

Anthocyrtidium sp 1

Pterocorythidae

Undescribed

Records: 13

This species is small, with a small horn on cephalis. Pores on thorax are distinctively large.

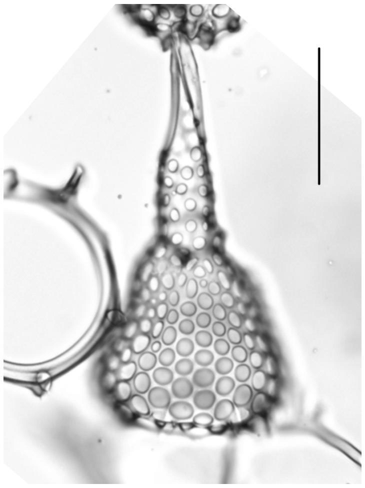

Anthocyrtidium sp 2

Pterocorythidae

Undescribed

Records: 17

This species has a small thorax, disproportionate to the size of cephalis, compared to other Anthocyrtidium species. Pores on thorax are smaller than those of Anthocyrtidium sp 1.

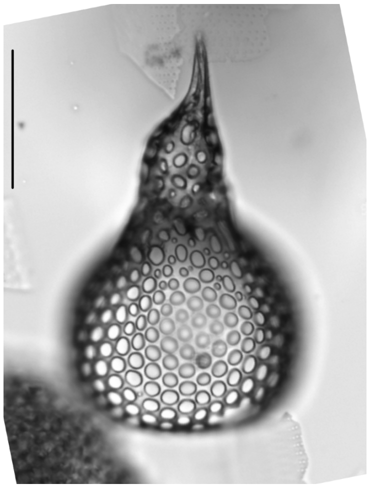

Anthocyrtidium zanguebaricum

Pterocorythidae

(Ehrenberg) Haeckel, 1887

Records: 144

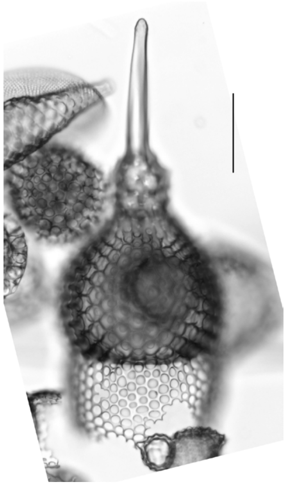

Calocycletta caepa

Pterocorythidae

Moore, 1972

Records: 41

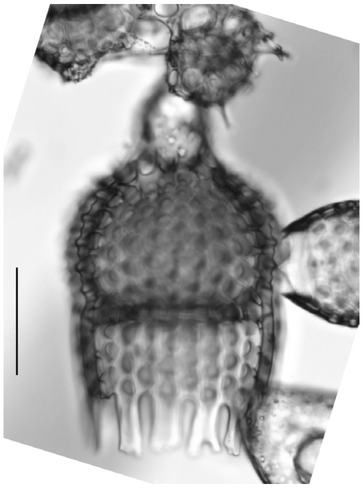

Calocycletta robusta

Pterocorythidae

Moore, 1971

Records: 28

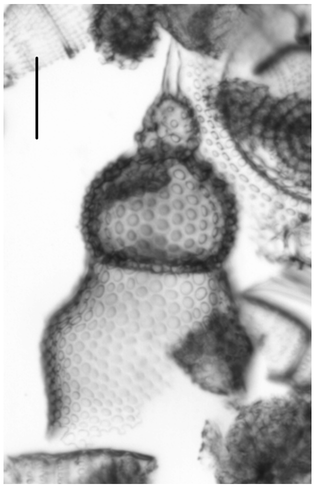

Calocycletta sp 1

Pterocorythidae

Undescribed

Records: 12

This species is distinct for its large, tent-like abdomen, which is widest midway between top and bottom.

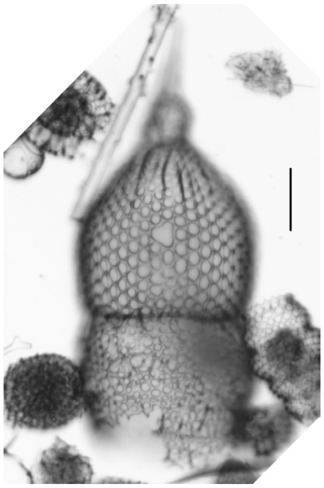

Calocycletta sp 2

Pterocorythidae

Undescribed

Records: 15

This species has distinct ridges on its large, pentagonal thorax. Abdomen is similar in width to thorax, but pores are significantly smaller and more irregular. This species is very large. Only one specimen has been observed.

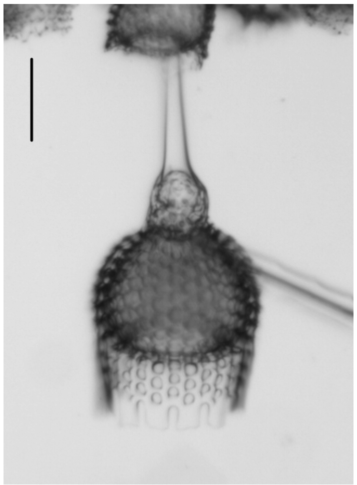

Calocycletta virginis

Pterocorythidae

Haeckel, 1887

Records: 16

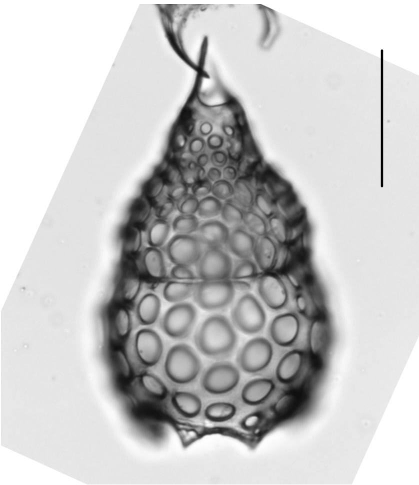

Lamprocyclas hannai

Pterocorythidae

(Campbell & Clark) Sanfilippo et al., 1985

Records: 149

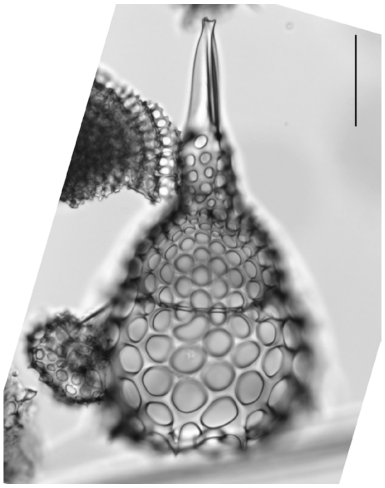

Lamprocyclas hannai cf

Pterocorythidae

Undescribed

Records: 14

Like L. hannai, except that horn on cephalis is much larger, and abdomen is relatively larger as well. Cephalis is also longer and more defined from thorax than it is in L hannai.

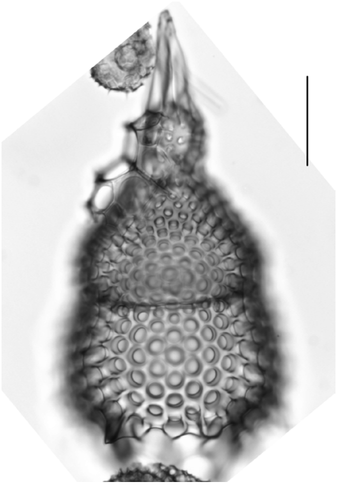

Lamprocyclas margatensis
Pterocorythidae
(Campbell & Clark) Caulet, 1986
Records: 77

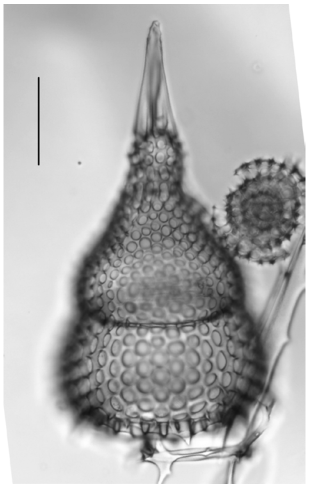

Lamprocyclas maritalis
Pterocorythidae
Haeckel, 1887
Records: 122

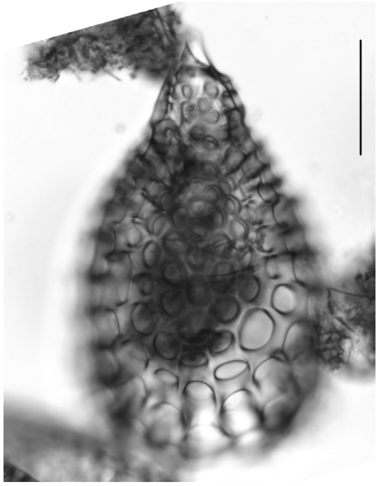

Lamprocyrtis daniellae
Pterocorythidae
Caulet, 1986
Records: 9

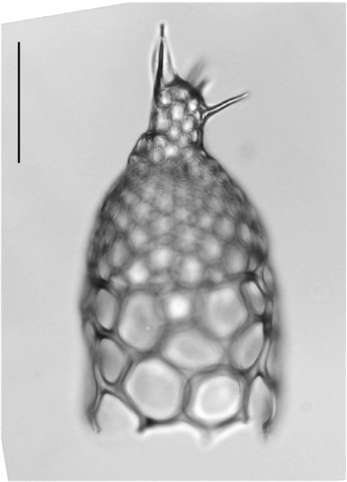

Lamprocyrtis heteroporos
Pterocorythidae
(Hays) Kling, 1973
Records: 37

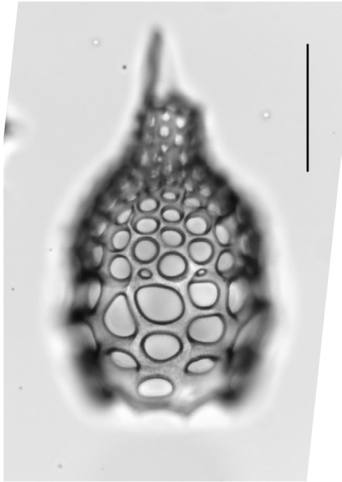

Lamprocyrtis neoheteroporos
Pterocorythidae
Kling, 1973
Records: 36

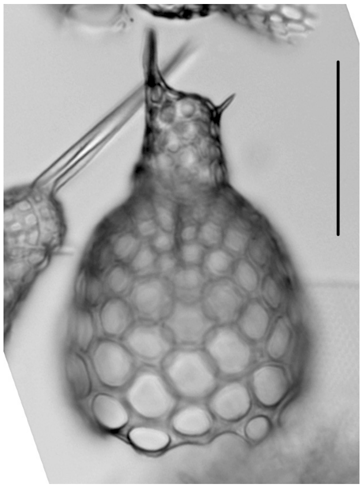

Lamprocyrtis nigriniae
Pterocorythidae
(Caulet) Kling, 1973
Records: 30

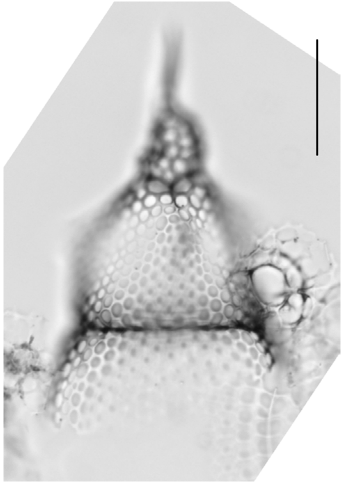

Pterocorys campanula
Pterocorythidae
Haeckel, 1887
Records: 66

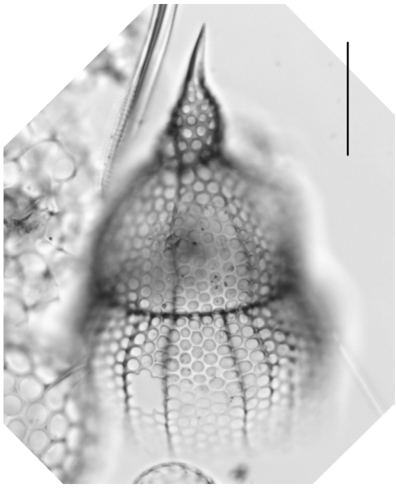

Pterocorys hertwigii
Pterocorythidae
(Haeckel) Petrushevskaya, 1971
Records: 60

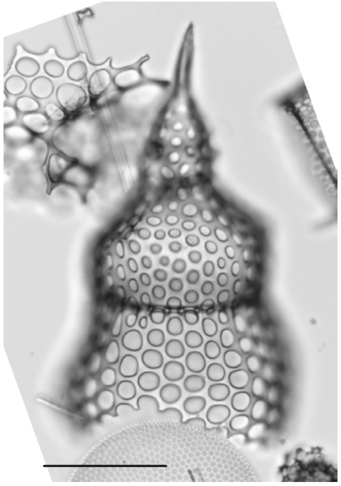

Pterocorys minythorax
Pterocorythidae
(Nigrini) Nigrini & Moore, 1979
Records: 56

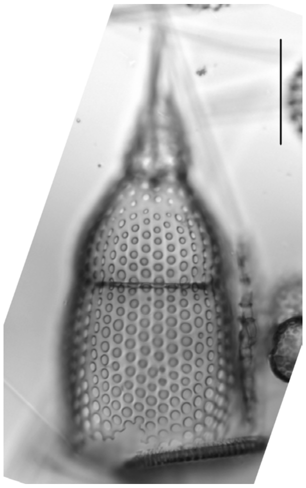

Pterocorys sp 1
Pterocorythidae
Undescribed
Records: 118

This species has small, circular pores arranged linear from thorax to base of abdomen. Widest point of shell tends to be the intersection of thorax and abdomen.

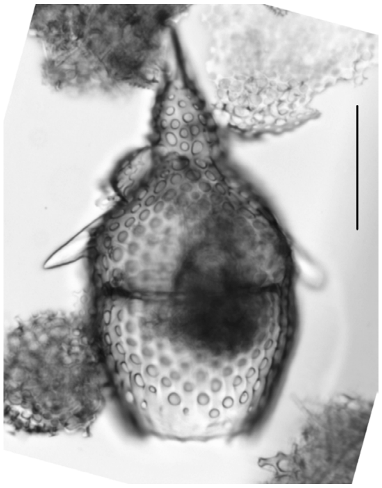

Pterocorys sp 2
Pterocorythidae
Undescribed
Records: 6

This species is distinct for its set of three, strong, conical wings pointing downward out of the thorax.

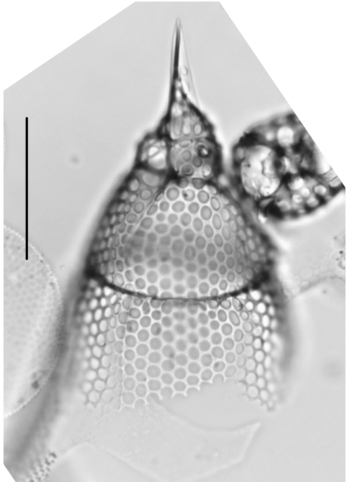

Pterocorys sp 3
Pterocorythidae
Undescribed
Records: 18

This species has a slightly campanulate thorax, but differs from P. campanula with its wide, short, and semi-trianglular cephalis, with a conical horn that rises gradually from the cephalis.

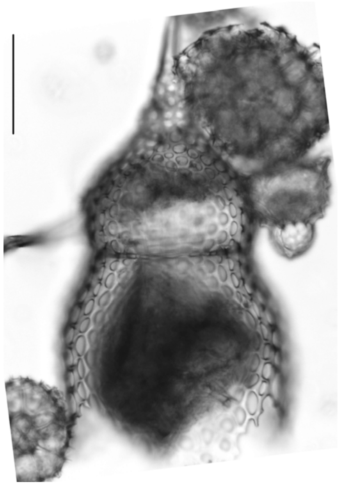

Pterocorys sp 4

Pterocorythidae

Undescribed

Records: 9

This species is large compared to the other Pterocorys species, with a long thorax, widest at its center point. Abdomen is also widest near its center point.

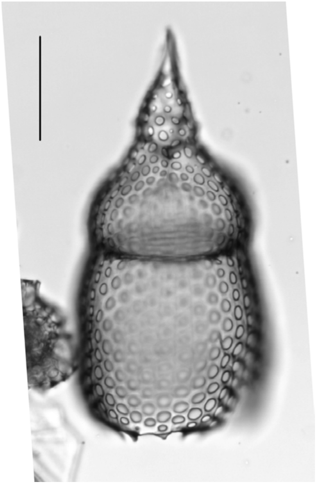

Pterocorys zancleus

Pterocorythidae

(Mueller) Nigrini & Moore, 1979

Records: 184

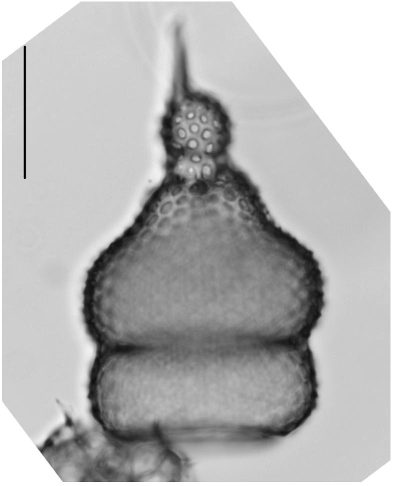

Theocorythium sp 1

Pterocorythidae

Undescribed

Records: 10

This species has a thorax similar to T. trachelium and T. vetulum, but the abdomen is significantly smaller than in either of those species.

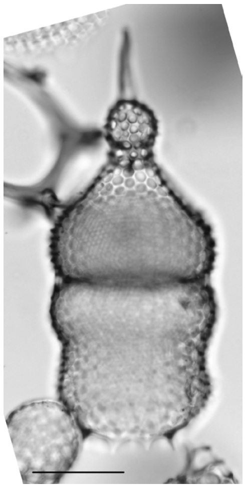

Theocorythium trachelium

Pterocorythidae

(Ehrenberg) Nigrini, 1967

Records: 38

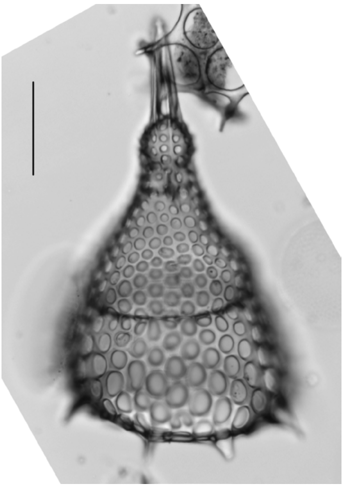

Theocorythium vetulum

Pterocorythidae

Nigrini, 1971

Records: 103

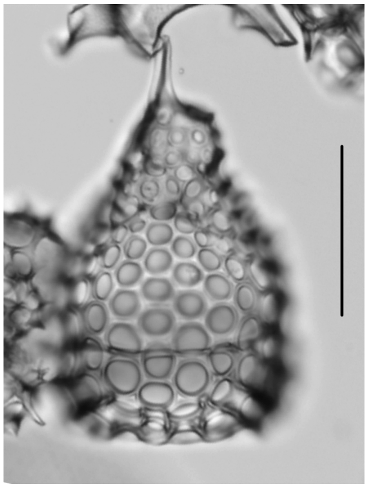

unknown lamprocyclid sp 1

Pterocorythidae

Undescribed

Records: 52

This species is very small, with a short abdomen.

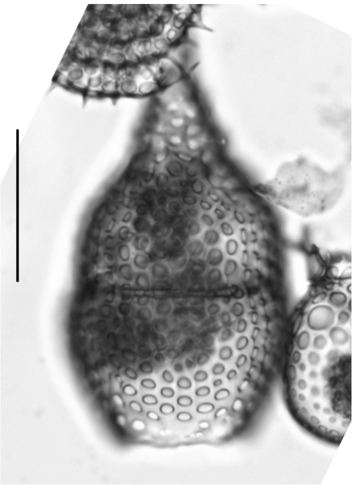

unknown lamprocyclid sp 2

Pterocorythidae

Undescribed

Records: 11

This species has a similar overall shape to L. hannai, but its pores are significantly smaller and it is more lightly silicified.

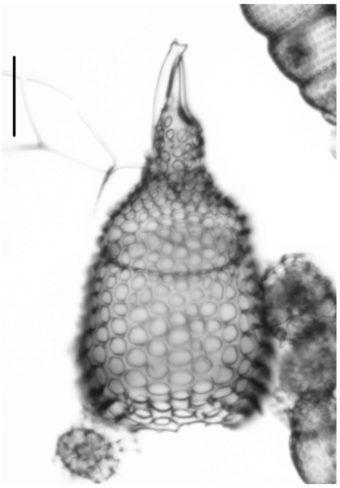

unknown lamprocyclid sp 3

Pterocorythidae

Undescribed

Records: 28

This species is similar to L. hannai cf, except that its thorax is longer.

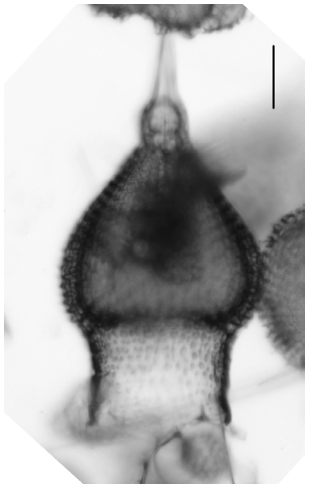

unknown pterocorythid sp 1

Pterocorythidae

Undescribed

Records: 92

This species is distinct for its large size, with strongly framed pores on its pear-shaped thorax, and very finely latticed abdomen.

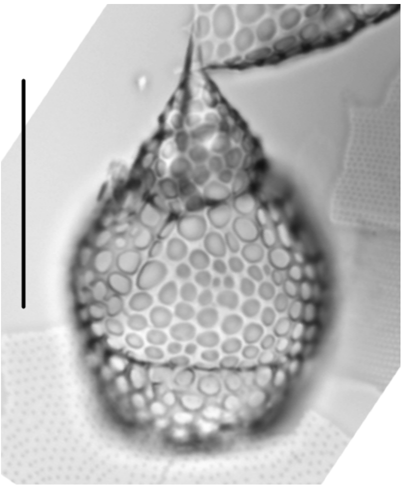

unknown pterocorythid sp 2

Pterocorythidae

Undescribed

Records: 13

This species has a similar size and shape to unknown lamprocyclid sp 1, but differs in that it is more lightly silicified, has smaller pores on thorax and abdomen, and the cephalis is structured completely differently. It resembles the genus Pterocorys rather than Lamprocyclas.

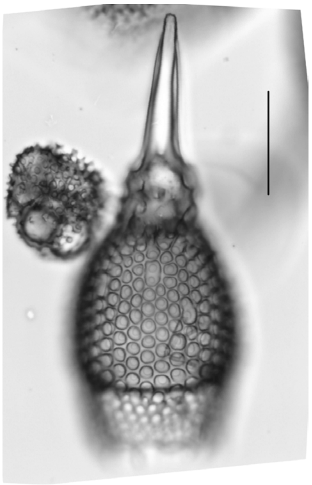

unknown pterocorythid sp 3

Pterocorythidae

Undescribed

Records: 17

This species has a strong conical horn on cephalis, relatively long and narrow cephalis, and a short abdomen with muted teeth.

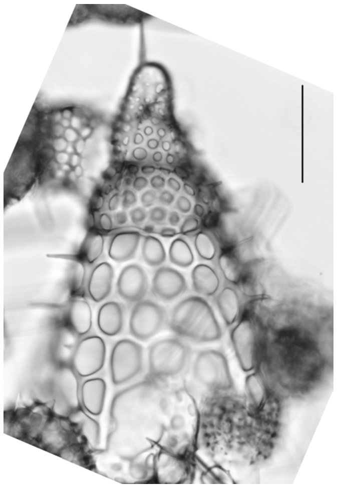

Anthocyrtella callospima

Theoperidae

Caulet, 1986

Records: 48

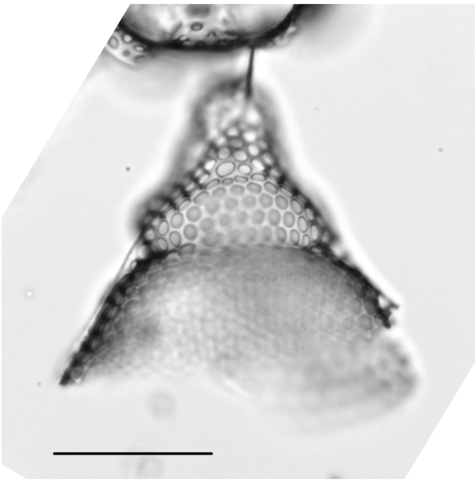

Anthocyrtella callospima cf

Theoperidae

Undescribed

Records: 19

This species has a similar shape to A. callopisma, but differs in that it is smaller and has much smaller pores.

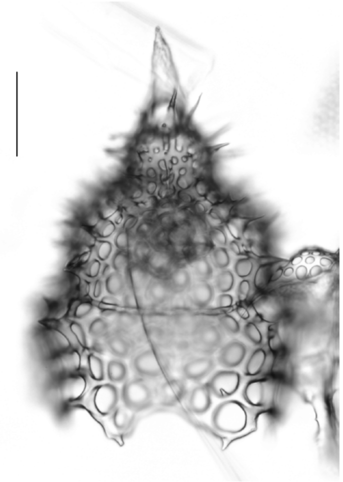

Calocyclas monumentum

Theoperidae

Haeckel, 1887

Records: 69

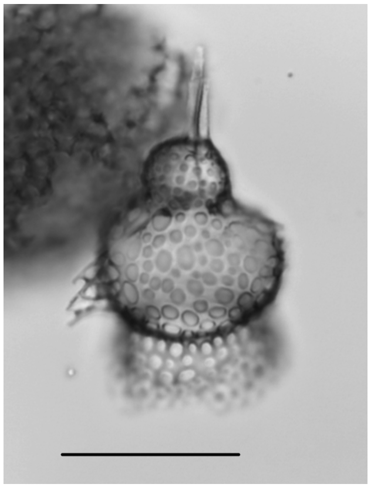

Clathrocanium insectum

Theoperidae

(Haeckel) Takahashi, 1991

Records: 55

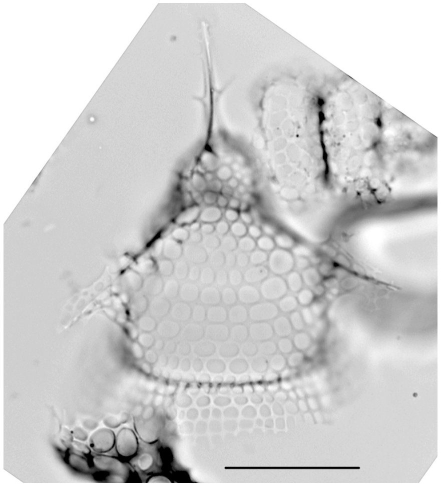

Clathrocanium insectum cf

Theoperidae

Undescribed

Records: 13

This species is different from C. insectum in that it has a campanulate thorax, rather than round, as well as rectangular pores. It also has a more decorative/thorny apical spine. This species is possibly synonymous with Pteropilium stratiotes (Haeckel 1887).

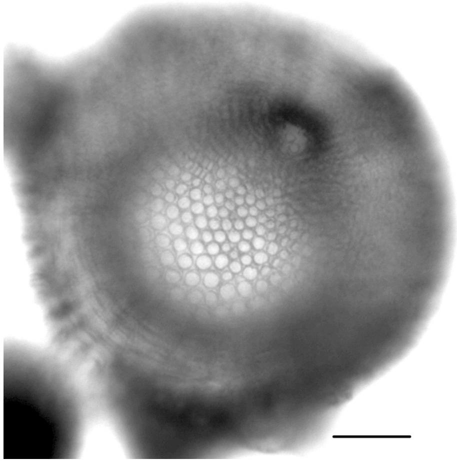

Cyclampterium brachythorax

Theoperidae

Sanfilippo & Riedel, 1970

Records: 15

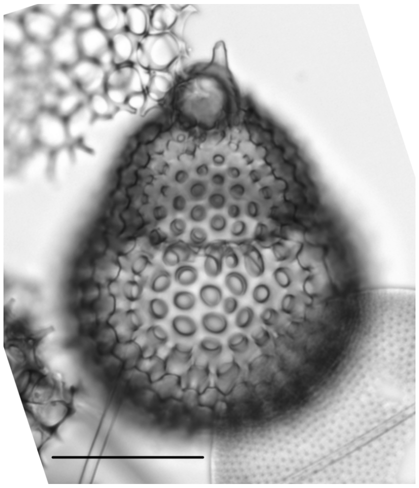

Cyrtocapsella japonica

Theoperidae

Tochilina, 1985

Records: 69

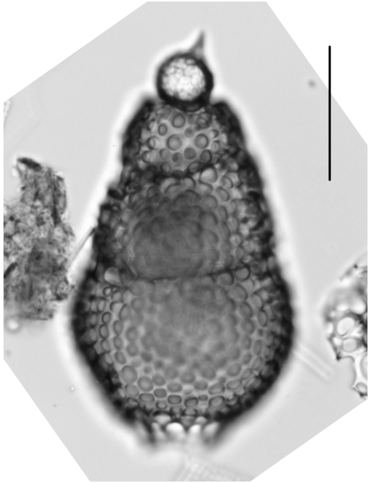

Cyrtocapsella sp 1
Theoperidae
Undescribed
Records: 54

This species has three segments, plus a small fourth segment/pylome on occasion. It is narrower than Cyrtocapsella tetrapera, and has more segments than C. japonica.

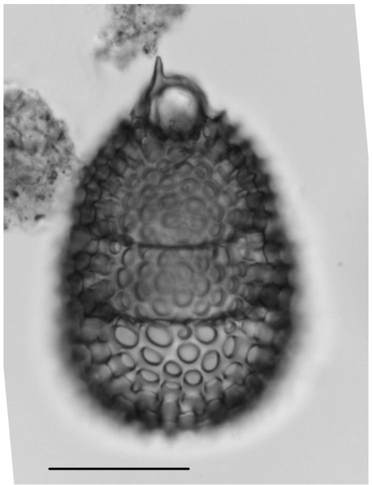

Cyrtocapsella tetrapera
Theoperidae
Haeckel, 1887
Records: 32

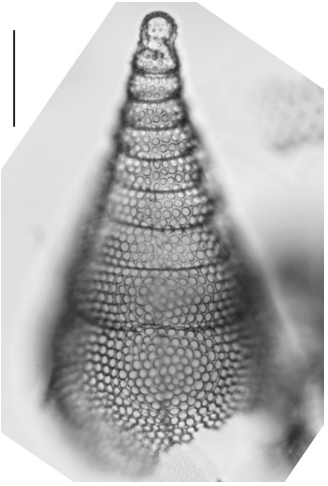

Cyrtopera aglaolampa
Theoperidae
Takahashi, 1991
Records: 14

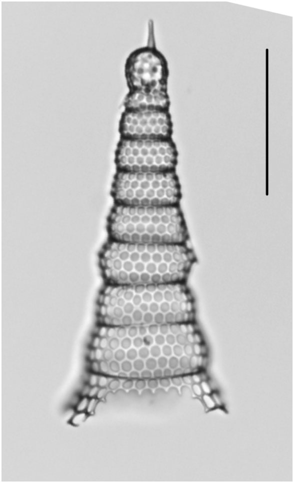

Cyrtopera languncula
Theoperidae
Haeckel, 1887
Records: 15

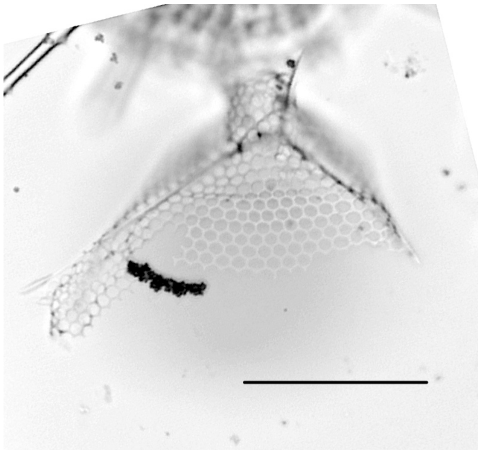

Dictyocodon palladius
Theoperidae
Haeckel, 1887
Records: 6

sensu Takahashi 1991, Pl. 37

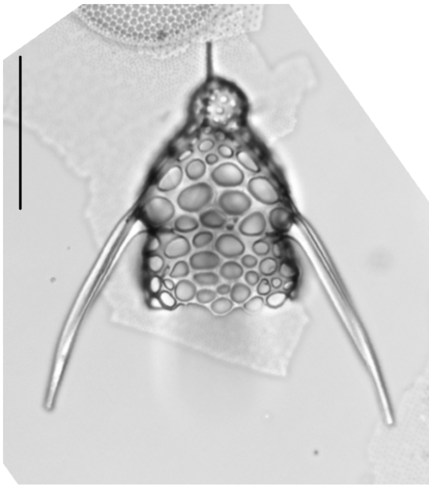

Dictyophimus crisiae
Theoperidae
Ehrenberg, 1854
Records: 96

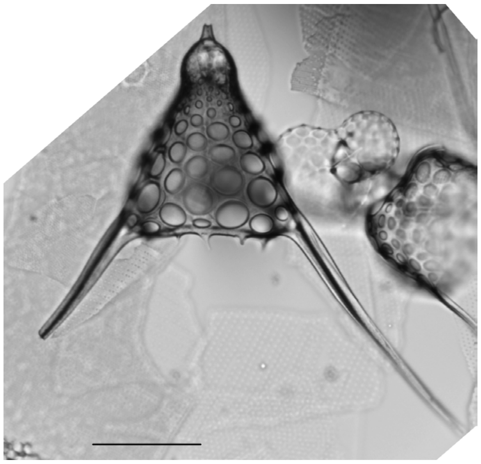

Dictyophimus hirundo
Theoperidae
(Haeckel) Petrushevskaya, 1975
Records: 46

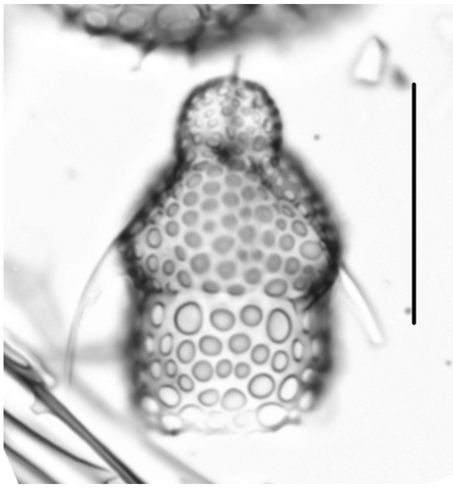

Dictyophimus killmari
Theoperidae
Renz, 1974
Records: 83

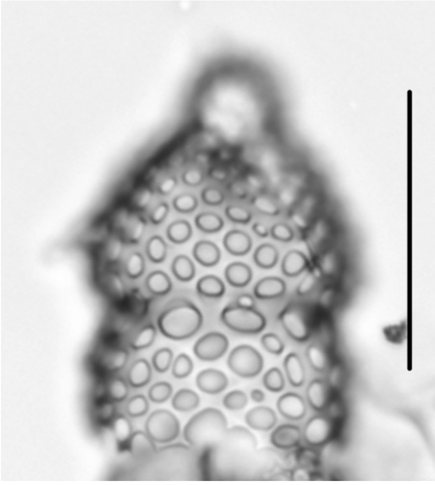

Dictyophimus killmari cf
Theoperidae
Undescribed
Records: 38

This species is similar to D. killmari, except that its thorax is significantly larger and wider. Differs from T. veneris in that it has three downward-pointing spines, a more triangular-shaped thorax, and larger on the thorax.

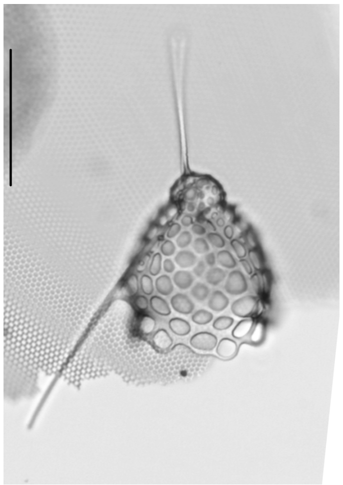

Dictyophimus killmari cf sp 2
Theoperidae
Undescribed
Records: 84

This species is similar to D. killmari, but has only a thorax, with no suture mark indicating an abdomen.

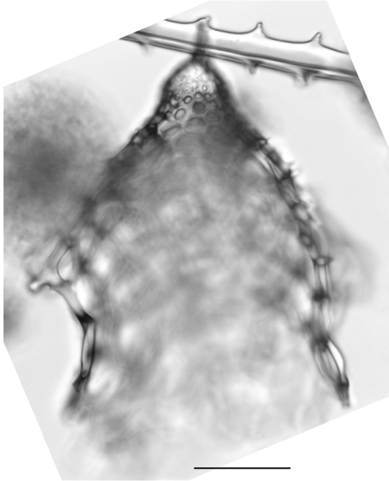

Dictyophimus kiwi
Theoperidae
Renaudie & Lazarus, 2012
Records: 17

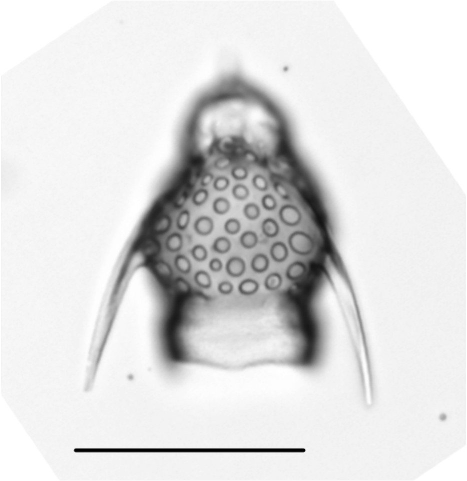

Dictyophimus macropterus
Theoperidae
(Ehrenberg) Takahashi, 1991
Records: 68

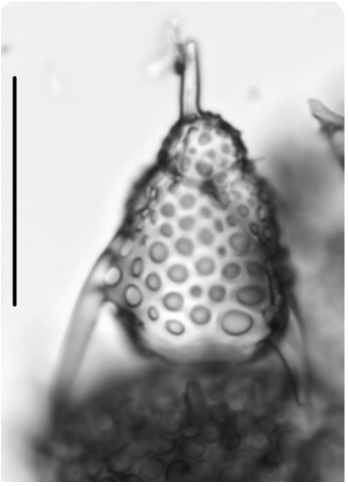

Dictyophimus sp 1
Theoperidae
Undescribed
Records: 25

This species resembles D. macropterus in size and general shape, but differs in that the base of the thorax is not as constricted.

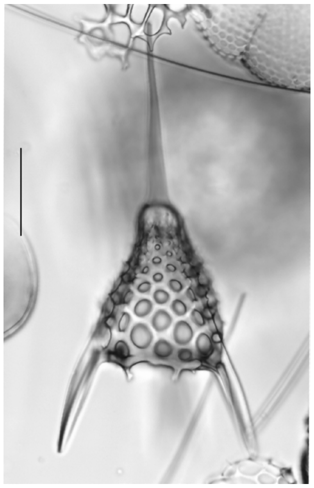

Dictyophimus splendens
Theoperidae
Clark, 1942
Records: 91

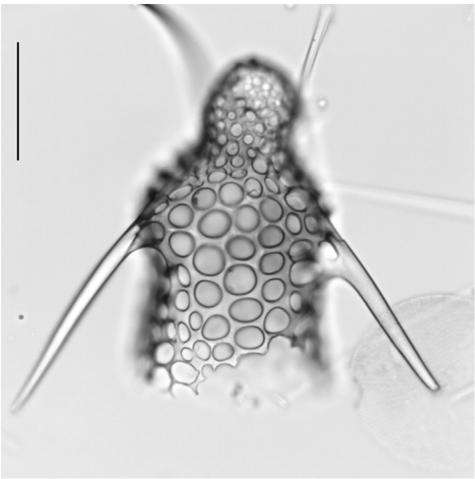

Dictyophimus? sp 2
Theoperidae
Undescribed
Records: 11

The shape of this species resembles D. crisiae, but differs in that it has a distinctly large, irregular cephalis, and conical feet rather than bladed ones.

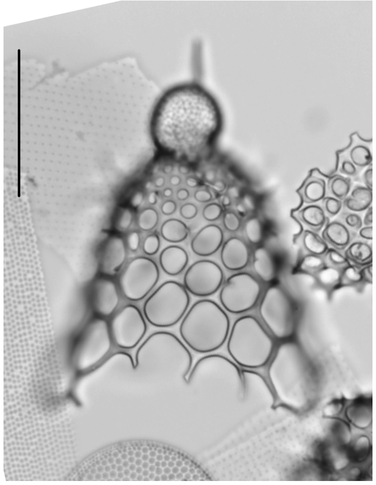

Gondwanaria campanulaeformis
Theoperidae
Campbell & Clark, 1945
Records: 51

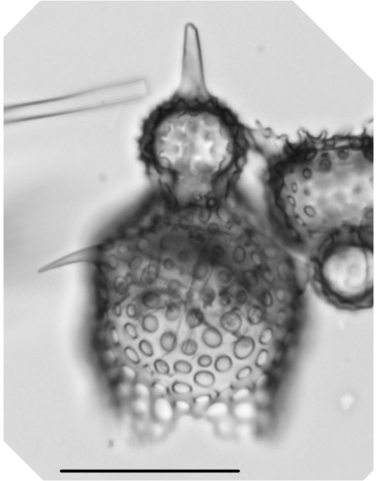

Gondwanaria cylindrica cf
Theoperidae
Undescribed
Records: 7

This species is similar to G. cylindrica (Funakawa 2000), but lacks the lower spine on the cephalis, and has a more narrow neck.

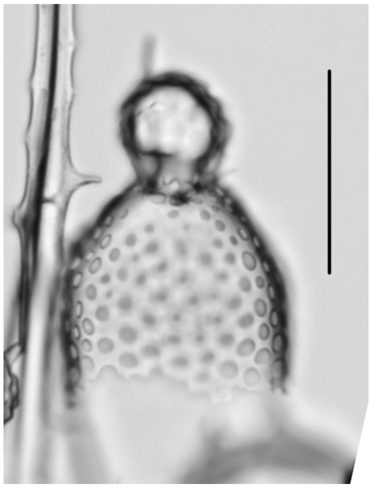

Gondwanaria hister
Theoperidae
Petrushevskaya, 1975
Records: 7

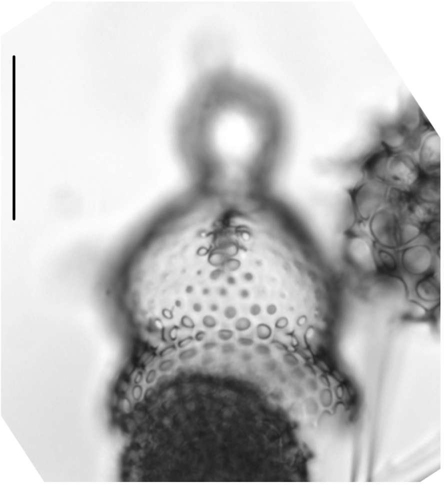

Gondwanaria sp 1
Theoperidae
Undescribed
Records: 6

This species is distinct for its very small pores on the thorax, which disappear into hyaline area at the midpoints between wings.

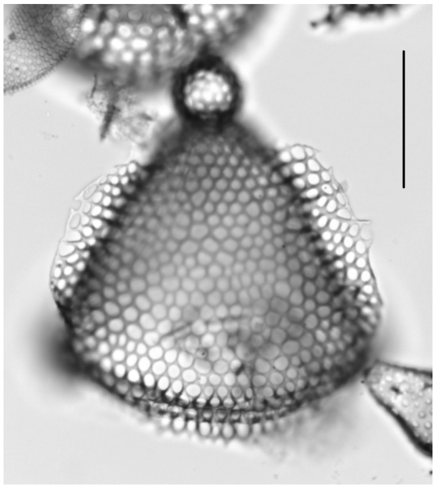

Lipmanella bombus
Theoperidae
Haeckel, 1887
Records: 19

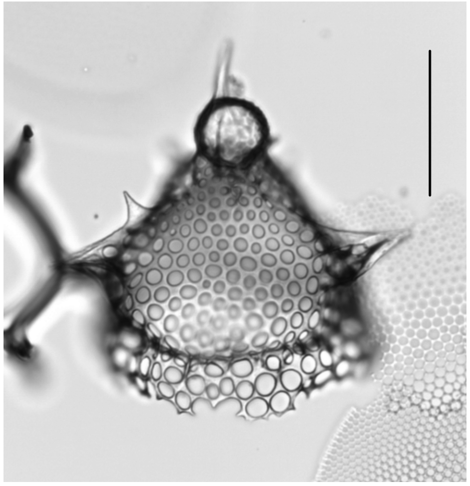

Lipmanella dictyoceras
Theoperidae
(Haeckel) Kling, 1973
Records: 43

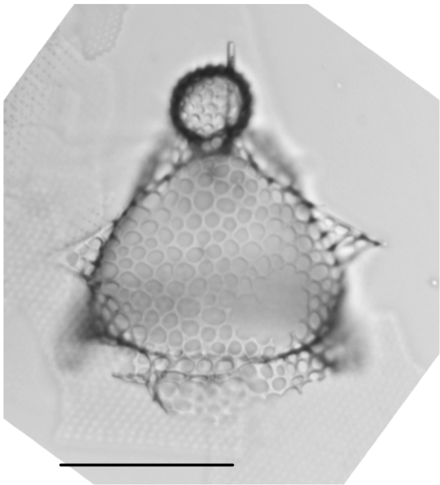

Lipmanella xiphephorum
Theoperidae
Jørgensen, 1899
Records: 26

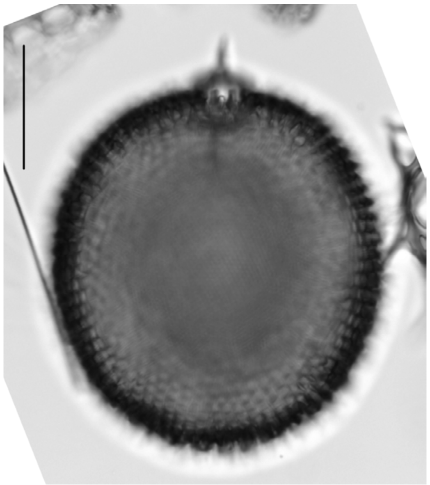

Lithopera bacca
Theoperidae
Ehrenberg, 1872
Records: 36

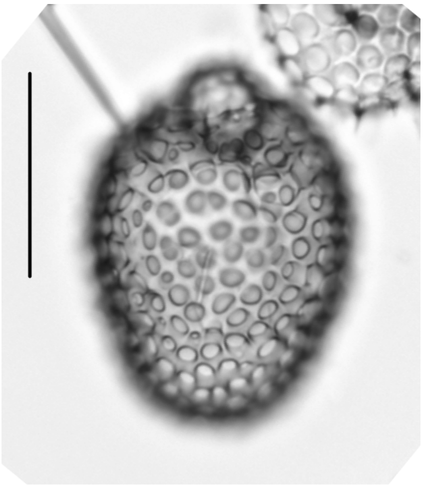

Lithopera neotera
Theoperidae
Sanfilippo, 1970
Records: 31

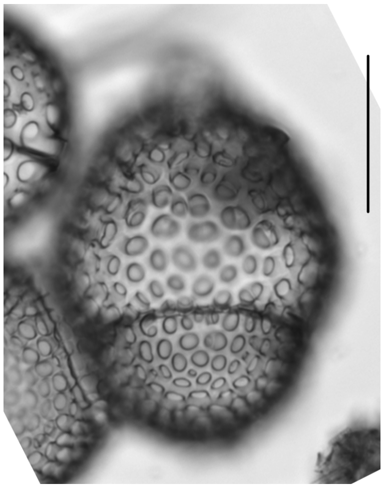

Lithopera renzae
Theoperidae
Sanfilippo, 1970
Records: 23

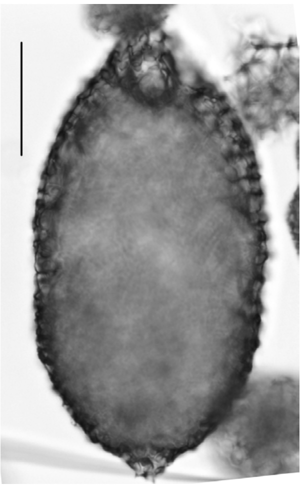

Lithopera thornburgi
Theoperidae
Sanfilippo, 1970
Records: 36

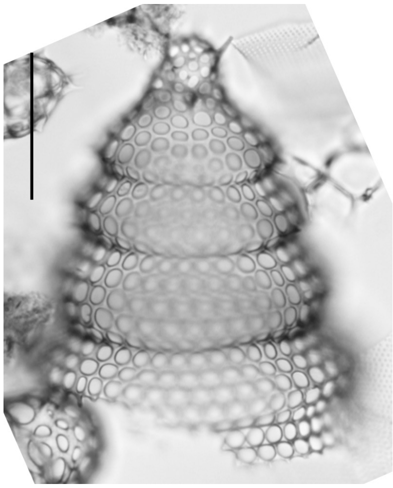

Lithostrobus hexagonalis
Theoperidae
Haeckel, 1887
Records: 19

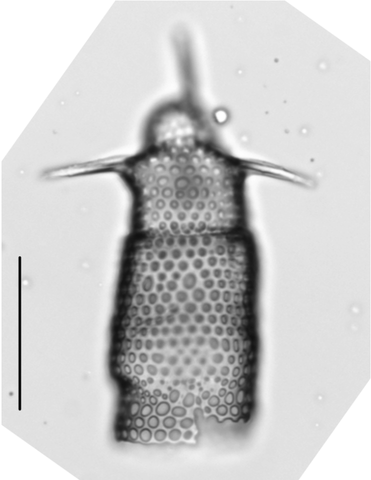

Stichopilidium kruegeri
Theoperidae
(Popofsky) Nishimura & Yamauchi, 1984
Records: 84

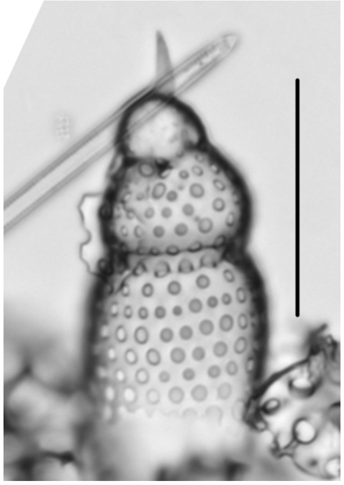

Stichopilidium kruegeri cf
Theoperidae
Undescribed
Records: 24

This species is like S. kruegeri except that it lacks wings on it thorax.

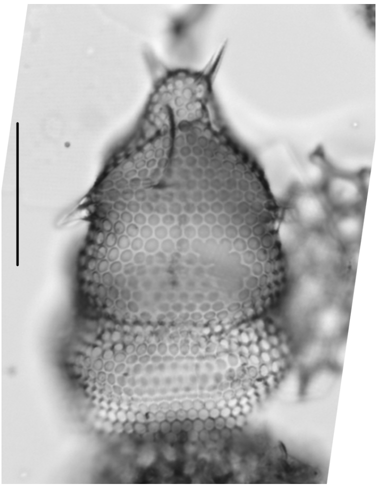

Stichopilium bicorne
Theoperidae
Haeckel, 1887
Records: 102

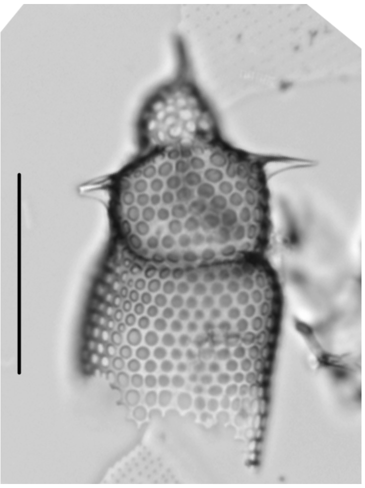

Stichopilium bicorne cf

Theoperidae

Undescribed

Records: 49

This species has some structural similarities to S. bicorne, but it only has one cephalic spine instead of two, and is overall smaller with a more round cephalis. It differs from S. kruegeri in that its thorax is substantially wider.

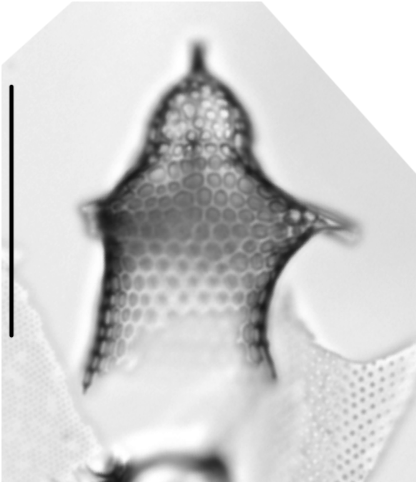

Stichopilium bicorne cf sp 2

Theoperidae

Undescribed

Records: 18

This species is similar in shape to S. bicorne cf, except that there is no abdomen, or no clear separation between thorax and abdomen.

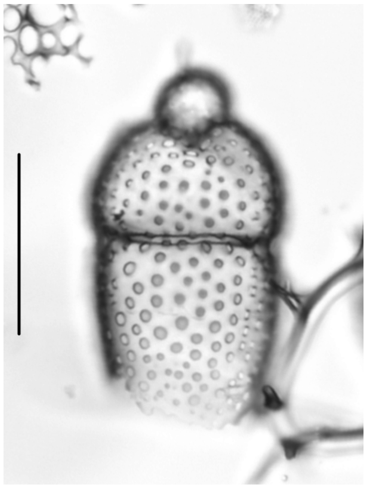

Theocorys veneris

Theoperidae

Haeckel, 1887

Records: 104

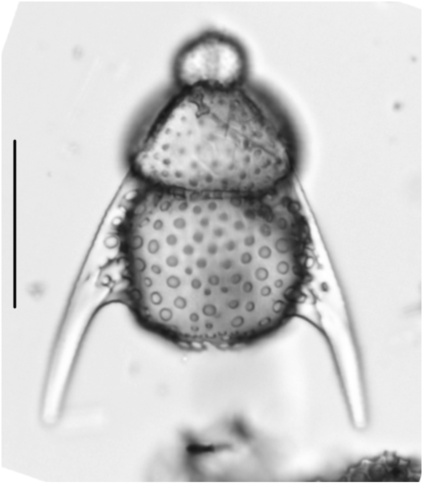

Theocorys veneris cf

Theoperidae

Undescribed

Records: 40

This species has a similar overall shape to T. veneris, but differs in its distinct feet/spines (three) that run from the base of the thorax to middle of abdomen, and then separate and point outward from the shell. These feet often have pores.

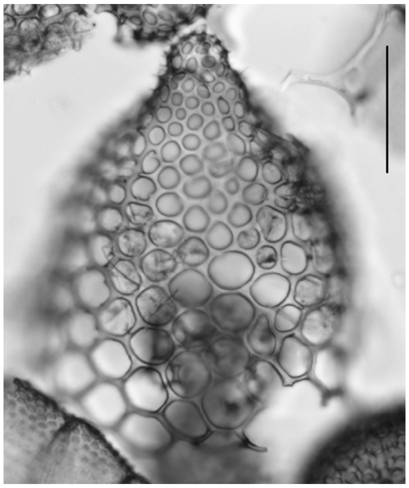

unknown theoperid sp 10

Theoperidae

Undescribed

Records: 18

This species is very long, with only one segment. Pores gradually increase in size from base of cephalis to the bottom of the thorax.

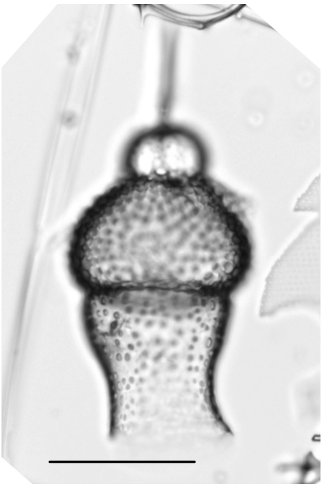

unknown theoperid sp 7

Theoperidae

Undescribed

Records: 46

This species has a rounded thorax, with very small pores, and a thin, veil-like abdomen that constricts in toward its base. Thorax occasionally has short wings preserved.

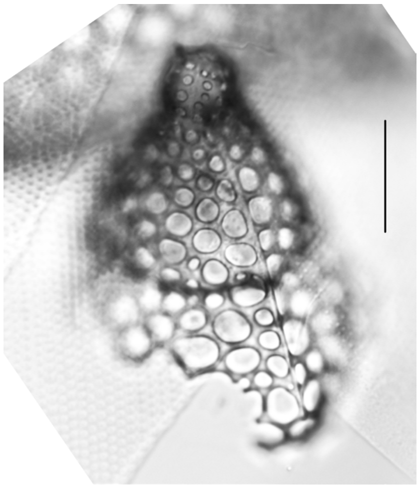

Valkyria pukapuka
Theoperidae
O'Connor, 1997
Records: 15

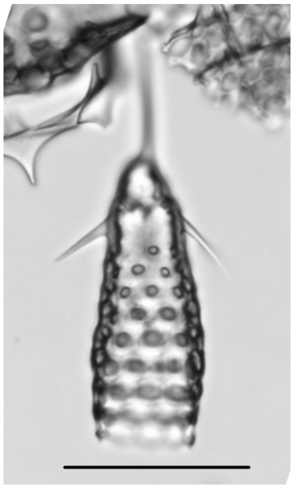

Artostrobus annulatus
Theoperidae (cornutellid)
(Bailey) Haeckel, 1887
Records: 38

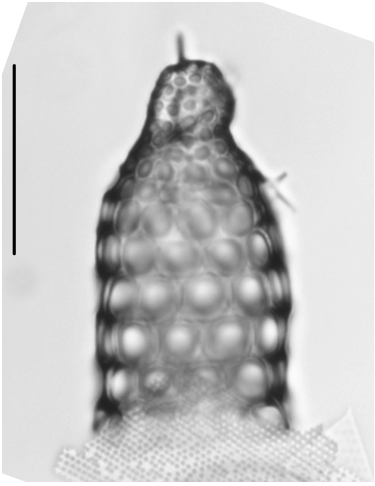

Artostrobus joergenseni
Theoperidae (cornutellid)
Petrushevskaya, 1967
Records: 39

sensu Kamikuri, 2017

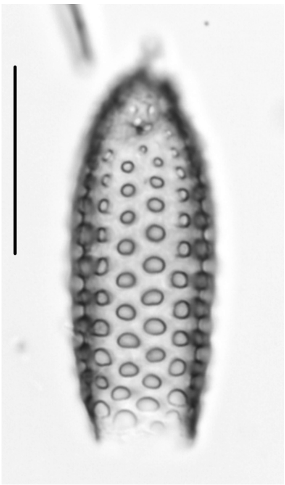

Artostrobus pretabulatus
Theoperidae (cornutellid)
Petrushevskaya, 1975
Records: 23

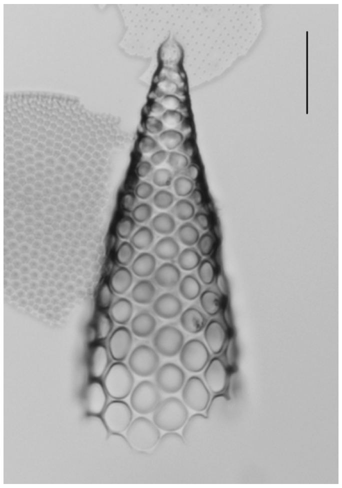

Cornutella profunda
Theoperidae (cornutellid)
Ehrenberg, 1856
Records: 30

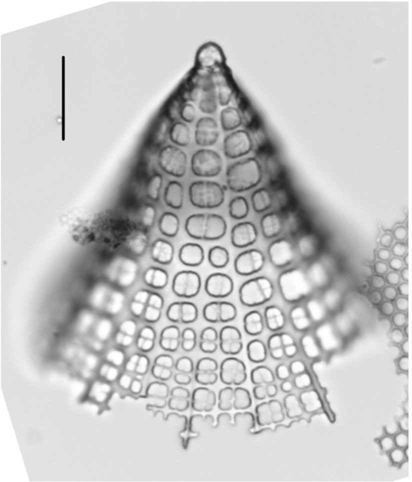

Litharachnium tentorium
Theoperidae (cornutellid)
Haeckel, 1860
Records: 39

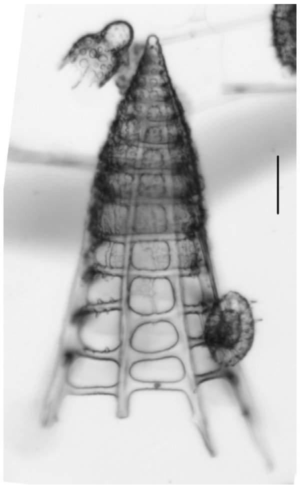

Peripyramis circumtexta

Theoperidae (cornutellid)

Haeckel, 1887

Records: 54

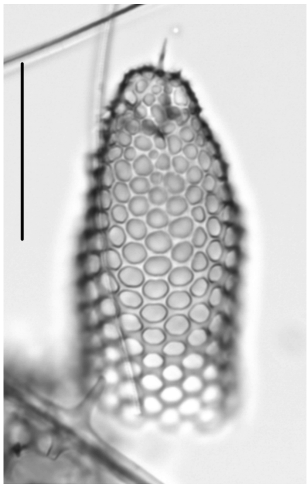

Sethoconus tabulatus

Theoperidae (cornutellid)

Haeckel, 1887

Records: 59

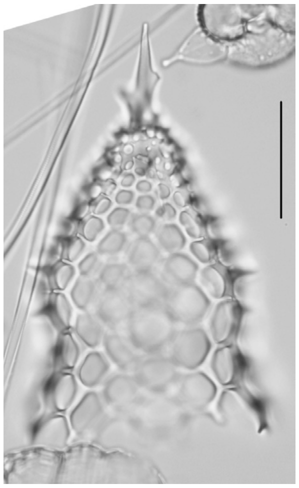

Sethoconus tabulatus cf

Theoperidae (cornutellid)

Undescribed

Records: 11

This species is similar to S. tabulatus, except that it has a more irregular outline, larger pores, and a thorny exterior.

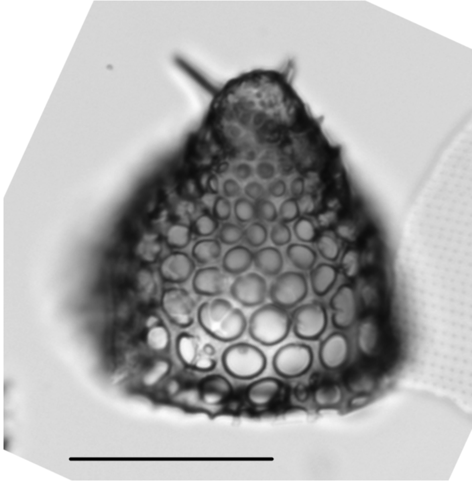

Cycladophora bicornis

Theoperidae (cycladophorid)

(Popofsky) Lombari & Lazarus, 1988

Records: 74

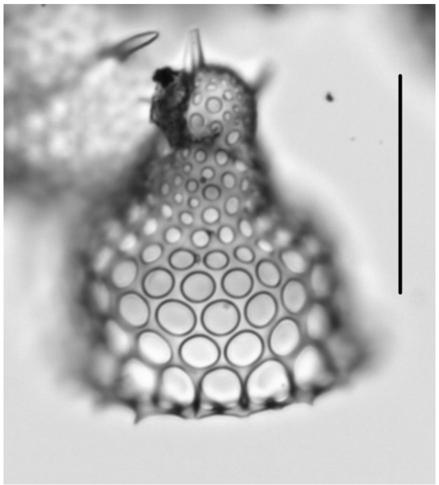

Cycladophora bicornis cf

Theoperidae (cycladophorid)

Undescribed

Records: 21

This species is similar to C. bicornis, except that the shape of the thorax is different; it has a distinct inflection point.

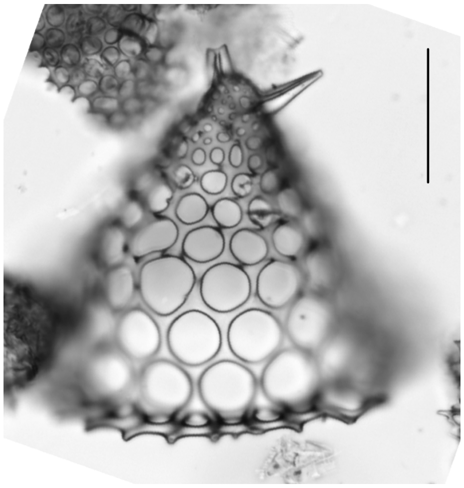

Cycladophora cabrilloensis

Theoperidae (cycladophorid)

(Campbell & Clark) Lombari & Lazarus, 1988

Records: 87

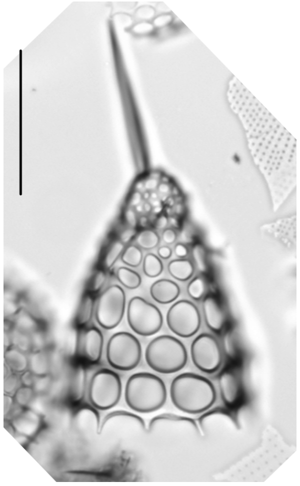

Cycladophora conica
Theoperidae (cycladophorid)
Lombari & Lazarus, 1988
Records: 60

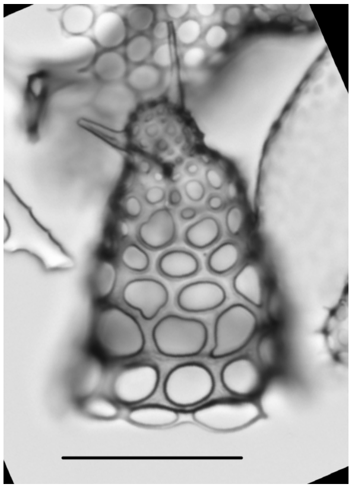

Cycladophora cornutoides
Theoperidae (cycladophorid)
Petrushevskaya, 1967
Records: 70

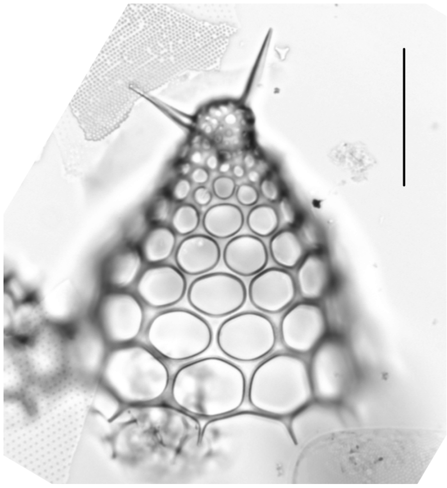

Cycladophora cosma
Theoperidae (cycladophorid)
Lombari & Lazarus, 1988
Records: 82

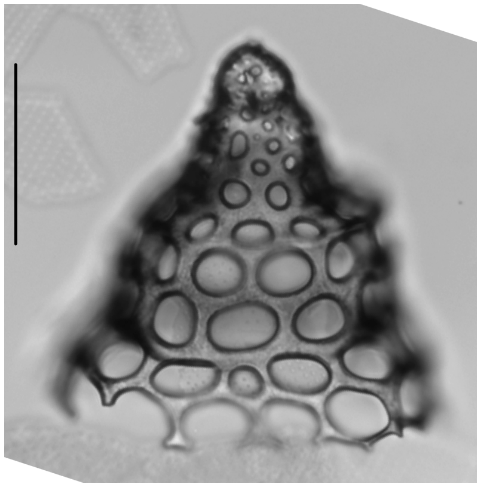

Cycladophora davisiana
Theoperidae (cycladophorid)
Ehrenberg, 1861
Records: 69

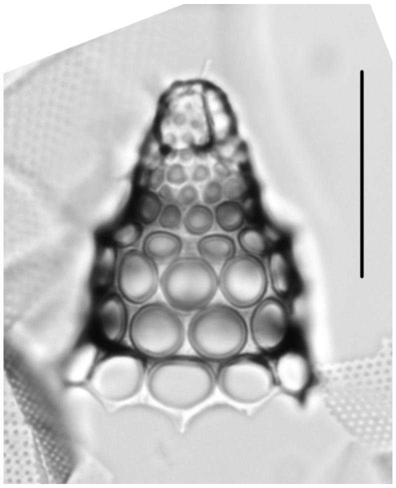

unknown Cycladophora sp 1
Theoperidae (cycladophorid)
Undescribed
Records: 36

This species has a similar angular structure to C. davisiana, but differs in that it is smaller and is built more symmetrically.

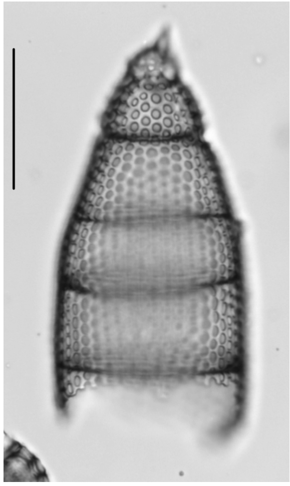

Eucyrtidium acuminatum
Theoperidae (eucyrtinid)
Ehrenberg, 1847
Records: 187

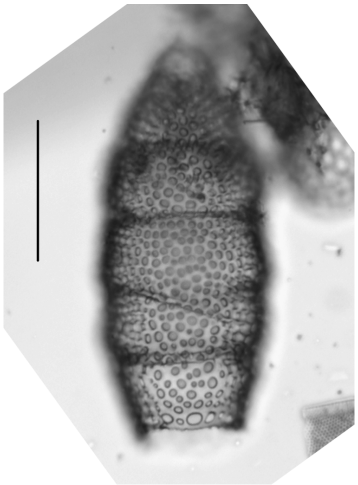

Eucyrtidium aderces

Theoperidae (eucyrtinid)

Nigrini & Caulet, 1992

Records: 25

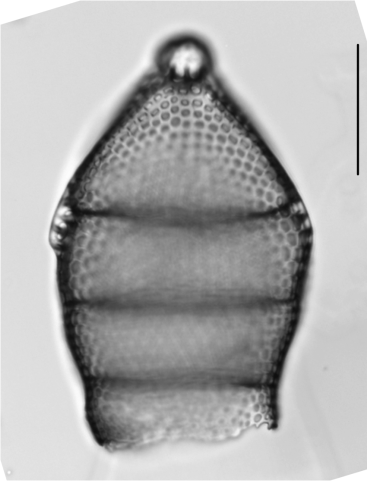

Eucyrtidium anomalum

Theoperidae (eucyrtinid)

Haeckel, 1862

Records: 61

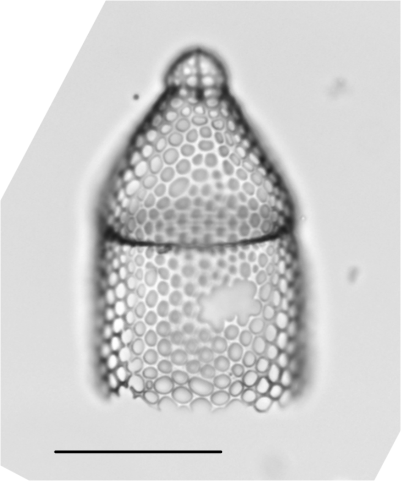

Eucyrtidium anomalum cf sp 1

Theoperidae (eucyrtinid)

Undescribed

Records: 29

This species has a longer abdomen than E. anomalum and E. anomalum cf sp 2. It is also distinct for having a slightly wider thorax base than abdomen.

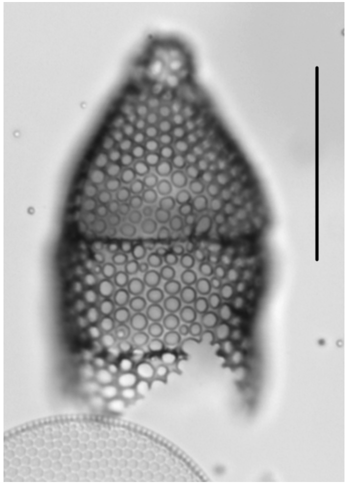

Eucyrtidium anomalum cf sp 2

Theoperidae (eucyrtinid)

Undescribed

Records: 62

The large thorax of this species is similar to E. anomalum, but overall this species is narrower in outline, and tends to have curved segments.

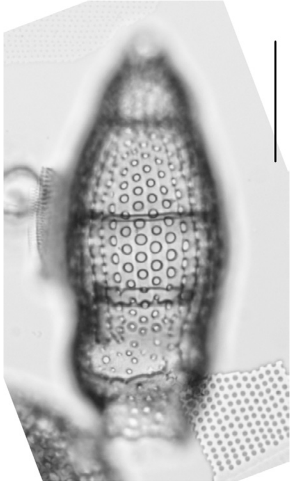

Eucyrtidium calvertense

Theoperidae (eucyrtinid)

Martin, 1904

Records: 18

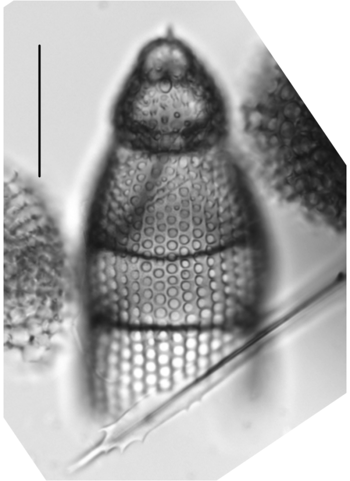

Eucyrtidium cienkowski

Theoperidae (eucyrtinid)

Haeckel, 1887

Records: 41

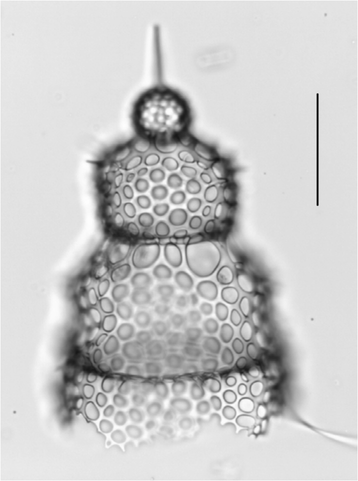

Eucyrtidium diaphanes
Theoperidae (eucyrtinid)
Sanfilippo, 1973
Records: 18
sensu Kamikuri, 2017 (Stichocorys diaphanes)

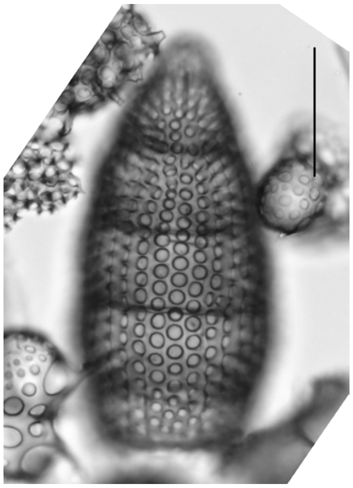

Eucyrtidium erythromystax
Theoperidae (eucyrtinid)
Nigrini & Caulet, 1992
Records: 22

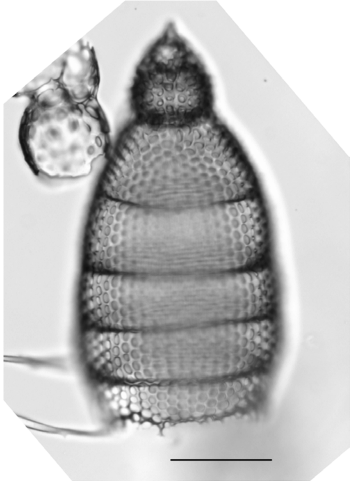

Eucyrtidium hexagonatum
Theoperidae (eucyrtinid)
Haeckel, 1887
Records: 131

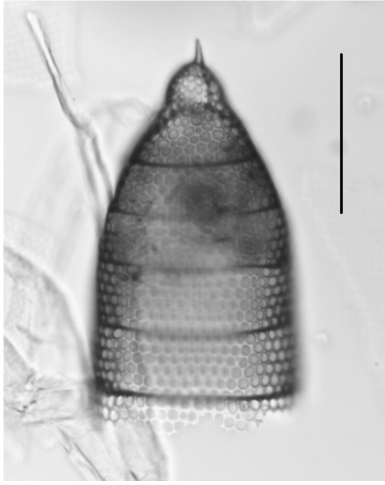

Eucyrtidium hexastichum
Theoperidae (eucyrtinid)
Haeckel, 1887
Records: 53

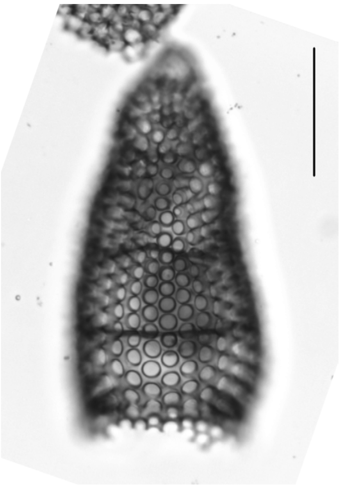

Eucyrtidium indiensis
Theoperidae (eucyrtinid)
Caulet, 1979
Records: 5

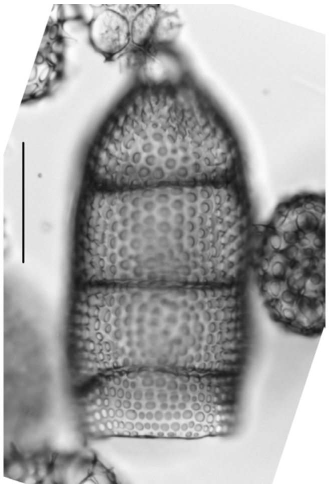

Eucyrtidium infundibulum
Theoperidae (eucyrtinid)
Haeckel, 1887
Records: 84
different from definition of Benson, 1983

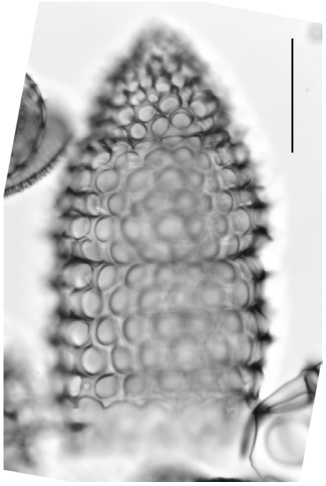

Eucyrtidium sp 1

Theoperidae (eucyrtinid)

Undescribed

Records: 6

This species has three segments, and unusually large pores for Eucyrtidium. Could be synonymous with E. funnellium

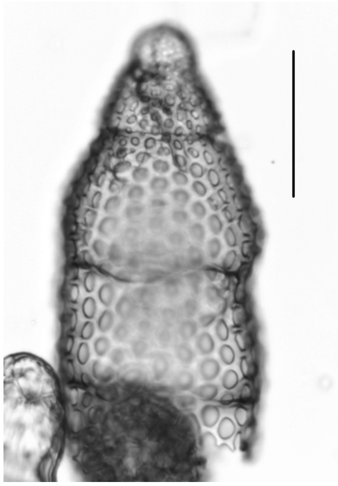

Eucyrtidium teuscheri

Theoperidae (eucyrtinid)

Haeckel, 1887

Records: 123

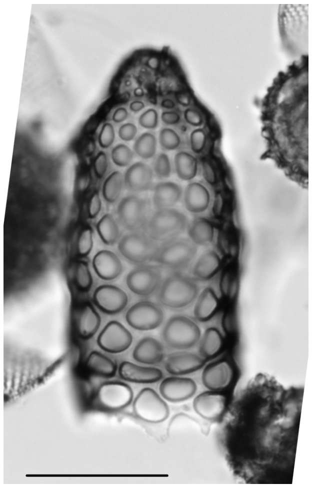

Eucyrtidium teuscheri cf

Theoperidae (eucyrtinid)

Undescribed

Records: 25

Similar shape and pore size to E. teuscheri, except that segments have no clear distinction with a suture.

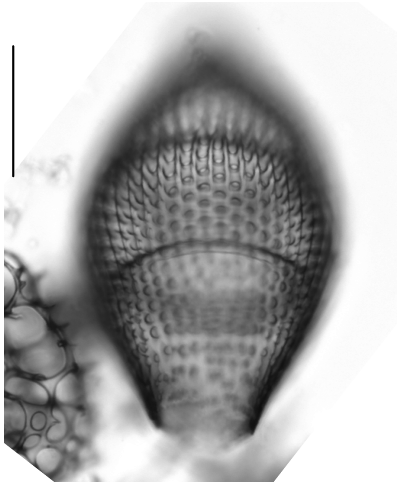

Eucyrtidium yatsuoense

Theoperidae (eucyrtinid)

Nakaseko, 1955

Records: 9

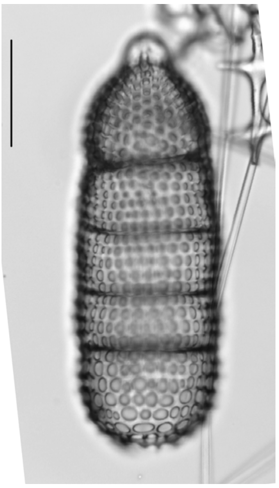

Lithocampe sp 1 Nigrini

Theoperidae (eucyrtinid)

Nigrini, 1967

Records: 48

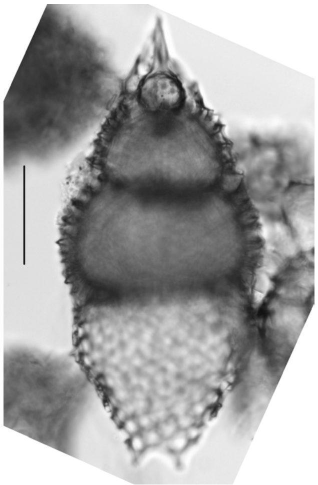

Stichocorys armata

Theoperidae (eucyrtinid)

(Haeckel) Riedel & Sanfilippo, 1971

Records: 8

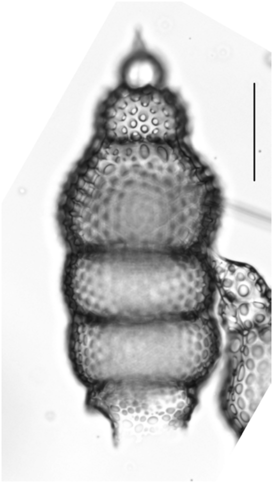

Stichocorys delmontensis

Theoperidae (eucyrtinid)

(Campbell & Clark) Sanfilippo & Riedel, 1970

Records: 118

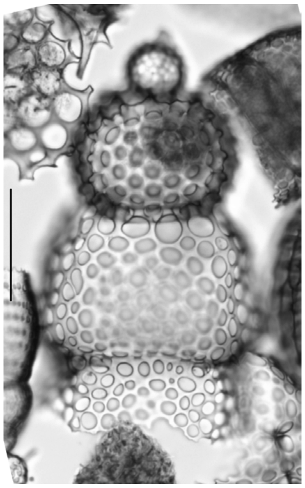

Stichocorys johnsoni

Theoperidae (eucyrtinid)

Caulet, 1986

Records: 12

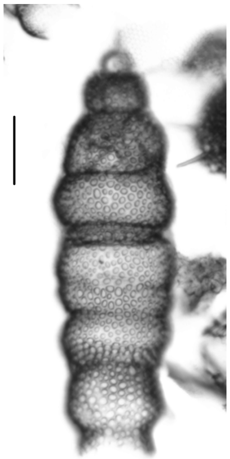

Stichocorys peregrina

Theoperidae (eucyrtinid)

Riedel, 1953

Records: 144

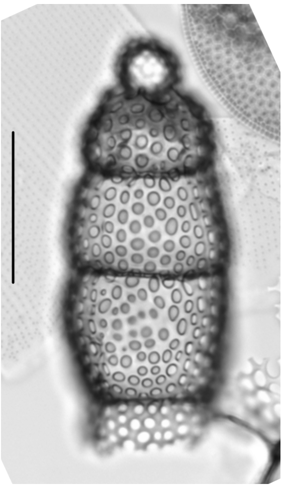

Stichocorys peregrina cf

Theoperidae (eucyrtinid)

Undescribed

Records: 103

This species achieves maximum width at the third or fourth segment, like S. peregrina. It differs in that the thorax is relatively longer and wider than S. peregrina, and its overall shape is smoother.

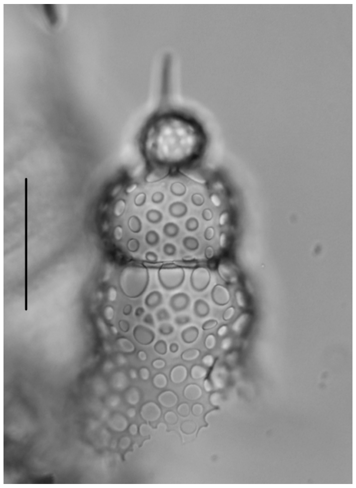

Stichocorys sp 1

Theoperidae (eucyrtinid)

Undescribed

Records: 24

This species has an extended abdomen, without post-abdominal segments. The first row of pores on both the thorax and the abdomen are elongated, as if stretched.

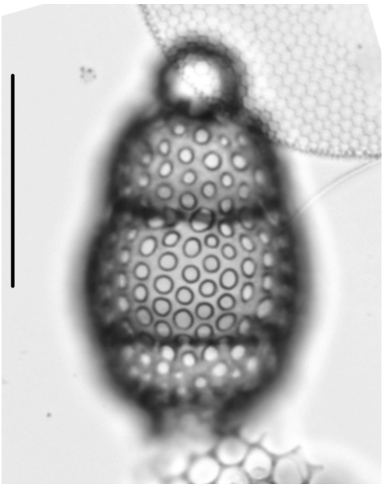

Stichocorys sp 1 cf

Theoperidae (eucyrtinid)

Undescribed

Records: 38

The abdomen of this species is its widest segment. It typically has three segments, with a semi-enclosed base. It differs in that it is more narrow and small than Cyrtocapsella sp 1 and Cyrtocapsella tetrapera.

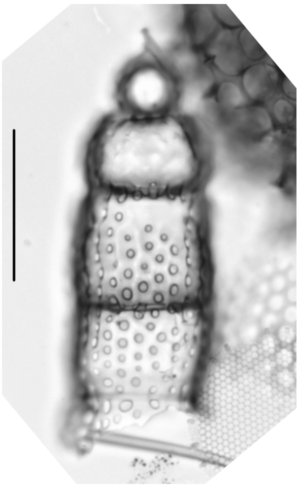

Stichocorys sp 2

Theoperidae (eucyrtinid)

Undescribed

Records: 180

This species often has a similar shape to S. delmontensis, or S. peregrina, but differs in its tendency to have a hyaline thorax, and a very rough and wrinkly shell exterior.

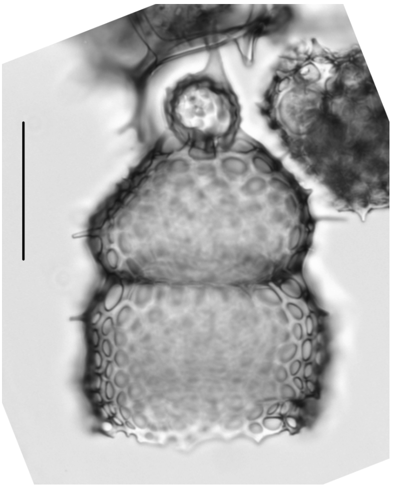

Stichocorys sp 3

Theoperidae (eucyrtinid)

Undescribed

Records: 11

This species is wider than other Stichocorys species, and has small thorns on thorax and abdomen.

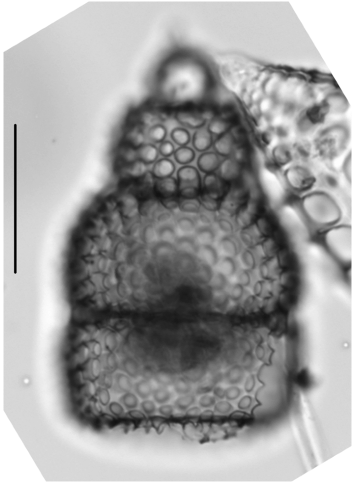

Stichocorys sp delmontensis-peregrina

Theoperidae (eucyrtinid)

Undescribed

Records: 17

This species appears to be an intermediate between S. delmontensis and S. peregrina, with similar size of third and fourth segments.

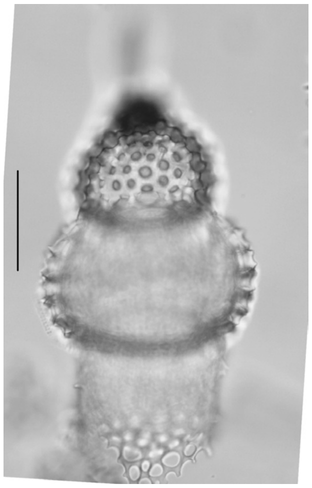

Stichocorys wolffii

Theoperidae (eucyrtinid)

Haeckel, 1887

Records: 13

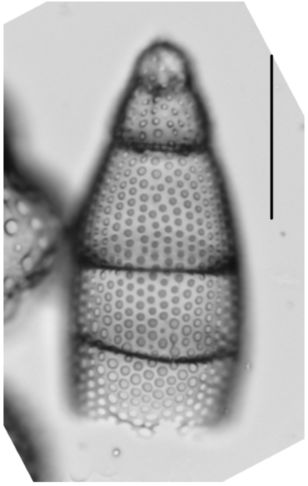

unknown theoperid sp 4

Theoperidae (eucyrtinid)

Undescribed

Records: 51

This species has a smooth, conical shape, with small, regular pores.

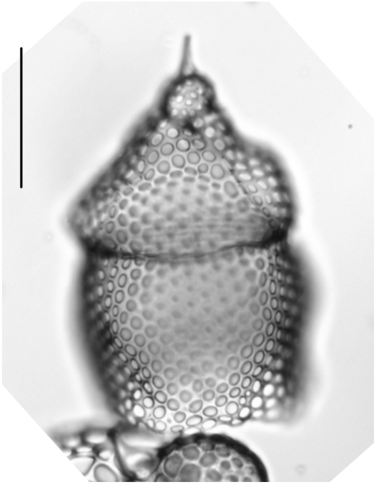

unknown theoperid sp 5

Theoperidae (eucyrtinid)

Undescribed

Records: 35

This species has a large, tent-like thorax, that becomes significantly wider as it approaches the abdomen. Abdomen is long, with no additional segments.

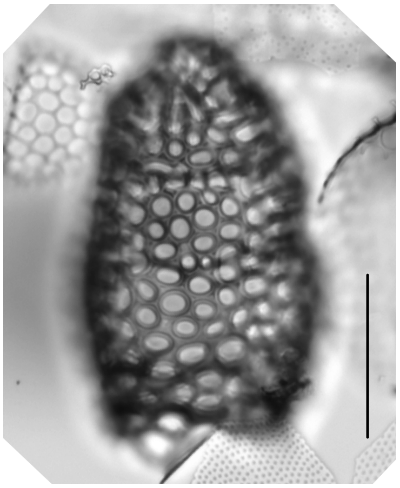

unknown theoperid sp 6

Theoperidae (eucyrtinid)

Undescribed

Records: 9

This species has large, irregular pores, and a lumpy outline. It is distinguished from Eucyrtidium sp 1 in that it is smaller, with shorter segments, and more irregular arrangement of pores.

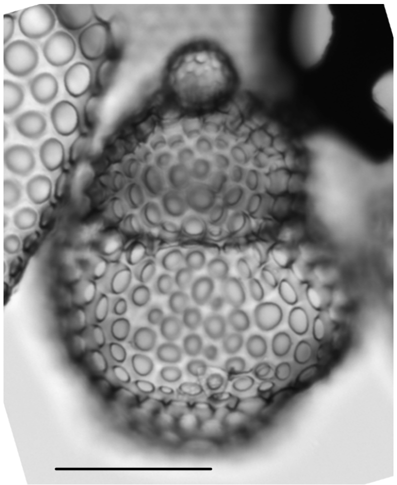

unknown theoperid sp 8

Theoperidae (eucyrtinid)

Undescribed

Records: 4

This species is distinguished for its unusually wide thorax and abdomen.

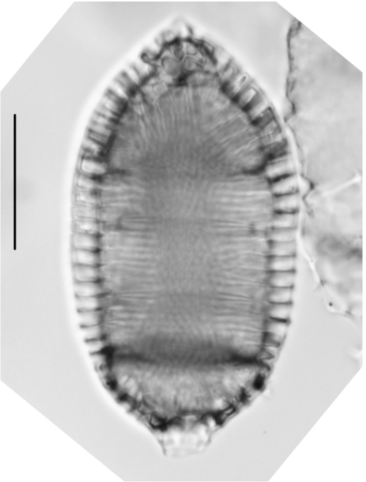

unknown theoperid sp 9

Theoperidae (eucyrtinid)

Undescribed

Records: 9

This species is similar to Lithocampe sp 1 of Nigrini 1967, but differs in that it does not have as many post-thoracic segments, segments are not as thick, and outer wall of shell is more robust.

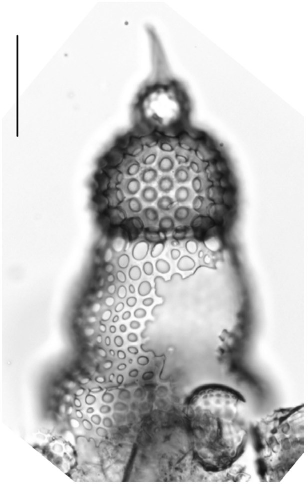

Lophocyrtis aspera

Theoperidae (lophocyrtid)

(Ehrenberg) Sanfilippo & Caulet, 1998

Records: 9

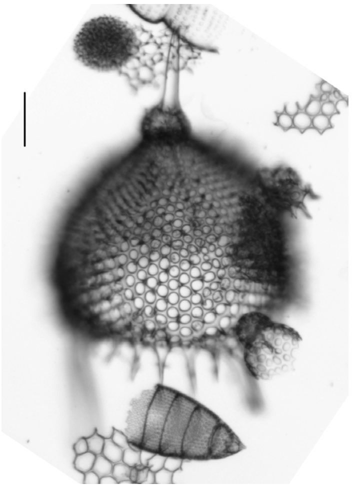

Lophocyrtis sp A Kamikuri

Theoperidae (lophocyrtid)

Kamikuri, 2017

Records: 27

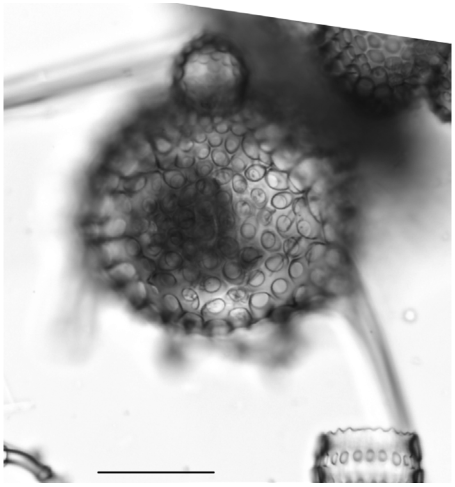

Lychnocanium nodosum

Theoperidae (pterocanid)

Haeckel, 1887

Records: 21

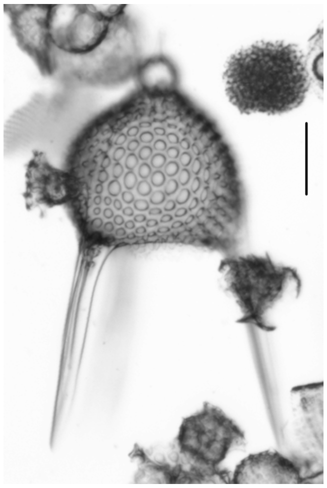

Lychnocanoma sp 1

Theoperidae (pterocanid)

Undescribed

Records: 71

This species is distinct for its large size, long thorax, and robust, slightly-curved, bladed feet.

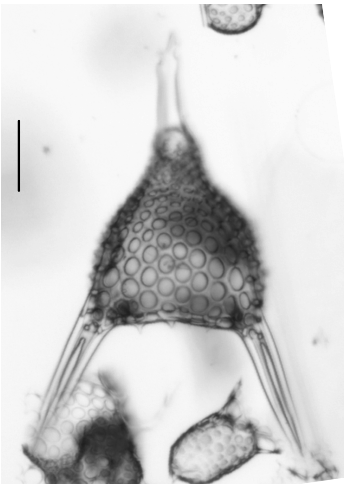

Pterocanium audax

Theoperidae (pterocanid)

Riedel, 1953

Records: 76

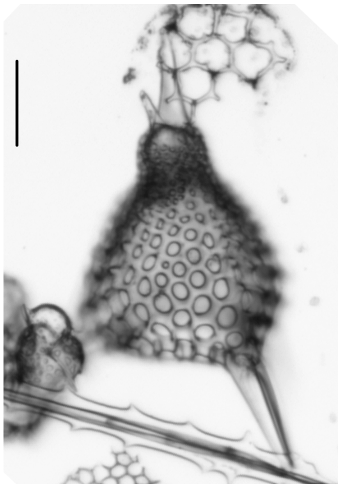

Pterocanium audax cf

Theoperidae (pterocanid)

Undescribed

Records: 9

This species resembles P. audax except that it has two spines on the cephalis, and the cephalis is relatively larger compared to the rest of the shell. There is also a less constricted neck on this species, versus P. audax.

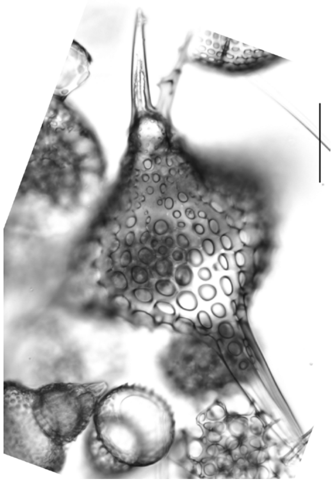

Pterocanium audax cf sp 2

Theoperidae (pterocanid)

Undescribed

Records: 10

This species resembles P. audax, but has two cephalic spines of similar size, and thorax has a different shape -- it is relatively short and wide compared to P. audax.

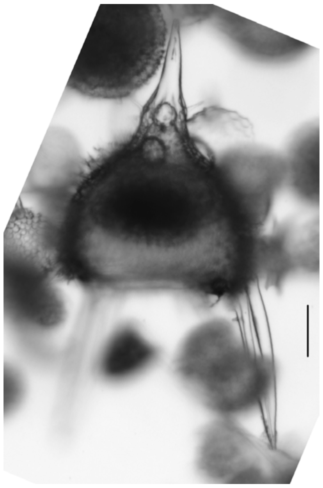

Pterocanium audax cf sp 3

Theoperidae (pterocanid)

Undescribed

Records: 31

This species has a thorax similar in shape to P. audax, but is at least 50% larger in size, with an apical spine to robust that it engulfs the small cephalis. Exterior of the shell also much thornier than P. audax.

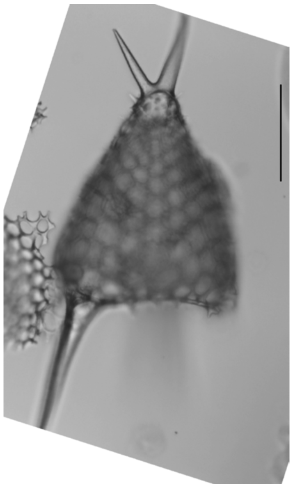

Pterocanium auritum

Theoperidae (pterocanid)

Nigrini & Caulet, 1992

Records: 40

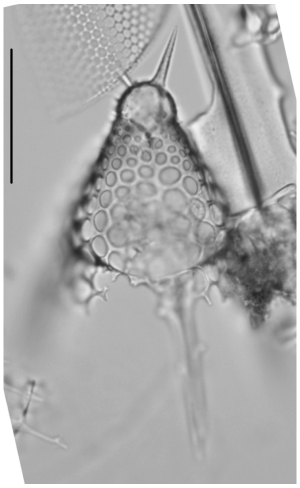

Pterocanium auritum cf
Theoperidae (pterocanid)
Undescribed
Records: 28

This species has many similar features to P. auritum, but it is consistently about half its size. The cephalis sizes are identical, however.

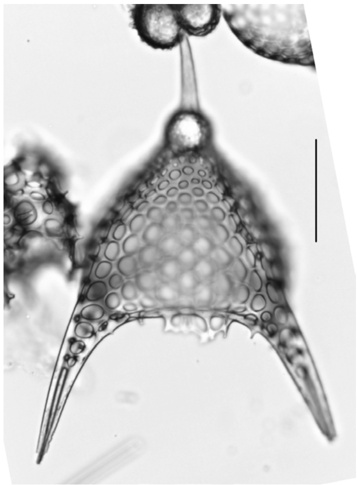

Pterocanium charybdeum trilobum
Theoperidae (pterocanid)
Haeckel, 1860
Records: 112

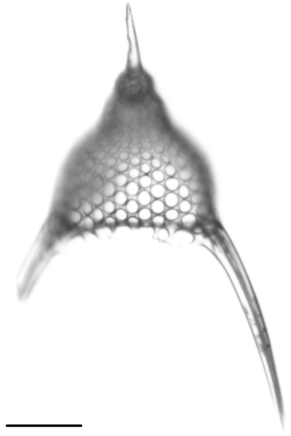

Pterocanium grandiporus
Theoperidae (pterocanid)
Nigrini, 1968
Records: 72

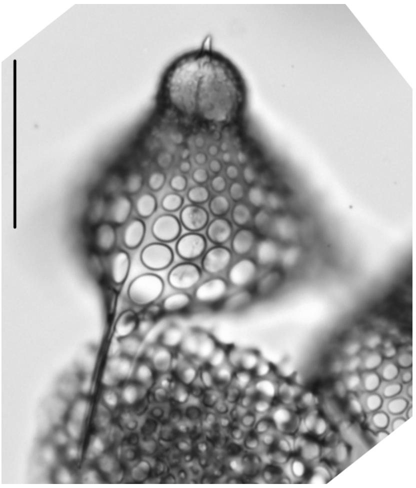

Pterocanium korotnevi
Theoperidae (pterocanid)
Dogel & Reshetnyak, 1952
Records: 18

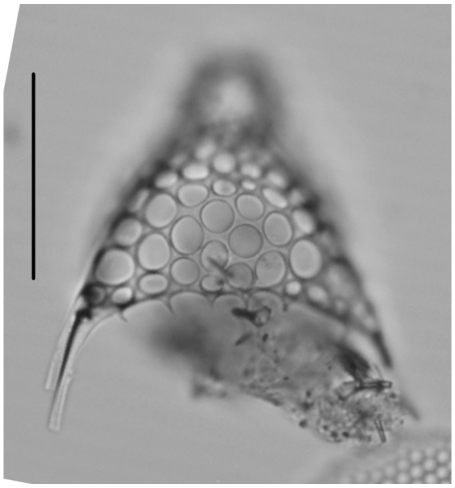

Pterocanium korotnevi cf
Theoperidae (pterocanid)
Undescribed
Records: 31

This species is a similar size to P. korotnevi, but differs in the curved base of its thorax, that bends upwards between each set of feet.

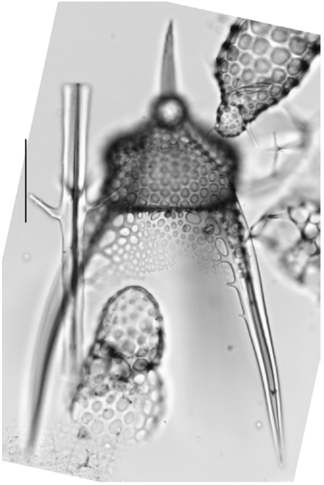

Pterocanium praetextum
Theoperidae (pterocanid)
(Ehrenberg) Haeckel, 1887
Records: 68

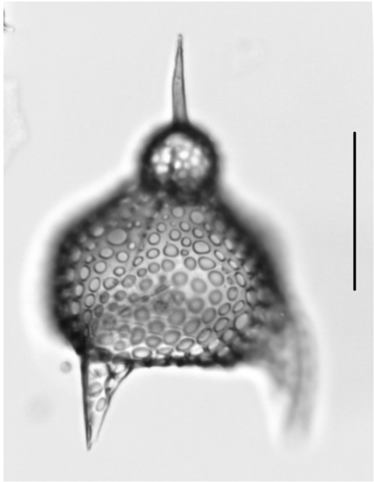

Pterocanium praetextum mini

Theoperidae (pterocanid)

Undescribed

Records: 12

Similar shape and features to P. praetextum, but significantly smaller.

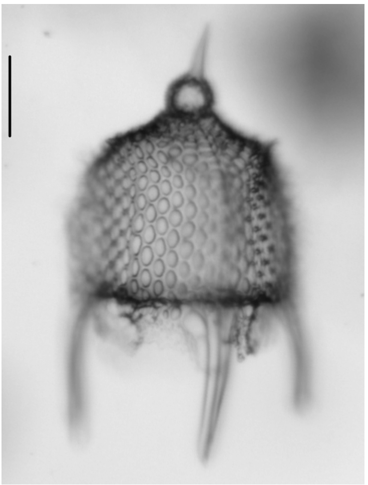

Pterocanium prismatium

Theoperidae (pterocanid)

Riedel, 1957

Records: 32

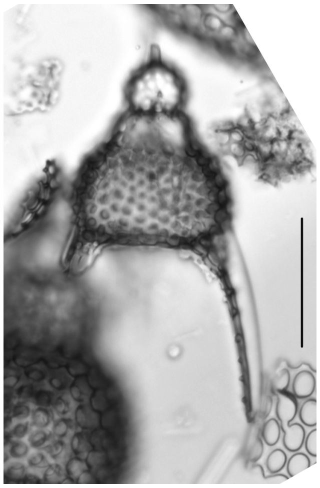

Pterocanium sp 1

Theoperidae (pterocanid)

Undescribed

Records: 16

This species is unique for its thorax shape, which has a small hyaline area toward the top, and flares out with a thickly-silicified, evenly-pored area from the middle to the base. Cephalis has two conical horns.

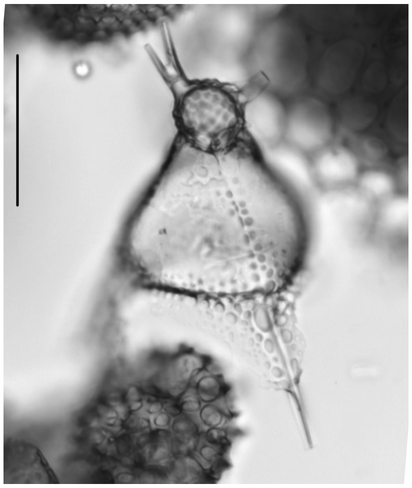

Pterocanium sp 2

Theoperidae (pterocanid)

Undescribed

Records: 14

This species is unique for its nearly-hyaline thorax, and two cephalic spines.

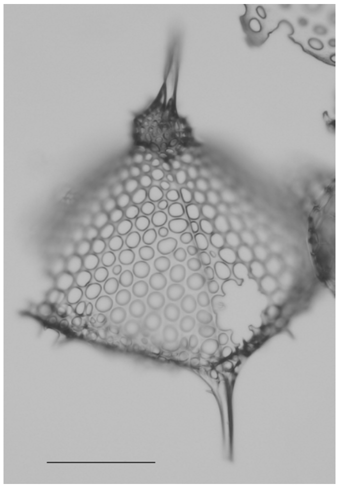

Pterocanium sp 3

Theoperidae (pterocanid)

Undescribed

Records: 44

This species differs from Pterocanium charybdeum trilobum in that it has a narrow double-layer of pores encircling the base of the thorax, has smaller, more regular, pores throughout thorax, and thorax is consistently pyramidal in shape.

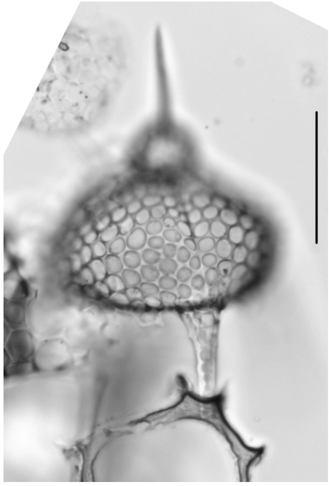

Pterocanium trilobum cf

Theoperidae (pterocanid)

Undescribed

Records: 19

This species resembles P. trilobum, but has a much flatter thorax. This species may be synonymous with P. depressum.

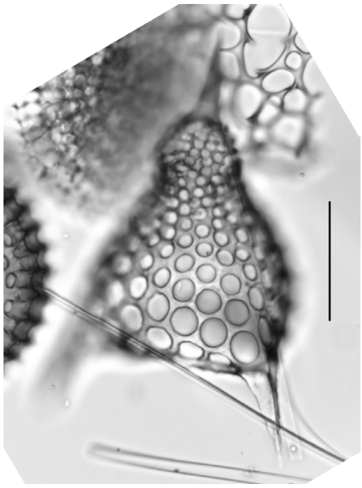

unknown pterocanid sp 2

Theoperidae (pterocanid)

Undescribed

Records: 25

This species is distinct for its elongated thorax, and relatively large, pored cephalis.

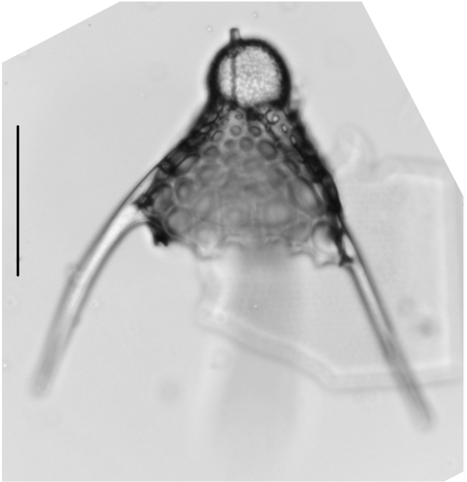

unknown pterocanid sp 6

Theoperidae (pterocanid)

Undescribed

Records: 13

This species has thin, conical feet with a matching apical spine. Cephalis is relatively lightly silicified compared to thorax.

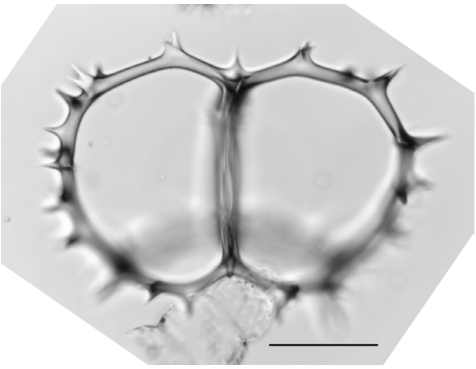

Acanthodesmia micropora

Trissocyclidae

(Popofsky) Petrushevskaya, 1971

Records: 86

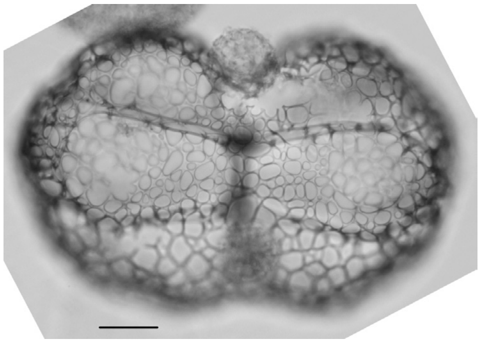

Amphispyris roggentheni

Trissocyclidae

Goll, 1980

Records: 26

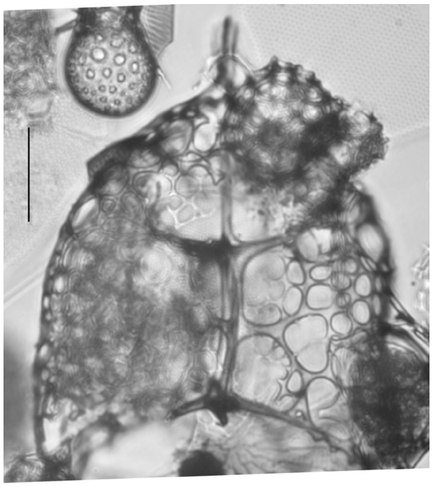

Androspyris anthropiscus

Trissocyclidae

Haeckel, 1887

Records: 24

sensu Van de Paverd, 1995

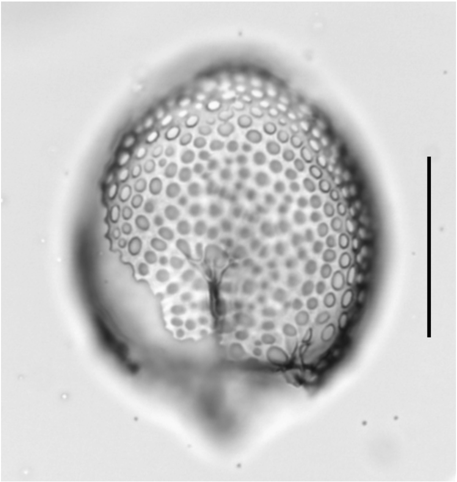

Cantharospyris platybursa

Trissocyclidae

Haeckel, 1887

Records: 19

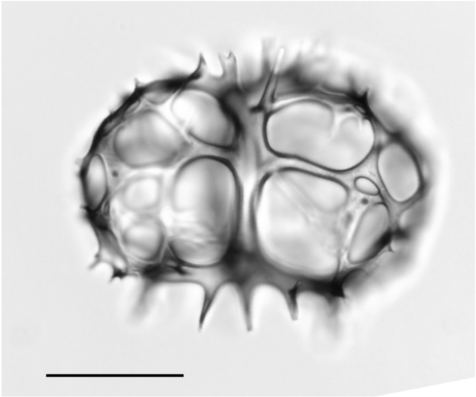

Ceratospyris borealis
Trissocyclidae
Bailey, 1856
Records: 76

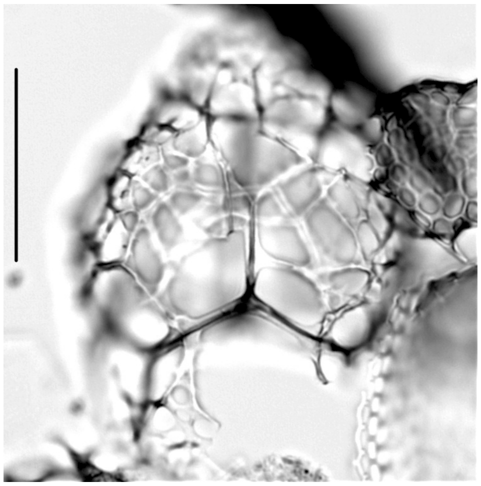

Ceratospyris laventaensis
Trissocyclidae
Campbell & Clark, 1944
Records: 47

sensu Renaudie

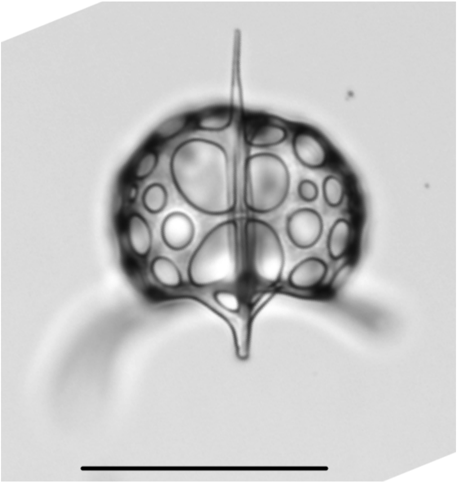

Corythospyris fiscella
Trissocyclidae
Goll, 1978
Records: 101

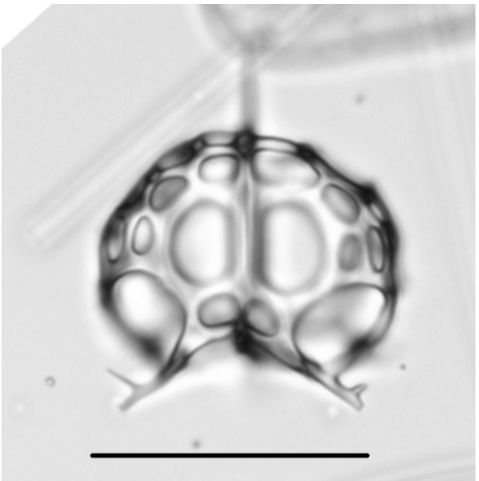

Corythospyris reuschi
Trissocyclidae
Goll & Bjørklund, 1989
Records: 17

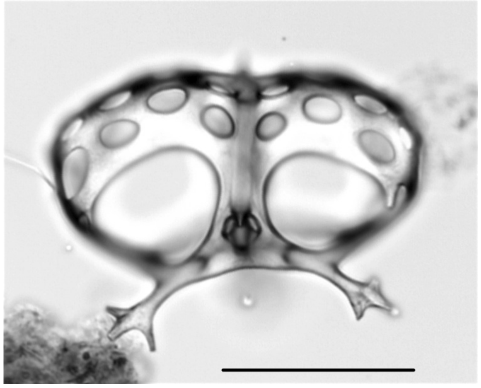

Corythospyris stapedius
Trissocyclidae
(Haeckel) Goll, 1978
Records: 126

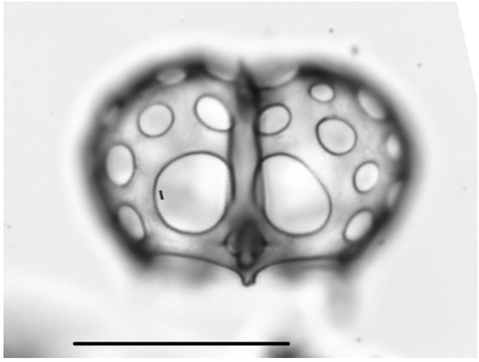

Corythospyris? sp 1
Trissocyclidae
Undescribed
Records: 139

This species has one set of large pores at the base of the D-ring, and elsewhere has small, regular pores of the same size. Three muted feet are also present. Shell exterior is smooth.

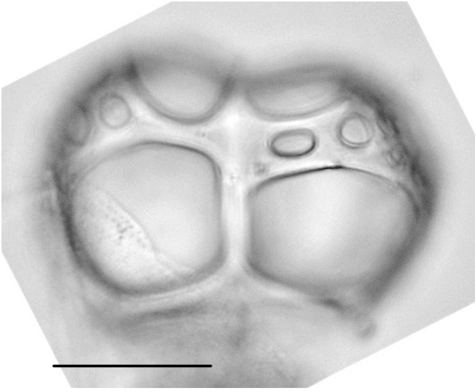

Corythospyris? sp 2

Trissocyclidae

Undescribed

Records: 55

This species has a distinctly asymmetrical distribution of pores on either lobe.

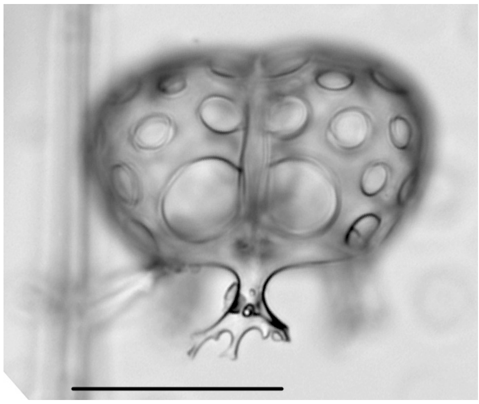

Corythospyris? sp 3

Trissocyclidae

Undescribed

Records: 55

This species resembles Corythospyris sp 1 but differs in that it has dendritic feet.

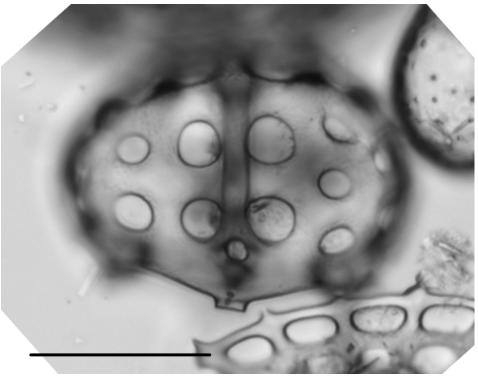

Dendrospyris pododendros

Trissocyclidae

(Carnevale) Goll, 1968

Records: 8

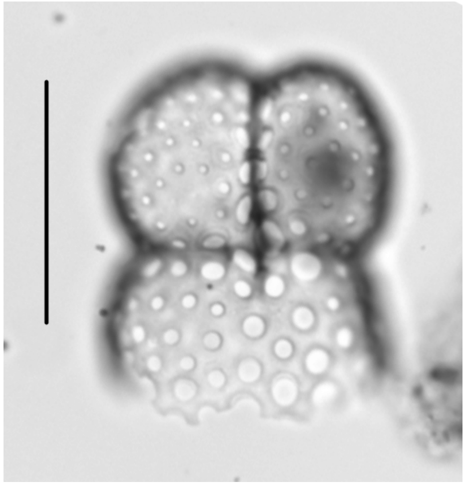

Dendrospyris sp 1

Trissocyclidae

Undescribed

Records: 30

This species is defined by its small pores and very light silicification. Thin apical horn is occasionally preserved.

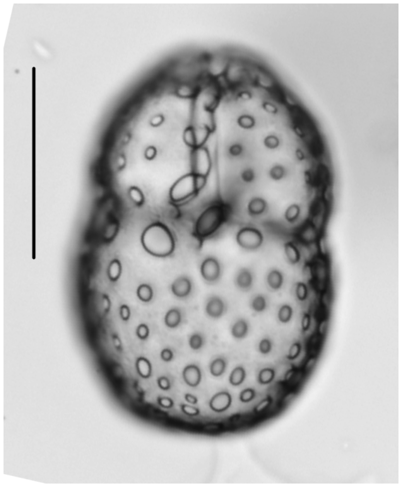

Dendrospyris sp 2

Trissocyclidae

Undescribed

Records: 5

This species is distinguishable for its small lobes around the D-ring, and enclosed basal lobe.

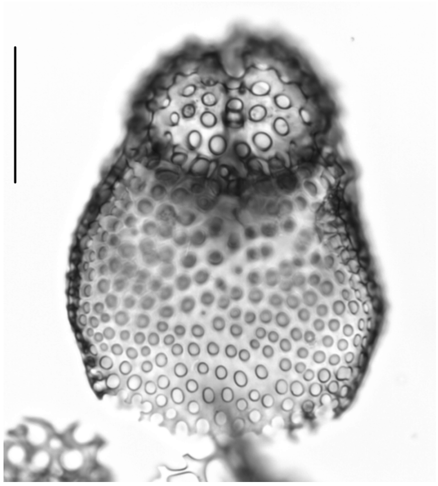

Dendrospyris sp A Kamikuri

Trissocyclidae

Kamikuri, 2017

Records: 47

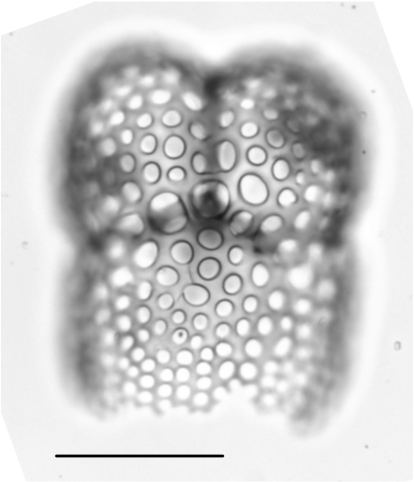

Dendrospyris stabilis

Trissocyclidae

Goll, 1968

Records: 125

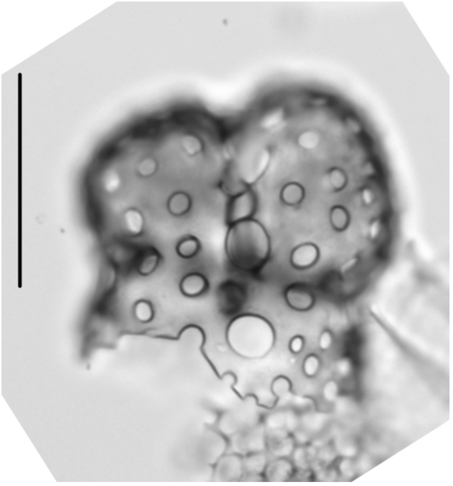

Dendrospyris stabilis cf

Trissocyclidae

Undescribed

Records: 28

This species is similar to D. stabilis, except that lobes are asymmetrical, one larger than the other. It also tends to be heavily-silicified, and occasionally has an extra deposit at the apex of the shell.

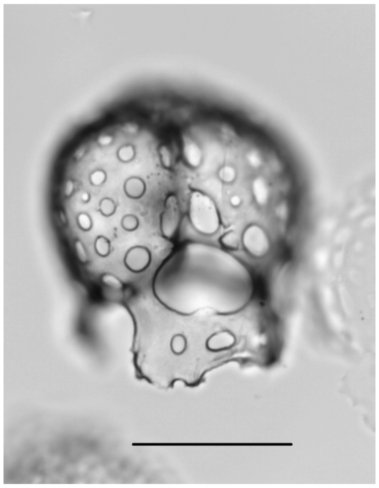

Dendrospyris suganoi cf

Trissocyclidae

Undescribed

Records: 59

This species is significantly less robust than the species described by Sugiyama and Furutani, 1992. But it has similar irregular pores on thorax, including one large pore in the center.

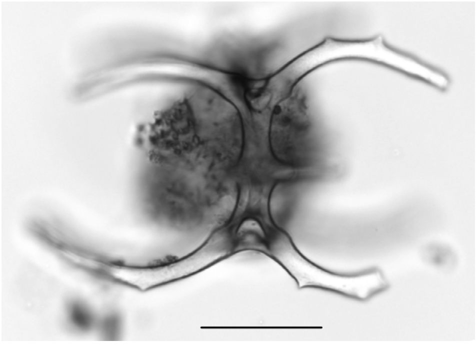

Eucoronis perspicillum

Trissocyclidae

Haeckel, 1887

Records: 22

sensu Kamikuri, 2017

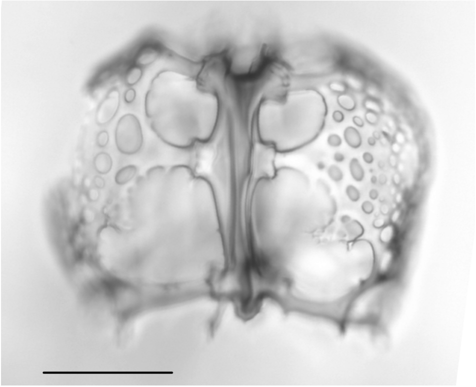

Giraffospyris annulispina

Trissocyclidae

Goll, 1969

Records: 45

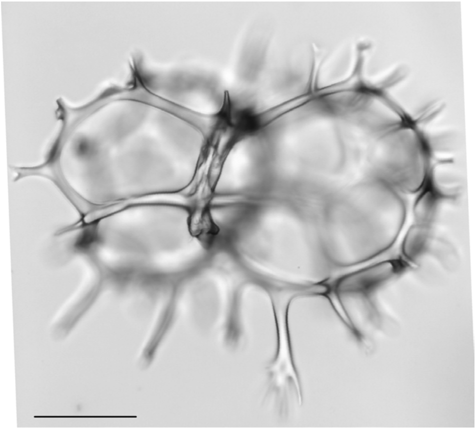

Giraffospyris circumflexa

Trissocyclidae

Goll, 1969

Records: 72

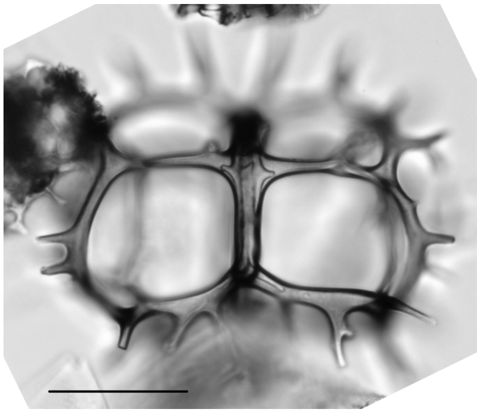

Giraffospyris sp 1
Trissocyclidae
Undescribed
Records: 98

This species has similar spines to G. circumflexa, but differs in that it has fewer branches in the shell structure. Differs from A. micropora in that its spines are more pronounced.

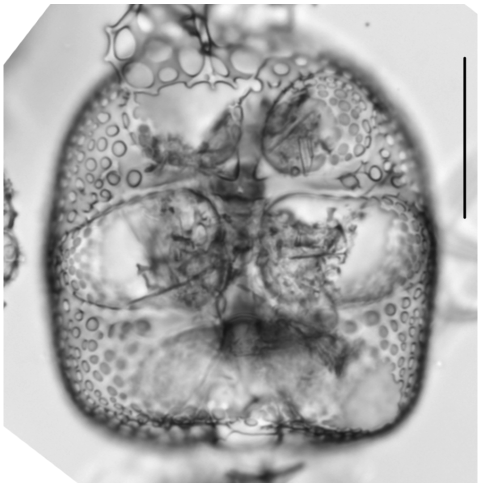

Liriospyris ovalis
Trissocyclidae
Goll, 1968
Records: 28

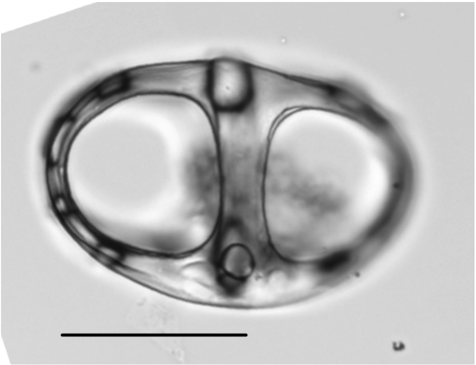

Liriospyris parkerae
Trissocyclidae
Riedel & Sanfilippo, 1971
Records: 7

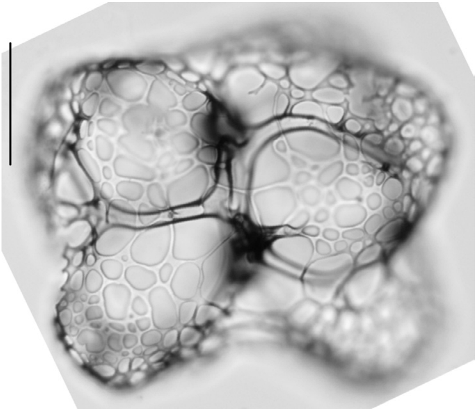

Liriospyris reticulata
Trissocyclidae
(Ehrenberg) Goll, 1968
Records: 106

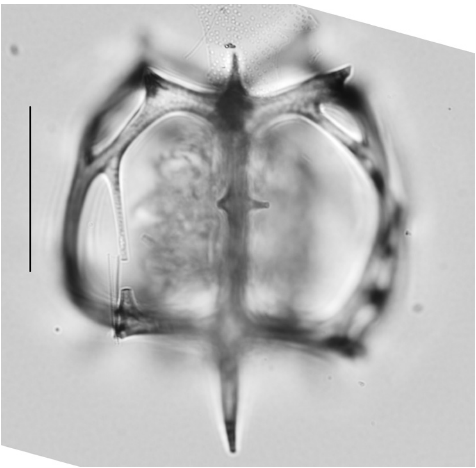

Lophospyris cheni
Trissocyclidae
Goll, 1976
Records: 36

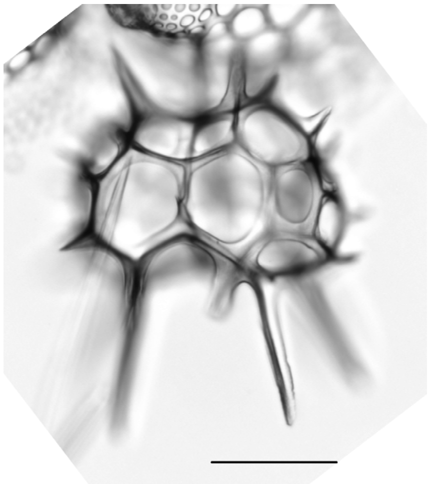

Lophospyris pentagona pentagona
Trissocyclidae
(Ehrenberg) Petrushevskaya, 1971
Records: 79

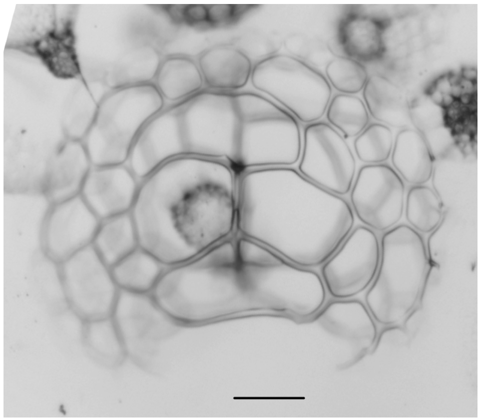

Nephrospyris renilla
Trissocyclidae
Haeckel, 1887
Records: 20

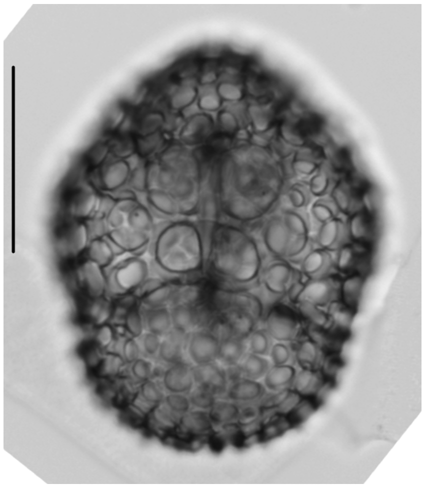

Tholospyris baconiana
Trissocyclidae
Goll, 1972
Records: 15

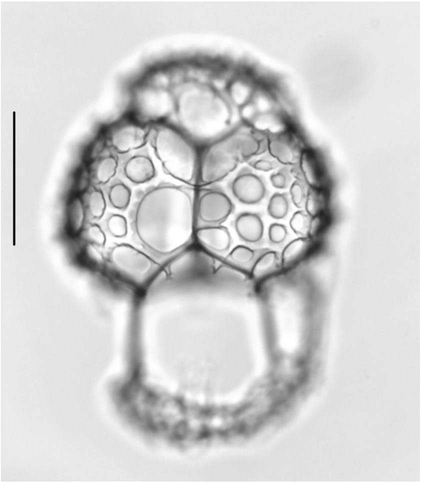

Tholospyris baconiana variabilis
Trissocyclidae
Goll, 1972
Records: 99

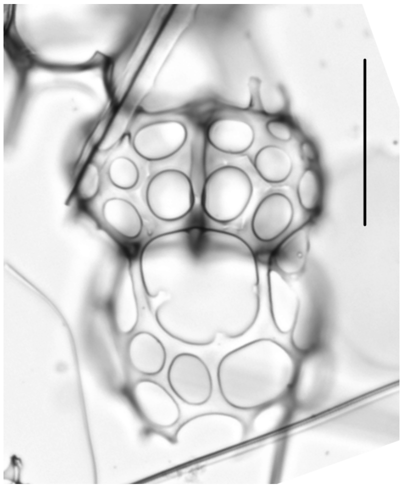

Tholospyris baconiana cf
Trissocyclidae
Undescribed
Records: 21

This species is different from T. baconiana for its very large pores on the veil.

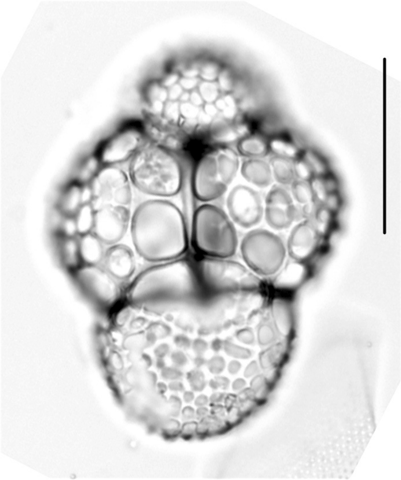

Tholospyris baconiana cf sp 2
Trissocyclidae
Undescribed
Records: 117

This species resembles T. baconiana, but has a thinner veil structure around the main part of the shell, that does not fully encompass the main shell as it does in T. baconiana.

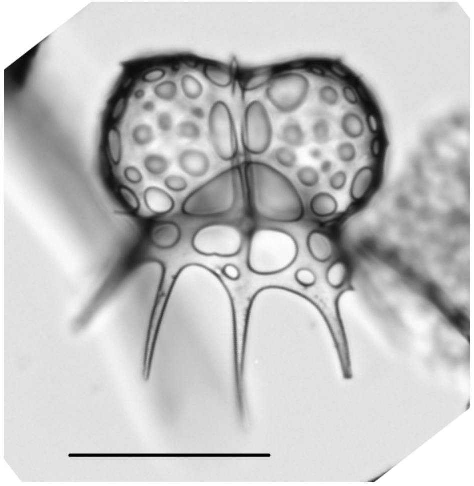

Tholospyris capoi
Trissocyclidae
(Goll) De Wever et al., 1990
Records: 100

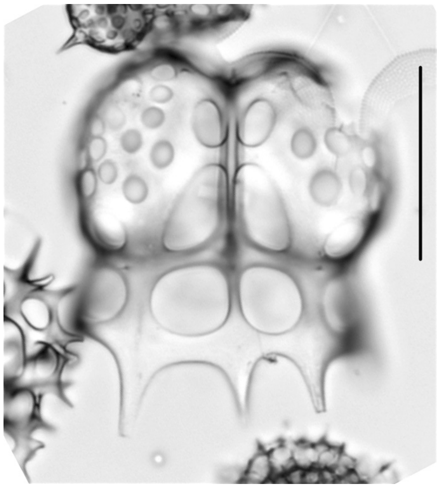

Tholospyris capoi cf

Trissocyclidae

Undescribed

Records: 25

This species has a cephalis somewhat similar to T. capoi, but the base differs with its very large pores.

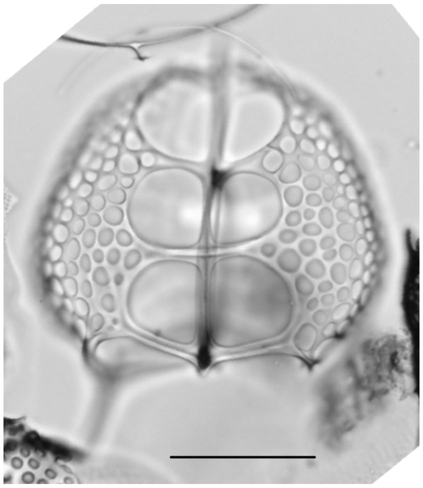

Tholospyris cortinisca

Trissocyclidae

Haeckel, 1887

Records: 133

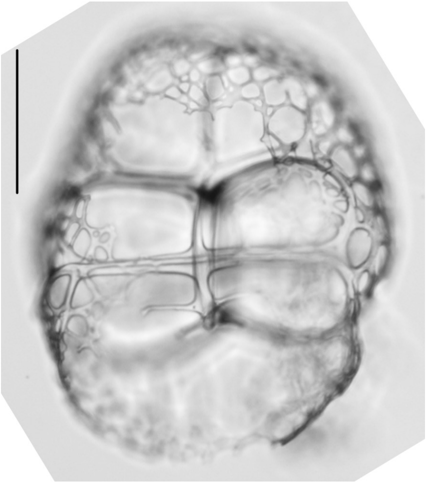

Tholospyris devexa

Trissocyclidae

Goll, 1969

Records: 68

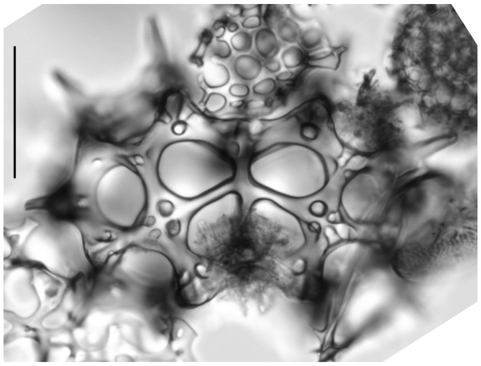

Tholospyris mammillaris

Trissocyclidae

Haeckel, 1887

Records: 32

sensu Kamikuri, 2017

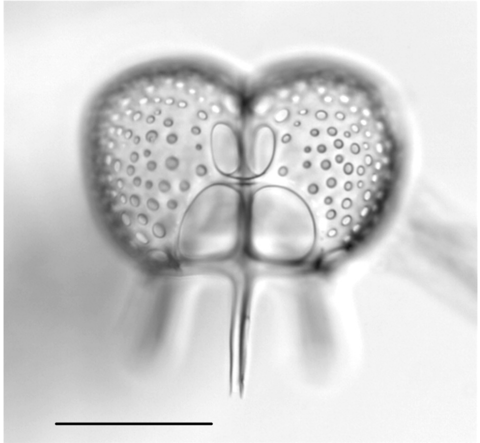

Tholospyris scaphipes

Trissocyclidae

(Haeckel) Goll, 1969

Records: 69

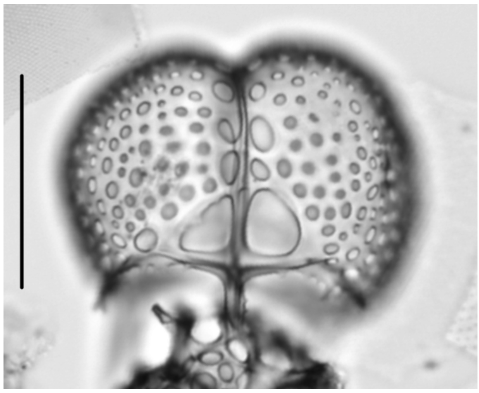

Tholospyris scaphipes cf

Trissocyclidae

Undescribed

Records: 16

This species is distinguished from T. scaphipes for its relatively larger size, and additional pores along the axis of the D-ring.

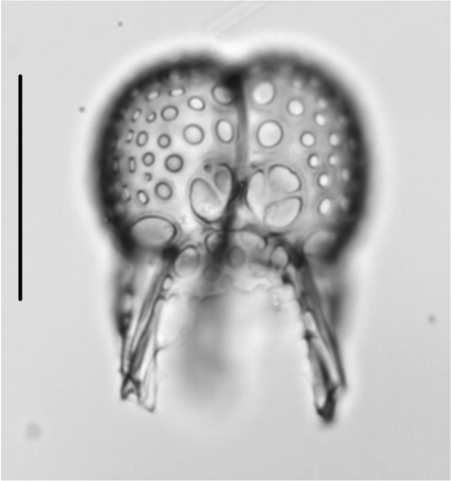

Tholospyris scaphipes cf sp 2

Trissocyclidae

Undescribed

Records: 59

This species resembles T. scaphipes, but has an additional thin latticed veil over main shell and feet.

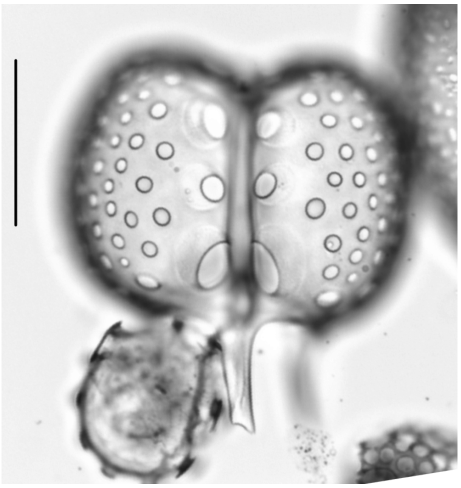

Tholospyris scaphipes square fm

Trissocyclidae

Undescribed

Records: 65

This species is similar to T. scaphipes, except that it has a more square outline.

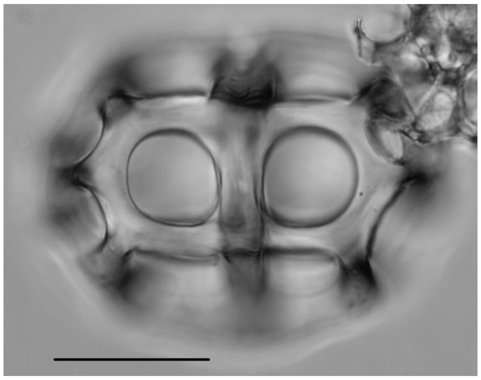

Tympanidium binoctonum

Trissocyclidae

Haeckel, 1887

Records: 46

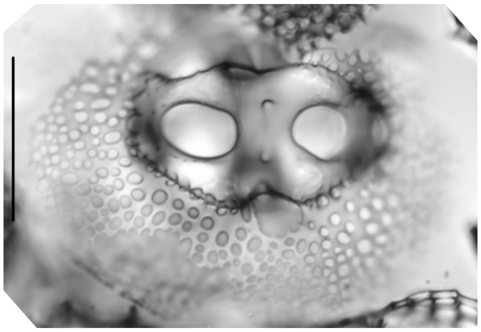

Tympanidium binoctonum cf sp 2

Trissocyclidae

Undescribed

Records: 75

This species resembles the shape of T. binoctonum, but differs in that it has a fine lattice developed around the shell.

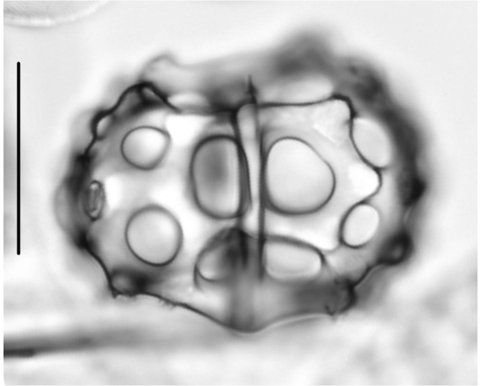

Tympanidium binoctonum cf sp 3

Trissocyclidae

Undescribed

Records: 84

This species resembles the pore structure of T. binoctonum, but differs in that it has bumps at the junctions of its bars. This species also tends to be smaller in overall size.

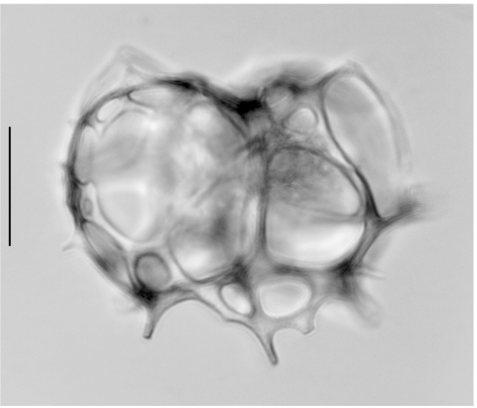

unknown trissocyclid group A sp 1

Trissocyclidae

Undescribed

Records: 16

This species is unique for its large, angular pores that rise above the ends of each lobe.

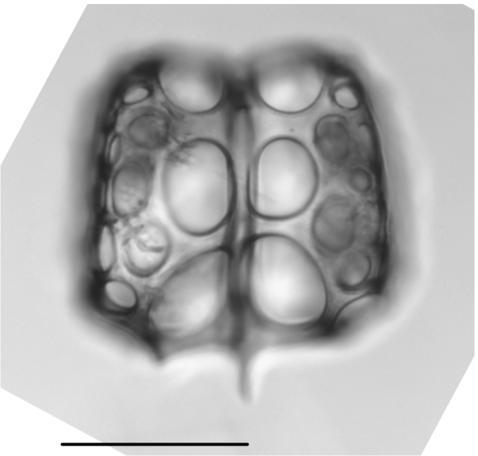

unknown trissocyclid group A sp 10

Trissocyclidae

Undescribed

Records: 110

This species is distinctive for its square shape, and three sets of mirrored pores along the D-ring on each side of the shell.

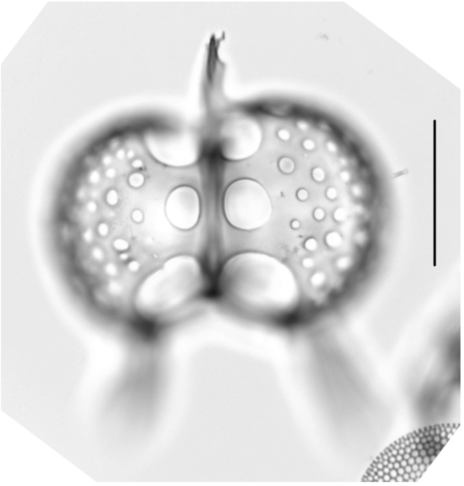

unknown trissocyclid group A sp 10 cf sp 2

Trissocyclidae

Undescribed

Records: 55

This species has three sets of large pores at the D-ring, like unknown trissocyclid group A sp 10. But, it differs in having a more rounded shape to each of the lobes.

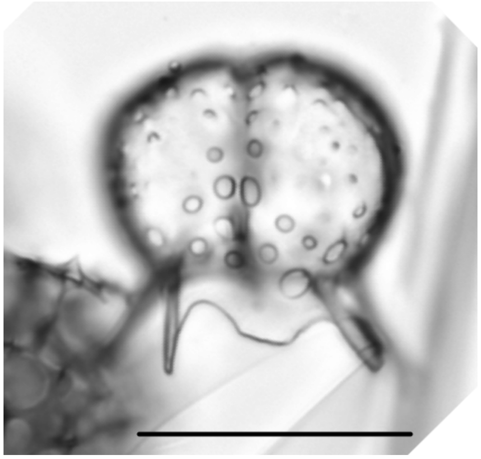

unknown trissocyclid group A sp 11

Trissocyclidae

Undescribed

Records: 24

This species has rounded lobes, irregular rounded pores, and lacks abundant spines, differentiating it from Tholospyris capoi.

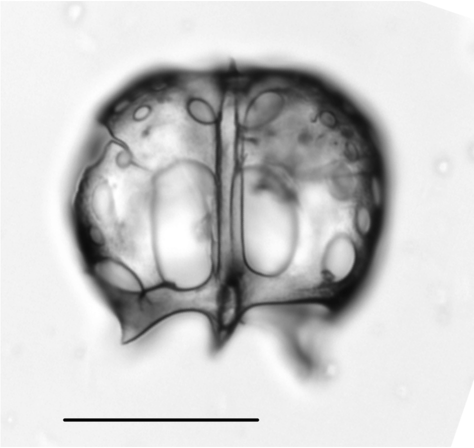

unknown trissocyclid group A sp 11 cf

Trissocyclidae

Undescribed

Records: 26

This species resembles unknown latticed so 11, but differs in that it only has two sets of mirrored pores on each side of the D-ring.

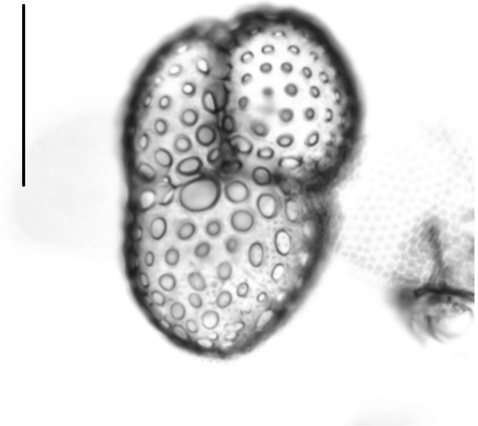

unknown trissocyclid group A sp 12

Trissocyclidae

Undescribed

Records: 17

This species resembles Dendrospyris stabilis, but differs in that it has an enclosed base. It also differs from Dendrospyris sp 2 in that the cephalis is wider than the thorax.

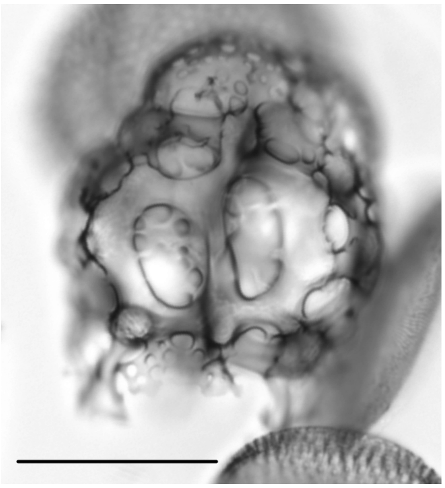

unknown trissocyclid group A sp 15

Trissocyclidae

Undescribed

Records: 13

This species is small, with large, irregular pores that do not mirror each other on either side of the D-ring. It has a fine, latticed veil on both top and bottom of the shell.

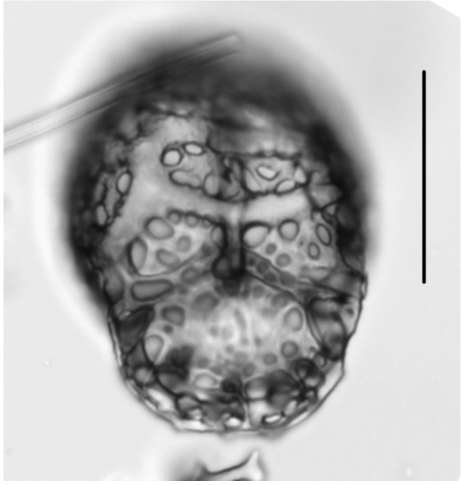

unknown trissocyclid group A sp 15 cf

Trissocyclidae

Undescribed

Records: 4

This species has strong, smooth bars, and more regular pores, differentiating it from unknown trissocyclid group A sp 15.

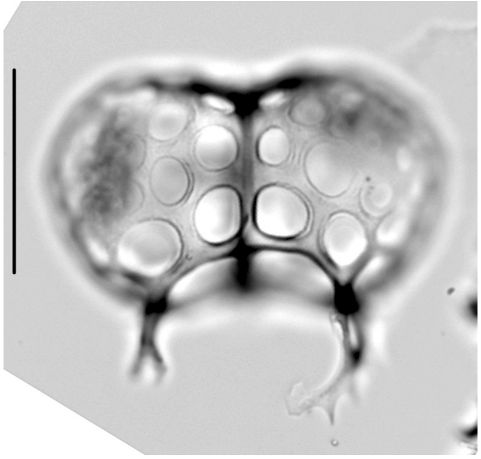

unknown trissocyclid group A sp 17

Trissocyclidae

Undescribed

Records: 38

This species differs from Tholospyris scaphipes in that it has less of a size differential between pores adjacent to D-ring and elsewhere on the shell.
It also has curved, branching feet, rather than straight and bladed.

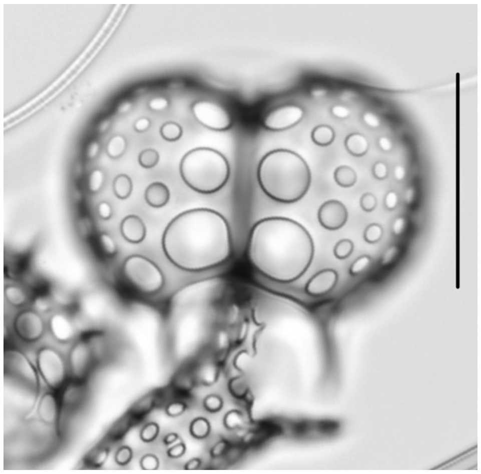

unknown trissocyclid group A sp 17 cf

Trissocyclidae

Undescribed

Records: 94

This species is highly variable. It is defined by having a row of slightly-enlarged pores adjacent to the D-ring, small, straight feet, and a smooth exterior that is sub-rounded.

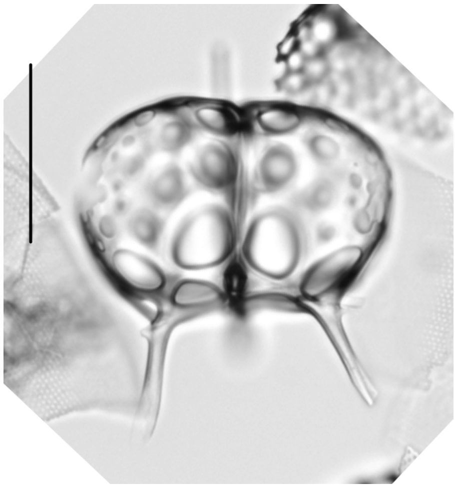

unknown trissocyclid group A sp 18

Trissocyclidae

Undescribed

Records: 23

This species has an apical horn, conical feet, and reaches its widest point above the center of the main lobes.

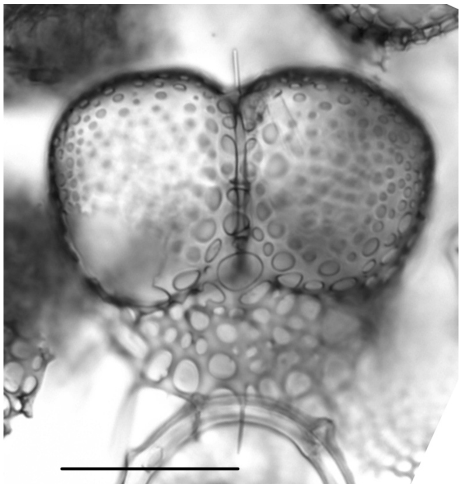

unknown trissocyclid group A sp 19

Trissocyclidae

Undescribed

Records: 32

This species is relatively large, with small pores, and an overall shape resembling the Dendrospyris genus. The thorax is comprised of irregular, angular pores with many spines. Small apical horn is present.

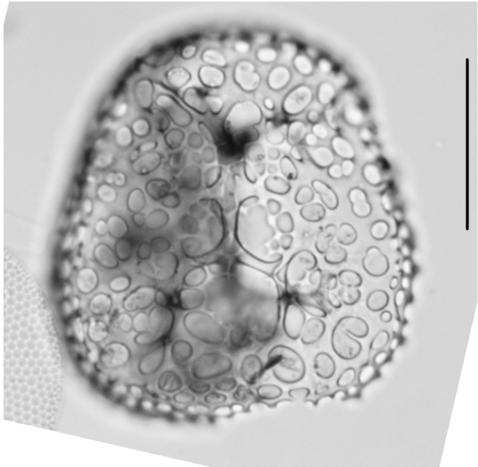

unknown trissocyclid group A sp 2

Trissocyclidae

Undescribed

Records: 49

This species has a distinct shape, and no clear differentiation between the main body of the shell and the lattice veil. Pores have a unique partitioning pattern.

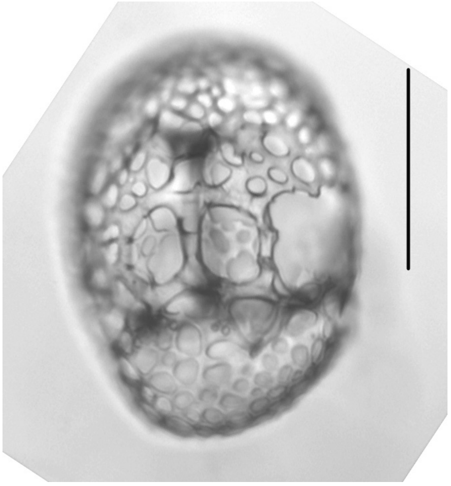

unknown trissocyclid group A sp 2 cf

Trissocyclidae

Undescribed

Records: 16

This species resembles unknown trissocyclid group A sp 2, but is smaller and has a less distinct, more oval overall shape.

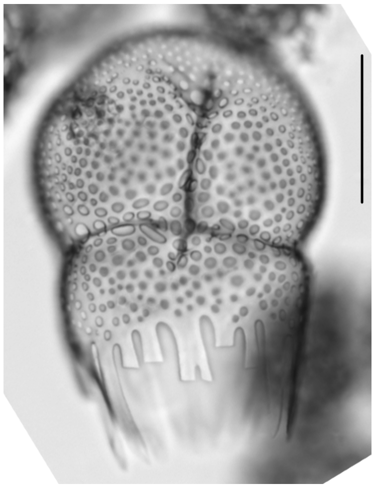

unknown trissocyclid group A sp 21

Trissocyclidae

Undescribed

Records: 47

This species resembles Dendrospyris stabilis, but differs in that it has long teeth at its base, smaller pores throughout, occasional hyaline area, and sometimes lacks an indentation between lobes, creating a completely round outline.

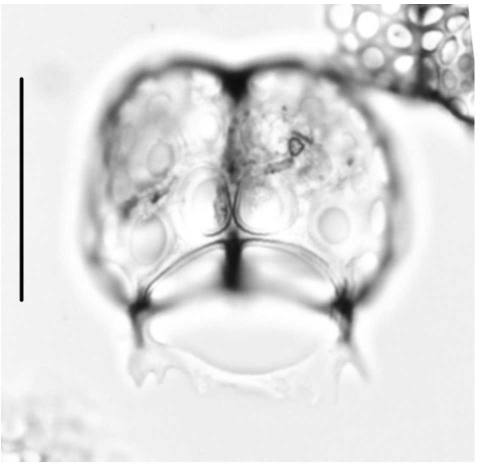

unknown trissocyclid group A sp 26

Trissocyclidae

Undescribed

Records: 22

This species is widest at the base of the main shell, has small pores, and has an enclosed bar that connects two of its feet. It is small and tends to be lightly silicified.

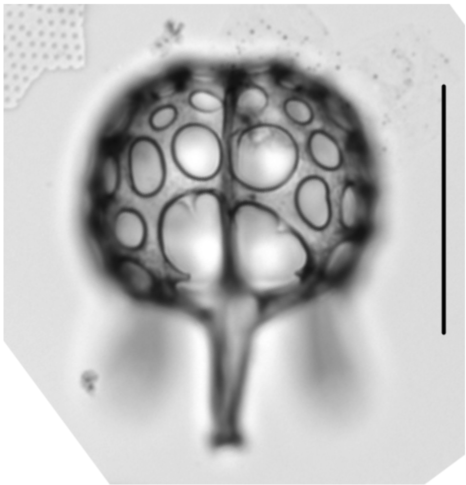

unknown trissocyclid group A sp 27

Trissocyclidae

Undescribed

Records: 25

This species is small and round, with no indentation between lobes. Feet are bladed and tend to angle outward.

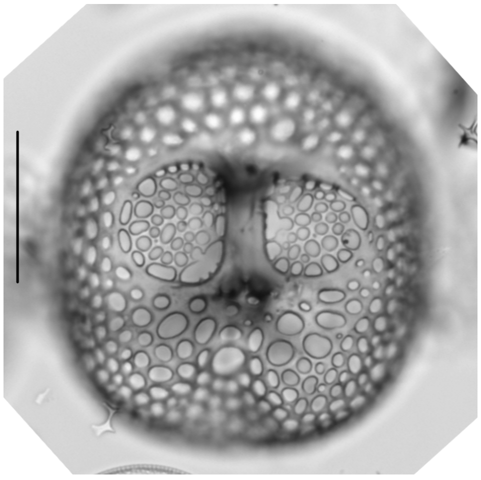

unknown trissocyclid group A sp 28

Trissocyclidae

Undescribed

Records: 18

This species is similar to Liriospyris ovalis, but differs in that it has only one set of large, latticed-over shell openings, is more round in overall outline, and lacks a pylome.

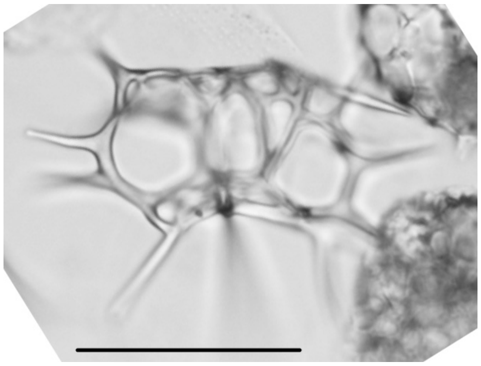

unknown trissocyclid group A sp 29

Trissocyclidae

Undescribed

Records: 12

This species is distinct for its incomplete lobes that end in spines.

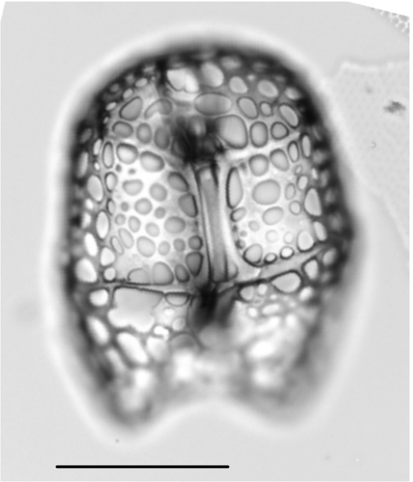

unknown trissocyclid group A sp 3

Trissocyclidae

Undescribed

Records: 16

This species resembles L. reticulata, but differs in that it is much smaller, has a quadrate outline, and is more elongated in one direction.

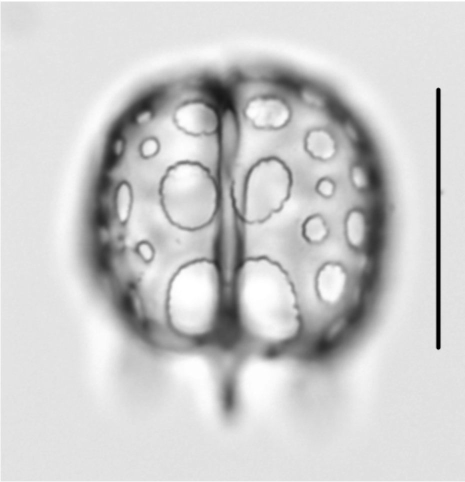

unknown trissocyclid group A sp 30

Trissocyclidae

Undescribed

Records: 75

This species has pores that over show a finely-latticed overgrowth. It is small and tends to be rectangular in outline. Three feet are usually preserved. The top of this species' shell is slightly rounded, distinguishing it from unknown trissocyclid group A sp 10.

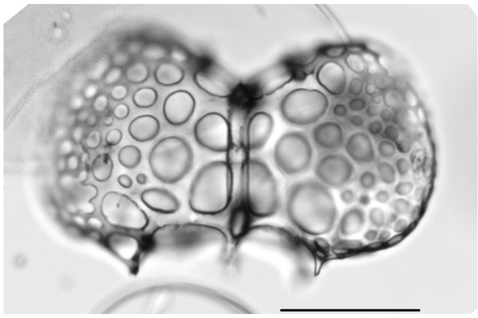

unknown trissocyclid group A sp 31

Trissocyclidae

Undescribed

Records: 17

This species has widely-set lobes, with much smaller pores on the ends of the lobes than near the D-ring.

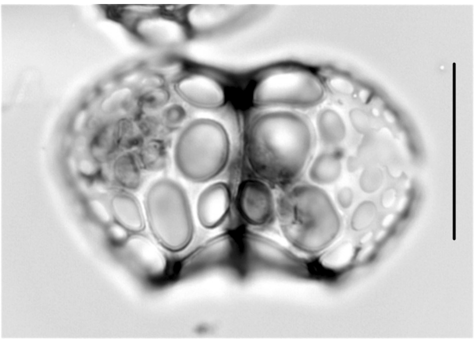

unknown trissocyclid group A sp 31 cf

Trissocyclidae

Undescribed

Records: 24

This species is similar to sp 31, but differs in that it is significantly smaller and is relatively more tall than wide.

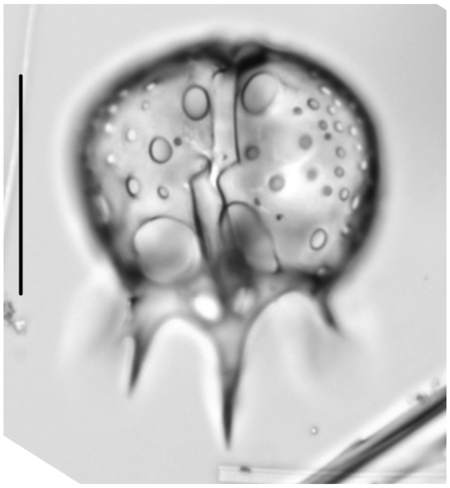

unknown trissocyclid group A sp 33

Trissocyclidae

Undescribed

Records: 208

This species is tallest at its D-ring, which is higher than the lobes. Lobes are smooth and rounded. Feet are typically present. Differs from unknown trissocyclid group A sp 30 in its smooth exterior lacking secondary lattice, and differs from unknown trissocyclid group A sp 10 in its rounder shape.

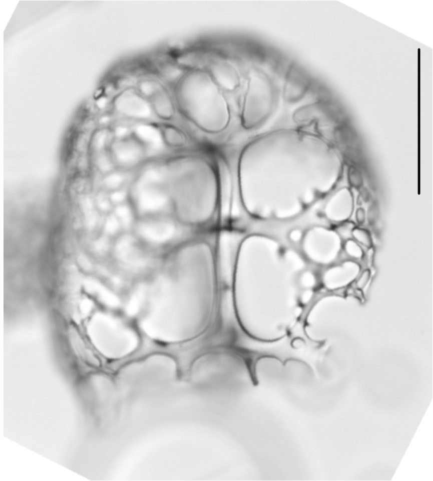

unknown trissocyclid group A sp 34

Trissocyclidae

Undescribed

Records: 14

This species is somewhat similar to unknown trissocyclid group A sp 2, but differs in its more long and narrow shape.

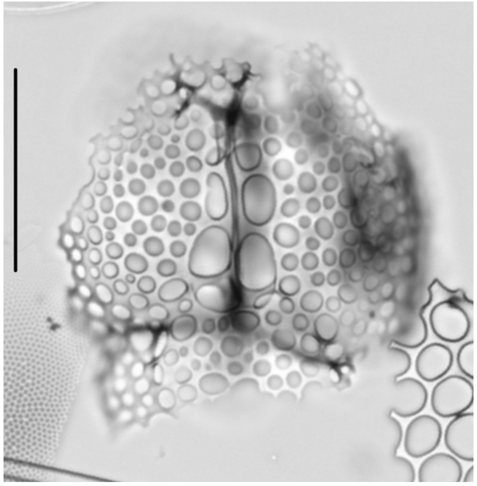

unknown trissocyclid group A sp 35

Trissocyclidae

Undescribed

Records: 8

This species resembles unknown trissocyclid group A species 21, but differs in that it has larger, different-shaped pores adjacent to the D-ring, a relatively narrow thorax, and no visible teeth.

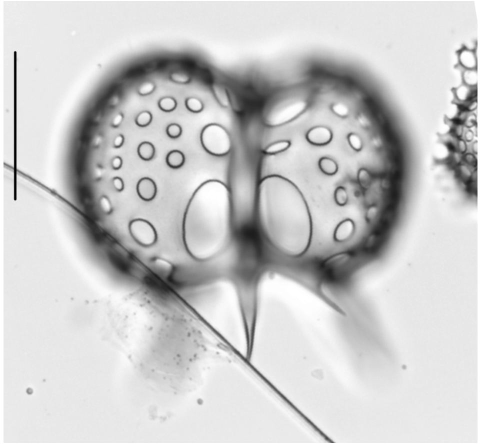

unknown trissocyclid group A sp 36

Trissocyclidae

Undescribed

Records: 34

This species differs from unknown trissocyclid group A sp 10 cf sp 2 in that it tends to be more heavily silicified, and has different-shaped large pores adjacent to the D-ring. Pores are not perfectly mirrored and appear slightly “stretched”.

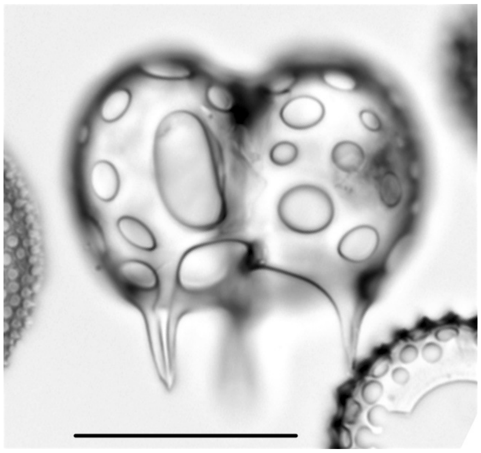

unknown trissocyclid group A sp 37

Trissocyclidae

Undescribed

Records: 17

This species has mis-matched pores on each lobe.

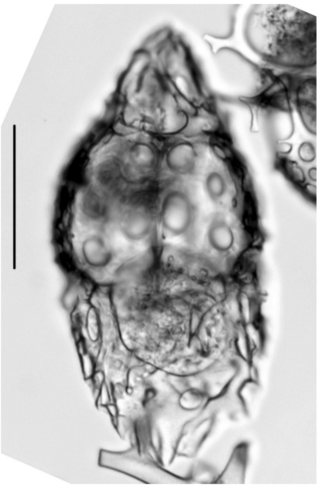

unknown trissocyclid group A sp 38

Trissocyclidae

Undescribed

Records: 21

This species is similar to Tholospyris baconiana variabilis, but has much more massive decorative characters below and above the main shell. The apical horn is a thick triangular feature, and the basal feature is thick, typically-hyaline, and creates a single large oval opening. Also differs from T. baconiana variabilis in that pore son the main shell are not as angular.

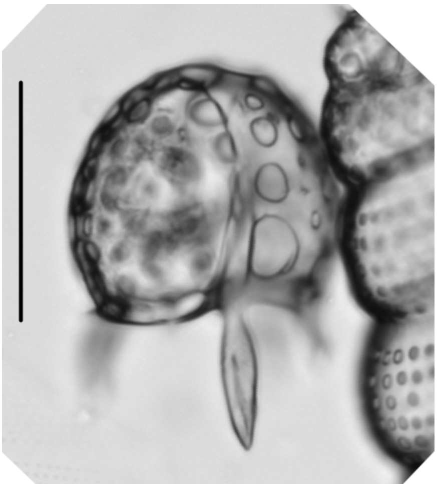

unknown trissocyclid group A sp 39

Trissocyclidae

Undescribed

Records: 7

This species differs from unknown trissocyclid group A sp 33 in that it is more heavily silicified.

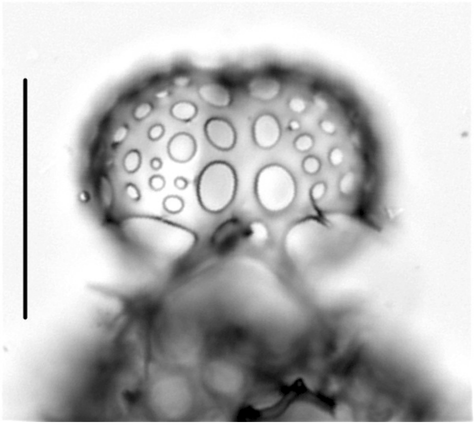

unknown trissocyclid group A sp 40

Trissocyclidae

Undescribed

Records: 47

This species has distinctively large pores at the basal sides of the shell, a small apical horn at a slight indent between lobes, branching feet, and one side of the shell with very large pores and the other side with very small. The widest point is the central part of the lobes.

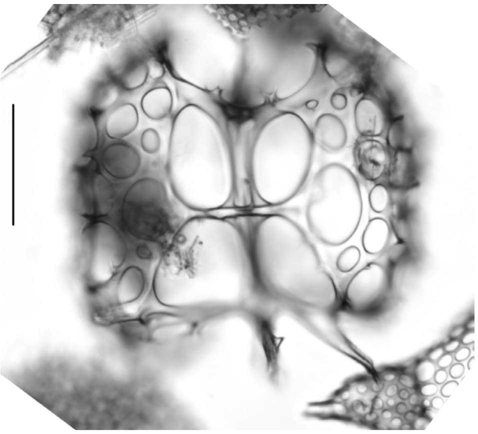

unknown trissocyclid group A sp 41

Trissocyclidae

Undescribed

Records: 46

This species is large, with angular, barbed pores. Different from unknown plagonid group A sp 2 in that is proportionally less wide and more tall.

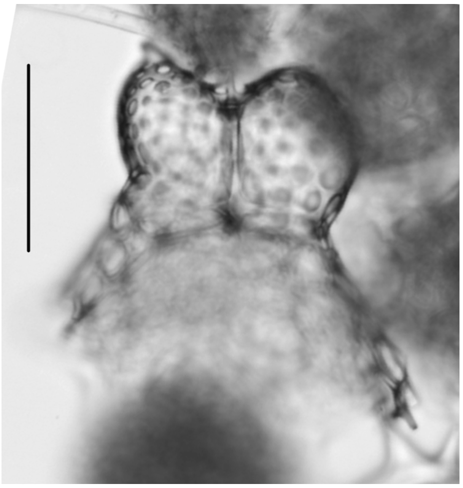

unknown trissocyclid group A sp 42

Trissocyclidae

Undescribed

Records: 12

This species is distinct for its long thorax, which is also wider than the cephalis. Also has two large spines pointing outward from the top of each lobe.

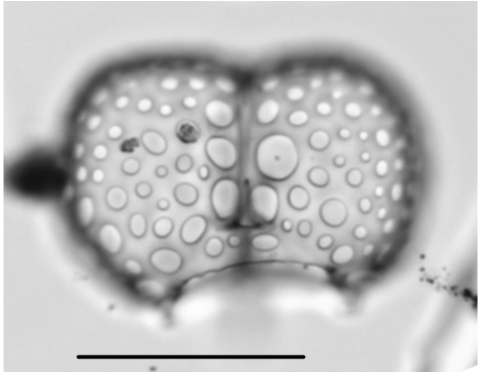

unknown trissocyclid group A sp 43

Trissocyclidae

Undescribed

Records: 47

This species is unique for its small pores, and small protruding flap of silica along the bottom of the front part of the shell, below the row of pores lining the D-ring.

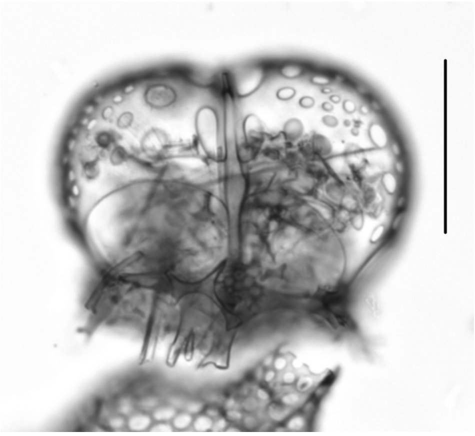

unknown trissocyclid group A sp 44

Trissocyclidae

Undescribed

Records: 27

This species has a smooth shell exterior, with small pores on the lobes, but very narrow pores along the D-ring, and a set of very large openings beside the bottom of the D-ring.

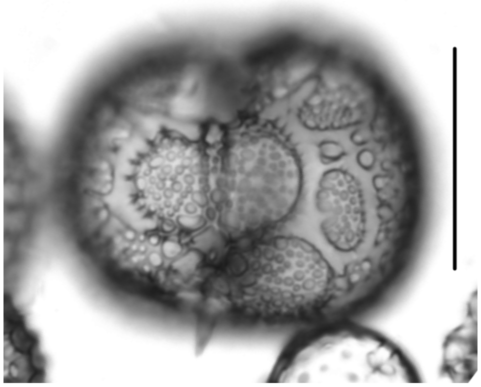

unknown trissocyclid group A sp 45

Trissocyclidae

Undescribed

Records: 9

The exterior bar and lattice pattern of this species resembles unknown trissocyclid group A sp 15 q, but differs in its overall shape. It lacks the extra decorative features above and below the cephalis of the shell.

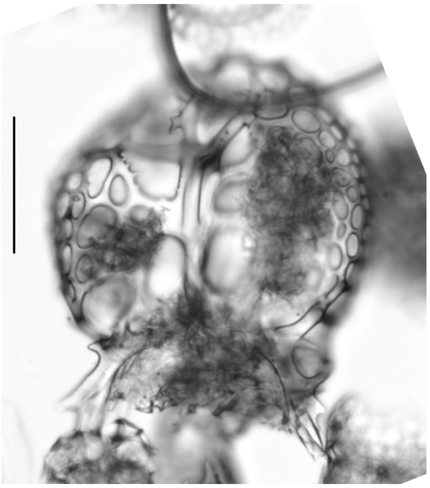

unknown trissocyclid group A sp 46

Trissocyclidae

Undescribed

Records: 14

This species has large, somewhat angular pores, with large, branching feet, and is longer than it is wide.

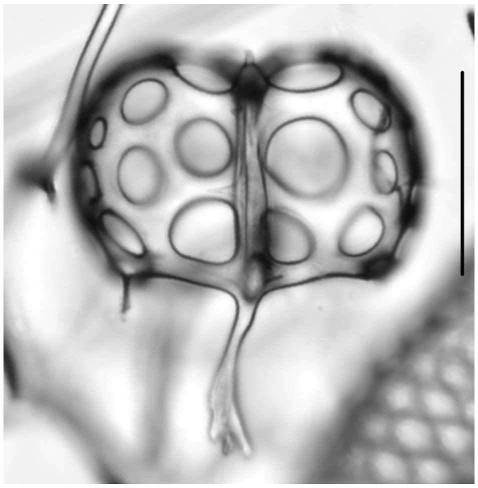

unknown trissocyclid group A sp 47

Trissocyclidae

Undescribed

Records: 13

This species is distinct for having few pores and thick bars, with branching feet. It is about twice as long as it is tall.

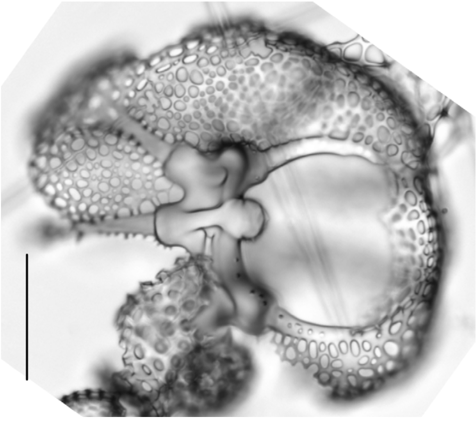

unknown trissocyclid group A sp 48

Trissocyclidae

Undescribed

Records: 9

This species is distinct for its large size, very small and irregular pores, and thick, knobby D-ring.

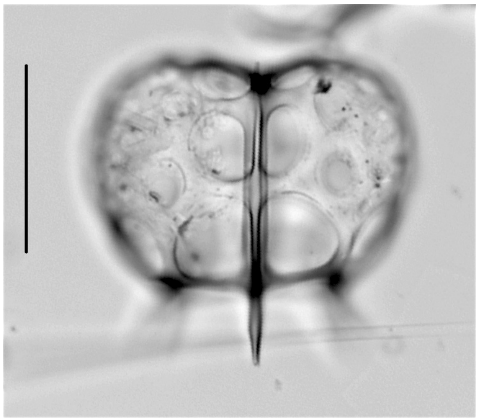

unknown trissocyclid group A sp 49

Trissocyclidae

Undescribed

Records: 25

This species is lightly silicified, small, with three sets of enlarged pores on each side of the D-ring. The tope of the shell is the widest part.

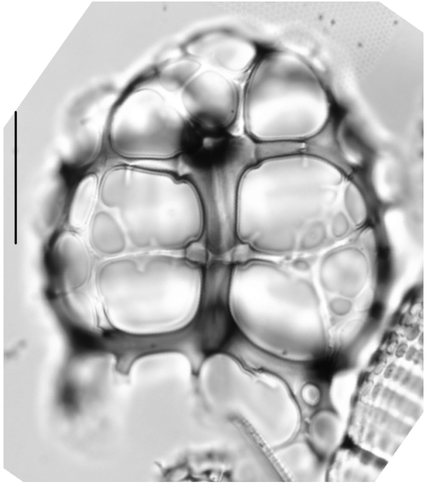

Tholospyris procera

Trissocyclidae

Goll, 1969

Records: 51

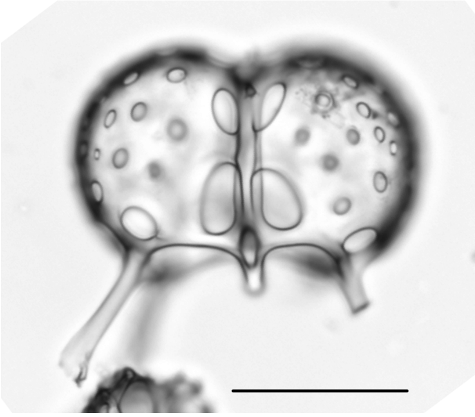

unknown trissocyclid group A sp 51

Trissocyclidae

Undescribed

Records: 11

This species has small round pores, except for the stretched pores around the D-ring. Between the pores there is a lot of hyaline space. Feet are conical and point outwards.

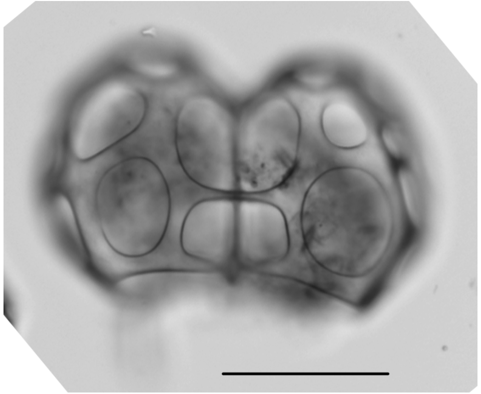

unknown trissocyclid group A sp 52

Trissocyclidae

Undescribed

Records: 18

This species is unique for its relatively large size and very large pores throughout the shell.

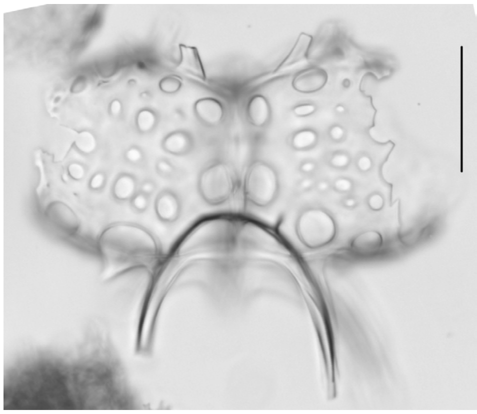

unknown trissocyclid group A sp 53

Trissocyclidae

Undescribed

Records: 10

This species has long, robust feet, and wide-set lobes on the cephalis with strong spines rising from approximately midway across the top of each lobe.

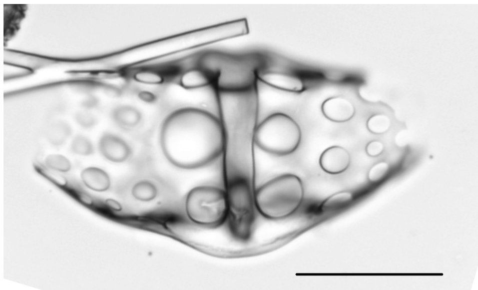

unknown trissocyclid group A sp 54

Trissocyclidae

Undescribed

Records: 4

This species has unique, elongated lobes that do not close at the ends.

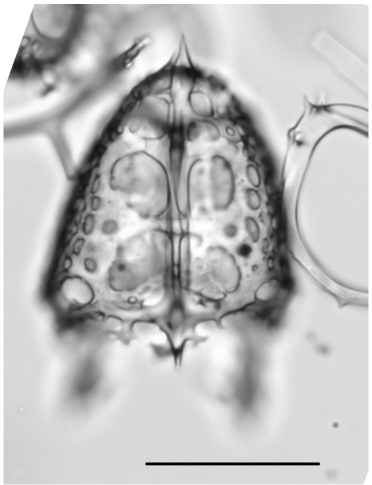

unknown trissocyclid group A sp 55

Trissocyclidae

Undescribed

Records: 17

This species is triangular in outline, robust, with a strong apical spine and feet.

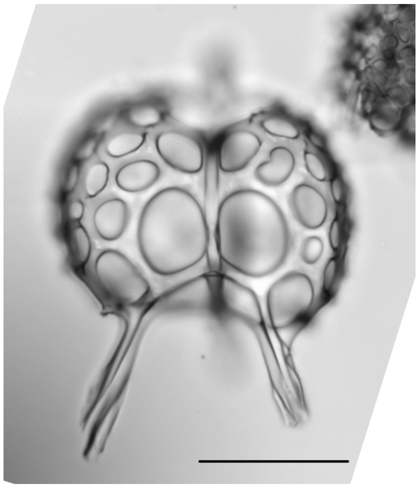

unknown trissocyclid group A sp 56

Trissocyclidae

Undescribed

Records: 15

This species has relatively large pores, with bladed feet and a branching apical spine.

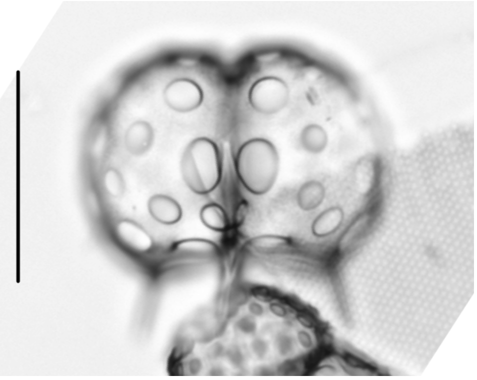

unknown trissocyclid group A sp 57

Trissocyclidae

Undescribed

Records: 14

This species is relatively small, with regular, oval pores.

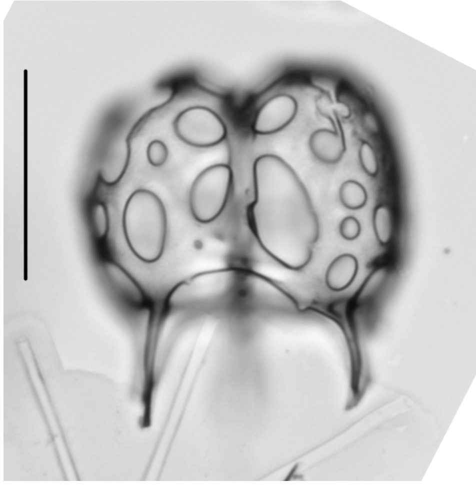

unknown trissocyclid group A sp 58

Trissocyclidae

Undescribed

Records: 11

This species has irregular pores, bladed feet, and has flattened lobes.

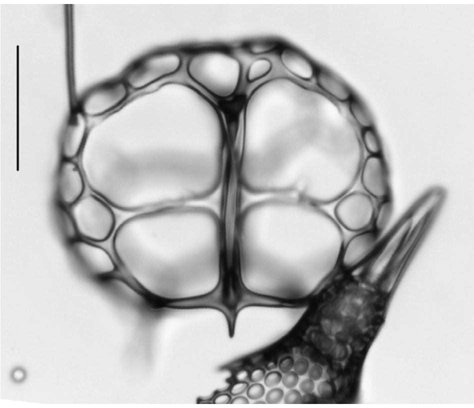

unknown trissocyclid group A sp 58

Trissocyclidae

Undescribed

Records: 6

The general shape of this species resembles Androspyris anthropiscus, but ti differs in that it has sets of four very large pores on either side of the D ring. Pores throughout the shell are also larger than A. anthropiscus.

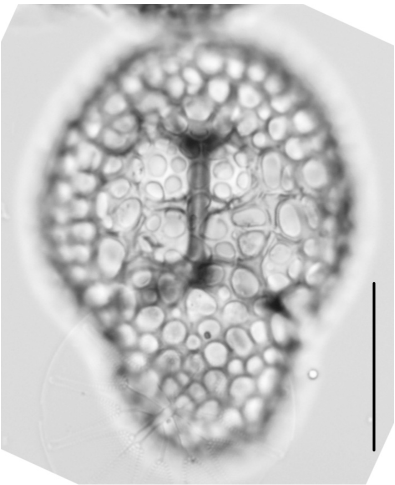

unknown trissocyclid group A sp 6

Trissocyclidae

Undescribed

Records: 22

This species is similar to unknown trissocyclid group A sp 2, but differs in shape outline; the bottom lobe is attached with a clear inflection, separating it from the rest of the shell.

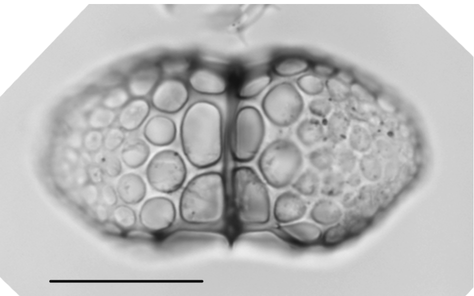

unknown trissocyclid group A sp 8

Trissocyclidae

Undescribed

Records: 60

This species differs from unknown trissocyclid group A sp 31 in that lobes are pointed, rather than flat on the sides. There is also no indentation between the lobes, as in sp 31. Overall pore size and structure are, however, very similar between these two species.

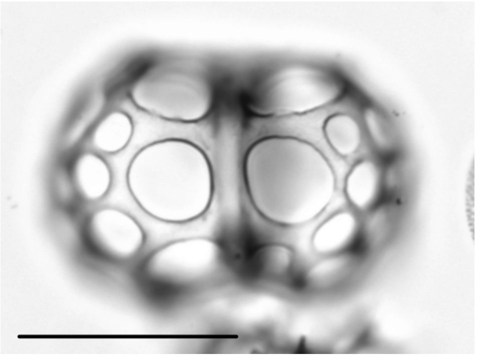

unknown trissocyclid group B sp 16

Trissocyclidae

Undescribed

Records: 49

This species has large pores around the D-ring, which reduce to about half-size at the end of each lobe. It lacks spines and has a generally smooth exterior.

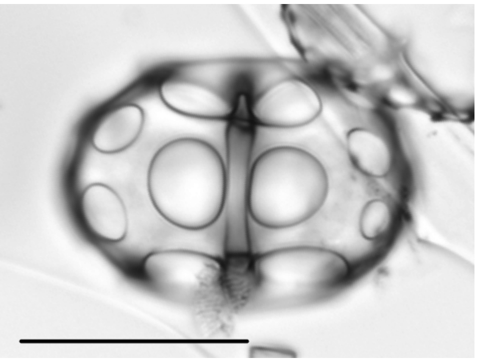

unknown trissocyclid group B sp 23

Trissocyclidae

Undescribed

Records: 21

This species resembles unknown trissocyclid group B sp 16, but has larger pores at the ends-points of each lobe.

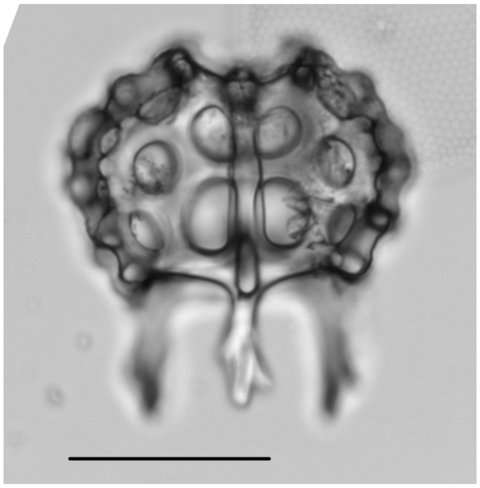

unknown trissocyclid group B sp 26

Trissocyclidae

Undescribed

Records: 10

This species has strong, forked feet, and a knobby shell exterior.

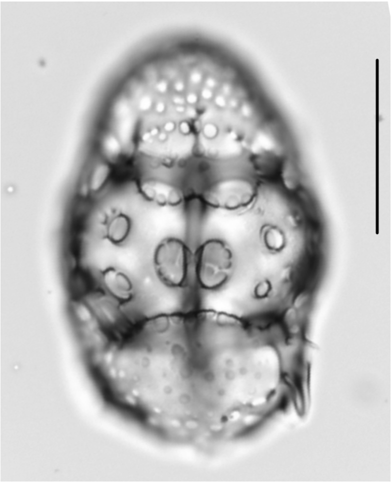

unknown trissocyclid group B sp 27

Trissocyclidae

Undescribed

Records: 9

This species has few, relatively small pores, and finely-latticed, delicate lobes above and below the main shell.

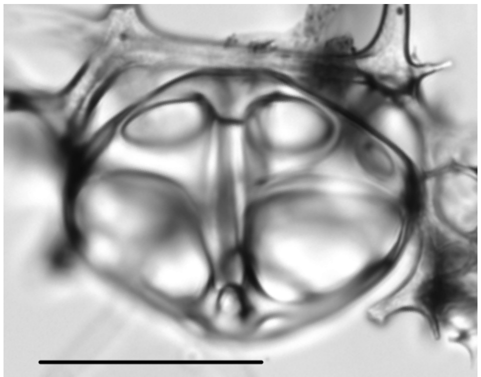

unknown trissocyclid group B sp 28

Trissocyclidae

Undescribed

Records: 31

This species has a distinct curved overall shape, with a smooth exterior and few large pores.

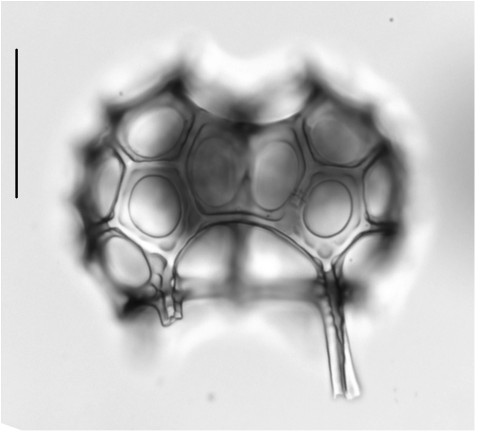

unknown trissocyclid group B sp 30

Trissocyclidae

Undescribed

Records: 18

This species resembles T. baconiana variabilis, but has pores of similar size throughout the shell, rather than small pores at the ends of each lobe.

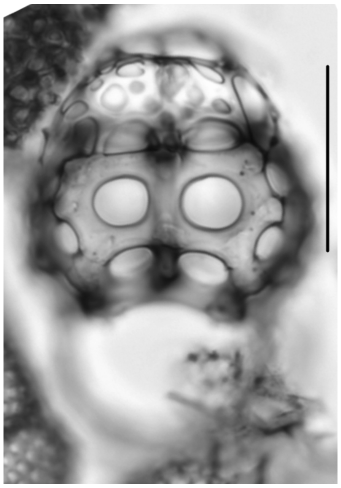

unknown trissocyclid group B sp 31

Trissocyclidae

Undescribed

Records: 83

This species resembles T. binoctonum, but has an additional latticed veil feature on the upper part of the shell, and a robust, large-pored feature around the bottom of the shell.

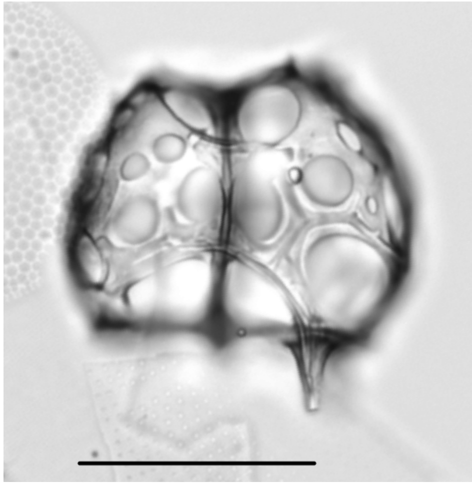

unknown trissocyclid group B sp 5

Trissocyclidae

Undescribed

Records: 22

This species is distinct for its set of small pores on the front of the shell.

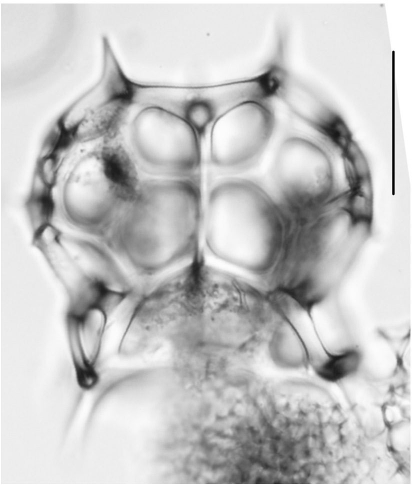

unknown trissocyclid group B sp 7

Trissocyclidae

Undescribed

Records: 21

This species resembles L. pentagona, but differs in that the bottom of the shell has a continuation of the same angular latticed pattern on the rest of the shell, rather than bladed feet.

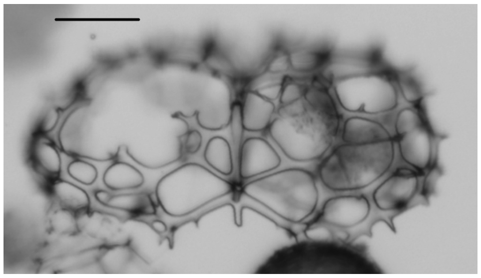

unknown trissocyclid group C sp 2

Trissocyclidae

Undescribed

Records: 9

This species is relatively large, with wide lobes extending from the D-ring. It also has distinct, barbed angular pores.

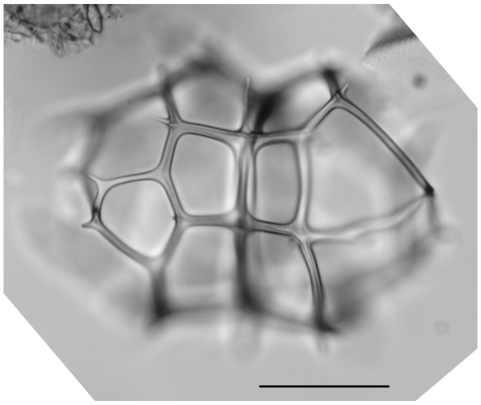

unknown trissocyclid group C sp 3

Trissocyclidae

Undescribed

Records: 14

This species is angular in outline, small, with large angular pores. Differs from L. pentagona in lacking bladed bars.

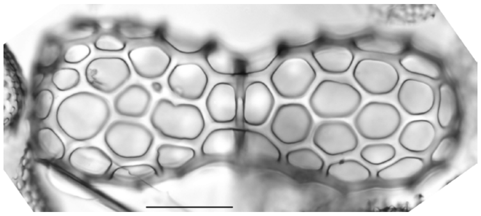

unknown trissocyclid group C sp 4

Trissocyclidae

Undescribed

Records: 42

This species resembles unknown plagonid group A sp 2, but lacks barbs on its pores.

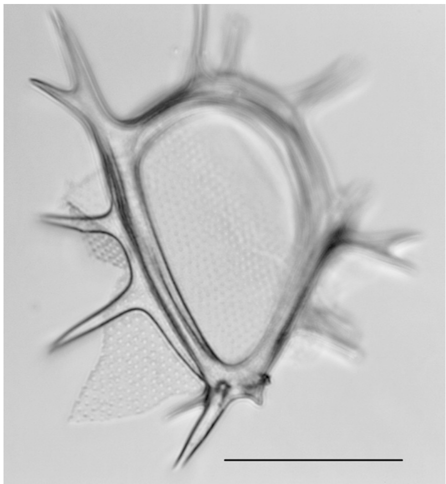

Zygocircus piscicaudatus

Trissocyclidae (stephanid)

Popofsky, 1913

Records: 9

sensu Goll, 1979

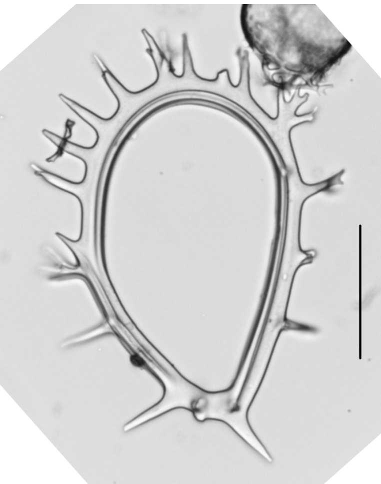

Zygocircus productus

Trissocyclidae (stephanid)

(Hertwig) Haeckel, 1887

Records: 36

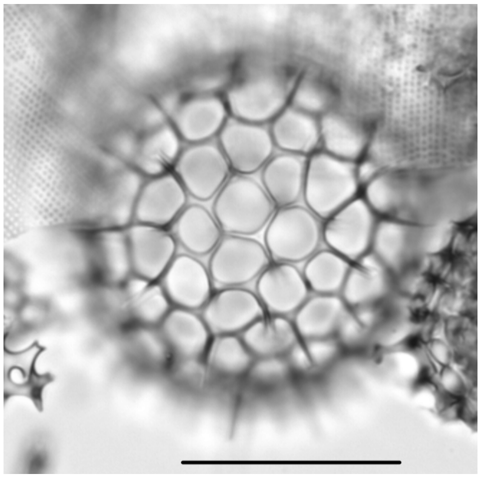

Acanthosphaera actinota

Actinommidae

Haeckel, 1860

Records: 28

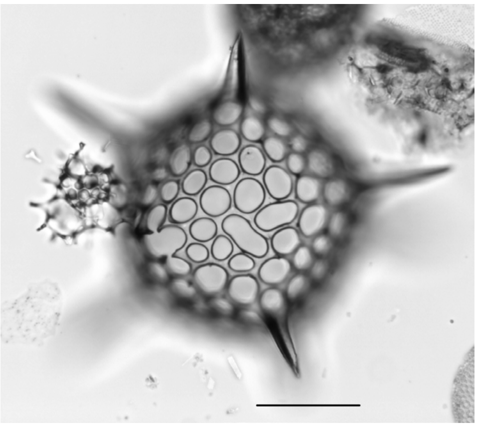

Acanthosphaera actinota cf

Actinommidae

Undescribed

Records: 50

This species differs from A. actinota in having rounder, more irregular pores, overall larger size, and long, bladed rather than short, conical spines. Overall outline of shell is typically lumpy, not perfectly round.

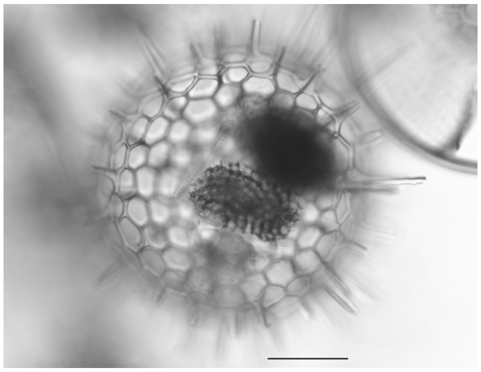

Acanthosphaera insignis

Actinommidae

(Hertwig) Popofsky, 1912

Records: 9

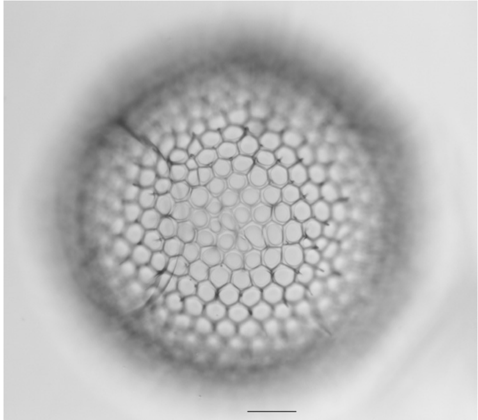

Acanthosphaera tunis

Actinommidae

(Haeckel) Takahashi, 1991

Records: 26

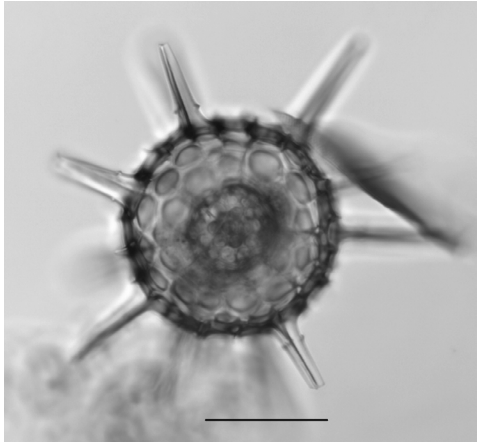

Actinomma boreale

Actinommidae

Cleve, 1899

Records: 86

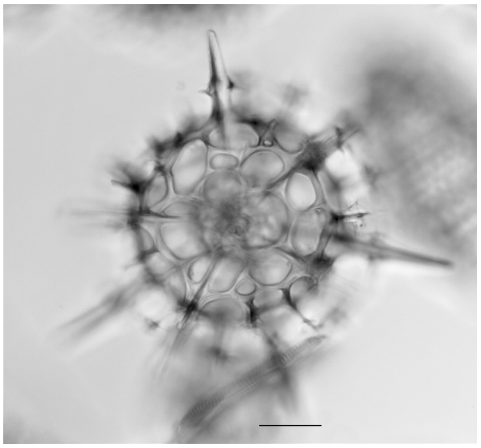

Actinomma boreale cf

Actinommidae

Undescribed

Records: 39

This species is similar to Actinommidae boreale, but differs in consistently having larger, more angular pores on the cortical shell.

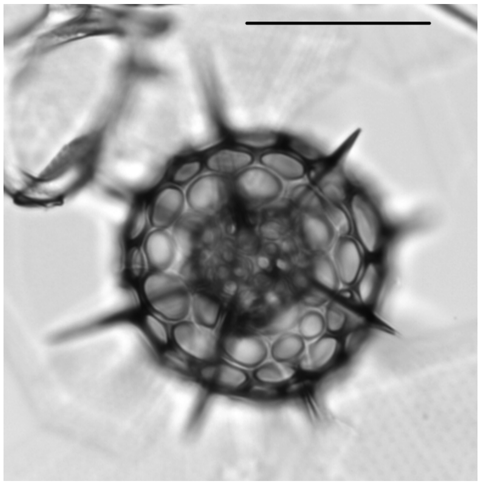

Actinomma leptodermum

Actinommidae

Jørgensen, 1900

Records: 103

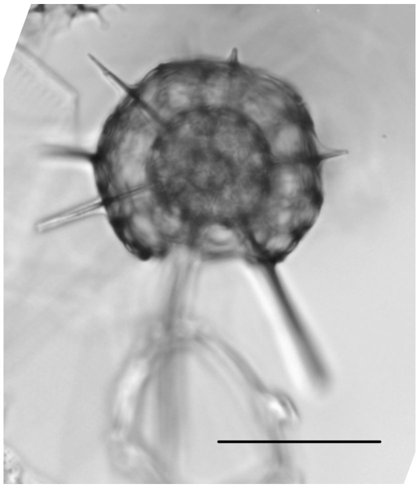

Actinomma leptoderma longispina

Actinommidae

Cortese & Bjørklund, 1998

Records: 22

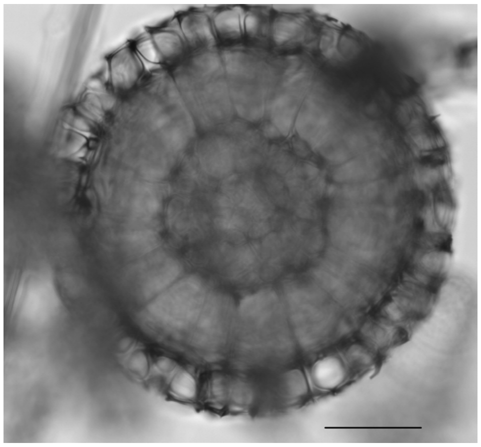

Actinomma antarcticum cf

Actinommidae

Undescribed

Records: 89

Similar, but not identical to the species described by Haeckel, 1887

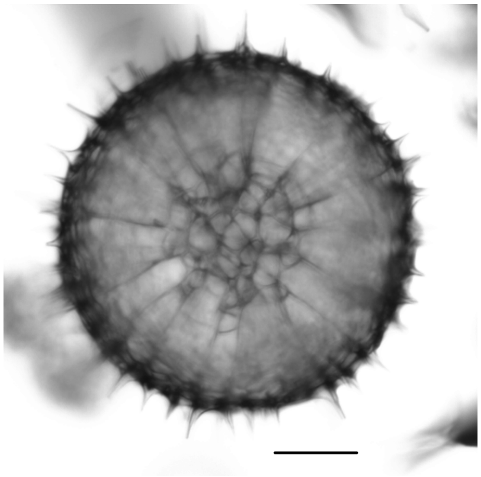

Actinomma medianum cf

Actinommidae

Undescribed

Records: 25

Differs from A. medianum in that is has a medullary shell without a regular shape, just a network of spines.

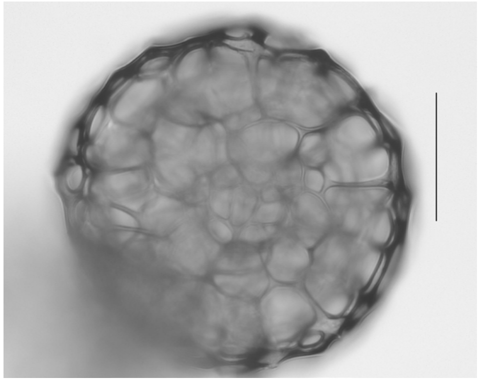

Actinomma medianum cf sp 2

Actinommidae

Undescribed

Records: 27

This species differs from Actinomma medianum in that the cortical shell is significantly smaller, and the medullary shells fill a relatively large portion of the shell's interior.

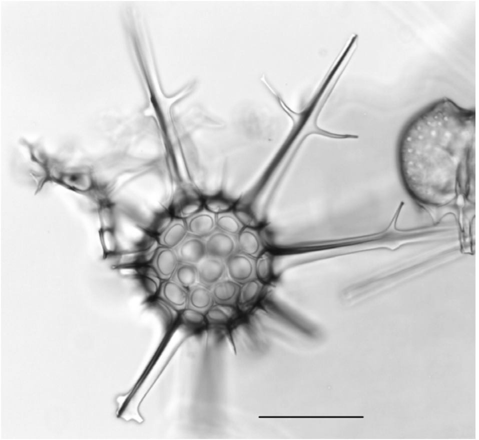

Anomalacantha dentata

Actinommidae

Mast, 1910

Records: 20

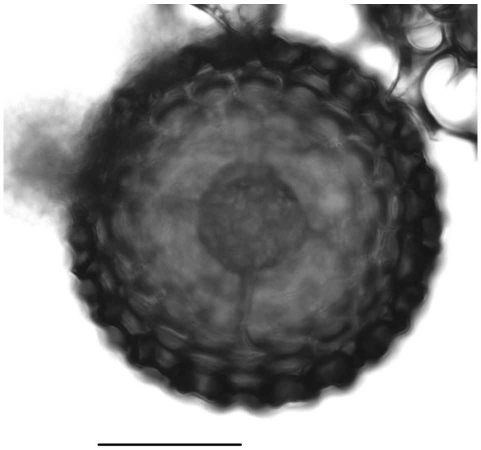

Carposphaera raiai

Actinommidae

Blueford, 1982

Records: 13

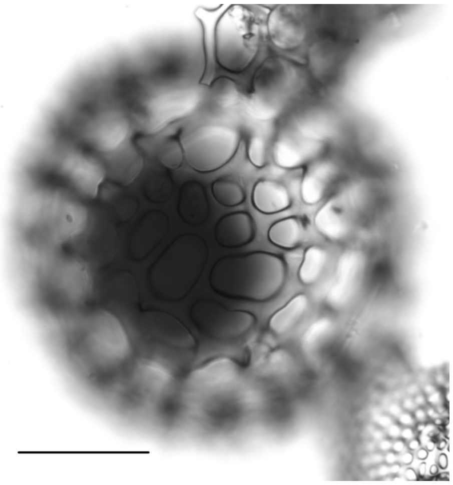

Carposphaera acanthophora
Actinommidae
(Popofsky) Benson, 1966
Records: 5

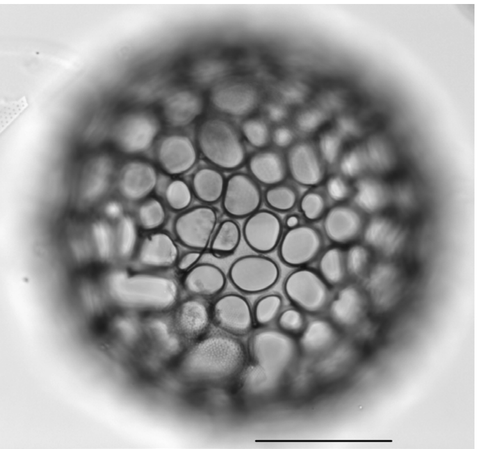

Carposphaera annikae
Actinommidae
Renaudie & Lazarus, 2012
Records: 27

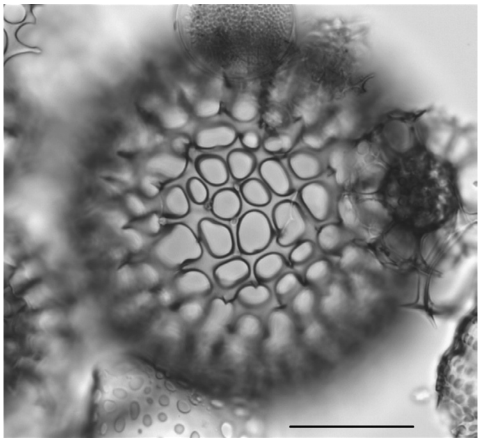

Carposphaera annikae cf
Actinommidae
Undescribed
Records: 7

This species differs from Carposphaera annikae in that the cortical shell is thicker and the barbs on the pores are more robust.

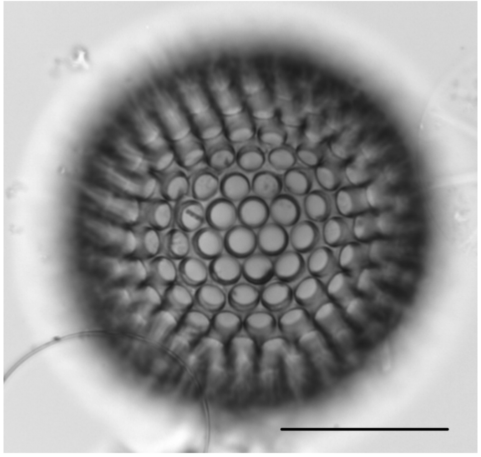

Cenosphaera sp 2
Actinommidae
Undescribed
Records: 41

This species is distinct for its round pores, relatively thin shell, and lack of spines and medullary shell.

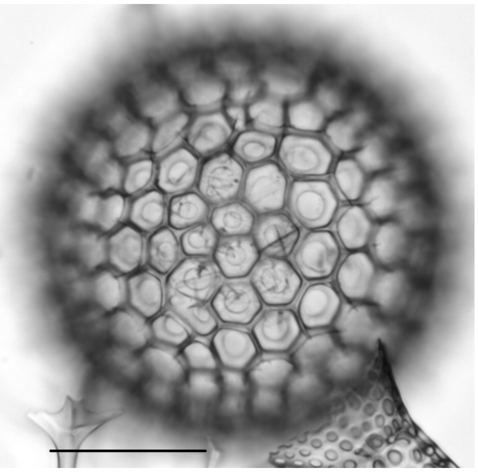

Cenosphaera sp B Kamikuri
Actinommidae
Kamikuri, 2017
Records: 31

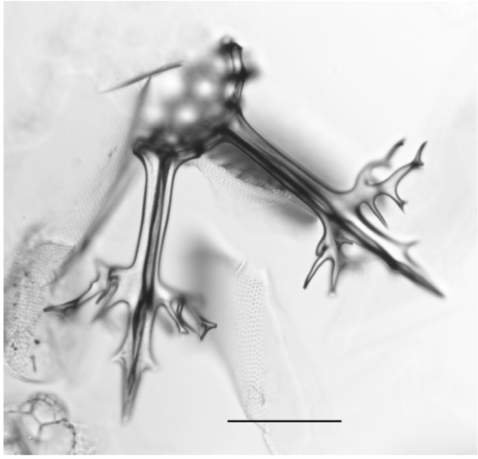

Cladococcus abietinus
Actinommidae
Haeckel, 1887
Records: 23

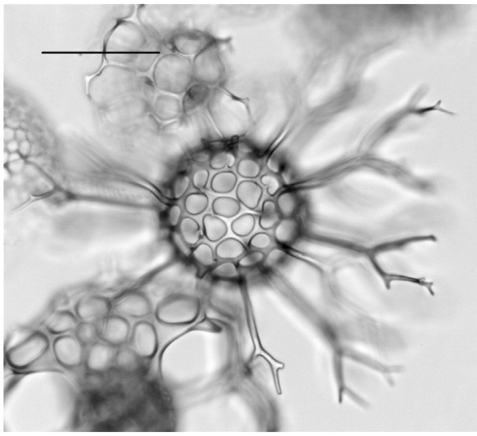

Cladococcus cervicornis
Actinommidae
Haeckel, 1860
Records: 28

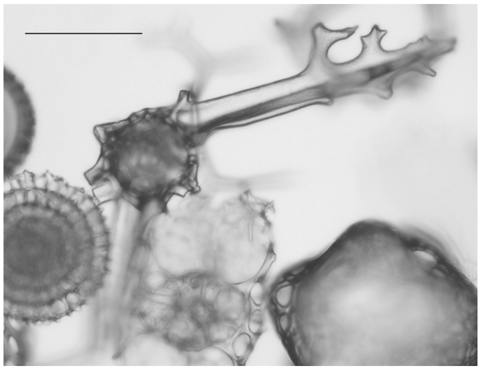

Cladococcus pinetim
Actinommidae
Haeckel, 1887
Records: 11

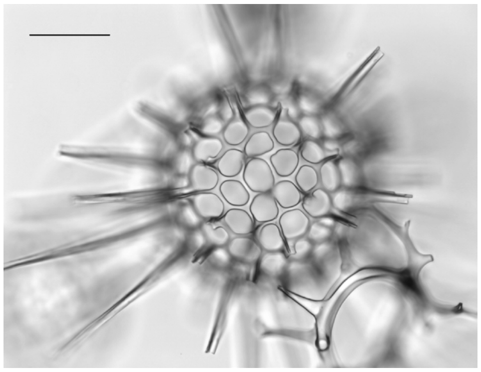

Cladococcus sp 1
Actinommidae
Undescribed
Records: 43

This species has small, sub-angular pores, and thin bladed spines. Spines lack serrations and branching, differentiating it from the other Cladococcus species.

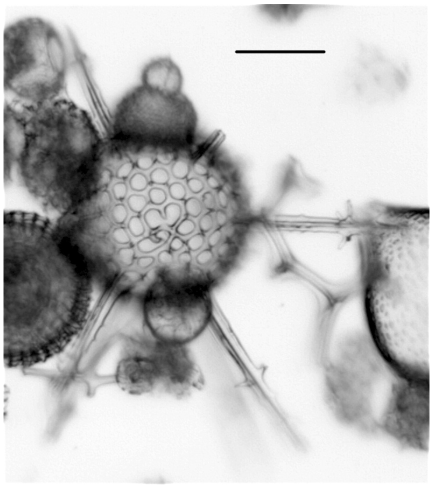

Cladococcus sp 2
Actinommidae
Undescribed
Records: 29

This species has thick, bladed spines that branch, as well as small, needle-like conical spines. Overall size is relatively large.

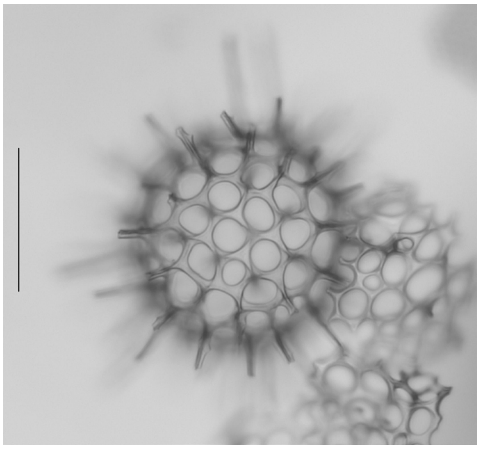

Cladococcus viminalis
Actinommidae
Haeckel, 1862
Records: 11

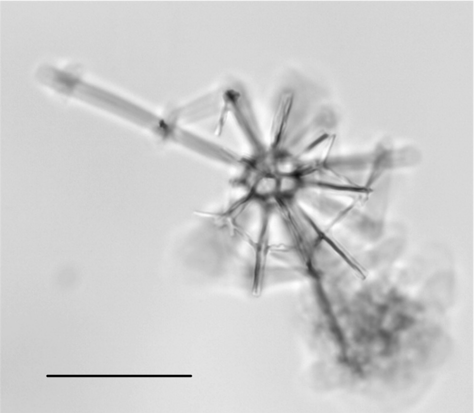

Drymosphaera pseudosagenoscena
Actinommidae
Sugiyama, 1992
Records: 19

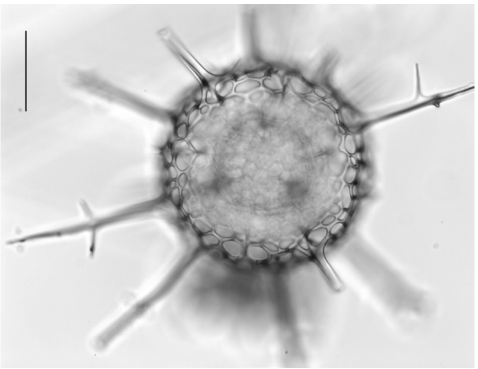

Haeckeliella macrodoras

Actinommidae

(Haeckel) Hollande & Enjumet, 1960

Records: 17

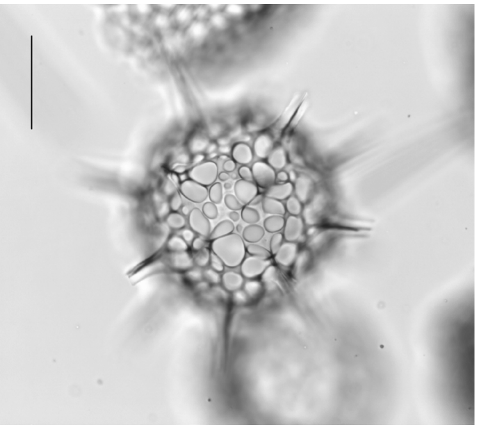

Haeckeliella macrodoras cf

Actinommidae

Undescribed

Records: 47

This species has a similar texture to H. macrodoras on the cortical shell, but is overall smaller and lacks a medullary shell.

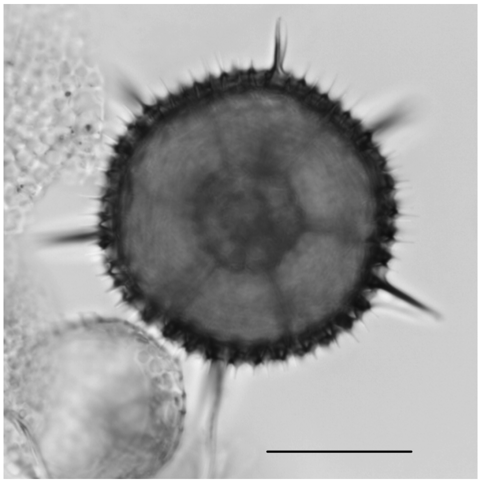

Haliommetta miocenica

Actinommidae

Campbell & Clark, 1944

Records: 178

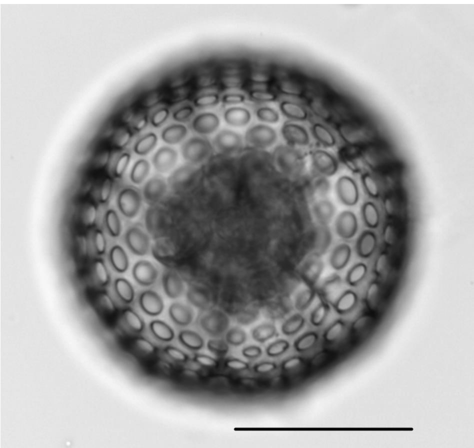

Haliommetta miocenica cf

Actinommidae

Undescribed

Records: 103

This species is like Haliometta miocenica, but differs in that it has a relatively more lightly silicified cortical shell, and tends to have muted external spines, if they are present at all.

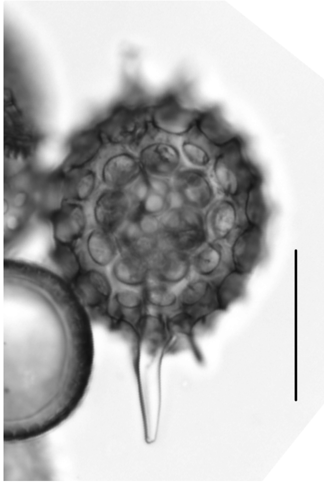

Heliosoma (Heliosomantha) alveolalum

Actinommidae

Campbell & Clark, 1944

Records: 39

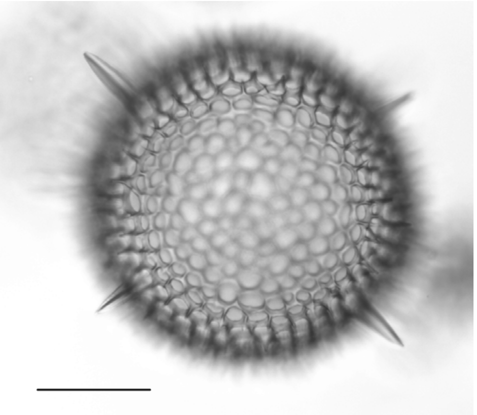

Heliosphaera radiata

Actinommidae

Popofsky, 1912

Records: 53

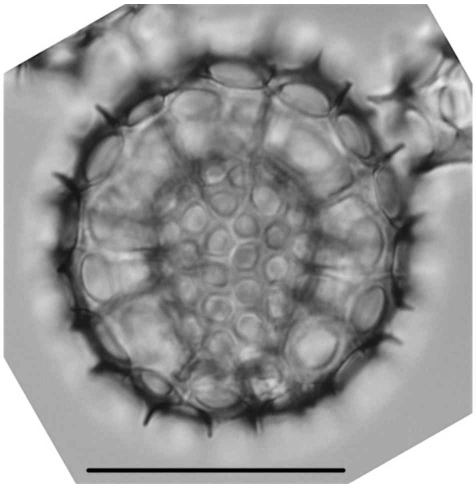

Helisoma dispar
Actinommidae
Blueford, 1982
Records: 11

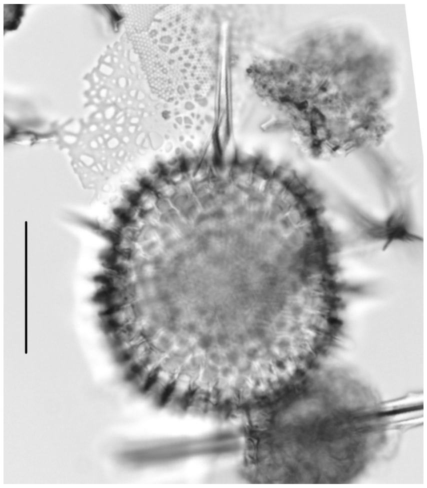

Helisoma dispar cf sp 1
Actinommidae
Undescribed
Records: 33

This species is lumpy and oval-shaped, with smaller pores and thick cortical shell. Two faint medullary shells are visible in most specimens.

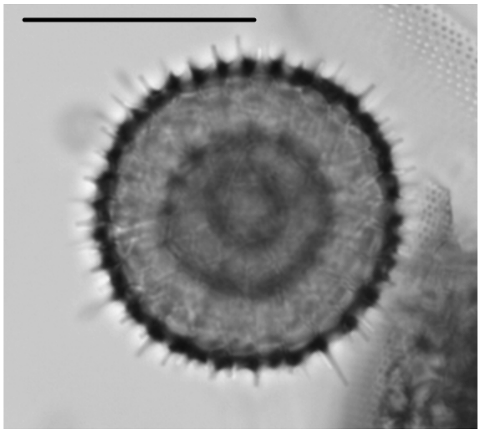

Liosphaera sp 1
Actinommidae
Undescribed
Records: 18

This species belongs to Liosphaera, but does not match any described species. Medullary shells are relatively large, cortical shell has spiny, irregular, small pores.

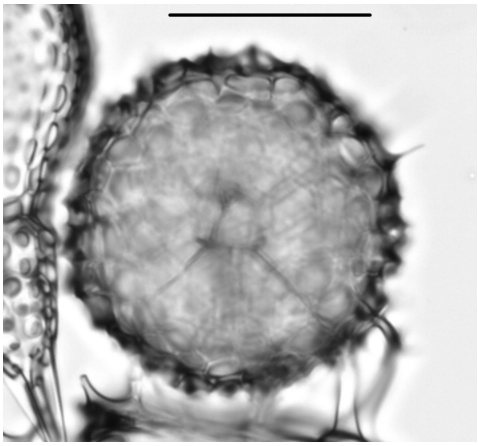

Lonchosphaera mariae
Actinommidae
Dumitrica, 2014
Records: 6

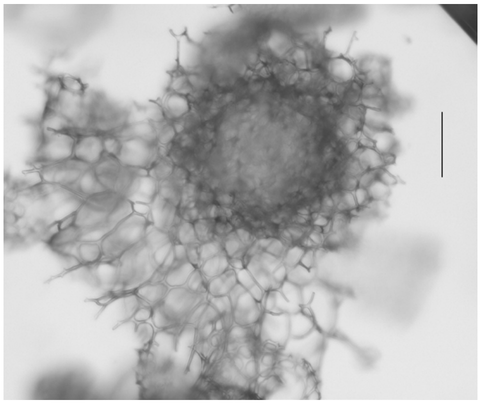

Octodendron nidum (?)
Actinommidae
Tan & Tchang, 1976
Records: 46

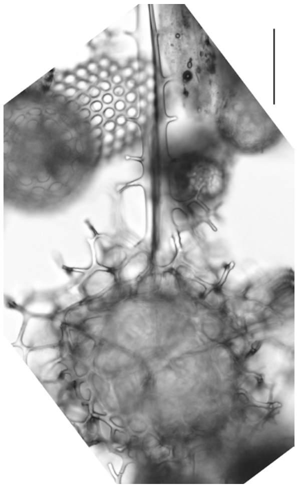

Rhizoplegma? sp 1
Actinommidae
Undescribed
Records: 33

This species resembles Rhizoplegma boreale (Popofsky, 1908), but is significantly larger than the described species.

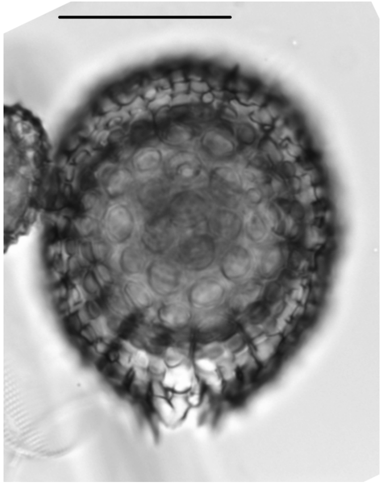

Sphaeropyle langii
Actinommidae
Dreyer, 1889
Records: 55

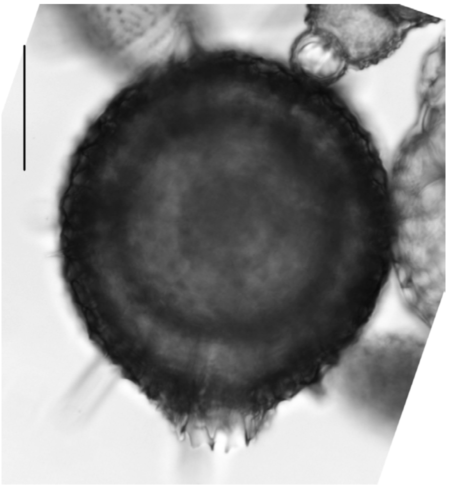

Sphaeropyle robusta
Actinommidae
Kling, 1973
Records: 35

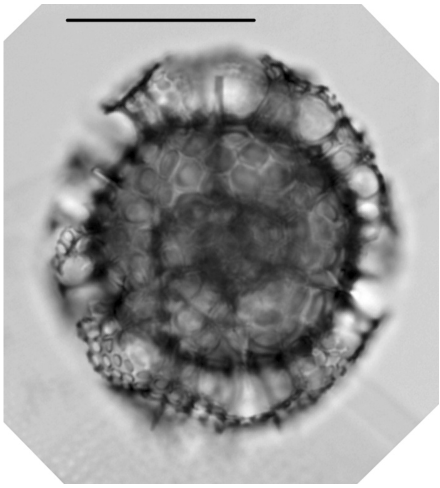

Sphaeropyle robusta cf
Actinommidae
Undescribed
Records: 45

This species differs in that the inner medullary shell is oval-shaped rather than spherical. The cortical shell is also less robust.

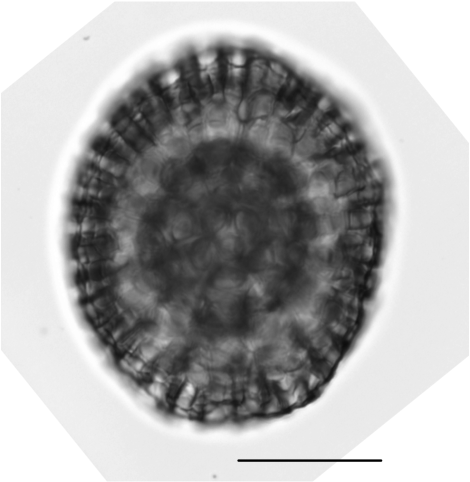

Sphaeropyle sp 5
Actinommidae
Undescribed
Records: 24

This species differs from Sphaeropyle robusta and Sphaeropyle langii in that its medullary shell is significantly smaller relative to the cortical shell.

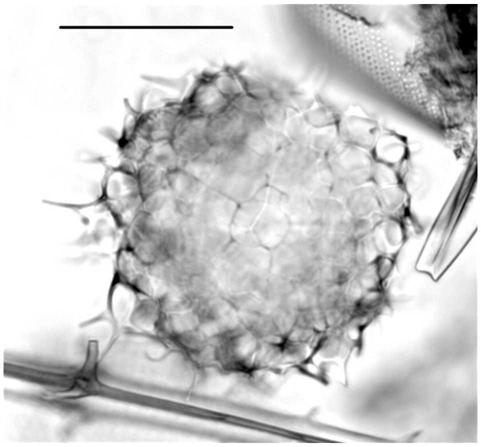

Spongosphaera streptacantha ?
Actinommidae
Haeckel, 1860
Records: 19

This seems to match the species described by Haeckel, except that the rare specimens lacked massive bladed spines, or only exhibited broken spine bases.

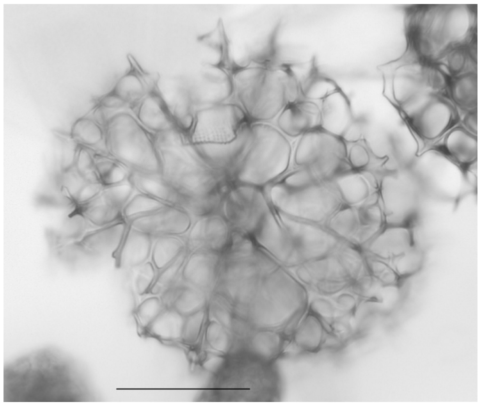

Styptosphaera sp. A (Takahashi)
Actinommidae
Takahashi, 1991
Records: 20

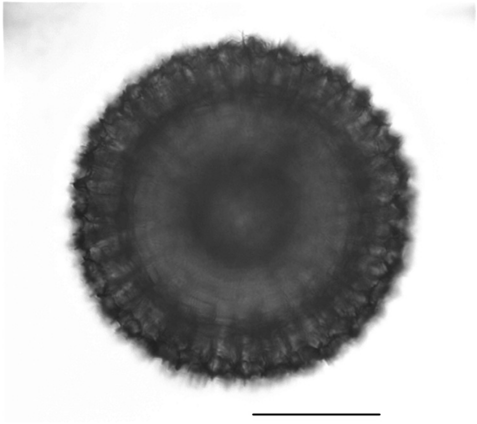

Thecosphaera japonica

Actinommidae

Nakaseko, 1971

Records: 18

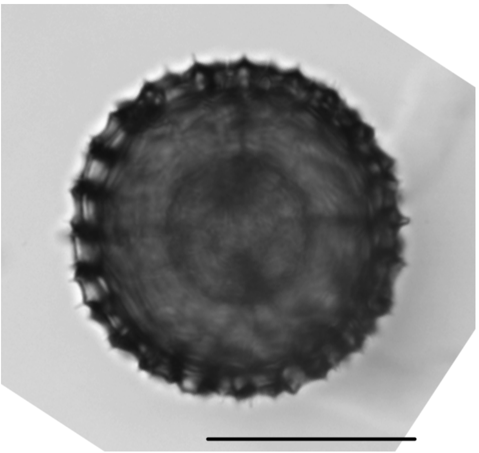

Thecosphaera pseudojaponica

Actinommidae

Nakaseko, 1971

Records: 11

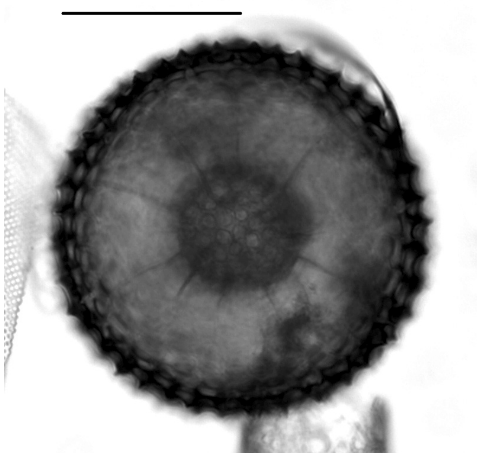

Thecosphaera sp 1

Actinommidae

Undescribed

Records: 17

This species differs from Thecosphera sp 2 in that it has a spiny exterior, and it differs from Thecosphera sp 3 in that it has a well-developed double medullary shell.

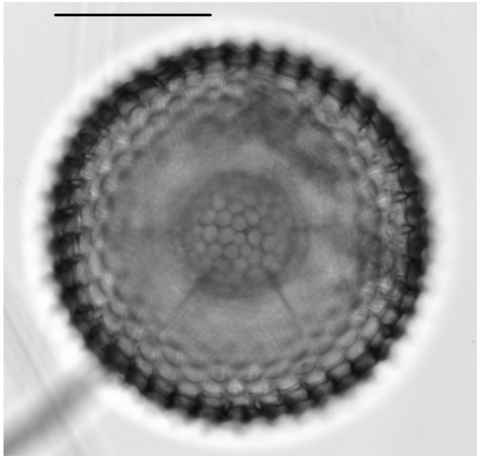

Thecosphaera sp 2

Actinommidae

Undescribed

Records: 99

This species has a relatively smooth exterior, and straight bars connecting the double medullary shell to the cortical shell, which differentiates this species from Thecosphaera sp 1.

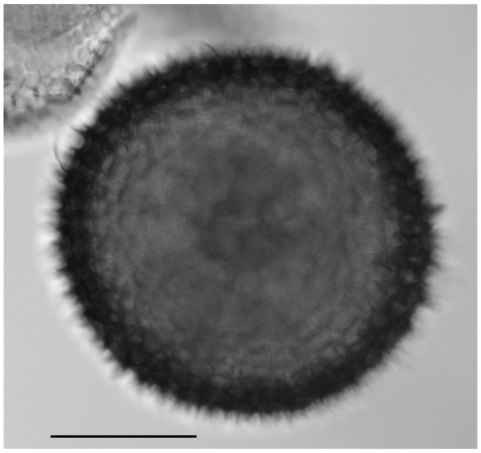

Thecosphaera sp 3

Actinommidae

Undescribed

Records: 8

This species has irregular pores, poorly-developed medullary shell, and a thorny exterior, differentiating this species from Thecosphera sp 1 and sp 2.

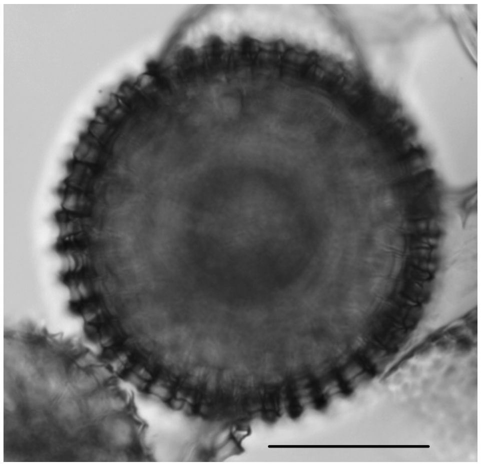

Thecosphaera sp A Kamikuri

Actinommidae

Kamikuri, 2017

Records: 28

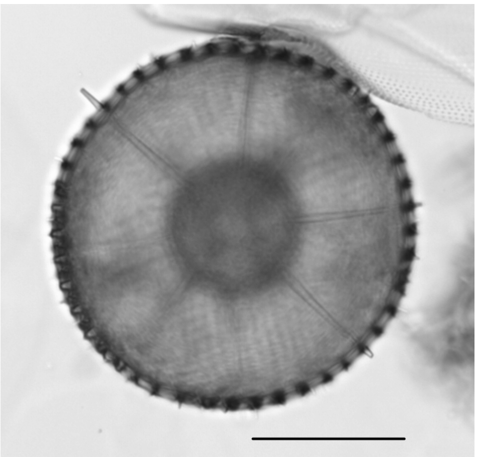

Thecosphaera sp D Kamikuri

Actinommidae

Kamikuri, 2017

Records: 6

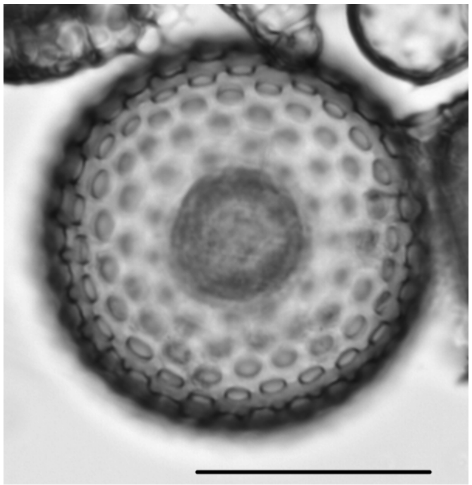

Thecosphaera sp E Kamikuri

Actinommidae

Kamikuri, 2017

Records: 27

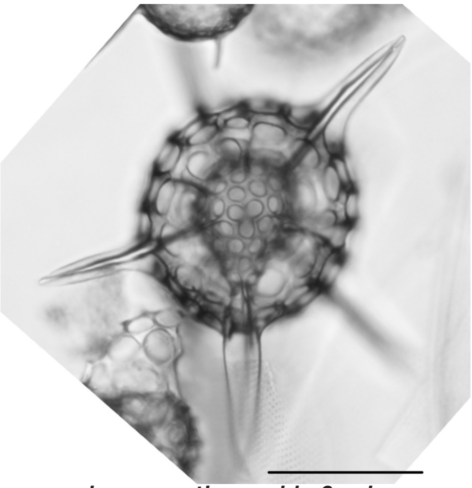

unknown actinommid >6 spines (Druppatractus?) sp 1

Actinommidae

Undescribed

Records: 12

This species has a pear-shaped medullary shell, similar to Druppatractus species. However, is has more than six thick, bladed spines differentiating it from the other species.

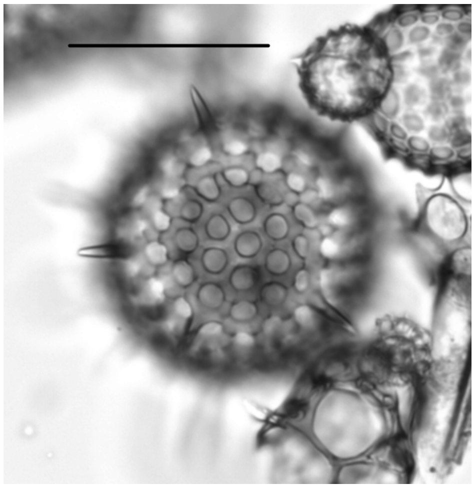

unknown actinommid >6 spines sp 11

Actinommidae

Undescribed

Records: 8

This species has a rough exterior, small, framed pores, and many short, conical spines. Two medullary shells.

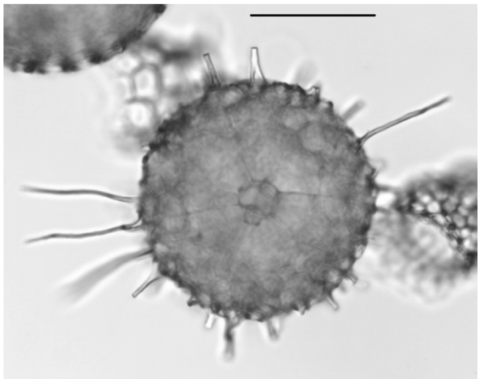

unknown actinommid >6 spines sp 12

Actinommidae

Undescribed

Records: 24

This species is unique for its spongy exterior, crooked, conical spines, and a small, irregular-ly-shaped medullary shell.

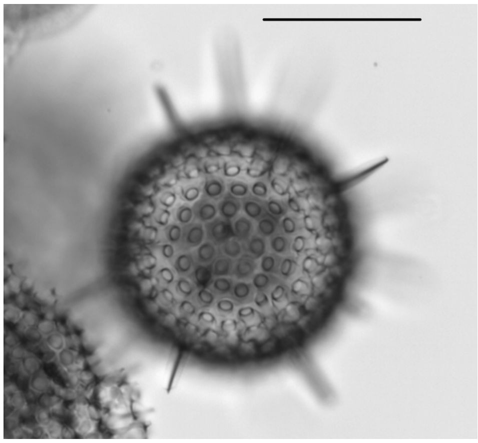

unknown actinommid >6 spines sp 13

Actinommidae

Undescribed

Records: 41

This species resembles unknown actinommid >6 spines sp 11, but differs int hat it has bladed spines rather than conical, relatively smaller pores, and slightly larger overall size.

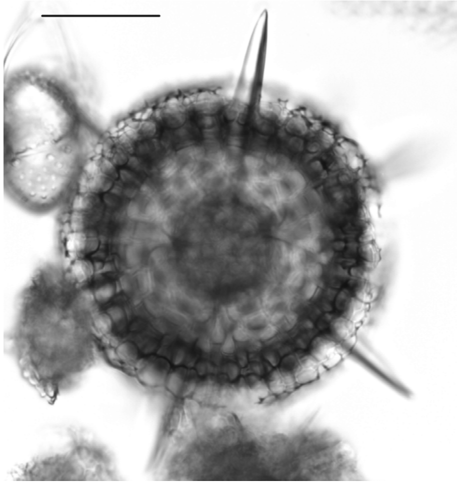

unknown actinommid >6 spines sp 14

Actinommidae

Undescribed

Records: 74

This species differs from unknown actinommid >6 spines sp 13 in that it has fewer spines, and has a thicker cortical shell with significantly larger pores. Some specimens have an extra fine veil surrounding the cortical shell.

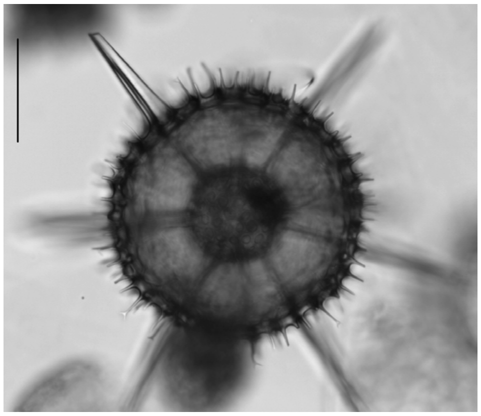

unknown actinommid >6 spines sp 15

Actinommidae

Undescribed

Records: 6

This species is large, with framed, sub-angular pores, with small conical spines coming from each junction of pore angles. It has approximately twelve long, bladed spines and two relatively small medullary shells.

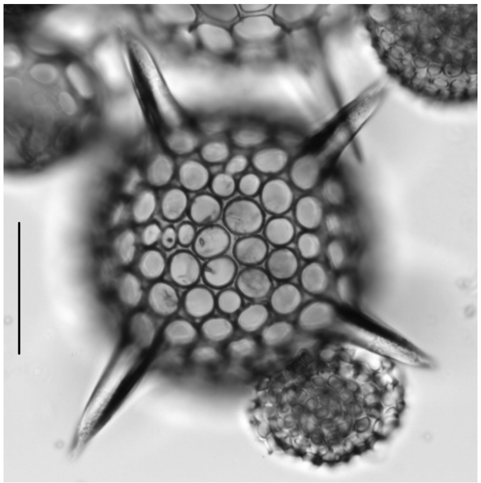

unknown actinommid >6 spines sp 16

Actinommidae

Undescribed

Records: 25

This species is similar to Acanthosphaera actinota cf, but regularly has a more rounded shape to its cortical shell.

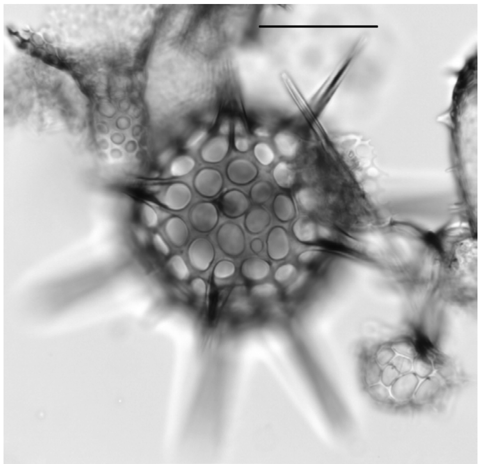

unknown actinommid >6 spines sp 17

Actinommidae

Undescribed

Records: 18

This species has many features in common with unknown actinommid >6 spines sp 14, but differs in that the cortical shell is significantly thinner-walled, with slightly larger pores. Pores tend to be slightly enlarged at the base of each bladed spine.

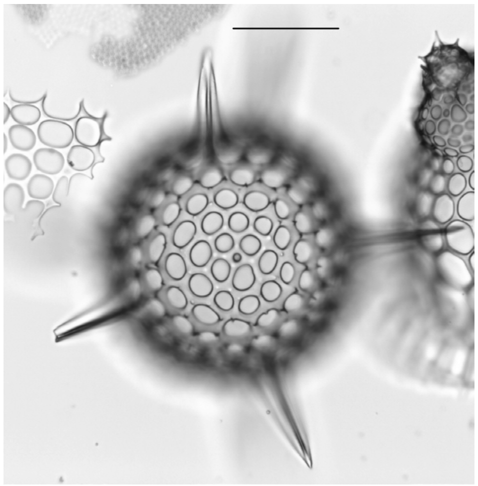

unknown actinommid >6 spines sp 18

Actinommidae

Undescribed

Records: 68

This species resembles Acanthosphera actinota cf, but has relatively smaller pores. It differs from unknown actinommid >6 spines sp 16 in that it has a thicker cortical shell.

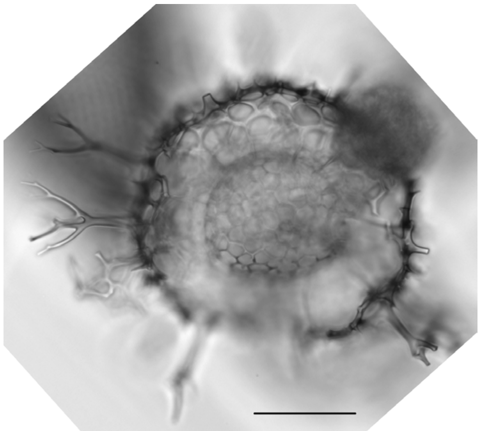

unknown actinommid >6 spines sp 19

Actinommidae

Undescribed

Records: 10

This species has a lightly-silicified cortical shell, but is most distinct for its oval-shaped medullary shell, and conical, dendritic spines. This species may belong to Heliodiscidae, rather than Actinommidae, but the single specimen found is inconclusive.

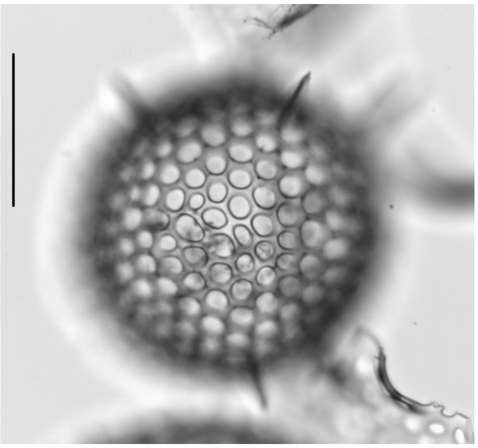

unknown actinommid >6 spines sp 20

Actinommidae

Undescribed

Records: 9

This species is like Heliosphaera radiata, but differs in that it has framed pores rather than smooth ones.

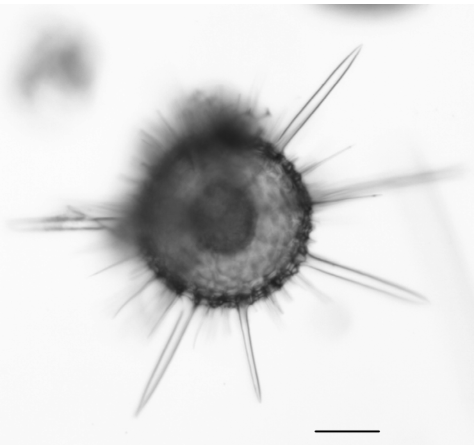

unknown actinommid >6 spines sp 21

Actinommidae

Undescribed

Records: 13

This species is unique for its semi-flattened cortical shell and unusually-long conical spines with pointed tips. This species may belong to Heliodiscidae, but the single specimen observed does not have a clearly eccentric inner medullary shell. It is also very large.

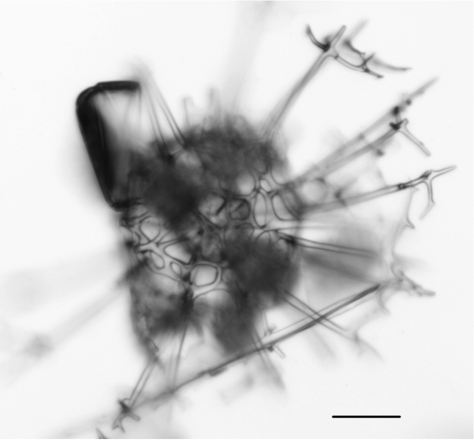

unknown actinommid >6 spines sp 22

Actinommidae

Undescribed

Records: 16

This species is defined by its large size and very large, rough, angular pores, and long branching spines. Only one specimen was found, and eccentric inner medullary shell suggests it could be a heliodiscid.

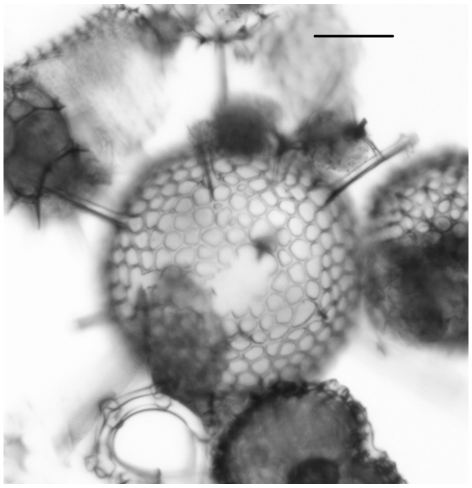

unknown actinommid >6 spines sp 23

Actinommidae

Undescribed

Records: 7

This species has thin, bladed, branching spines, relatively small pores, and no evidence of a medullary shell, differentiating it from unknown actinommid >6 spines sp 21 and unknown actinommid >6 spines sp 22.

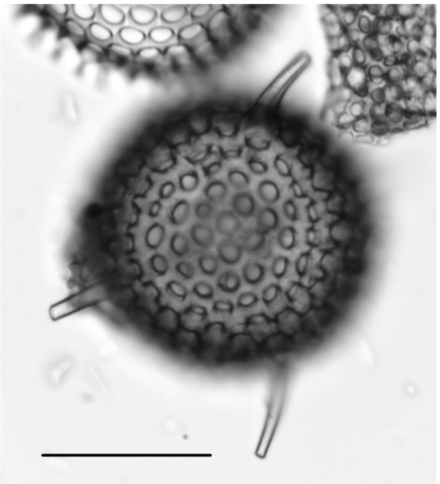

unknown actinommid >6 spines sp 24

Actinommidae

Undescribed

Records: 6

This species is unique for its approximately six curved, thin, conical spines, robust, rugged cortical shell, and small, irregular medullary shell.

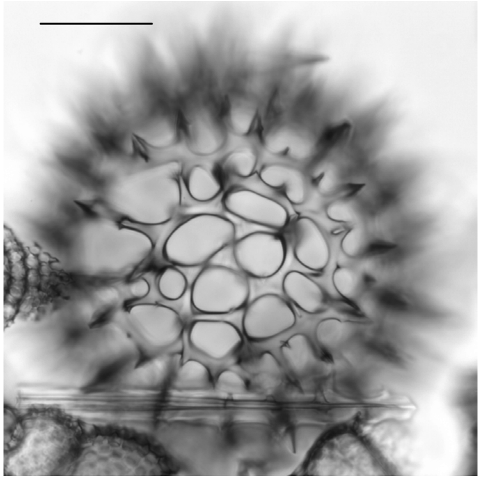

unknown actinommid >6 spines sp 25

Actinommidae

Undescribed

Records: 11

This species has large, angular, irregular pores, with short, thick spines at and between angular junctions.

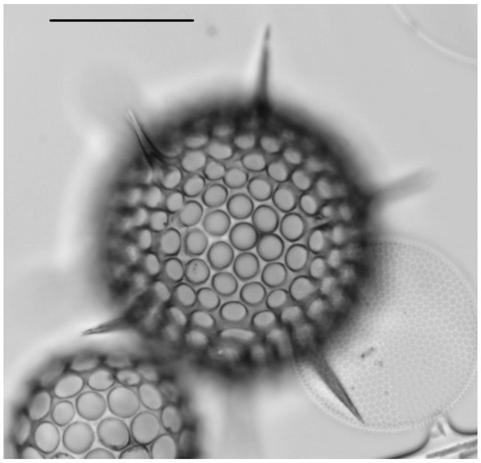

unknown actinommid >6 spines sp 26

Actinommidae

Undescribed

Records: 11

This species resembles unknown actinommid >6 spines sp 16, but differs in that it has small, conical spines rather than bladed ones.

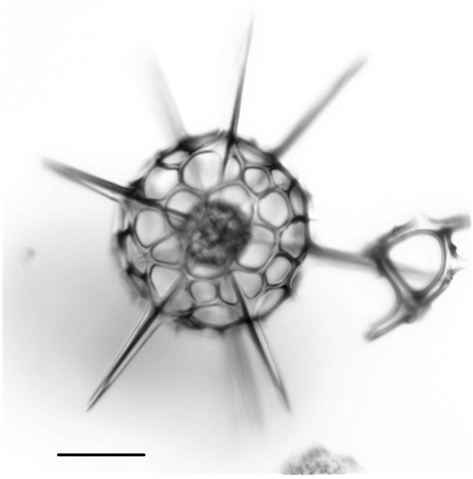

unknown actinommid >6 spines sp 27

Actinommidae

Undescribed

Records: 6

This species is unique for its large size, and very large cortical shell pores. It has ~12 regularly-spaced thin bladed spines. Two small medullary shells are present.

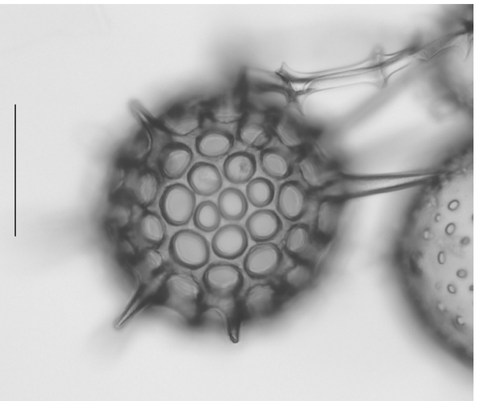

unknown actinommid >6 spines sp 28

Actinommidae

Undescribed

Records: 12

This species is unique for its large and irregular framed pores, and lack of medullary shell.

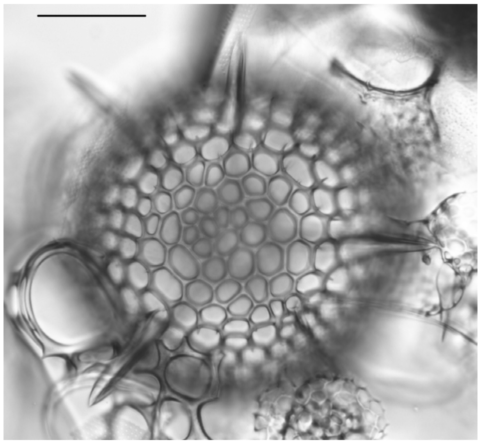

unknown actinommid >6 spines sp 6

Actinommidae

Undescribed

Records: 24

This species has small, angular pores with barbs. Approximately twelve bladed spines, and a relatively small double medullary shell.

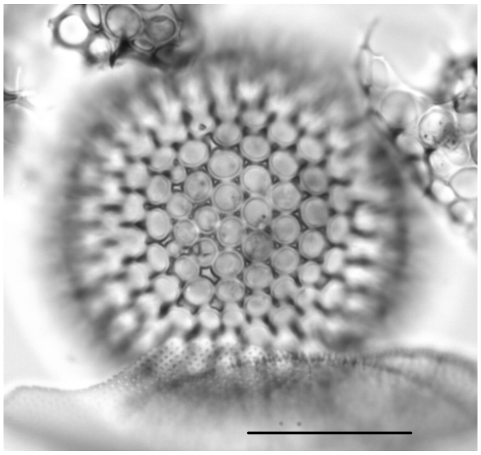

unknown actinommid sp 10

Actinommidae

Undescribed

Records: 29

This species has small, round, and rough pores, and lacks a medullary shell. Differs from Cenosphaera sp 2 in that it has smaller pores.

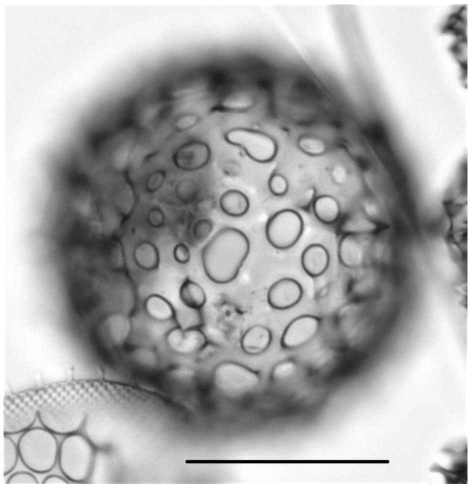

unknown actinommid sp 14

Actinommidae

Undescribed

Records: 21

This species is unique for its rough exterior with highly irregular pores.

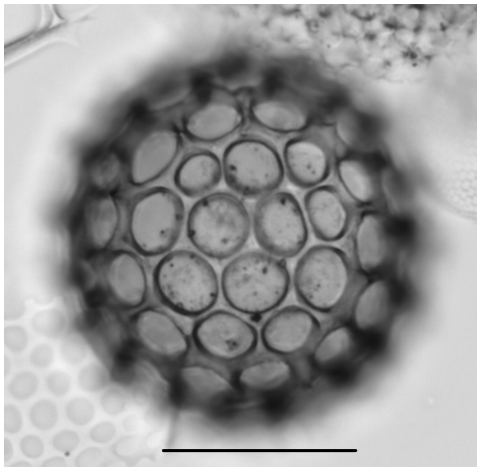

unknown actinommid sp 18

Actinommidae

Undescribed

Records: 42

This species differs from Cenosphaera sp 2 and unknown actinommid sp 10 in that it has relatively large pores and small shell diameter.

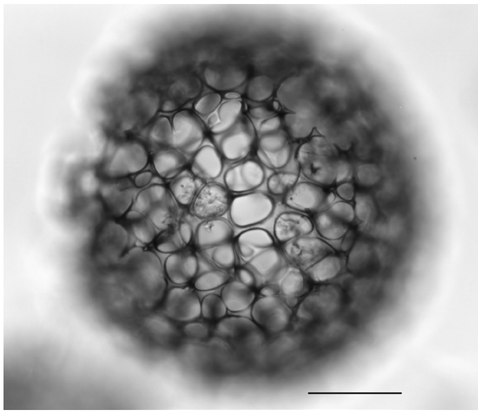

unknown actinommid sp 19

Actinommidae

Undescribed

Records: 14

This species is unique for having a spongy double cortical shell wall.

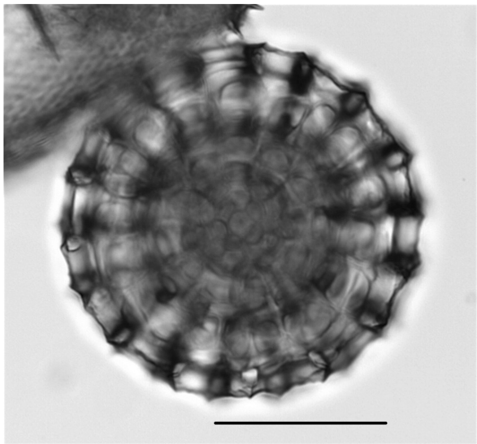

unknown actinommid sp 20

Actinommidae

Undescribed

Records: 6

This species is unique for having two medullary shells equal distance between them and between the cortical shell. The shell is robust, and the cortical shell has very large pores.

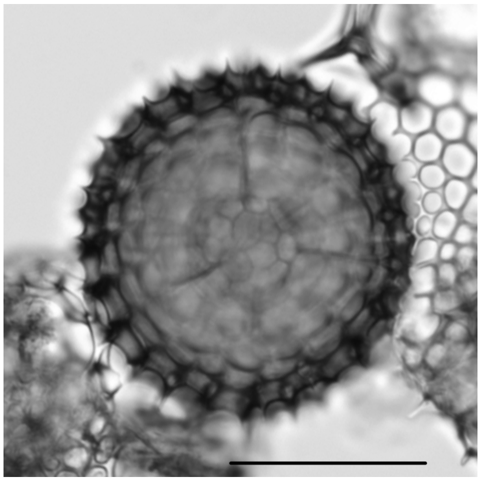

unknown actinommid sp 21

Actinommidae

Undescribed

Records: 6

This species is small, with a thorny cortical shell, and fragile, angular medullary shell.

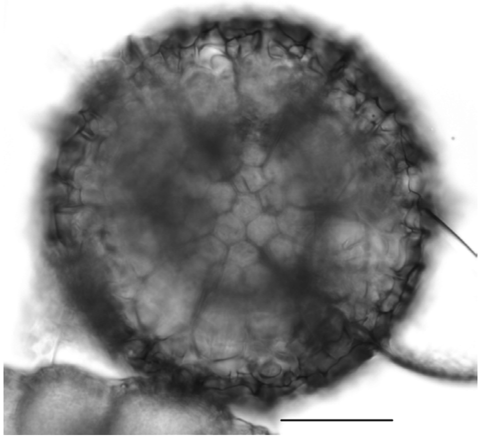

unknown actinommid sp 22

Actinommidae

Undescribed

Records: 9

This species is unique for its thick, rough cortical shell, and large spherical medullary shell with hexagonal pores.

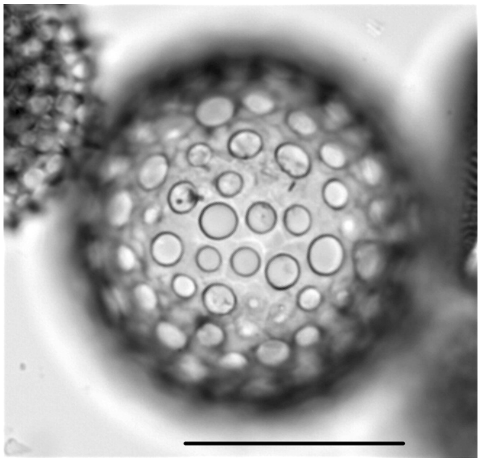

unknown actinommid sp 23

Actinommidae

Undescribed

Records: 19

This species has circular pores that are irregularly distributed on shell surface. This species is small and has no medullary shell.

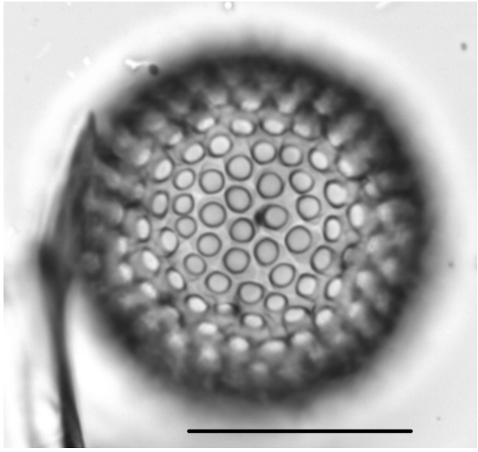

unknown actinommid sp 24

Actinommidae

Undescribed

Records: 8

This species differs from unknown actinommid sp 23 for having smaller, more closely spaced pores. It differs from unknown actinommid sp 10 in that it has weaker frames on the pores and is ~25% smaller.

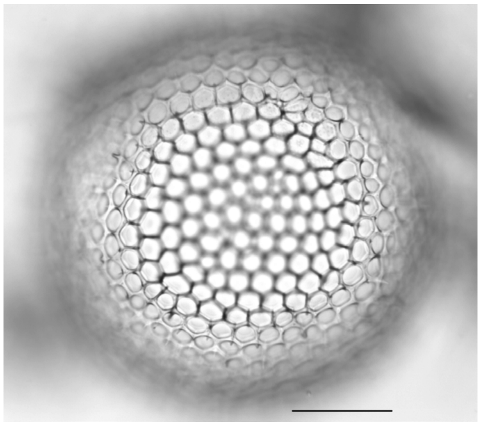

unknown actinommid sp 25

Actinommidae

Undescribed

Records: 5

This species is unique for its large size, and very regular, hexagonal pores, with small, regularly-distributed spines.

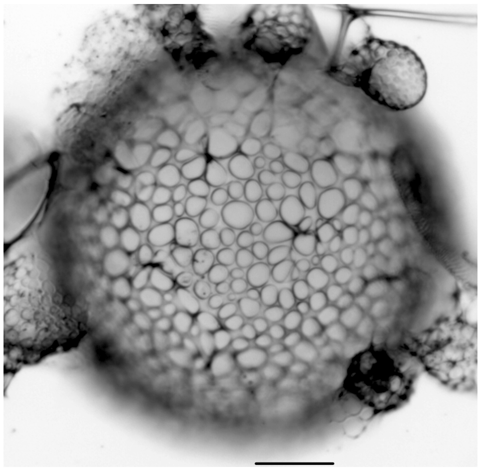

unknown actinommid sp 26

Actinommidae

Undescribed

Records: 16

This species differs from Carposphaera annikae in that it is larger in size and has smaller pores relative to the size of the shell.

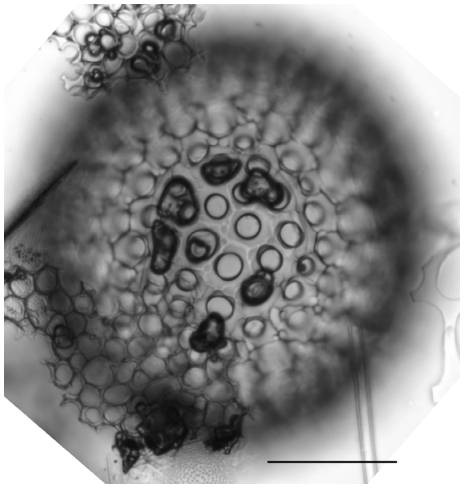

unknown actinommid sp 27

Actinommidae

Undescribed

Records: 6

This species differs from the others in that it is very thick-walled, with large framed pores, and a slightly oval shape. Frames on pores are distinctly wrinkly.

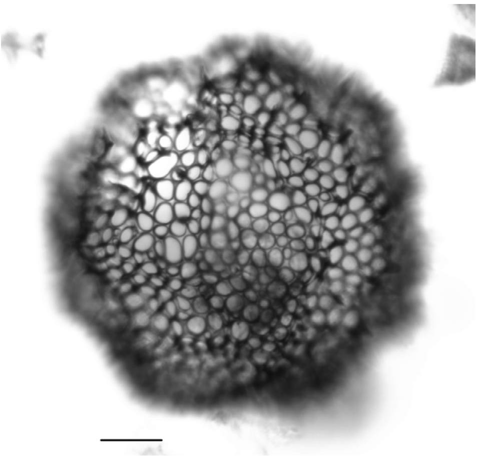

unknown actinommid sp 28

Actinommidae

Undescribed

Records: 17

This species is unique for its irregular, lumpy outer shell. Surface of shell is thorny, and its diameter is relatively large.

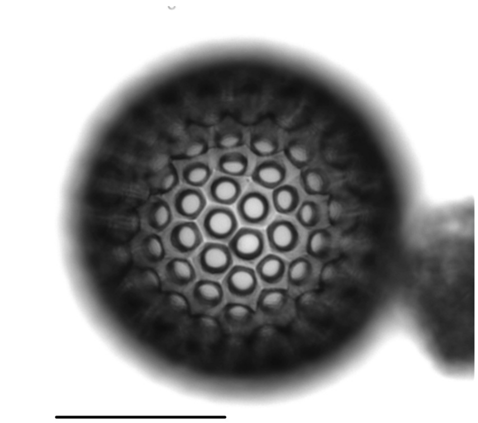

unknown actinommid sp 29

Actinommidae

Undescribed

Records: 21

This species is relatively small, with regular, hexagonally-framed pores and no visible medullary shell.

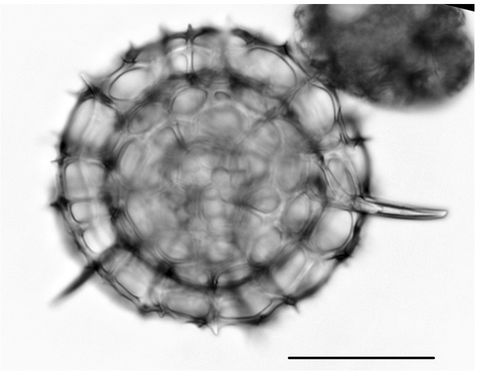

unknown actinommid sp 30

Actinommidae

Undescribed

Records: 12

This species resembles Heliosoma dispar, except that it has an additional outer cortical shell, and has sporadic bladed spines.

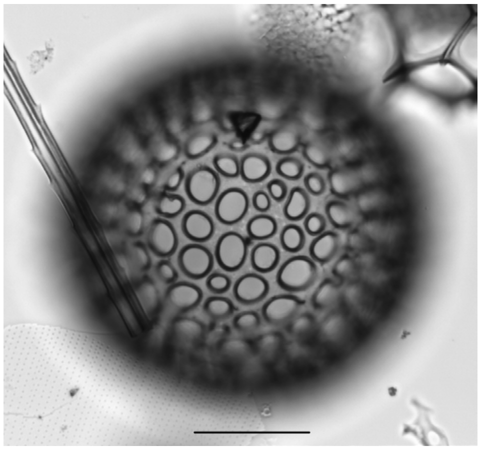

unknown actinommid sp 5

Actinommidae

Undescribed

Records: 22

This species resembles unknown actinommid sp 23, but is approximately double its size.

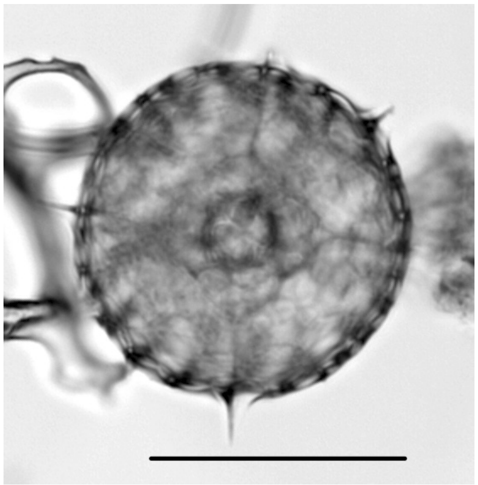

unknown actinommid sp 6

Actinommidae

Undescribed

Records: 18

This species is unique for its square-shaped medullary shell.

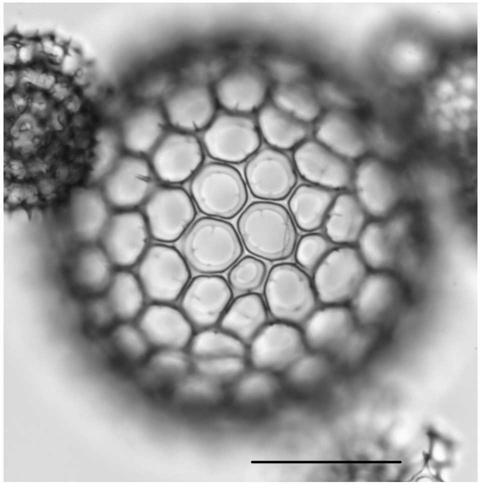

unknown actinommid sp 9

Actinommidae

Undescribed

Records: 113

This species is unique for having narrow, angular, framed pores with a fragile overgrowth that is sometimes preserved.

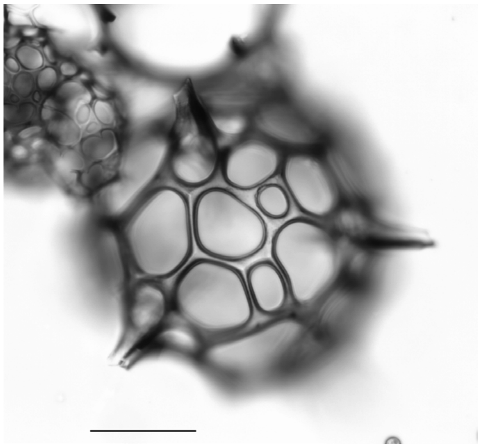

Hexacontium arachnoidale

Actinommidae (hexastylid)

Hollande & Enjumet, 1960

Records: 30

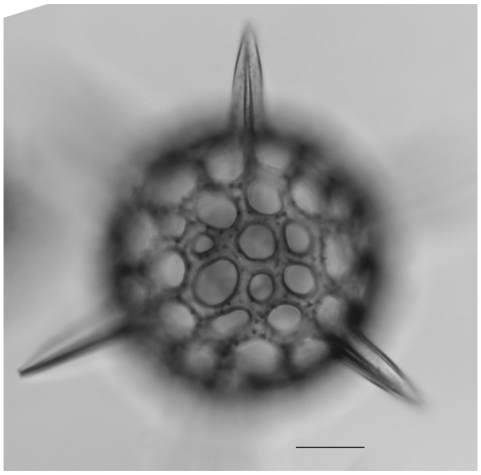

Hexacontium armatum-hostile

Actinommidae (hexastylid)

Cleve, 1900

Records: 81

Temporary name

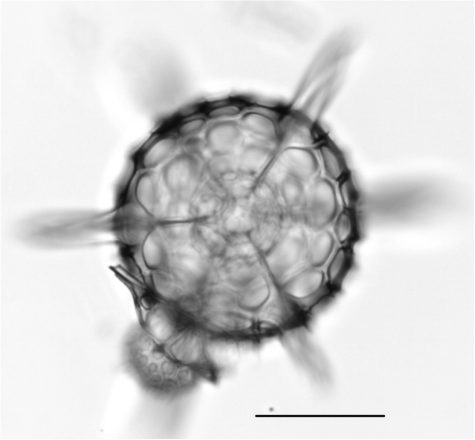

Hexacontium enthacanthum
Actinommidae (hexastylid)
Jørgensen, 1900
Records: 107

sensu Cortese and Bjørklund, 1998

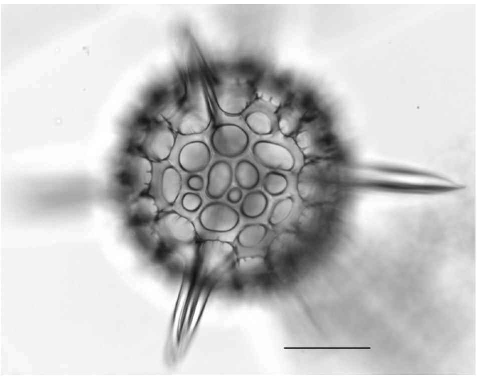

Hexacontium hostile cf
Actinommidae (hexastylid)
Undescribed
Records: 92

This species differs from H. hostile in that it has much larger, knobbier pores. Medullary shell is significantly smaller than in H. hostile.

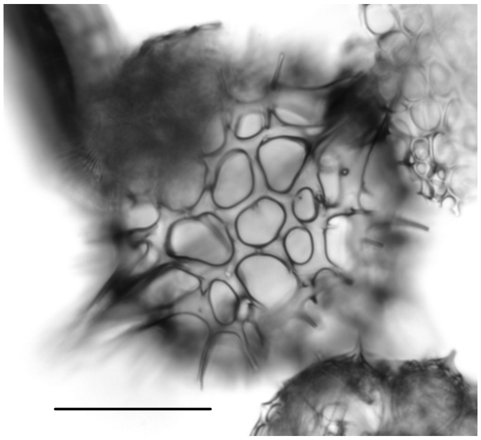

Hexacontium hostile cf sp 2
Actinommidae (hexastylid)
Undescribed
Records: 8

This species differs from H. hostile and H. hostile cf, except that spines are longer, cortical shell is thicker, and pores are more irregular.

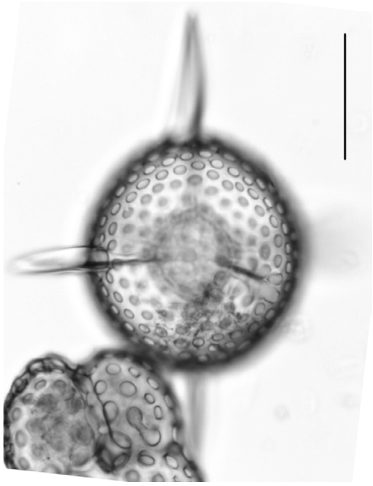

Hexacontium laevigatum
Actinommidae (hexastylid)
Haeckel, 1887
Records: 53

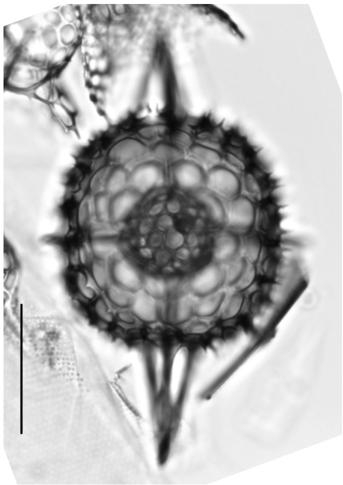

Hexacontium melpomene
Actinommidae (hexastylid)
(Haeckel) Van de Paverd, 1995
Records: 104

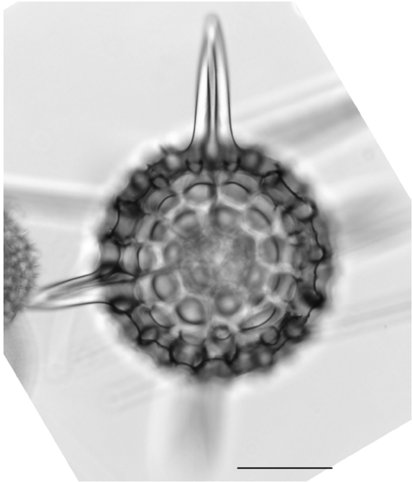

Hexacontium pachydermum
Actinommidae (hexastylid)
Jørgensen, 1900
Records: 54

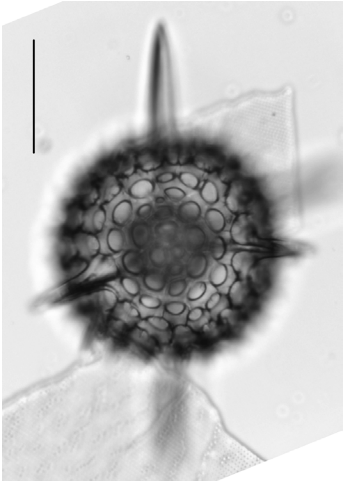

Hexacontium pachydermum cf

Actinommidae (hexastylid)

Undescribed

Records: 28

This species differs from H. pachydermum in that it has relatively smaller and more numerous pores. It also has a slightly larger cortical shell.

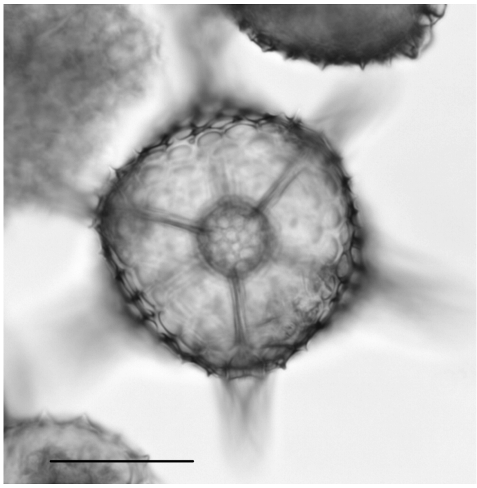

Hexalonche octahedra

Actinommidae (hexastylid)

Haeckel, 1887

Records: 36

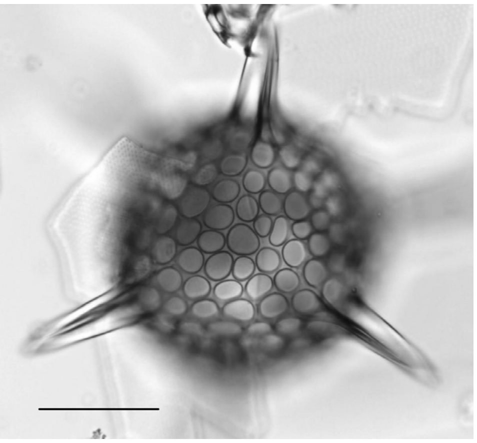

Hexastylus dimensivus

Actinommidae (hexastylid)

Haeckel, 1887

Records: 57

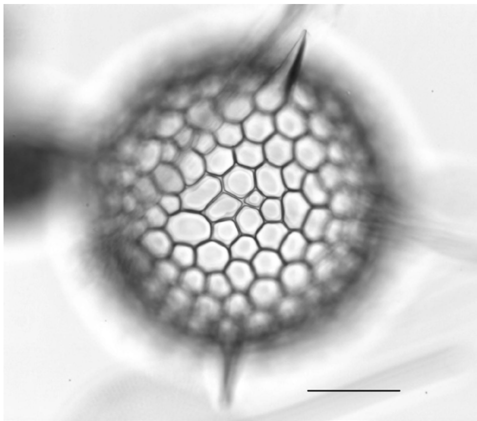

Hexastylus dimensivus cf

Actinommidae (hexastylid)

Undescribed

Records: 13

This species differs from H. dimensivus in that it has more angular pores.

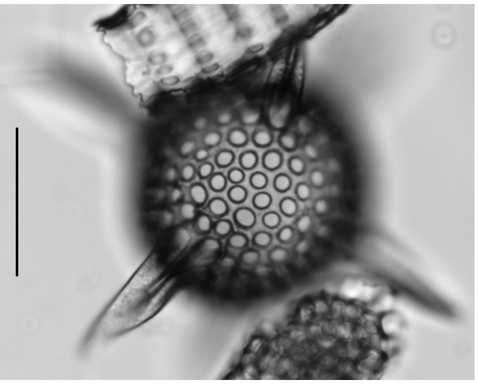

Hexastylus thaletis

Actinommidae (hexastylid)

Haeckel, 1887

Records: 16

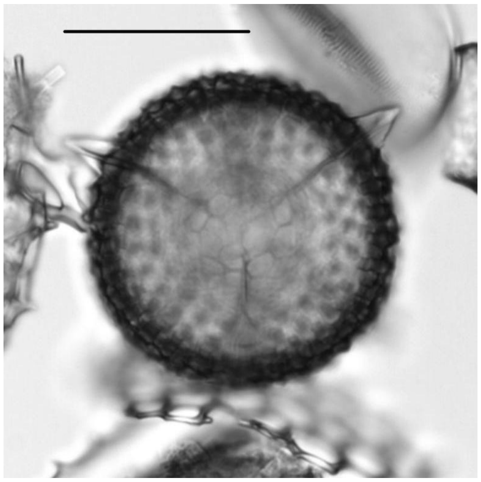

Hexastylus thaletis cf

Actinommidae (hexastylid)

Undescribed

Records: 9

This species differs from H. thaletis in that it has two medullary shells and shorter spines.

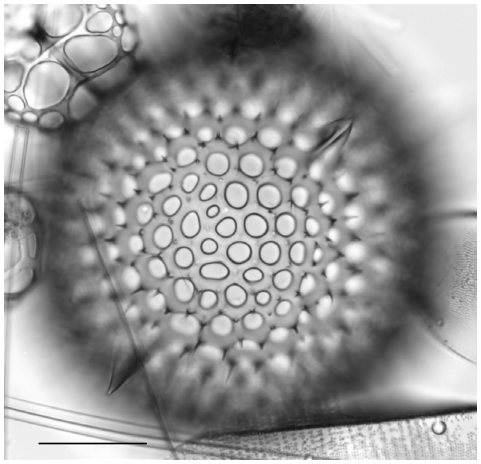

unknown hexastylid sp 1

Actinommidae (hexastylid)

Undescribed

Records: 8

This species is large, with very short, bladed spines. Smaller, needle-like spines are present throughout the cortical shell surface. No medullary shell is present. Pores are small, framed, angular, and irregular.

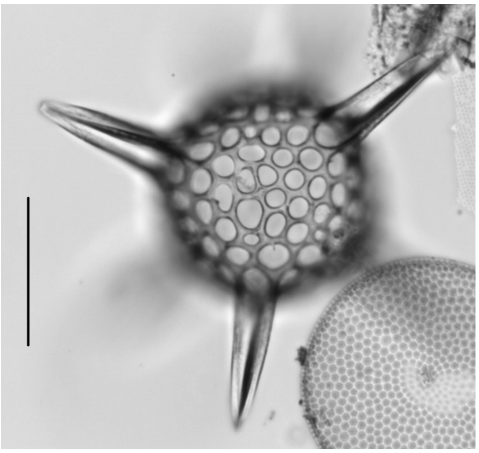

unknown hexastylid sp 11

Actinommidae (hexastylid)

Undescribed

Records: 79

This species has a spherical shape and no medullary shell. It is unique for having proportionally long, bladed spines, compared to the size of the cortical shell. Differs from H. dimensivus in that it has fewer, larger pores.

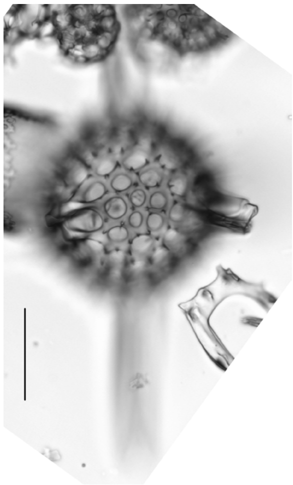

unknown hexastylid sp 11 cf

Actinommidae (hexastylid)

Undescribed

Records: 32

This species has similar spine and cortical shell proportions to unknown hexastylid sp 11, but differs in that it has needle-like spines throughout cortical shell, and has a medullary shell, and an overall less spherical shape. It differs from unknown hexastylid sp 27 in that it has a thicker cortical shell and tends to be less spherical in shape.

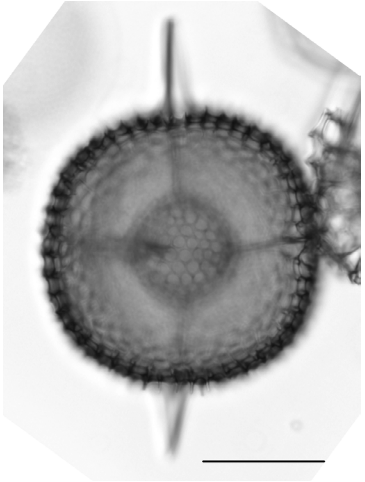

unknown hexastylid sp 12

Actinommidae (hexastylid)

Undescribed

Records: 77

This species is distinctive for its large, squarish-shaped cortical shell. It has small, sub-rounded pores, and a relatively thin shell wall.

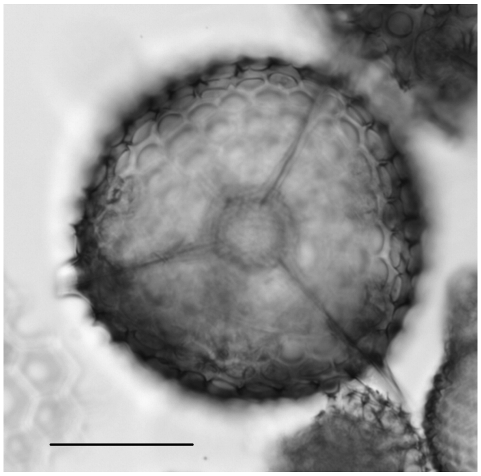

unknown hexastylid sp 13

Actinommidae (hexastylid)

Undescribed

Records: 29

This species has a distinctively small, smooth, and spherical medullary shell.

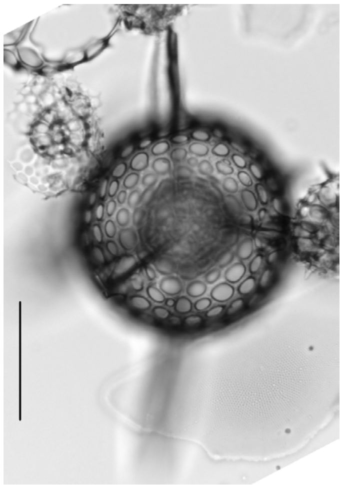

unknown hexastylid sp 14

Actinommidae (hexastylid)

Undescribed

Records: 75

This species resembles Hexacontium laevigatum, but has significantly larger pores and its medullary shell tends to be slightly larger.

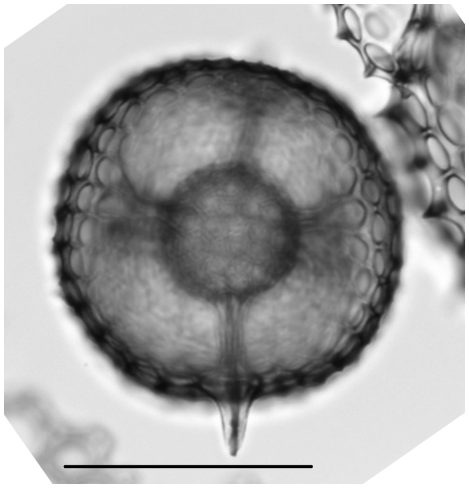

unknown hexastylid sp 14 cf

Actinommidae (hexastylid)

Undescribed

Records: 41

This species resembles unknown hexastylid sp 14, but differs in that it has slightly compressed sides, giving the cortical shell a more square shape.

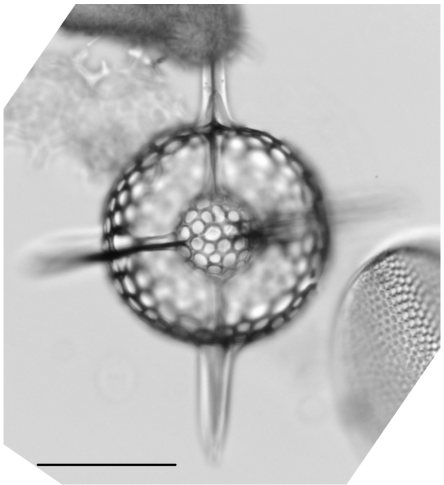

unknown hexastylid sp 14 cf sp 2

Actinommidae (hexastylid)

Undescribed

Records: 40

This species is like unknown hexastylid sp 14, but is consistently slightly smaller in overall size.

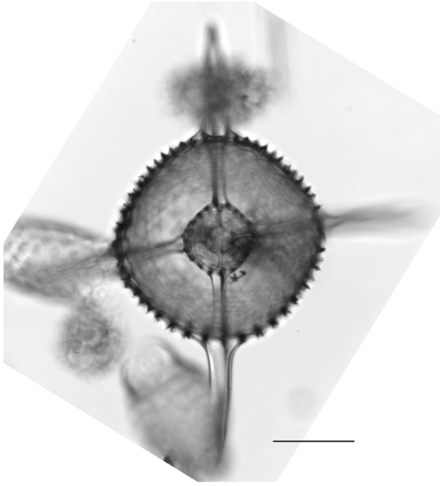

unknown hexastylid sp 16

Actinommidae (hexastylid)

Undescribed

Records: 24

This species is distinct for its large size and cube-shaped cortical shell. Differs from unknown hexastylid sp 12 in that it has relatively longer, bladed spines. It also tends to have a thornier exterior.

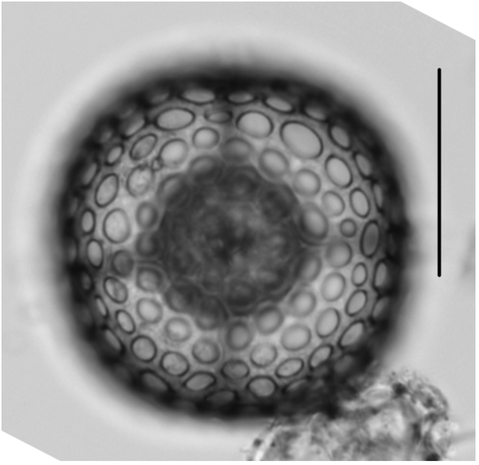

unknown hexastylid sp 18

Actinommidae (hexastylid)

Undescribed

Records: 26

This species has flattened sides where the six spines protrude. Differs from Hexacontium melpomene in that it has a smoother, thinner surface of cortical shell and small, muted spines.

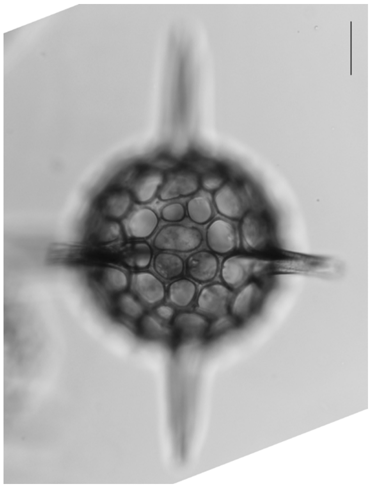

unknown hexastylid sp 19

Actinommidae (hexastylid)

Undescribed

Records: 24

This species' cortical shell diameter is larger than Hexacontium hostile, and outside of cortical shell is smoother. Pores at the base of spines are enlarged and medullary shell is somewhat irregular.

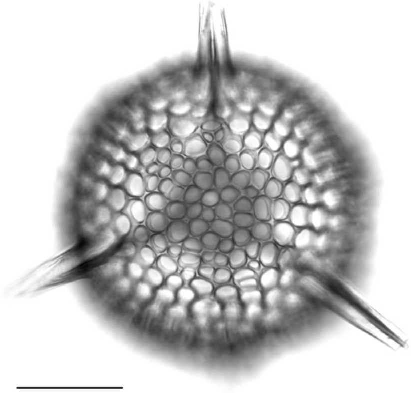

unknown hexastylid sp 2

Actinommidae (hexastylid)

Undescribed

Records: 104

This species is large, with small angular pores. Small needle-like spines all around shell, in addition to the six prominent bladed spines. Cortical and medullary shells are spherical in shape, and often two medullary shells are present.

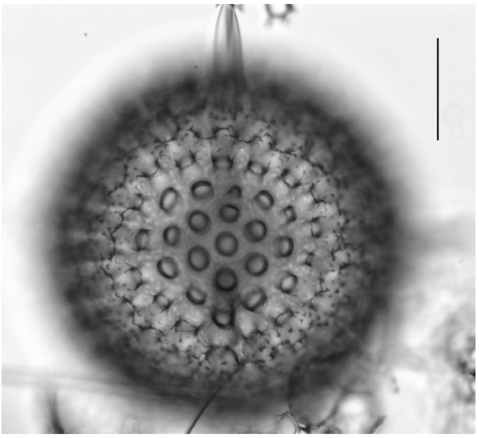

unknown hexastylid sp 2 cf

Actinommidae (hexastylid)

Undescribed

Records: 15

This species differs from unknown hexastylid sp 2 in that it has a more spiny exterior, significantly thicker cortical shell, and rounder pores.

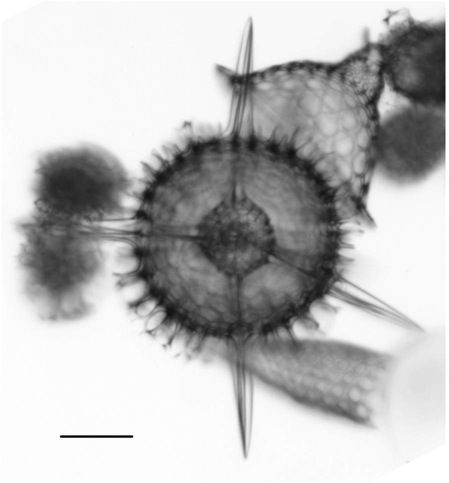

unknown hexastylid sp 20

Actinommidae (hexastylid)

Undescribed

Records: 56

This species resembles unknown hexastylid sp 2, but differs in that it has an extra spiny layer around the cortical shell. Shell surface is also rougher than unknown hexastylid sp 2.

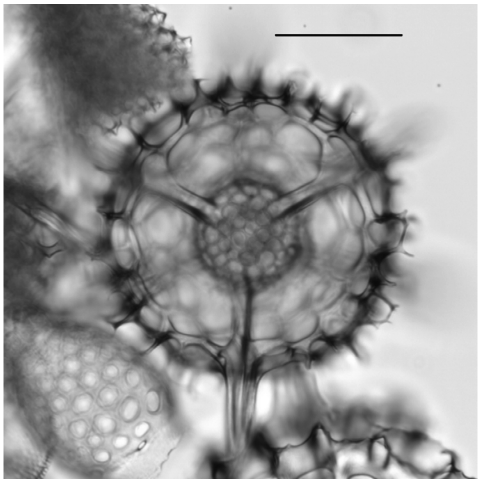

unknown hexastylid sp 21

Actinommidae (hexastylid)

Undescribed

Records: 13

This species differs from unknown hexastylid sp 20 in that it is significantly smaller, and barbs on exterior of shell do not form a full layer.

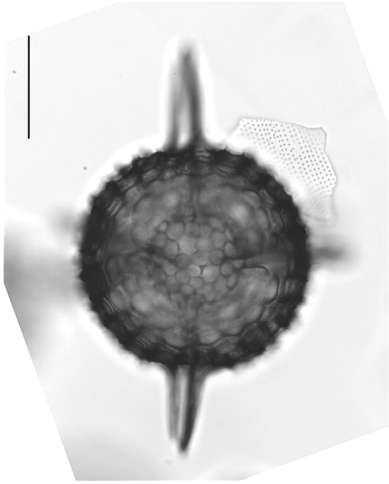

unknown hexastylid sp 22

Actinommidae (hexastylid)

Undescribed

Records: 37

This species resembles Hexacontium hostile, but has a relatively larger medullary shell.

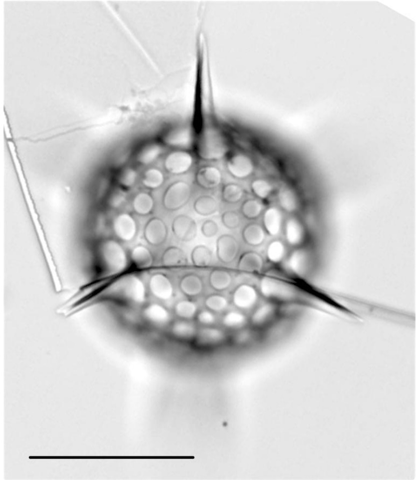

unknown hexastylid sp 23

Actinommidae (hexastylid)

Undescribed

Records: 22

This species is distinct for its short, bladed spines, smooth exterior, and small, spherical cortical shell. Medullary shell not always preserved.

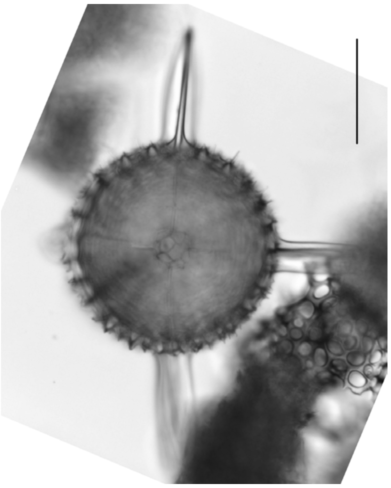

unknown hexastylid sp 24

Actinommidae (hexastylid)

Undescribed

Records: 57

This species has a distinctively small medullary shell, but differs from unknown hexastylid sp 13 in that its medullary shell is irregularly-shaped with very large, angular pores.

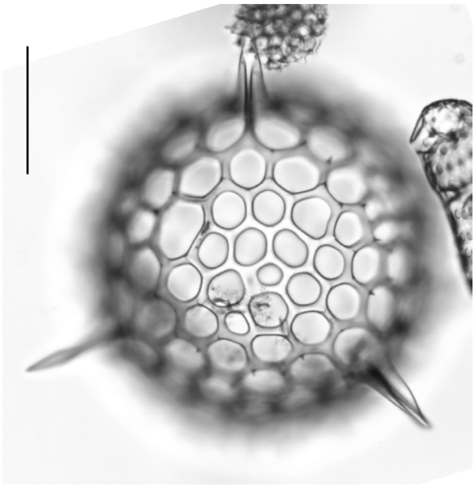

unknown hexastylid sp 25

Actinommidae (hexastylid)

Undescribed

Records: 53

This species is unique for its thick bars with small needles, short, bladed spines, and lack of a medullary shell.

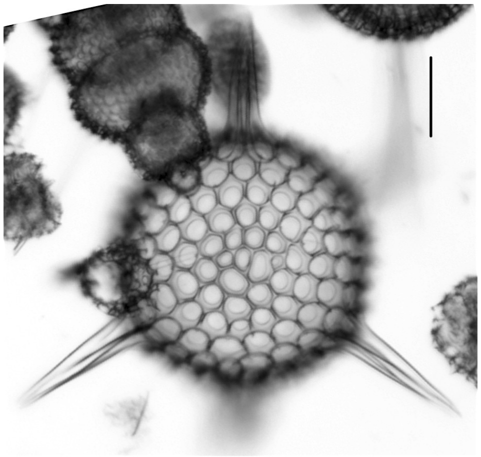

unknown hexastylid sp 25 cf

Actinommidae (hexastylid)

Undescribed

Records: 10

This species differs from unknown hexastylid sp 25 in that it has smaller, framed pores, which are round on the interior side, and angular on the exterior side.

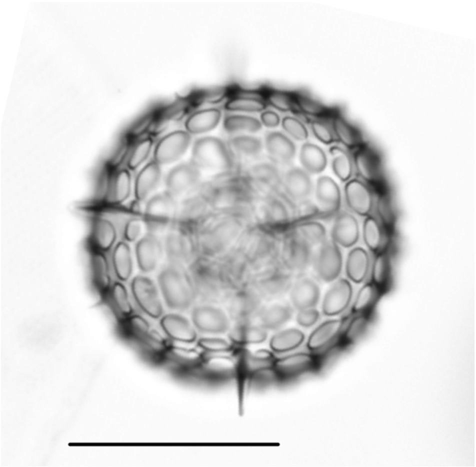

unknown hexastylid sp 26

Actinommidae (hexastylid)

Undescribed

Records: 29

This species is distinctively small, with irregular-shaped, barbed pores, short bladed spines, and a medullary shell.

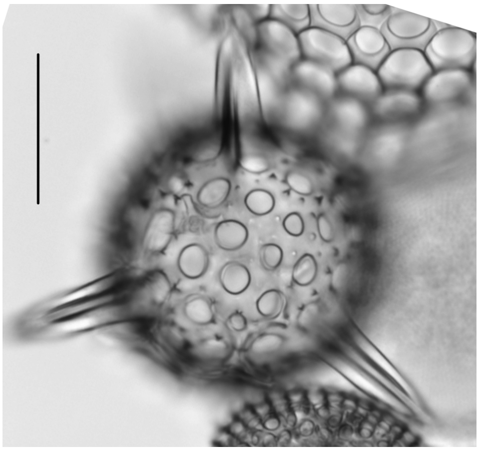

unknown hexastylid sp 27

Actinommidae (hexastylid)

Undescribed

Records: 33

This species is similar to unknown hexastylid sp 11 cf, but differs in that it is more spherical in shape, typically has a more fragile, poorly-deveoped medullary shell, and a smoother shell exterior.

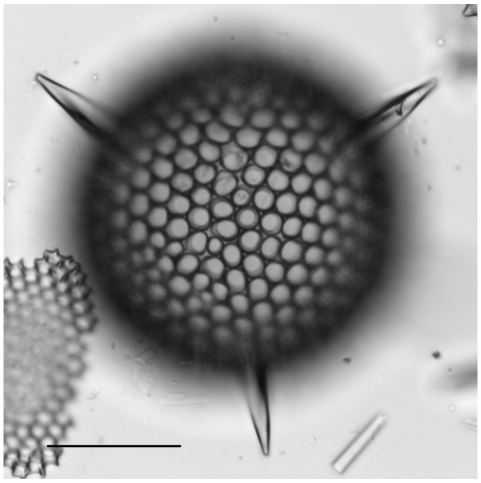

unknown hexastylid sp 28

Actinommidae (hexastylid)

Undescribed

Records: 10

This species shares many characters with unknown hexastylid sp 25 cf, but differs in that its diameter is smaller, with smaller, more numerous pores, and tends to have shorter bladed spines.

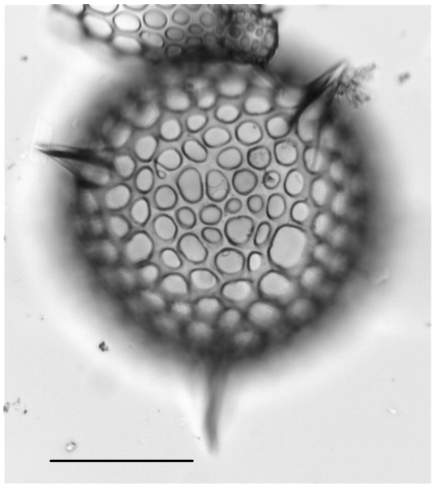

unknown hexastylid sp 29

Actinommidae (hexastylid)

Undescribed

Records: 21

This species differs from unknown hexastylid sp 28 in that its pores are more irregular, it has a medullary shell, and its diameter is slightly smaller.

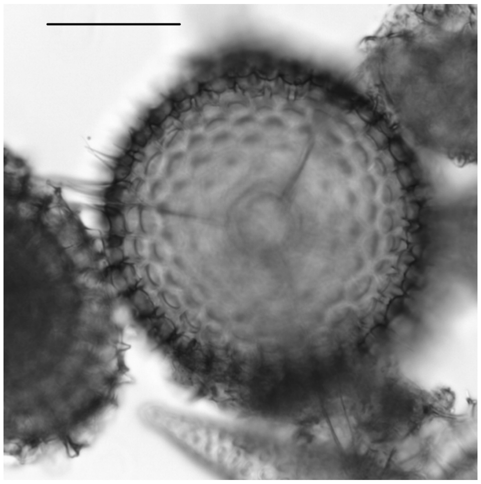

unknown hexastylid sp 30

Actinommidae (hexastylid)

Undescribed

Records: 16

This species resembles unknown hexastylid sp 13, but differs int hat it has the additional feature of short conical spines on cortical shell exterior. It differs from unknown hexastylid sp 11 cf and unknown hexastylid sp 27 in that it has smaller, more numerous pores, and a relatively smaller medullary shell.

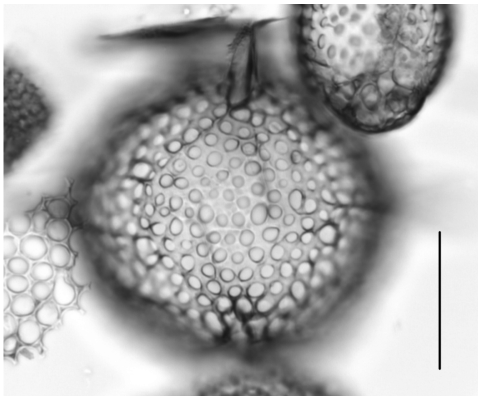

unknown hexastylid sp 31

Actinommidae (hexastylid)

Undescribed

Records: 9

This species is distinctive for its irregular ridges on the cortical shell surface, and oval shape. Spines are bladed and broad. Pores are very small, and no medullary shell is present.

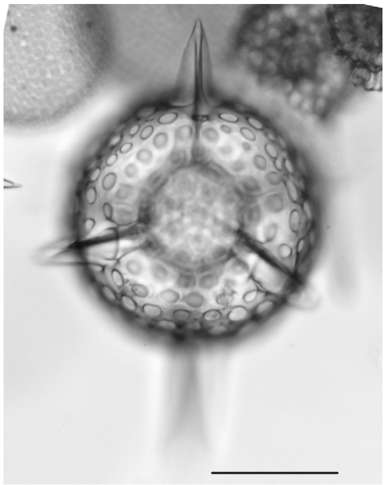

unknown hexastylid sp 32

Actinommidae (hexastylid)

Undescribed

Records: 40

This species differs from unknown hexastylid sp 27 in that it has a relatively larger medullary shell and smaller pores.

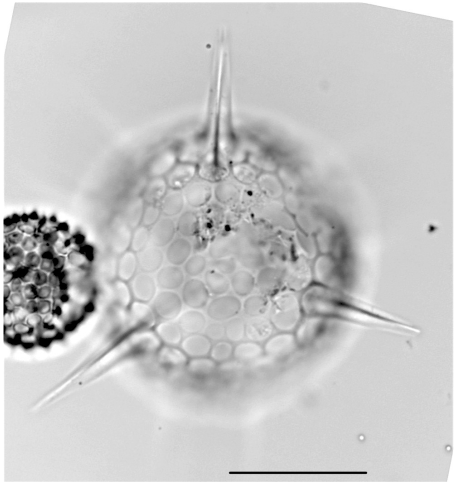

unknown hexastylid sp 33

Actinommidae (hexastylid)

Undescribed

Records: 14

This species has proportions that resemble unknown hexastylid sp 2, but differs in that it is much more lightly silicified and smaller in size.

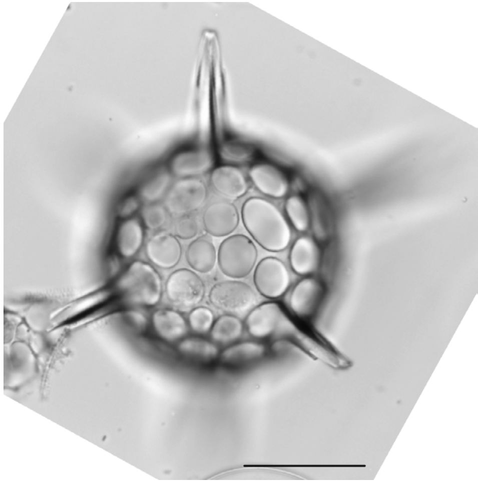

unknown hexastylid sp 34

Actinommidae (hexastylid)

Undescribed

Records: 42

This species differs from Hexacontium enthacanthum in that it has a much smaller medullary shell. It differs from unknown hexastylid sp 33 in that its pores are approximately twice as large.

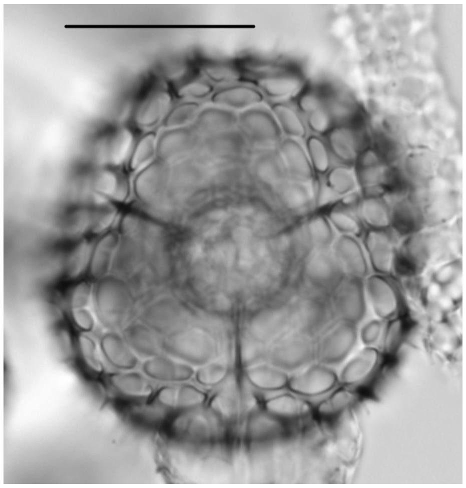

unknown hexastylid sp 35

Actinommidae (hexastylid)

Undescribed

Records: 14

This species is unique for its cortical shell indentations that are associated with internal bars. Pores are angular, sometimes hexagonal, and are barbed. Well-developed medullary shell is present.

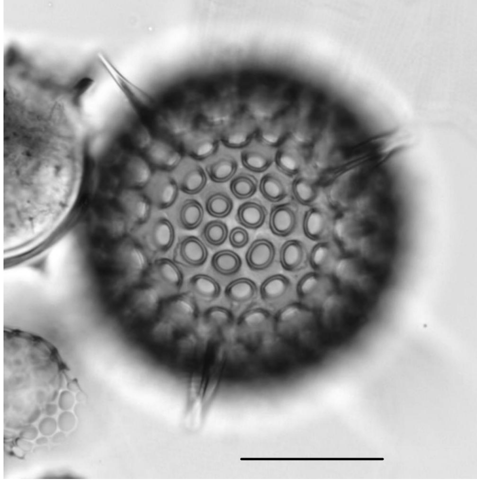

unknown hexastylid sp 36

Actinommidae (hexastylid)

Undescribed

Records: 16

This species differs from Hexastylus thaletis cf in that it is larger, and has larger, framed pores.

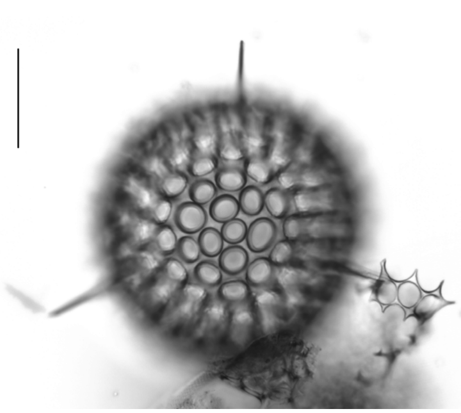

unknown hexastylid sp 36 cf

Actinommidae (hexastylid)

Undescribed

Records: 16

This species differs from unknown hexastylid sp 36 in that it has significantly larger pores on its cortical shell.

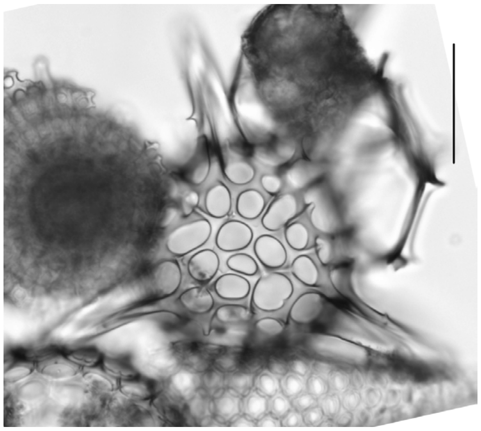

unknown hexastylid sp 37

Actinommidae (hexastylid)

Undescribed

Records: 6

This species has unique pore structure, which includes large, irregular pores with very strong barbs that point in various directions. This species also has robust cortical shell and six bladed spines, but lacks a medullary shell.

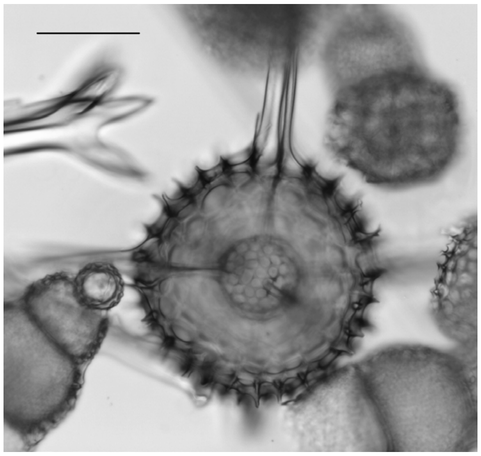

unknown hexastylid sp 38

Actinommidae (hexastylid)

Undescribed

Records: 36

This species resembles unknown hexastylid sp 2, but differs in that it has stronger barbs on pores, and cortical shell tends to be thicker, smaller, and less spherical.

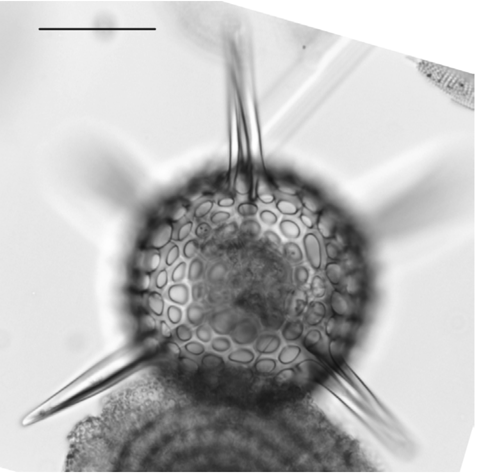

unknown hexastylid sp 39

Actinommidae (hexastylid)

Undescribed

Records: 5

This species differs from unknown hexastylid sp 2 in that it has a relatively larger medullary shell.

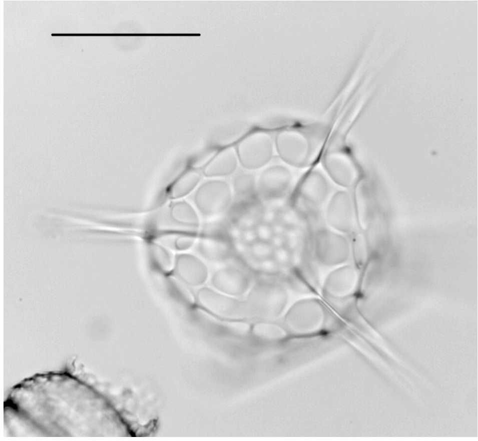

unknown hexastylid sp 4

Actinommidae (hexastylid)

Undescribed

Records: 9

This species is distinctly triangular, lightly silicified, with large pores.

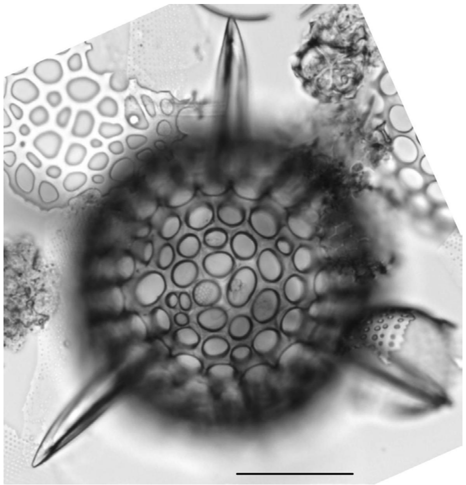

unknown hexastylid sp 40

Actinommidae (hexastylid)

Undescribed

Records: 12

This species is robust, spherical, with sub-rounded, irregular pores on the cortical shell.

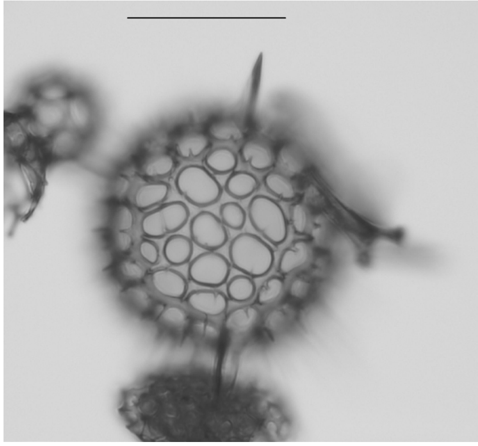

unknown hexastylid sp 41

Actinommidae (hexastylid)

Undescribed

Records: 13

This species is distinct for its small size, large and irregular pores, and numerous thin conical spines in addition to six strong bladed spines.

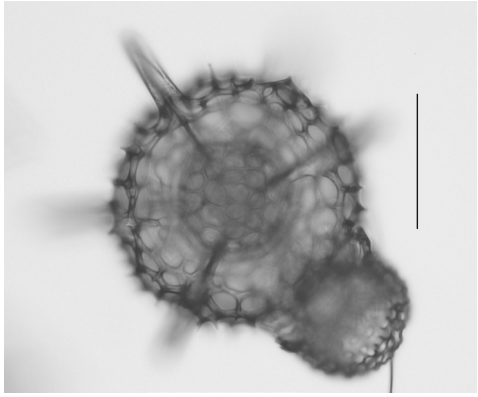

unknown hexastylid sp 42

Actinommidae (hexastylid)

Undescribed

Records: 13

The cortical shell of this species has a unique spongey texture and irregular outline, differentiating it from all other hexastylids observed.

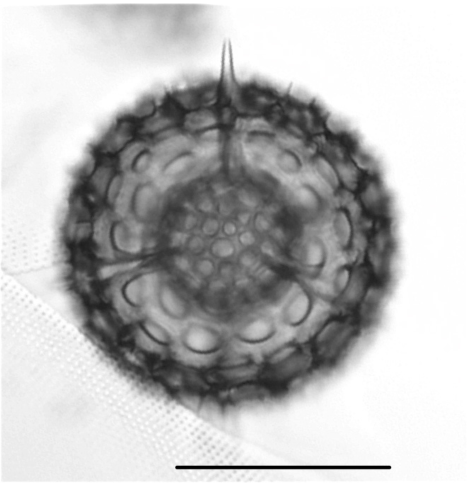

unknown hexastylid sp 8

Actinommidae (hexastylid)

Undescribed

Records: 61

This species is unique for its proportionally large medullary shell, conical spines, and rough cortical shell exterior.

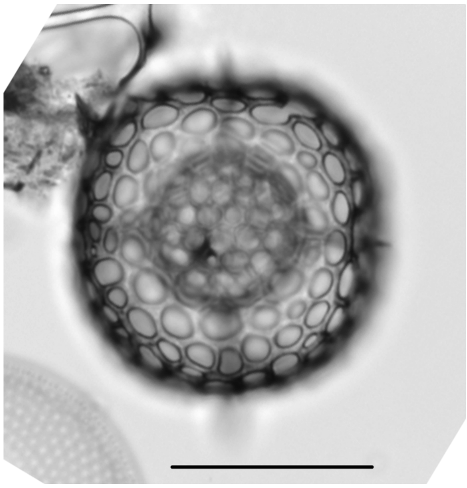

unknown hexastylid sp 9

Actinommidae (hexastylid)

Undescribed

Records: 73

This species differs from unknown hexastylid sp 8 in that it has a smoother exterior, more flattened sides and squarish cortical shell outline. It is significantly smaller than unknown hexastylid sp 18 and Hexacontium melpomene.

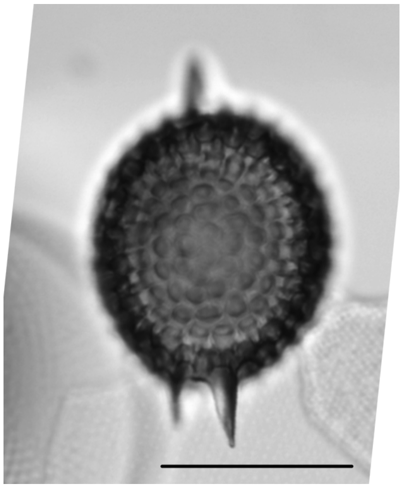

Amphisphaera cristata cf sp 1

Actinommidae (stylosphaerid)

Undescribed

Records: 75

This species has a relatively thick cortical shell, with small, irregular framed pores. There is no trace of a medullary shell. One of the bladed bipolar spines is distinctively split into two or three spines.

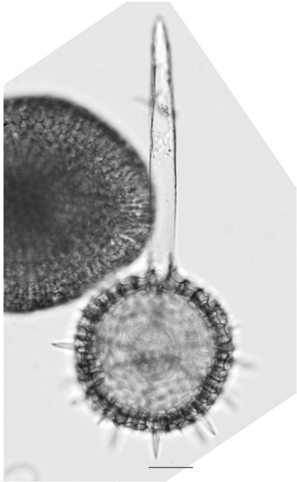

Amphisphaera sp D Kamikuri

Actinommidae (stylosphaerid)

Kamikuri et al. 2009

Records: 19

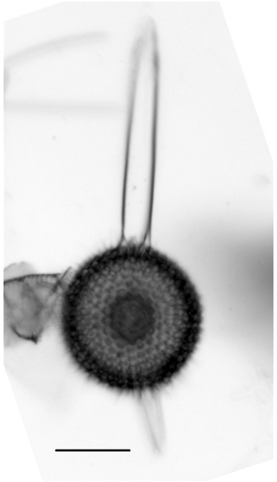

Amphistylus angelinus

Actinommidae (stylosphaerid)

(Campbell & Clark) Chen 1975

Records: 41

sensu Kamikuri, 2017

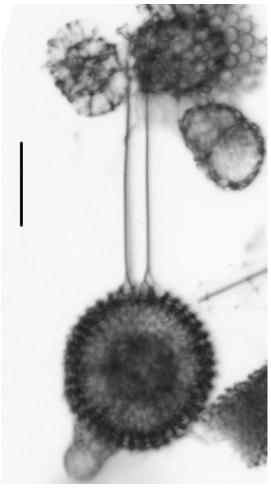

Amphistylus polistylus

Actinommidae (stylosphaerid)

Carnevale, 1908

Records: 6

sensu Kamikuri, 2017

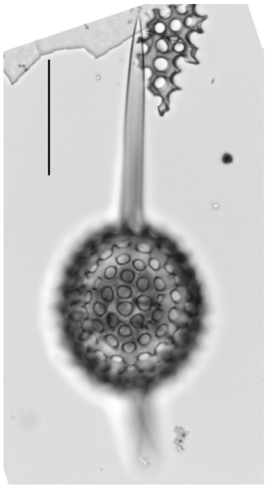

Druppatractus hastatus

Actinommidae (stylosphaerid)

Blueford, 1982

Records: 115

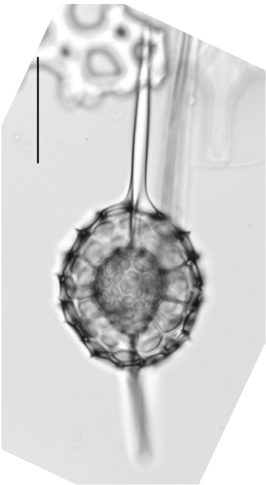

Druppatractus irregularis

Actinommidae (stylosphaerid)

Popofsky, 1912

Records: 89

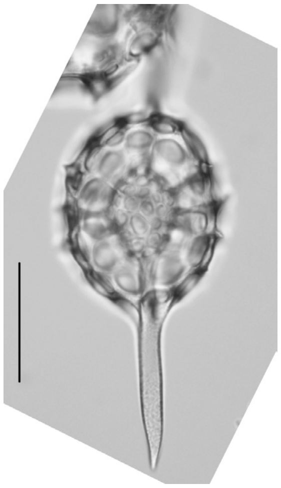

Druppatractus irregularis cf

Actinommidae (stylosphaerid)

Undescribed

Records: 7

This species is similar to D. irregularis, but differs in that the cortical shell and medullary shell are narrower.

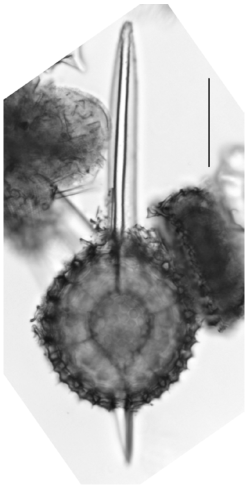

Druppatractus irregularis cf sp 2

Actinommidae (stylosphaerid)

Undescribed

Records: 54

This species differs from D. irregularis in that it has massive, bladed spines, and a wider, more robust cortical shell.

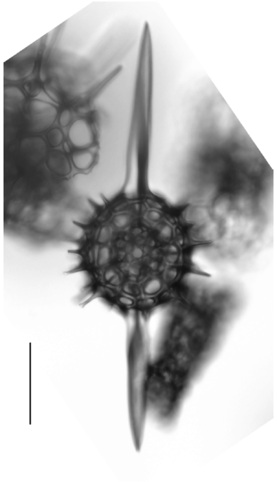

Druppatractus ostracion

Actinommidae (stylosphaerid)

Haeckel, 1887

Records: 24

sensu Takahashi 1991

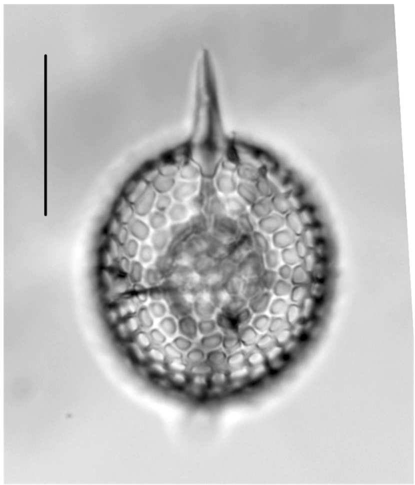

Druppatractus sp 1

Actinommidae (stylosphaerid)

Undescribed

Records: 53

This species has the pear-shaped medullary shell typical of Druppatractus species, but differs from the others in that it has a very fine-pored cortical shell.

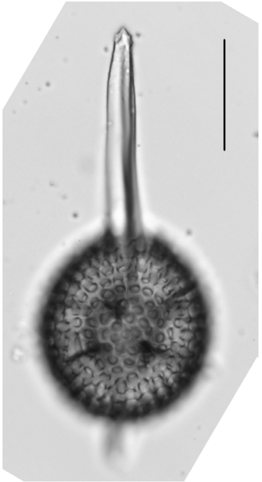

Lithatractus timmsi

Actinommidae (stylosphaerid)

Campbell & Clark, 1944

Records: 144

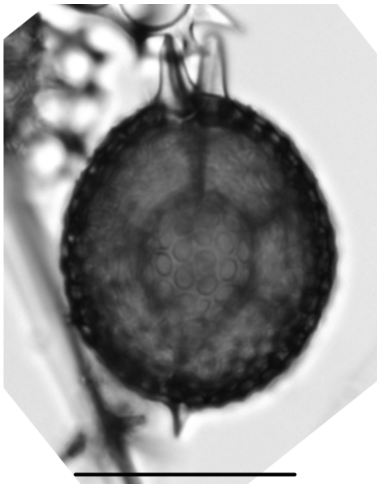

Lithatractus timmsi cf

Actinommidae (stylosphaerid)

Undescribed

Records: 42

This species is like Lithatractus timmsi, but has at least one of its bipolar spines split into at least two pieces. It differs from Amphisphaera cristata cf sp 1 in that it has a medullary shell.

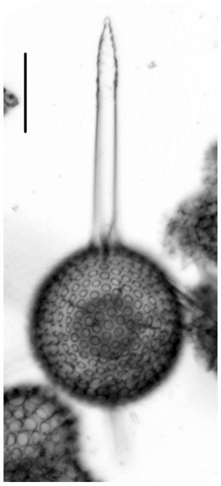

Sphaerostylus cristatus

Actinommidae (stylosphaerid)

Blueford, 1982

Records: 49

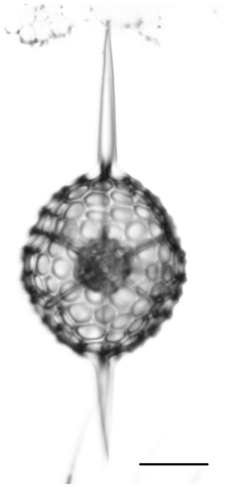

Stylacontarium bispiculum
Actinommidae (stylosphaerid)
Popofsky, 1912
Records: 61

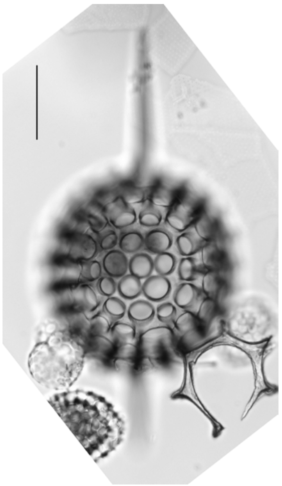

Stylacontarium bispiculum cf
Actinommidae (stylosphaerid)
Undescribed
Records: 17

This species differs from S. bispiculum in that it has a thicker cortical shell, and lacks any evidence of a medullary shell. Its overall shape is also slightly more spherical.

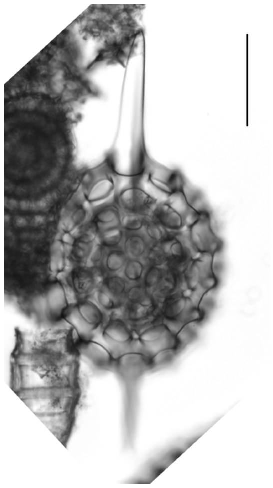

Stylatractus neptunus
Actinommidae (stylosphaerid)
Haeckel, 1887
Records: 57

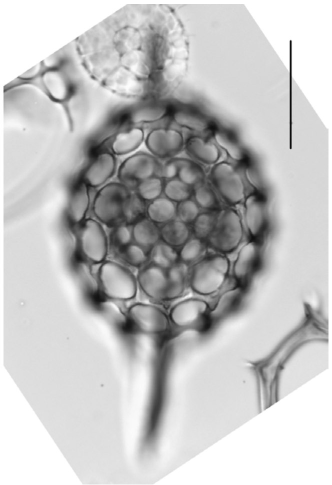

Stylatractus neptunus light
Actinommidae (stylosphaerid)
Undescribed
Records: 20

This species is like S. neptunus except has a much lighter cortical shell.

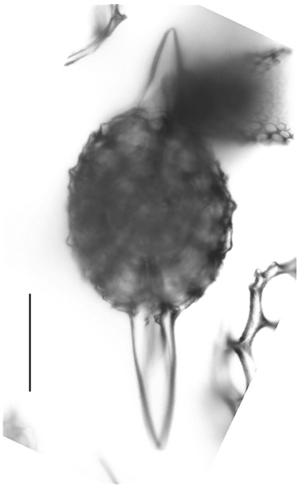

Stylatractus pluto
Actinommidae (stylosphaerid)
Haeckel, 1887
Records: 10

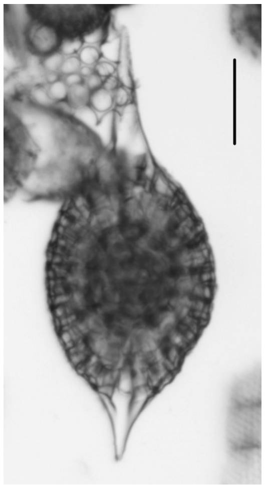

Stylatractus santaeannae
Actinommidae (stylosphaerid)
(Campbell & Clark)
Petrushevskaya & Kozlova, 1972
Records: 91

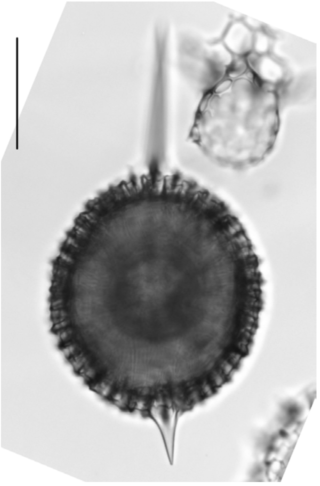

Stylatractus universus
Actinommidae (stylosphaerid)
Hays, 1970
Records: 137

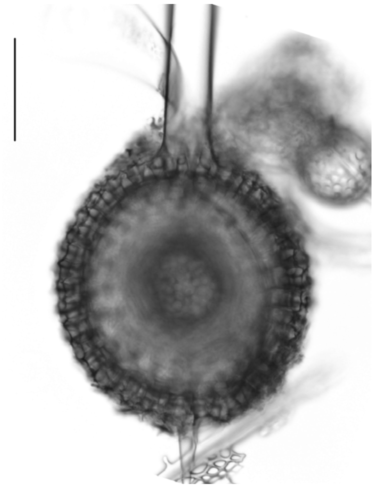

Stylatractus universus cf
Actinommidae (stylosphaerid)
Undescribed
Records: 36

This species resembles S. universus, but has a significantly smaller medullary shell.

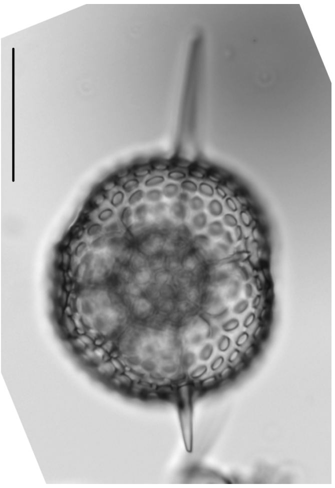

Stylatractus universus transparum
Actinommidae (stylosphaerid)
Lazarus, 1990
Records: 36

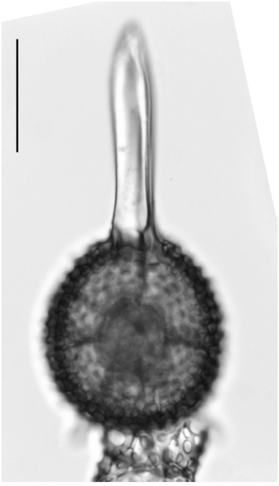

Stylosphaera communis
Actinommidae (stylosphaerid)
Carnevale, 1908
Records: 100

sensu Kamikuri, 2017

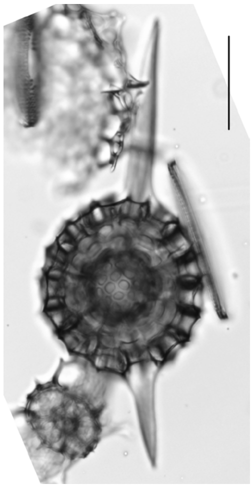

Stylosphaera sp A Kamikuri
Actinommidae (stylosphaerid)
Kamikuri, 2017
Records: 7

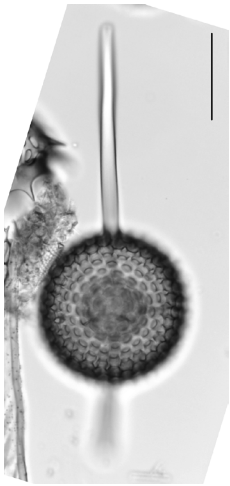

Stylosphaera sp B Kamikuri
Actinommidae (stylosphaerid)
Kamikuri, 2017
Records: 57

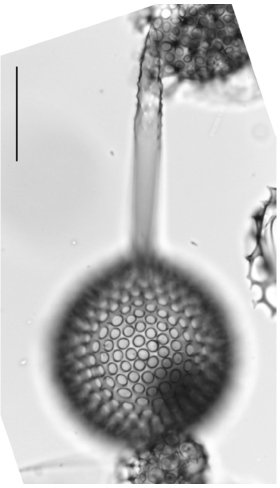

unknown bipolar actinommid 1

Actinommidae (stylosphaerid)

Undescribed

Records: 14

This species has small pores, with some trace of a medullary shell in some specimens, and a single very long conical spine with a rough tip. This species is slightly teardrop-shaped, with the more acute curve on either side of the long spine.

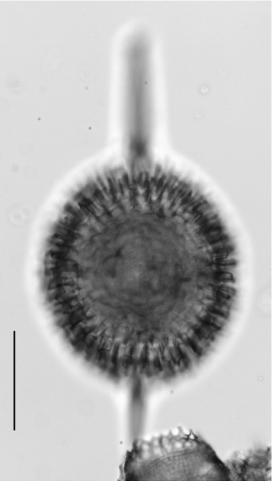

unknown bipolar actinommid 10

Actinommidae (stylosphaerid)

Undescribed

Records: 50

This species is identifiable for its thick cortical shell, with small, crowded pores.

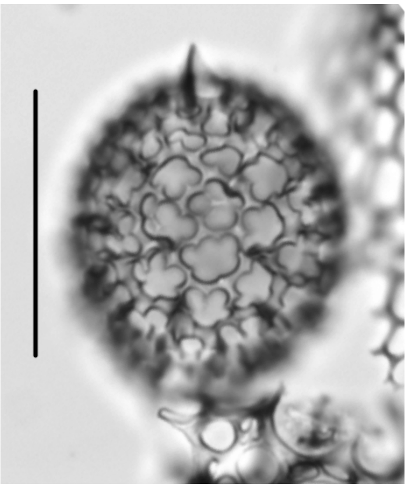

unknown bipolar actinommid 12

Actinommidae (stylosphaerid)

Undescribed

Records: 28

This species has distinct pore “florets” and is relatively small.

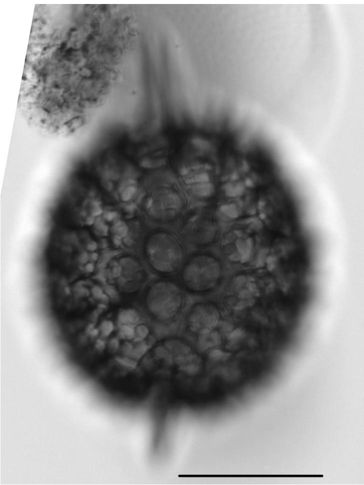

unknown bipolar actinommid 12 cf

Actinommidae (stylosphaerid)

Undescribed

Records: 31

This species differs from unknown bipolar sp 12 in that it is generally larger, has a pear-shaped medullary shell, and the pores are separate from the fine lattice covering them.

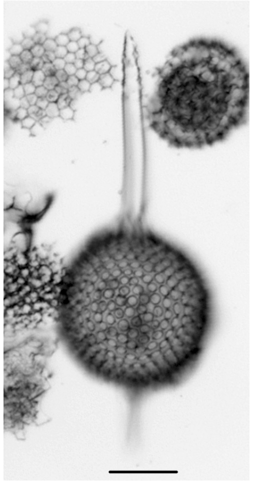

unknown bipolar actinommid 13

Actinommidae (stylosphaerid)

Undescribed

Records: 47

This species is distinguished from Stylosphaera sp B (Kamikuri), Sphaerostylus cristatus, and Amphistylus angelinus in that it lacks a medullary shell.

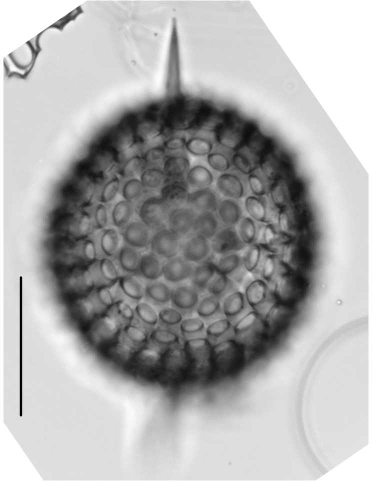

unknown bipolar actinommid 14

Actinommidae (stylosphaerid)

Undescribed

Records: 29

This species has small, framed pores, and thin, conical spines. Medullary shell is relatively large compared to cortical.

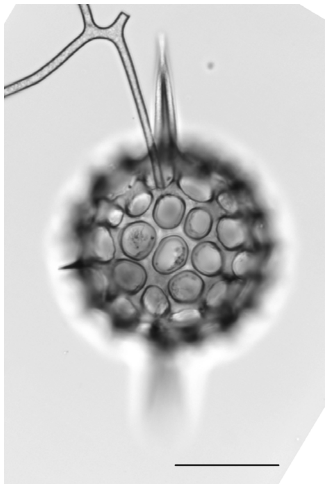

unknown bipolar actinommid 15

Actinommidae (stylosphaerid)

Undescribed

Records: 19

This species is defined by its spherical shape and large, irregular pores. It differs from Stylosphaera sp A (Kamikuri) in that it lacks a medullary shell.

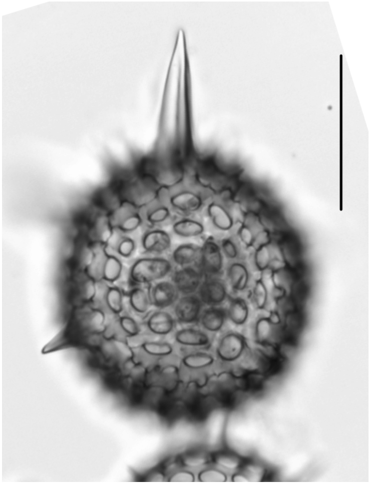

unknown bipolar actinommid 17

Actinommidae (stylosphaerid)

Undescribed

Records: 7

This species has a rough exterior, and small, irregular pores. No medullary shell is present. This species has two strong bladed spines, and several secondary bladed spines throughout the shell exterior.

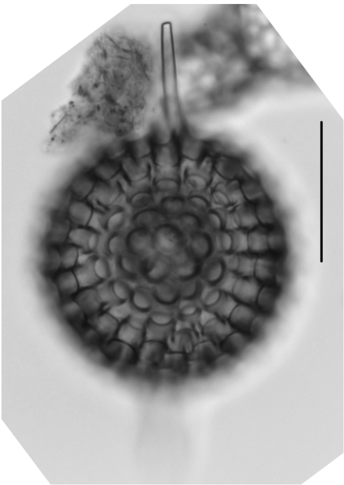

unknown bipolar actinommid 18

Actinommidae (stylosphaerid)

Undescribed

Records: 14

This species has a thick, spherical cortical shell, medullary shell, and conical shells. Differs from Stylosphaera sp A (Kamikuri) in that pores are smaller relative to shell dimensions. Differs from Stylacontarium bispiculum cf in that overall shape is spherical rather than slightly oval, and has a medullary shell.

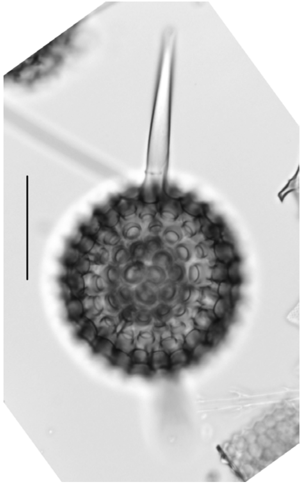

unknown bipolar actinommid 19

Actinommidae (stylosphaerid)

Undescribed

Records: 27

This species resembles Druppatractus hastatus, but has an overall more rounded shape, without the signature flattened sides of D. hastatus. Medullary shell is also more spherical and better developed.

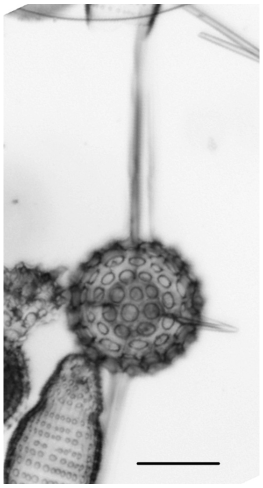

unknown bipolar actinommid 20

Actinommidae (stylosphaerid)

Undescribed

Records: 30

This species is unique for its six bladed spines, two of which are consistently stronger and longer than the rest. It is relatively large, with thick cortical shell, large medullary shell, and rough exterior.

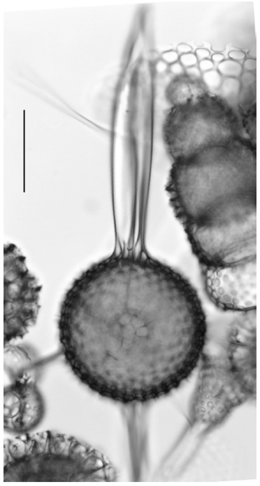

unknown bipolar actinommid 21

Actinommidae (stylosphaerid)

Undescribed

Records: 23

This species has a distinctive medullary shell, which lacks a definite shape and is similar to that of Saturnalis circularis. However, this species is clearly bipolar, with two very strong bladed spines, and several smaller bladed and conical spines. Cortical shell is thick, with small framed pores.

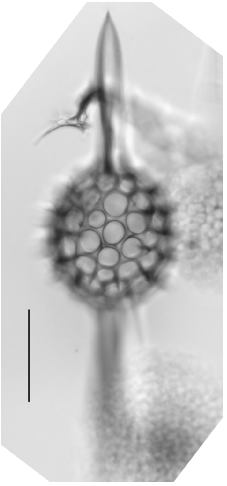

unknown bipolar actinommid 22

Actinommidae (stylosphaerid)

Undescribed

Records: 11

This species has an oval cortical shell, with many short spines. No medullary shells present. Bipolar spines are robust and bladed.

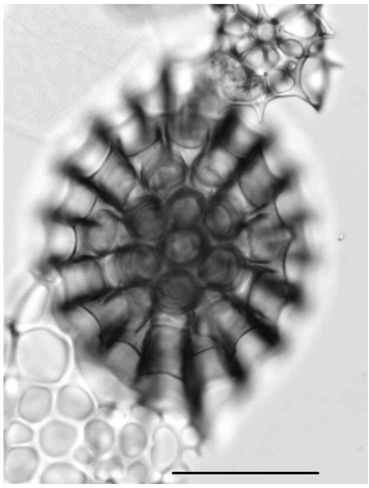

unknown bipolar actinommid 23

Actinommidae (stylosphaerid)

Undescribed

Records: 12

This species has distinct inward-pointing pores, which are very thick and smooth. Medullary shell and bipolar spines present. This species may be related to Stylatractus neptunus, but its pore orientation and smoothness differentiates it.

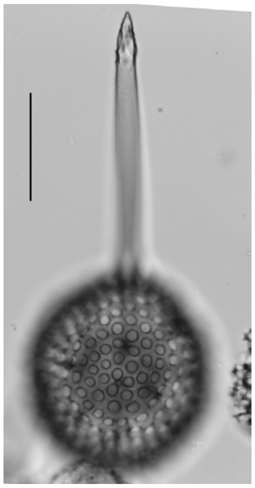

unknown bipolar actinommid 3

Actinommidae (stylosphaerid)

Undescribed

Records: 12

This species has slight constrictions where inner spines from the medullary shell meets the cortical shell. The single long spine has a rough tip. This species is also thin-walled, such that the medullary shell is visible while the cortical shell is in focus. Overall cortical and medullary shell appearance is similar to Stylatractus universus transparum, but differs in that it has only one major spine.

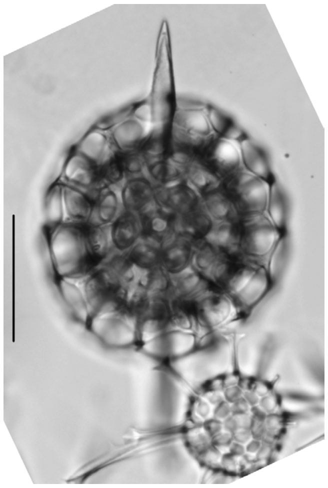

unknown bipolar actinommid 4

Actinommidae (stylosphaerid)

Undescribed

Records: 12

This species has a very thin cortical shell with large pores, strong outer medullary shell, and an inner medullary shell is present. There is approximately the same amount of space between cortical and medullary shells, which differentiates this species from the others.

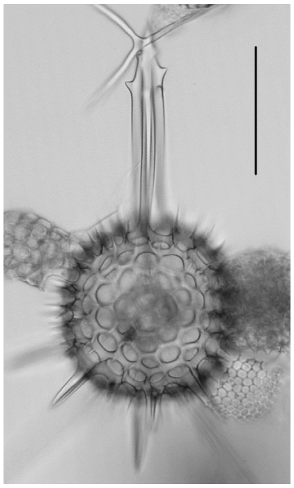

unknown bipolar actinommid 5

Actinommidae (stylosphaerid)

Undescribed

Records: 32

This species is unique for its squarish cortical shell shape, and barbed and thorny main bladed spine. Opposite this main spine are an array of smaller bladed spines. Pores are large and thorny on the cortical shell, and a medullary shell is present.

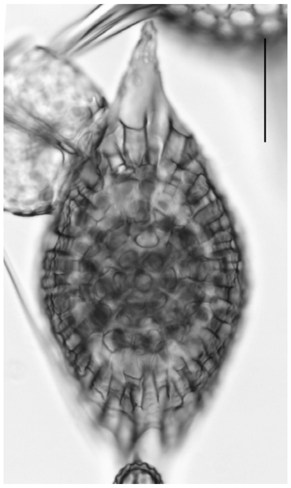

unknown stylatractid sp 2

Actinommidae (stylosphaerid)

Undescribed

Records: 10

This species is differentiated from Stylatractus santaeannae in having a more compressed shape, whereby the cortical shell blends smoothly into the thick, bladed bipolar spines. It also differs in that it has smaller, more irregular pores on the cortical shell.

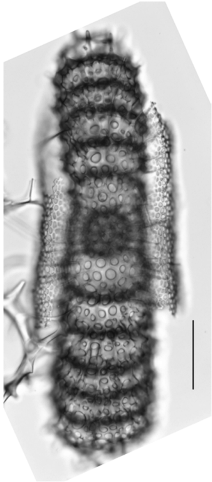

Diartus hughesi
Coccodiscidae
(Campbell & Clark) Sanfilippo et al., 1985
Records: 40

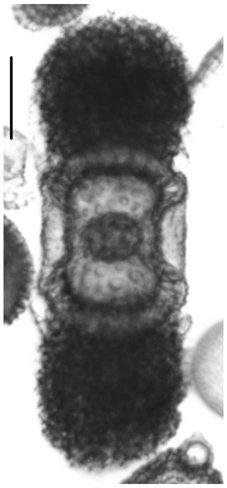

Diartus petterssoni
Coccodiscidae
(Riedel & Sanfilippo) Sanfilippo et al., 1985
Records: 35

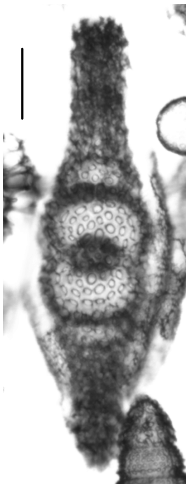

Didymocyrtis antepenultima
Coccodiscidae
(Riedel & Sanfilippo) Sanfilippo et al., 1985
Records: 34

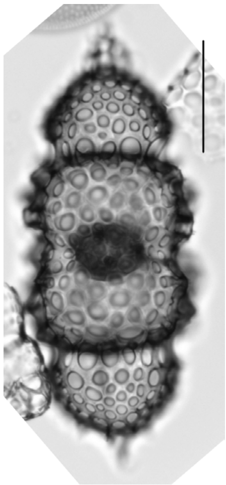

Didymocyrtis avita
Coccodiscidae
(Riedel) Sanfilippo & Riedel, 1980
Records: 78

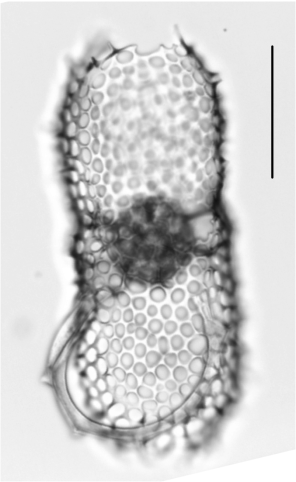

Didymocyrtis bassani
Coccodiscidae
(Carnevale) Sanfilippo & Riedel, 1980
Records: 13

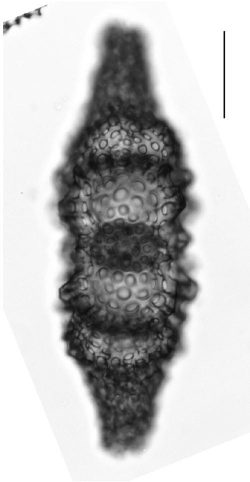

Didymocyrtis laticonus
Coccodiscidae
(Riedel) Sanfilippo & Riedel, 1980
Records: 71

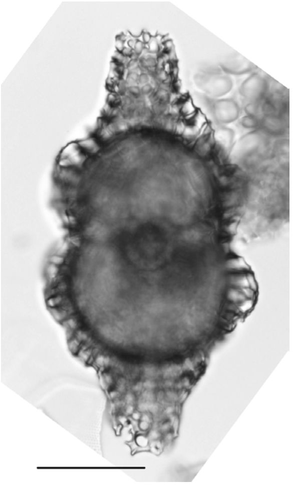

Didymocyrtis mammifera
Coccodiscidae
(Haeckel) Sanfilippo & Riedel, 1980
Records: 7

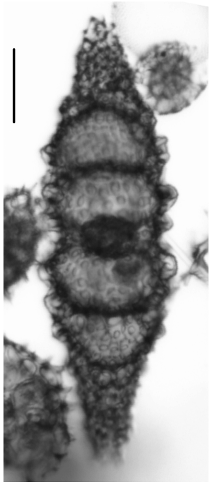

Didymocyrtis penultima
Coccodiscidae
(Riedel) Sanfilippo & Riedel, 1980
Records: 35

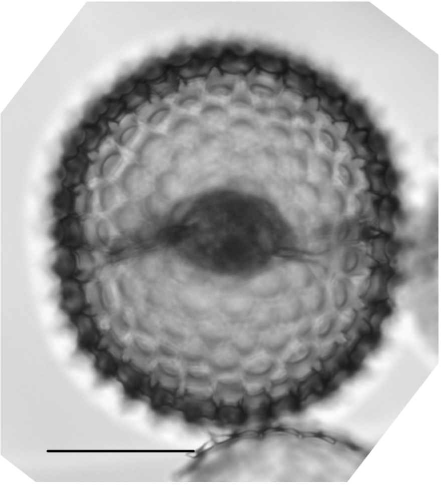

Didymocyrtis prismatica
Coccodiscidae
Haeckel, 1887
Records: 18

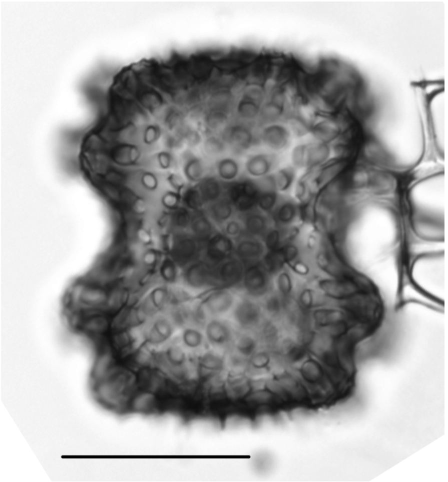

Didymocyrtis sp 1
Coccodiscidae
Undescribed
Records: 19

This species has strong plikae, tilted pores, and has no tubes or lobes preserved. May be a partial skeleton of another Didymocyrtis species, but all specimens appear to be con-specific.

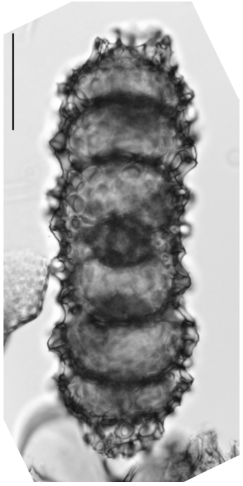

Didymocyrtis sp 2
Coccodiscidae
Undescribed
Records: 11

This species has small plikae, elongated main shell, and at least two large lobes on either side of the shell.

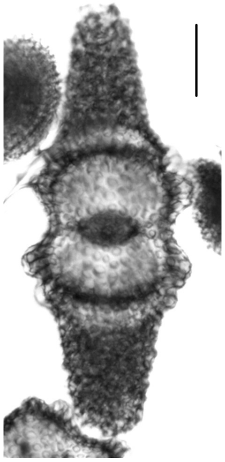

Didymocyrtis sp 3
Coccodiscidae
Undescribed
Records: 43

This species differs from Didymocyrtis laticonus in that it has an oval, lens-shaped medullary shell. It also tends to have a narrower set of lobes at the top and bottom of the main shell.

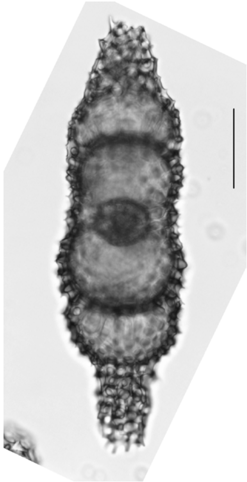

Didymocyrtis sp 4
Coccodiscidae
Undescribed
Records: 40

This species differs from Didymocyrtis laticonus in that it lacks strong plikae. It differs from Didymocyrtis antepenultima in that the first set of lobes outside the main shell are thicker, and tend to be slightly different sizes.

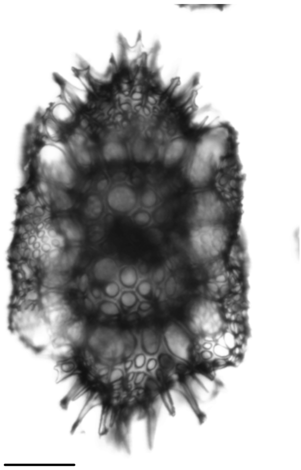

Didymocyrtis tetrathalamus
Coccodiscidae
(Haeckel) Sanfilippo & Riedel, 1980
Records: 71

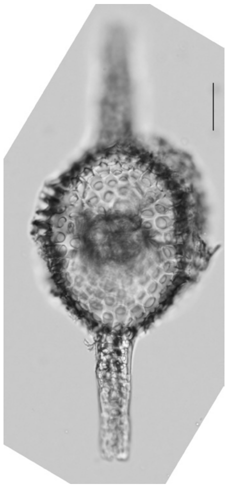

Didymocyrtis tubaria
Coccodiscidae
(Haeckel) Sanfilippo & Riedel, 1980
Records: 21

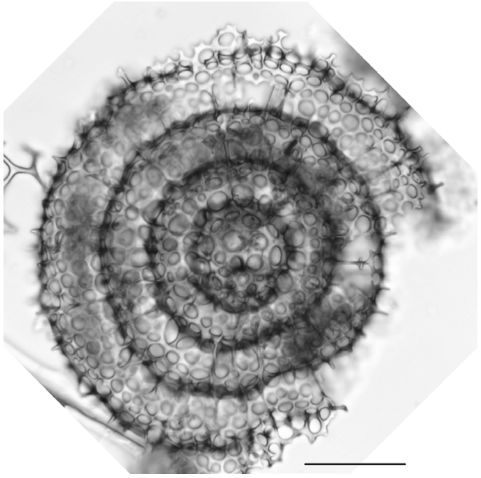

Excentrococcus annulatus
Heliodiscidae
Dumitrica, 1978
Records: 33

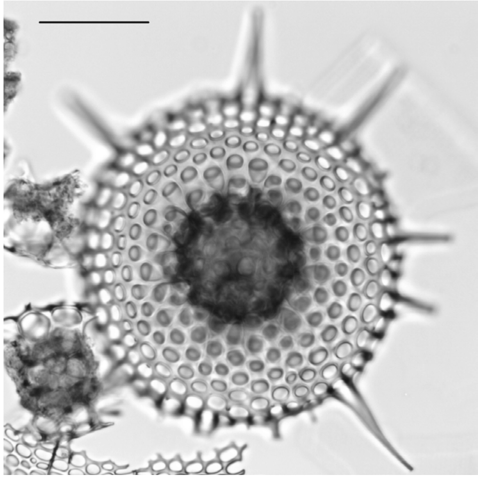

Heliodiscus asteriscus
Heliodiscidae
Haeckel, 1887
Records: 87

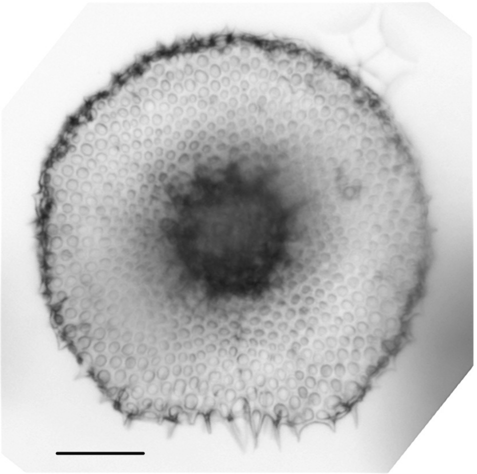

Heliodiscus riedeli
Heliodiscidae
Dumitrica, 2019
Records: 5

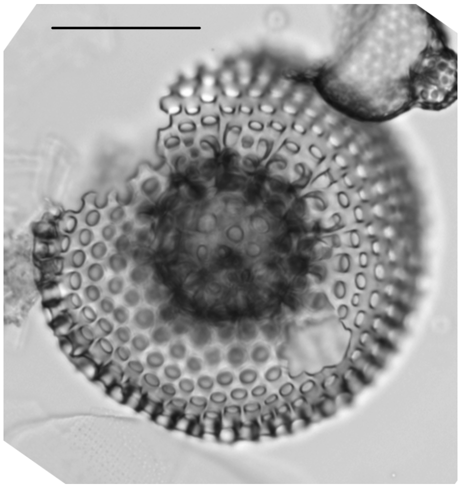

unknown heliodiscid sp 1

Heliodiscidae

Undescribed

Records: 26

This species differs from Heliodiscus asteriscus in that it has a smooth cortical shell exterior, without any radial spines.

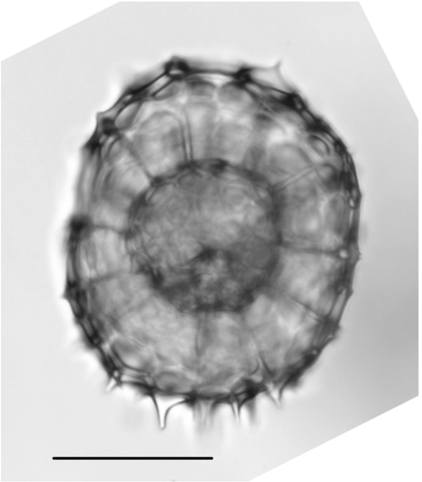

unknown heliodiscid sp 3

Heliodiscidae

Undescribed

Records: 40

This species differs from Heliodiscus asteriscus in that it has larger, more irregular pores with spines, and the cortical shell is relatively small compared to the size of the medullary shell. Overall outline varies from oval to quadrate.

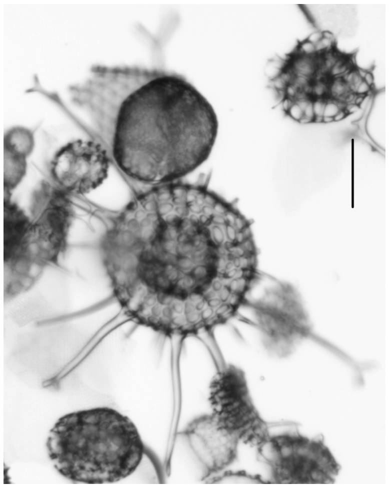

unknown heliodiscid sp 4

Heliodiscidae

Undescribed

Records: 16

This species has very long, branching conical spines around the perimeter of the cortical shell, and the medullary shell is relatively large compared to the size of the cortical shell. These features differentiate it from Heliodiscus asteriscus. Relatively thin cortical shell wall and small pores differentiate it from unknown heliodiscid sp 3.

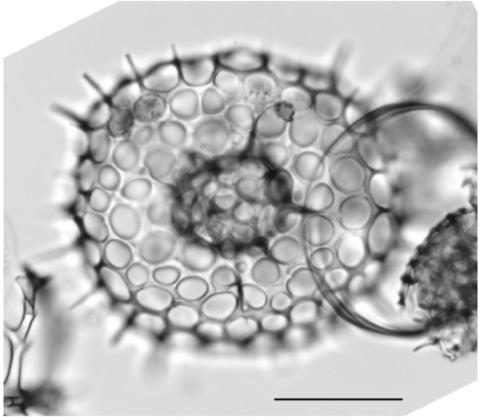

unknown heliodiscid sp 5

Heliodiscidae

Undescribed

Records: 78

This species resembles Heliodiscus asteriscus, but differs in that pores on the cortical shell are large, and not regularly arranged. Differs from unknown heliodiscid sp 3 in that medullary shell is smaller relative to cortical shell.

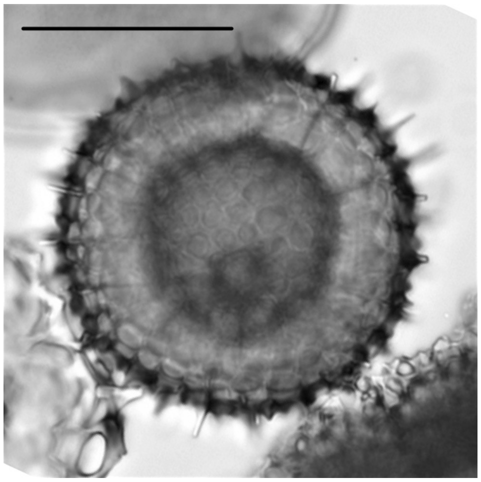

unknown heliodiscid sp 6

Heliodiscidae

Undescribed

Records: 13

This species has a very large outer medullary shell compared to the inner medullary shell, and spherical cortical shell.

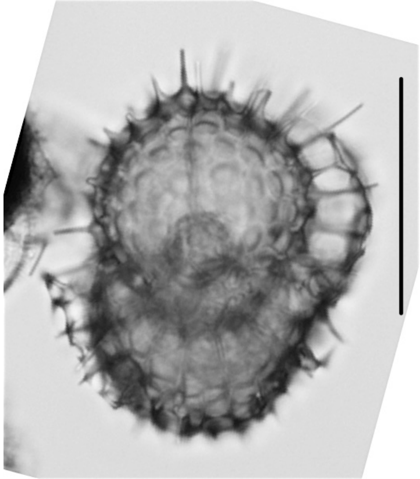

Dipylissa bensoni

Litheliidae

Dumitrica, 1988

Records: 52

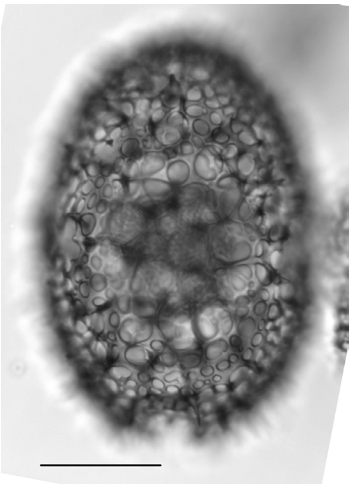

Larcopyle buetschlii

Litheliidae

Dumitrica, 1988

Records: 322

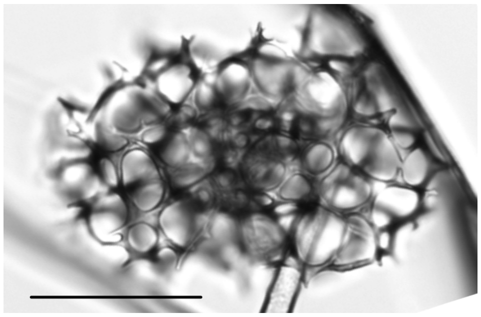

Larcopyle cervicornis

Litheliidae

Haeckel, 1887

Records: 54

sensu Zhang and Suzuki 2017

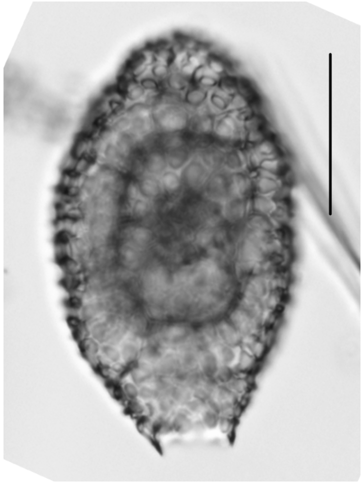

Larcopyle eccentricum

Litheliidae

Lazarus et al., 2005

Records: 19

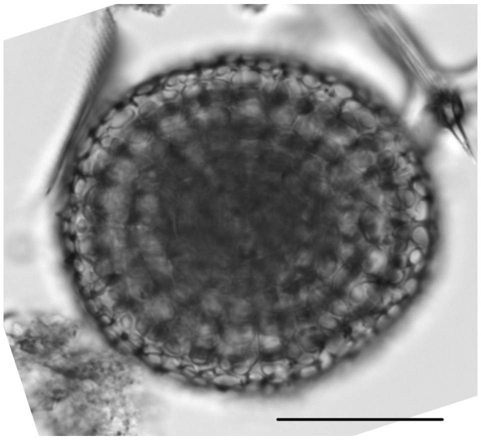

Larcopyle hayesi

Litheliidae

(Chen) Lazarus et al., 2005

Records: 101

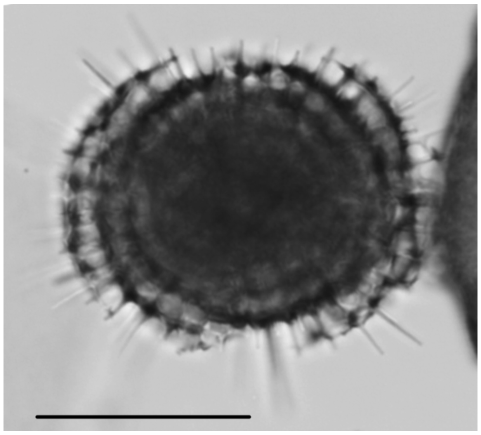

Larcopyle hayesi cf

Litheliidae

Undescribed

Records: 46

This species differs from Larcopyle hayesi in that it has narrower whorls.

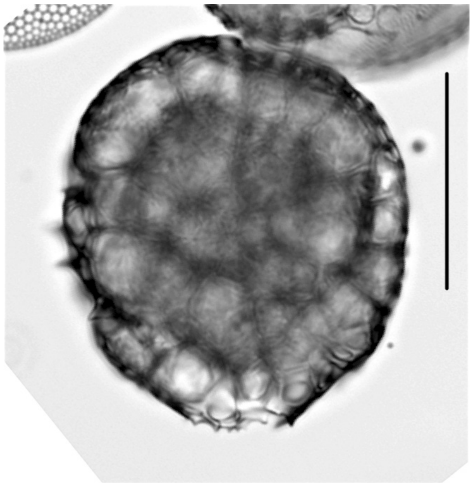

Larcopyle labrinthusa

Litheliidae

Lazarus et al., 2005

Records: 91

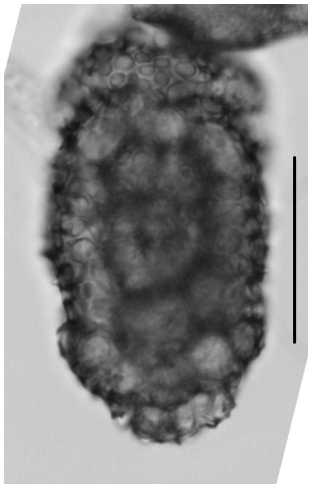

Larcopyle labrinthusa cf

Litheliidae

Undescribed

Records: 15

This species differs from L. labrinthusa in that its overall shape is more elongated.

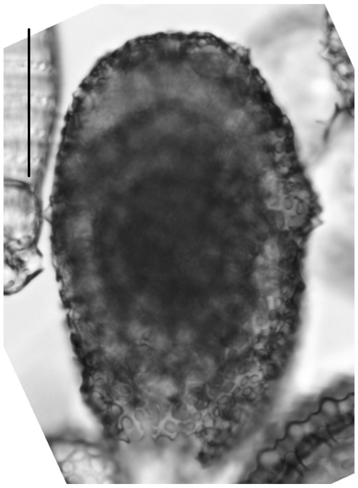

Larcopyle nebulum

Litheliidae

Lazarus et al., 2005

Records: 7

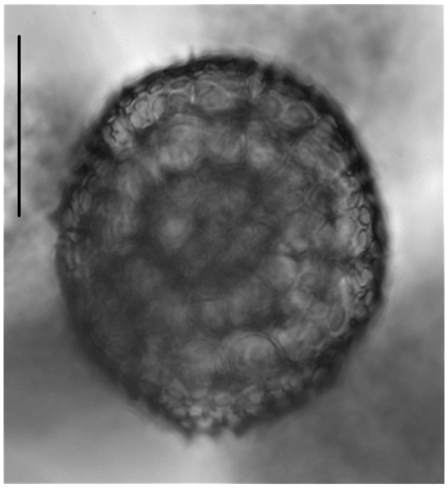

Larcopyle peregrinator

Litheliidae

Lazarus et al., 2005

Records: 36

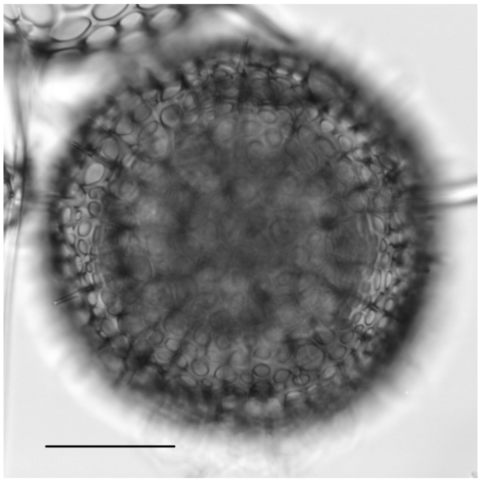

Larcopyle peregrinator cf

Litheliidae

Undescribed

Records: 91

Differs from Larcopyle peregrinator in that its overall shape is circular, and it lacks a clear pylome.

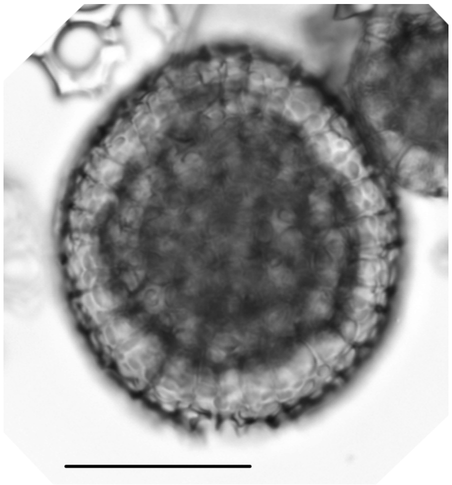

Larcopyle peregrinator cf sp 2

Litheliidae

Undescribed

Records: 11

Differs from Larcopyle peregrinator and Larcopyle peregrinator cf sp 2 in that whorls are tighter in the center of the shell, but the outermost whorl is slightly wider.

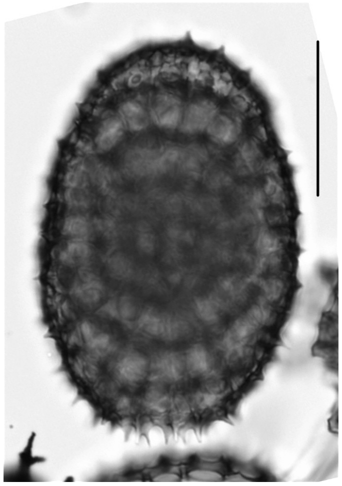

Larcopyle polyacantha

Litheliidae

(Campbell & Clark) Lazarus et al., 2005

Records: 127

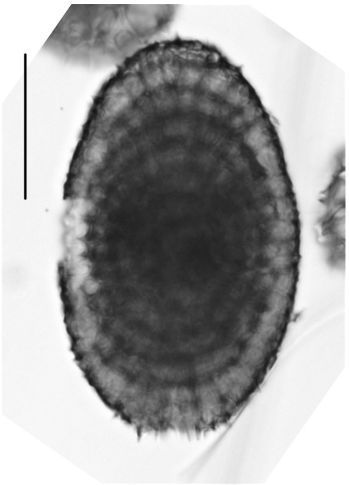

Larcopyle polyacantha fm 2
Litheliidae
Undescribed
Records: 77

This species differs from Larcopyle polyacantha in that the whorls are much more tightly spaced.

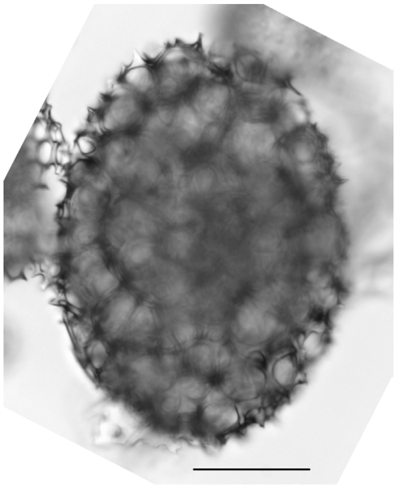

Larcopyle sp 1
Litheliidae
Undescribed
Records: 74

This species differs from Larcopyle polyacantha in that it has looser, more irregular whorls. It has more internal bars than Larcopyle cervicornis.

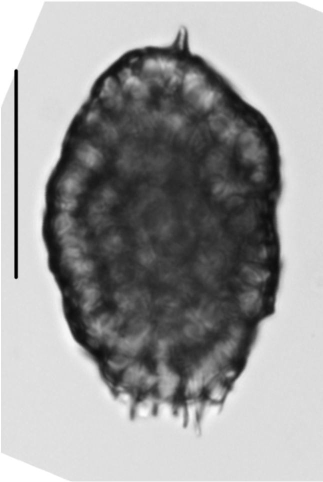

Larcopyle weddellium
Litheliidae
Lazarus et al., 2005
Records: 186

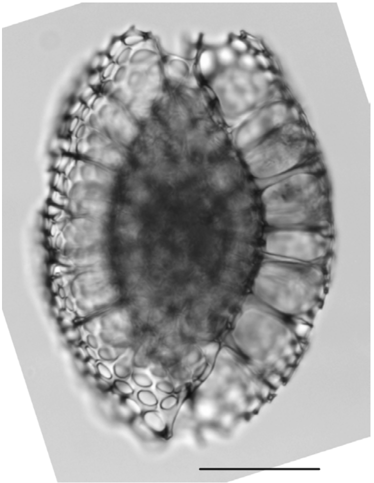

Larcospira moschkovskii
Litheliidae
Kruglikova, 1978
Records: 92

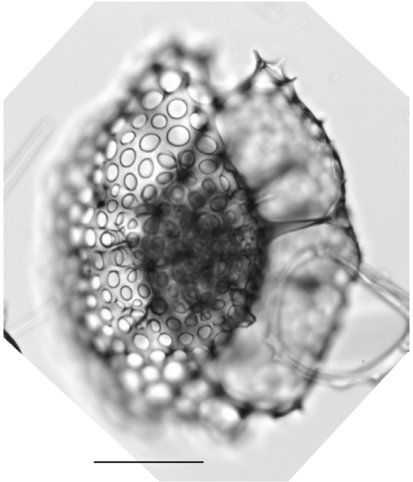

Larcospira quadrangula
Litheliidae
Haeckel, 1887
Records: 62

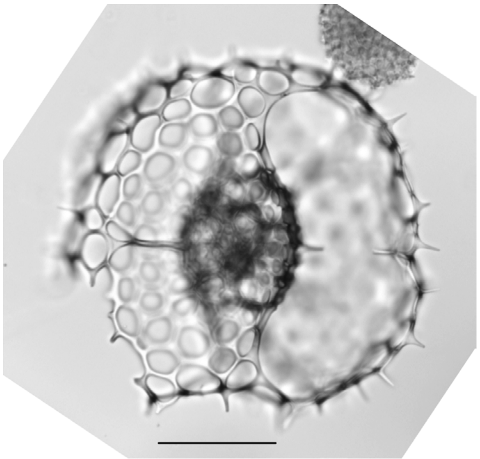

Larcospira teres
Litheliidae
Zhang & Suzuki, 2017
Records: 54

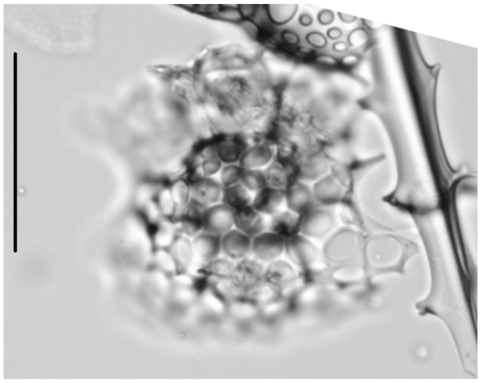

Larcospira tetragonicentrum
Litheliidae
Zhang & Suzuki, 2017
Records: 13

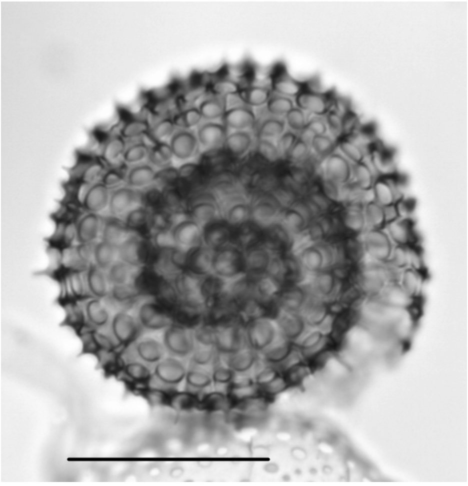

Lithelius klingi
Litheliidae
Kamikuri, 2010
Records: 39

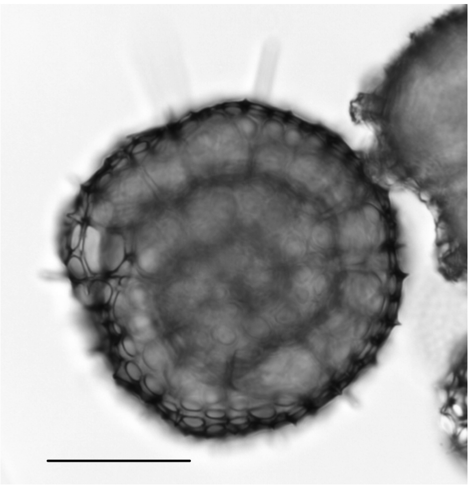

Lithelius klingi cf
Litheliidae
Undescribed
Records: 68

More lightly silicified, and irregular spiral shape than the species described by Kamikuri, 2010.

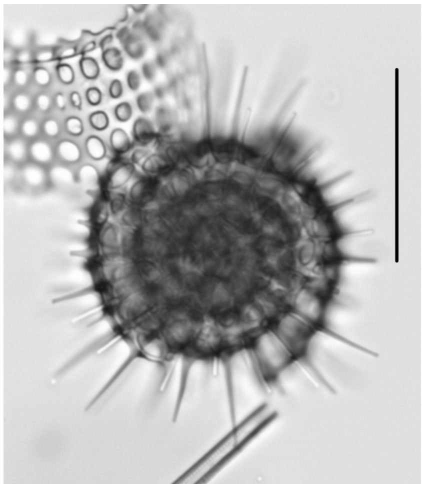

Lithelius minor
Litheliidae
Jørgensen, 1900
Records: 47

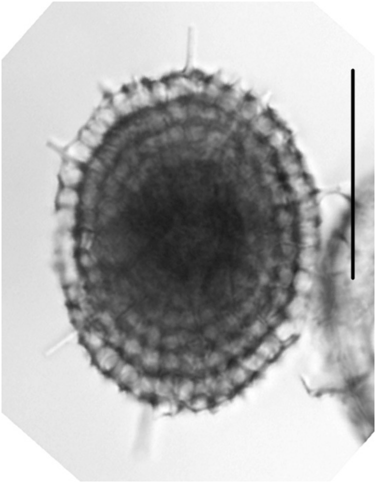

Lithelius minor cf
Litheliidae
Undescribed
Records: 30

Differs from Lithelius minor in that its shape is more oval, and internal structure is not double-whorled. May be synonymous with Stylodictya? sp. of Takahashi 1991.

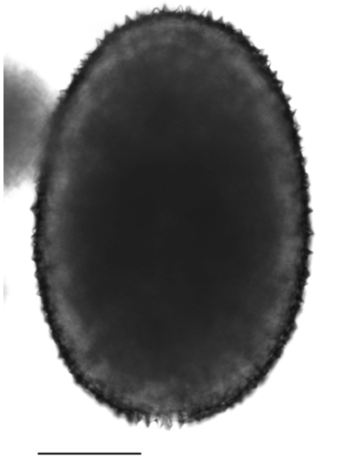

Lithelius sp A Kamikuri
Litheliidae
Kamikuri, 2017
Records: 11

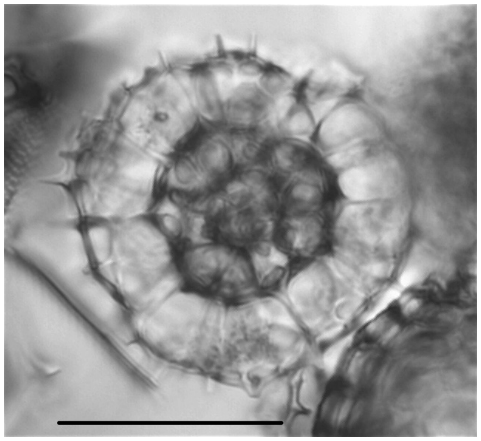

Qiuripylolena chikuchik
Litheliidae
Zhang & Suzuki, 2017
Records: 41

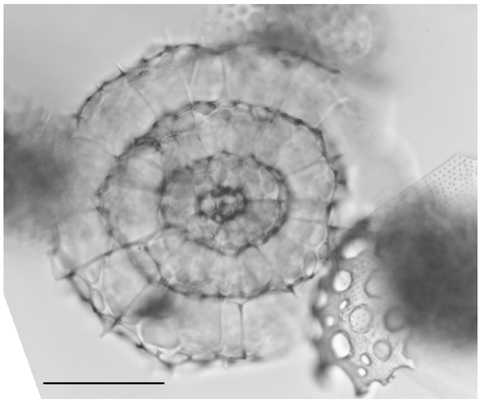

Qiuripylolena multiconcentrica
Litheliidae
Zhang & Suzuki, 2017
Records: 61

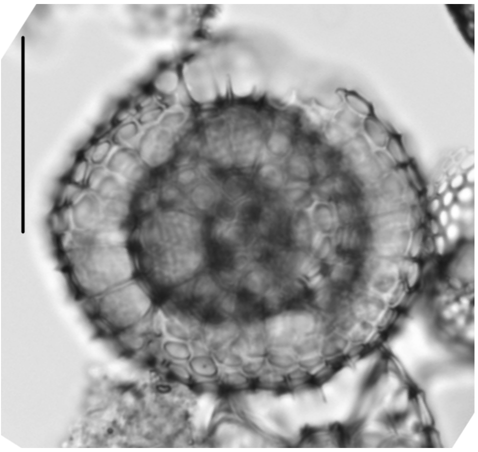

Qiuripylolena pompon
Litheliidae
Zhang & Suzuki, 2017
Records: 62

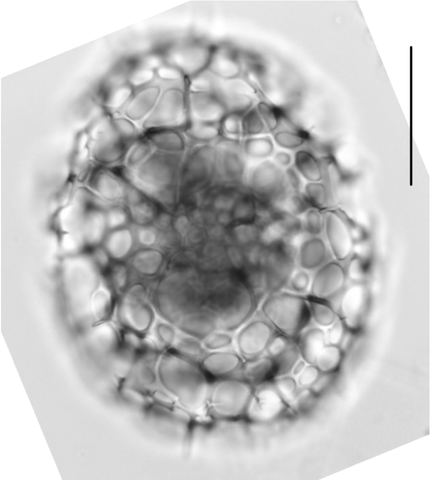

Sphaerolarnacillium cochleatum
Litheliidae
Zhang & Suzuki, 2017
Records: 57

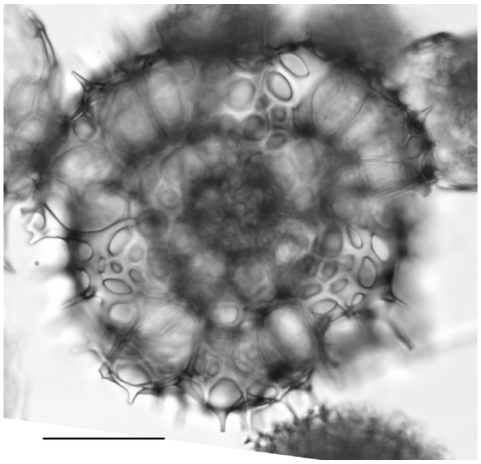

Sphaerolarnacillium cochleatum cf
Litheliidae
Undescribed
Records: 56

Differs from Sphaerolarnacillium cochleatum in that it has a more clearly triangular inner whorl.

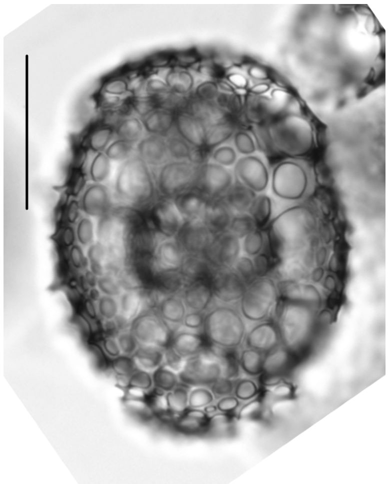

Sphaerolarnacillium exactum
Litheliidae
Zhang & Suzuki, 2017
Records: 34

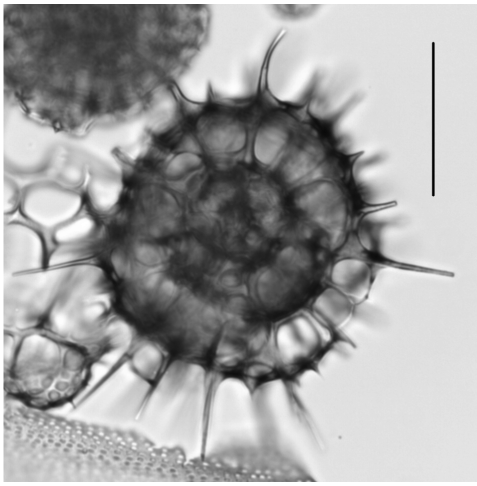

Sphaerolarnacillium tanzhiyuani

Litheliidae

Zhang & Suzuki, 2017

Records: 83

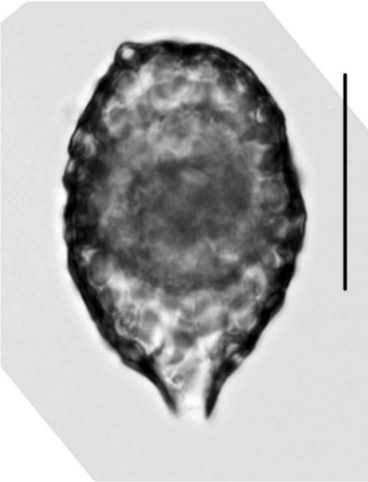

Sphaeropyle? sp 1

Litheliidae

Undescribed

Records: 53

This species is small, and is distinguished from Sphaeropyle? sp 2 by its horizontally-oriented, circular medullary shell.

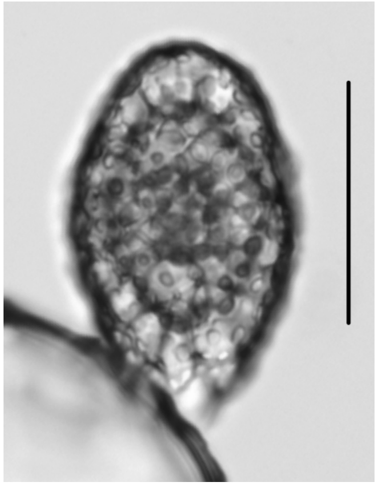

Sphaeropyle? sp 2

Litheliidae

Undescribed

Records: 45

Like Sphaeropyle? sp 1, except that its medullary shell is vertically oriented (same direction as the cortical shell), and this medullary shell is oval-shaped rather than round.

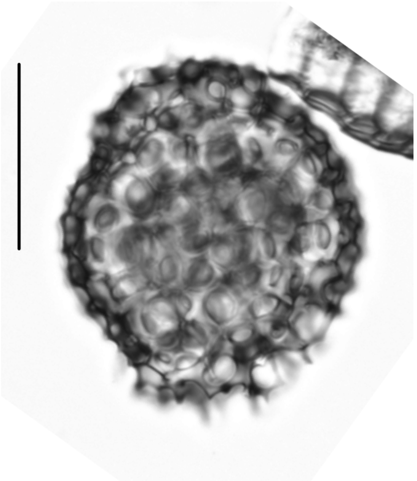

Sphaeropyle? sp 3

Litheliidae

Undescribed

Records: 11

This species differs from Sphaeropyle? sp 1 and Spheropyle? sp 2 in that its cortical shell is more robust and has a bulge in the middle.

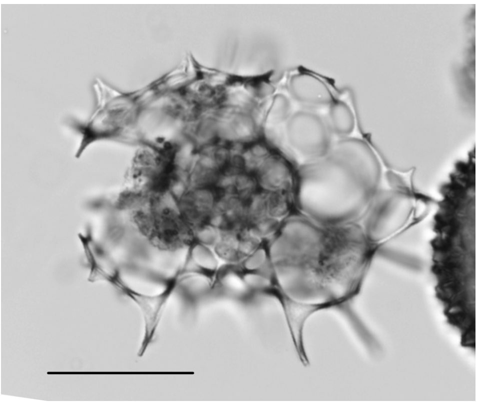

Sphaeropylolena laxa

Litheliidae

Zhang & Suzuki, 2017

Records: 9

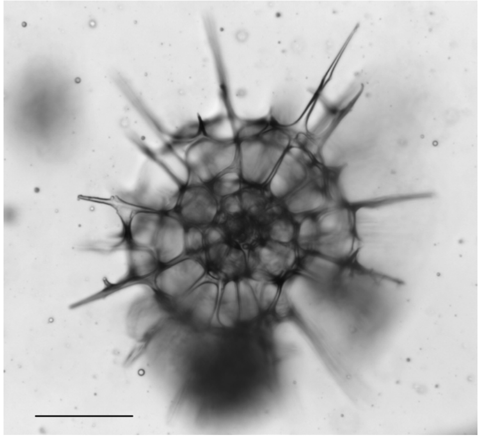

Sphaeropylolena tenellisinosa

Litheliidae

Zhang & Suzuki, 2017

Records: 24

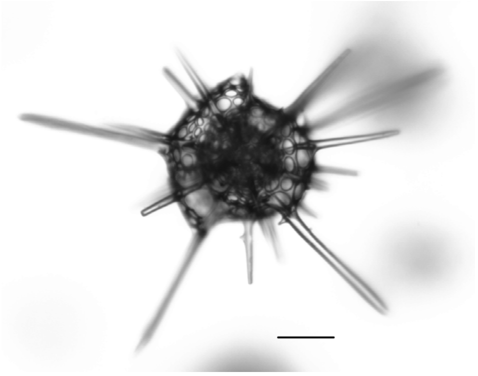

Streblacantha circumtexta

Litheliidae

Jørgensen, 1905

Records: 64

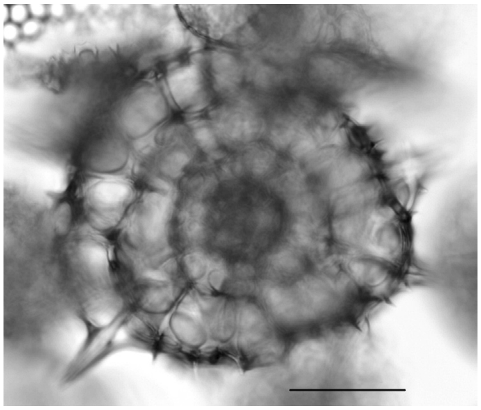

Streblacantha circumtexta cf

Litheliidae

Undescribed

Records: 7

This species is similar to Streblacantha circum-texta but with bladed spines, and a more defined internal structure.

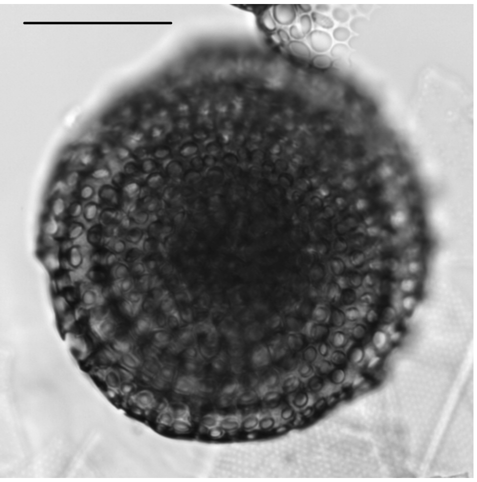

unknown litheliid sp 10

Litheliidae

Undescribed

Records: 35

This species resembles Stylodictya multispina but consistently has a thick layer of spongy material covering the center of the shell, such that inner whorls are not visible.

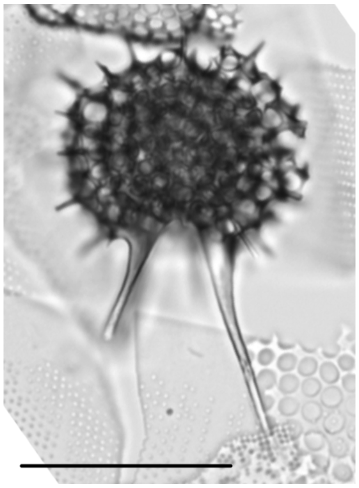

unknown litheliid sp 11

Litheliidae

Undescribed

Records: 8

This species has a distinct set of two, long conical spines with an indentation in the main body of the shell between them, forming a V shape.

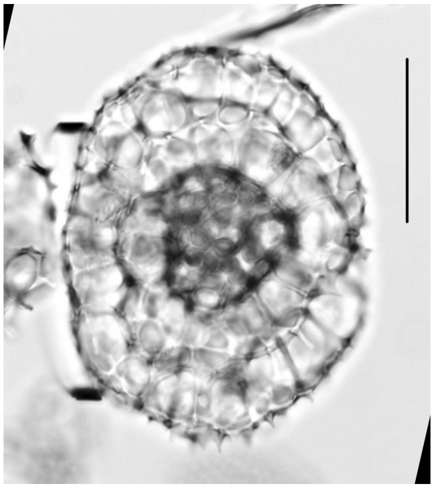

unknown litheliid sp 12

Litheliidae

Undescribed

Records: 68

This species has a tightly-whorled inner shell and loosely-whorled outer shell. Inner part is typically more heavily-silicified.

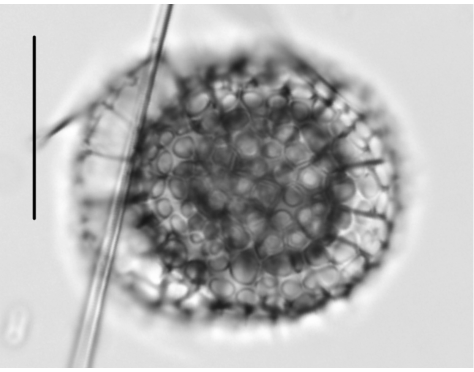

unknown litheliid sp 13

Litheliidae

Undescribed

Records: 14

This species differs from Qiuripylolena multicon-centrica in that it has irregular whorls, rather than clear oval-shaped whorls.

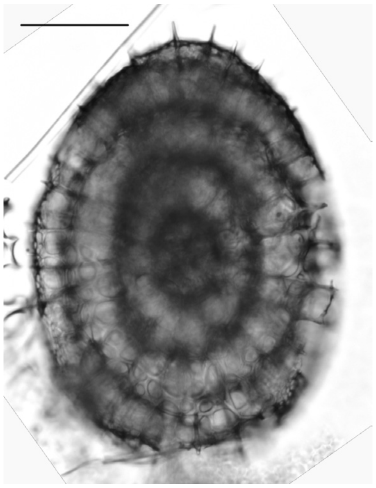

unknown litheliid sp 14

Litheliidae

Undescribed

Records: 15

This species differs from Larcopyle polyacantha in that it has oval-shaped inner shells that are oriented in the same direction as the outer shell.

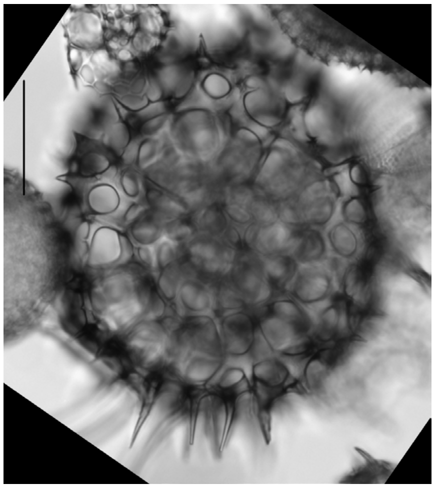

unknown litheliid sp 15

Litheliidae

Undescribed

Records: 7

This species has thick bars and large pores, with external spines concentrated on one size of the outer shell.

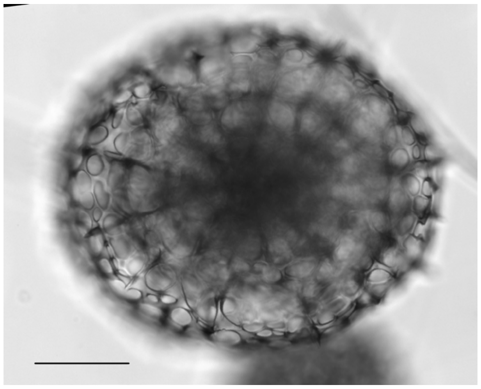

unknown litheliid sp 16

Litheliidae

Undescribed

Records: 8

This species is unique for having radial bars within the shell that lead from the outermost whorl to the interior.

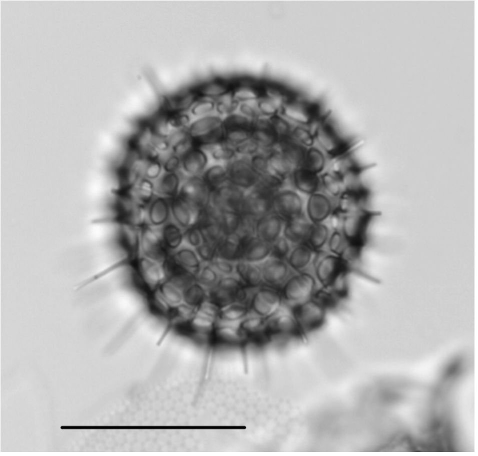

unknown litheliid sp 17

Litheliidae

Undescribed

Records: 13

This species differs from Lithelius minor in that it is single-whorled rather than double-whorled.

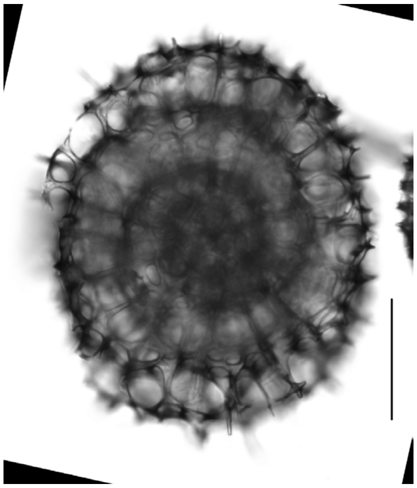

unknown litheliid sp 18

Litheliidae

Undescribed

Records: 4

This species is large and double-whorled, differentiating it from Qiuripylolena multiconcentrica, and unknown litheliid sp 4.

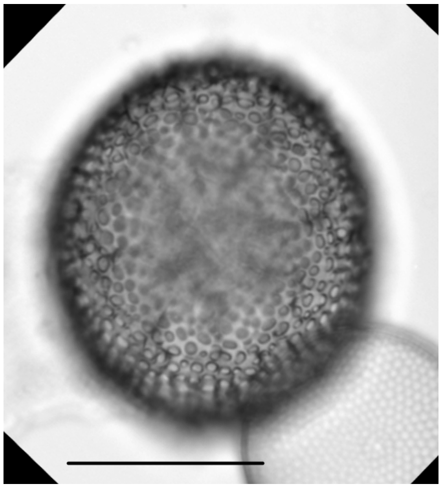

unknown litheliid sp 19

Litheliidae

Undescribed

Records: 10

This species is unique for having a lumpy exterior surface with small pores, and no inner structure present (or preserved?).

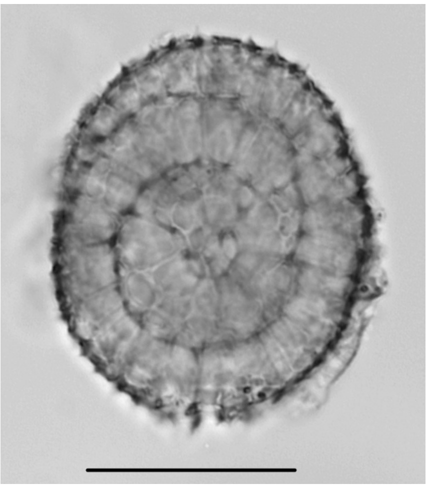

unknown litheliid sp 2

Litheliidae

Undescribed

Records: 12

This species is loosely double-whorled, differing from unknown litheliid sp 18 in that it is significantly smaller, with smaller pores and is more lightly silicified.

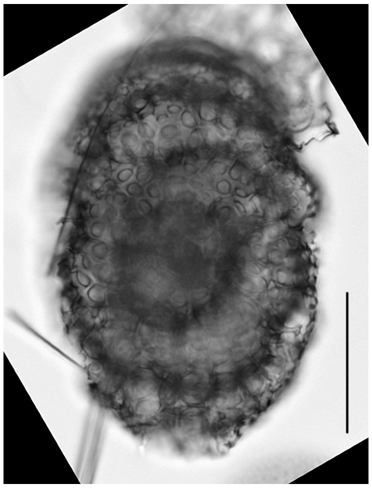

unknown litheliid sp 20

Litheliidae

Undescribed

Records: 12

This species has large, irregular internal whorls. It differs from unknown litheliid sp 21 in that it lacks a quadrate inner whorl.

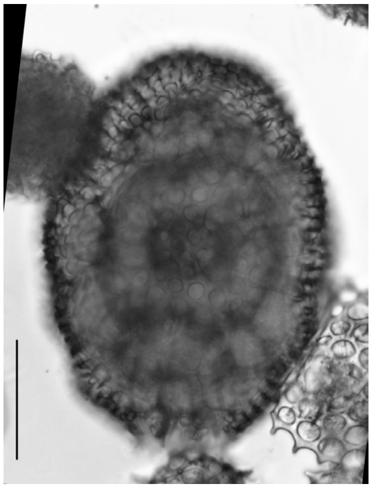

unknown litheliid sp 21

Litheliidae

Undescribed

Records: 78

This species is larger than unknown litheliid sp 20, and has a distinct square-shaped second inner whorl, with a larnacea structure at its center. Shell exterior has relatively small pores and is highly irregular. Differs from unknown litheliid sp 7 in that it has looser whorls and tends to be wider.

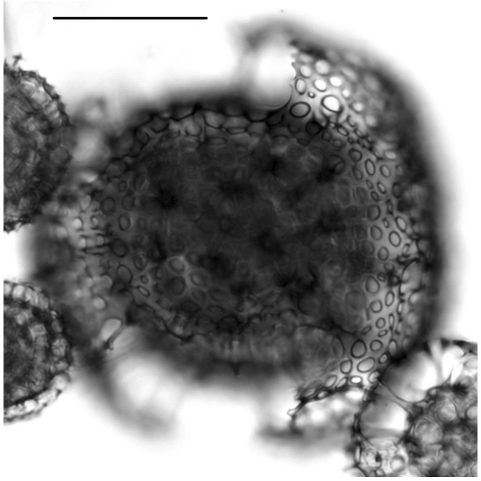

unknown litheliid sp 22

Litheliidae

Undescribed

Records: 70

This species is large, with dense internal bars, relatively small pores, and a sub- rounded shape. The bars leave distinct black spots, and small shell constrictions on the exterior and on internal whorls.

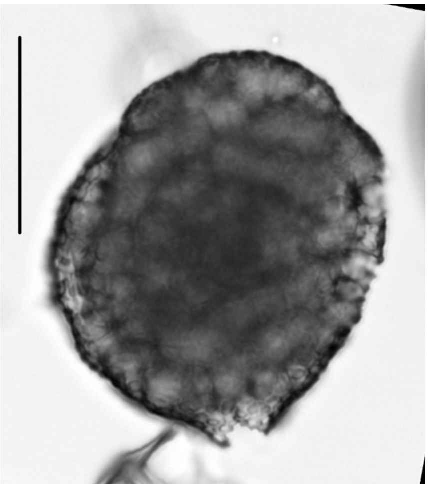

unknown litheliid sp 23

Litheliidae

Undescribed

Records: 12

This species has very tight and irregular whorls, bordering on a spongy structure. Small pylome is usually present.

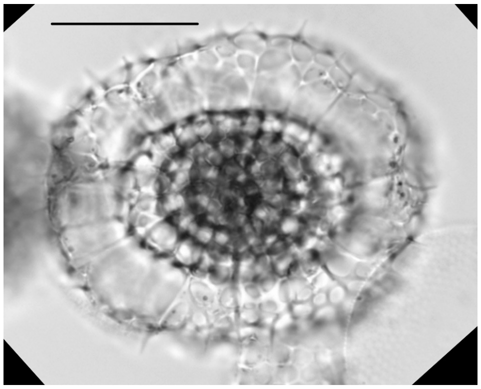

unknown litheliid sp 24

Litheliidae

Undescribed

Records: 13

This species has a tightly-coiled inner section, and an outermost coil that is approximately 4-5 times as wide.

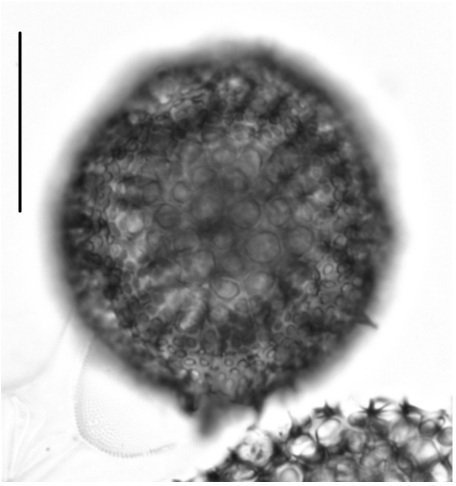

unknown litheliid sp 25

Litheliidae

Undescribed

Records: 5

The internal structure of this species resembles Larcopyle labrinthusa, but differs in that it has a double-spiral and a robust, large-pored, spherical cortical shell with a finely-pored extra layer around it.

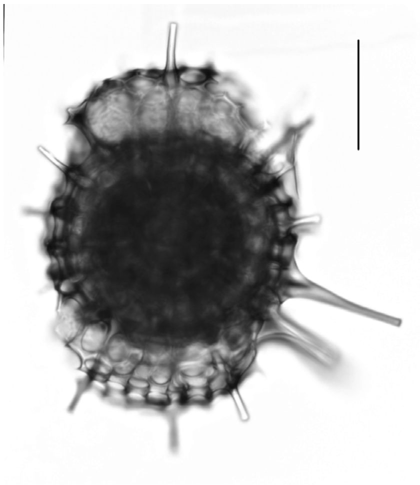

unknown litheliid sp 26

Litheliidae

Undescribed

Records: 5

The shape of this species resembles Sphaerolarnacillium exactum, but differs int hat it lacks a clear larnacea, and instead has more loosely-coiled inner shell structure that is also more heavily-silicified than S. exactum.

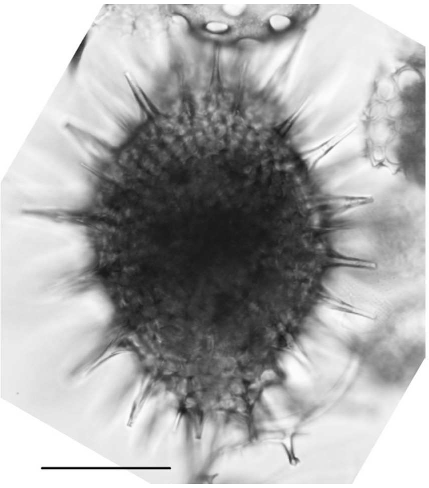

unknown litheliid sp 3

Litheliidae

Undescribed

Records: 36

This species is unique for having a tightly-coil, almost spongy shell structure, an defined oval shape, and strong spines that encompass the whole shell exterior.

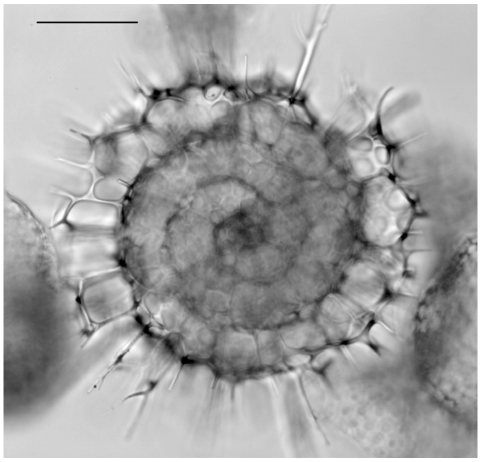

unknown litheliid sp 4

Litheliidae

Undescribed

Records: 19

This species is large and loosely-coiled like unknown litheliid sp 18, but differs in that it is overall circular rather an oval shaped.

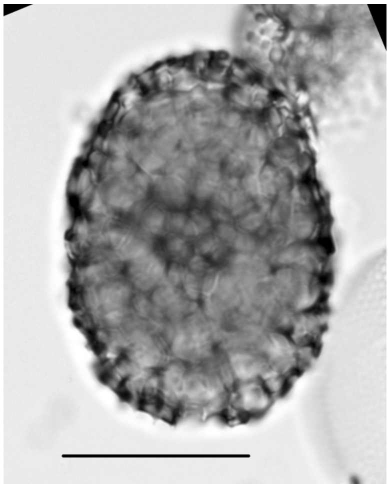

unknown litheliid sp 5

Litheliidae

Undescribed

Records: 54

This species is small, and usually lightly-silicified, with a small inner structure that is thicker that the rest of the shell. Differs from unknown litheliid sp 12 in that it is smaller, usually with smaller pores.

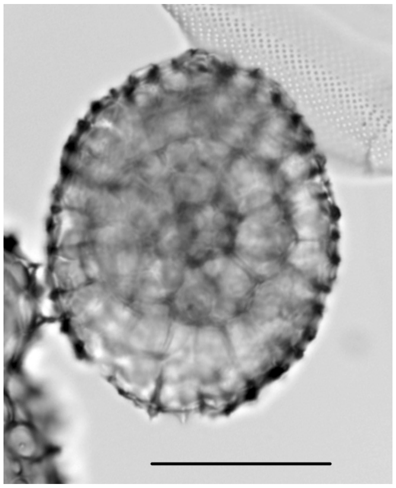

unknown litheliid sp 6

Litheliidae

Undescribed

Records: 86

This species is very loosely-coiled, with only 2-3 sets of internal whorls. Differs from Qiuripylolena multiconcentrica in that internal structures are more irregular. The outermost shell has smaller pores than the internal whorls. It is similar to unknown litheliid sp 12 but typically smaller with fewer whorls.

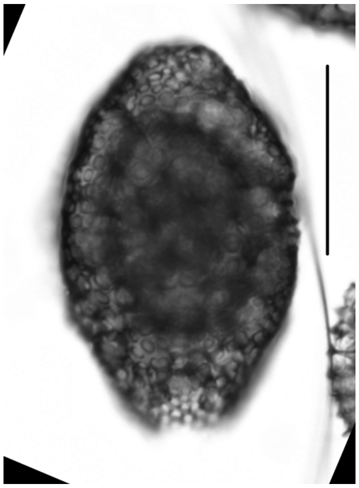

unknown litheliid sp 7

Litheliidae

Undescribed

Records: 58

This species differs from unknown litheliid sp 21 in that it is more tightly whorled, and tends to be more narrow. Outside of shell is lumpy, small-pored, and irregular.

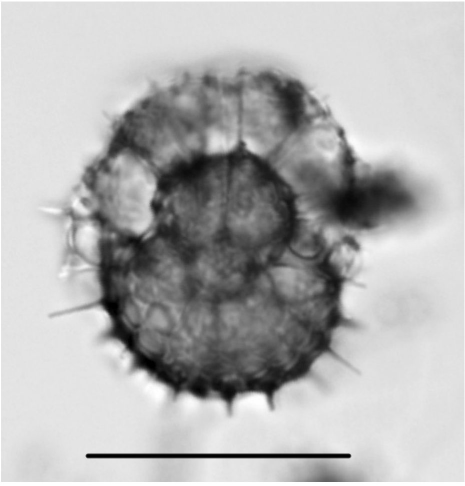

unknown litheliid sp 9

Litheliidae

Undescribed

Records: 27

This species is small, with trilobate inner structure.

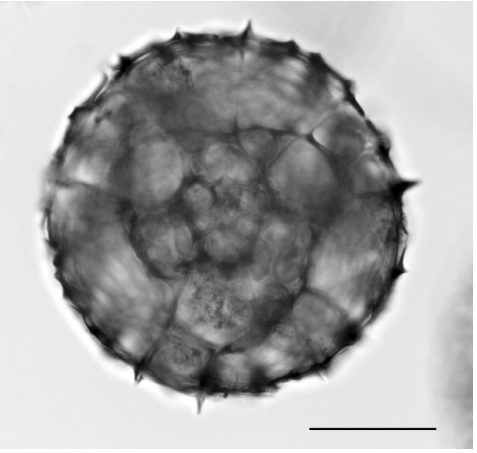

Hexapyle dodecantha

Pyloniidae

Haeckel, 1887

Records: 102

Other generic name: Hexacantha

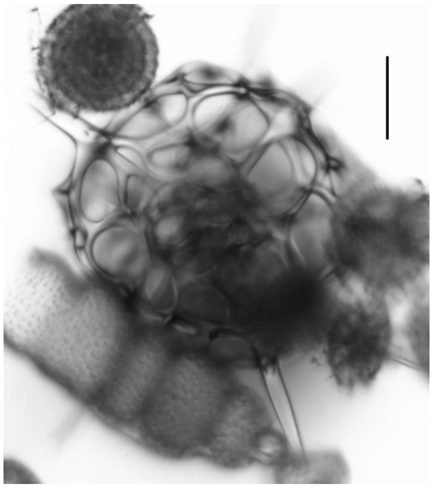

Pentapylonium implicatum

Pyloniidae

Dumitrica, 1991

Records: 7

This species differs from Streblacantha circumtexta in that it is several times larger, with very large pores on its exterior and prominent spines.

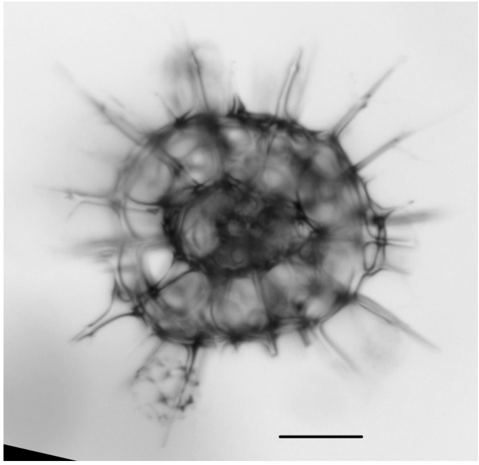

Phorticium multispinum

Pyloniidae

Popofsky, 1912

Records: 38

sensu Zhang and Suzuki 2017

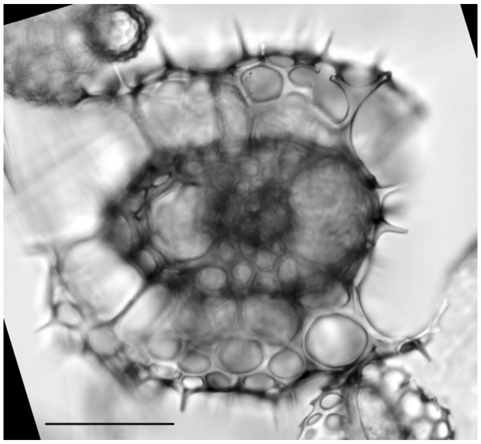

Phorticium polycladum

Pyloniidae

Tan & Tchang, 1976

Records: 81

sensu Zhang and Suzuki 2017

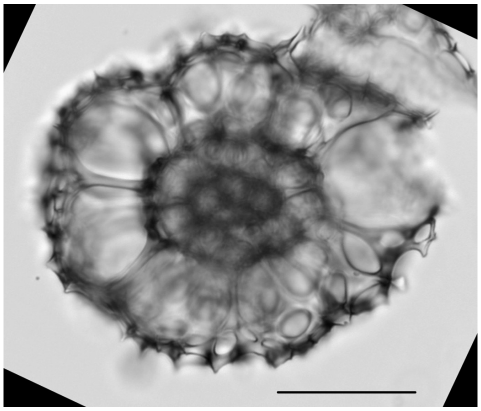

Phorticium scitulum

Pyloniidae

Zhang & Suzuki, 2017

Records: 72

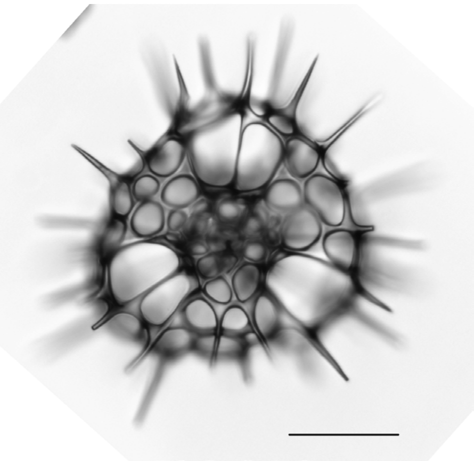

Pylodiscus spinulosus

Pyloniidae

Chen & Tan, 1989

Records: 28

sensu Zhang and Suzuki 2017

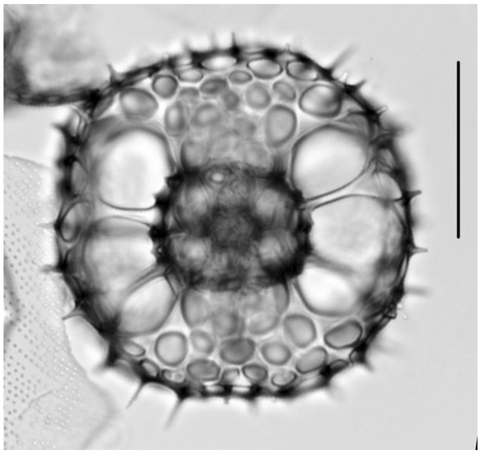

Tetrapyle circularis

Pyloniidae

Haeckel, 1887

Records: 76

sensu Zhang and Suzuki 2017

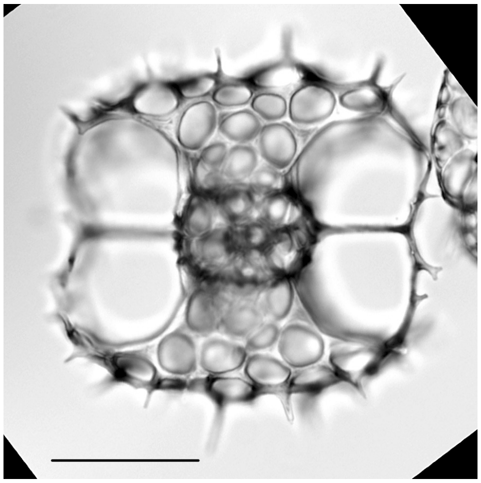

Tetrapyle fruticosa

Pyloniidae

Tan & Chen, 1990

Records: 56

sensu Zhang and Suzuki 2017

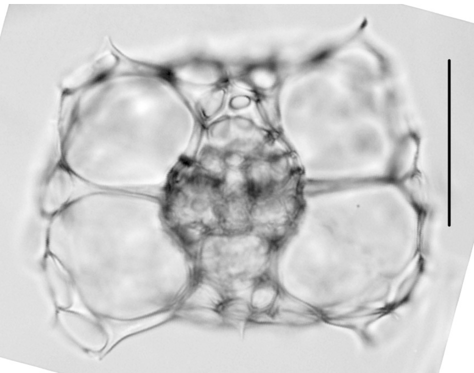

Tetrapyle octacantha

Pyloniidae

Müller, 1859

Records: 58

sensu Zhang and Suzuki 2017

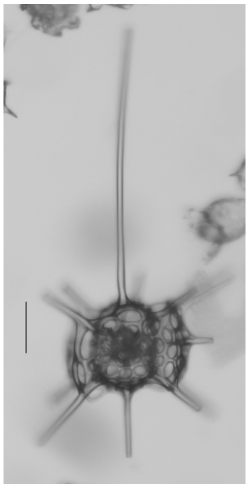

Tetrapyle sp 1

Pyloniidae

Undescribed

Records: 31

This species differs from other Tetrapyle species in that it has a thicker shell overall and longer, more robust spines.

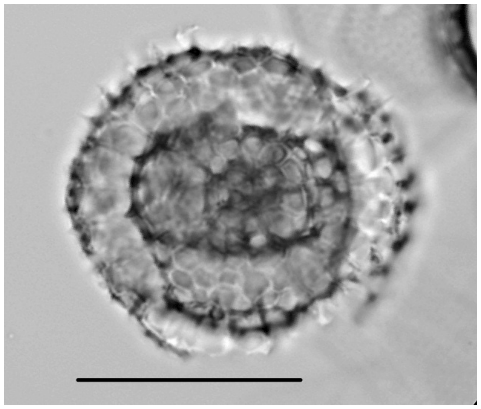

Tholomura hexonia

Pyloniidae

Haeckel, 1887

Records: 39

sensu Zhang and Suzuki 2017

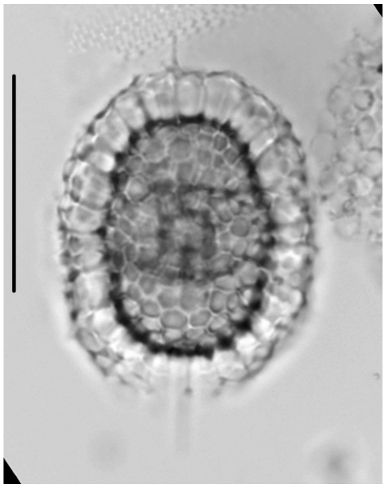

Tholomura hexonia cf

Pyloniidae

Undescribed

Records: 47

This species differs from Tholomura hexonia in that its outermost shell is oriented at 90 degrees to the inner larnacea, rather than in line with it.

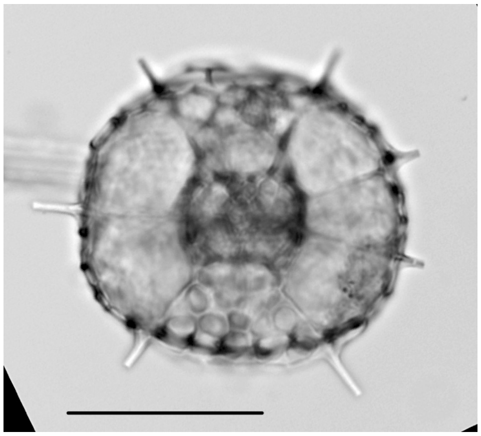

Tholomura pilula

Pyloniidae

Zhang & Suzuki, 2017

Records: 117

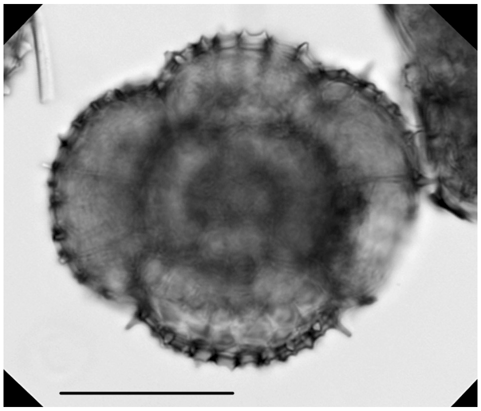

Tholomura polystyla

Pyloniidae

Chen, 1987

Records: 55

sensu Zhang and Suzuki 2017

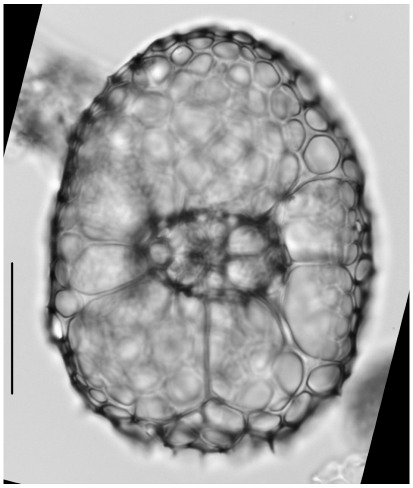

unknown pyloniid sp 1

Pyloniidae

Undescribed

Records: 29

This species has a relatively small inner larnacea structure, and a long, narrow outer shell oriented at 90 degrees to the inner shell.

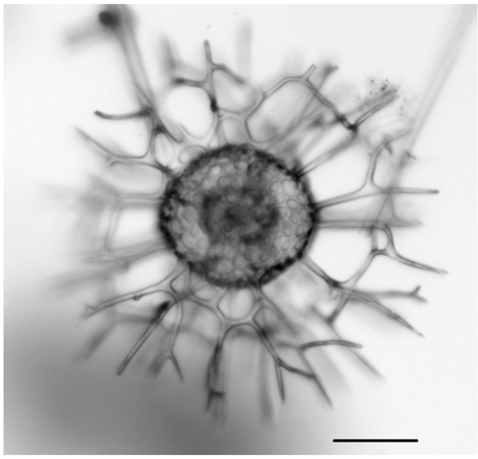

unknown pyloniid sp 10

Pyloniidae

Undescribed

Records: 17

This species is unique for its array of long, dendritic, conical spines all around the shell.

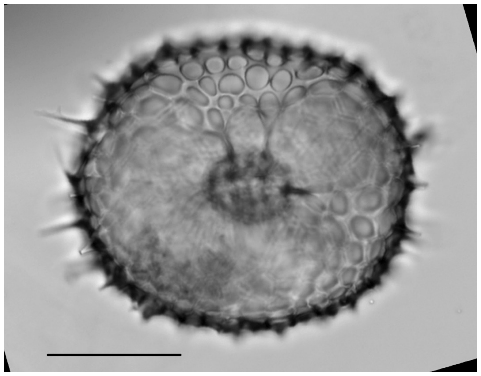

unknown pyloniid sp 11

Pyloniidae

Undescribed

Records: 6

This species differs from unknown pyloniid sp 1 in that the outer shell is aligned in the same direction as the inner larnacea. It differs from unknown pyloniid sp 7 in that the outer shell has smaller pores and less pronounced spines. Differs from Tetrapyle circularis in that inner shell is smaller, and it lacks key internal bars.

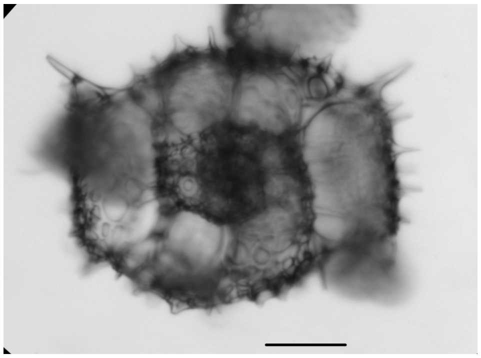

unknown pyloniid sp 12

Pyloniidae

Undescribed

Records: 13

This species is distinct for its large size and open gates.

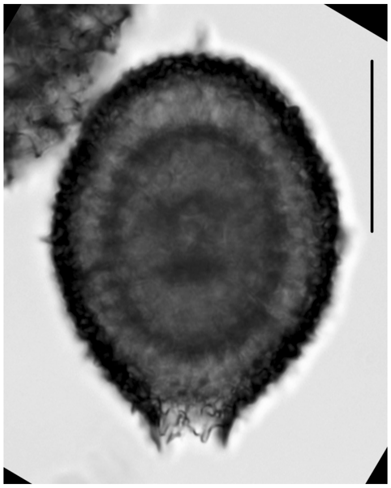

unknown pyloniid sp 13

Pyloniidae

Undescribed

Records: 5

This species differs from Sphaeropyle? sp 2 in that it is larger and more robust. It differs from Tholomura hexonia cf in that is has a more robust cortical shell.

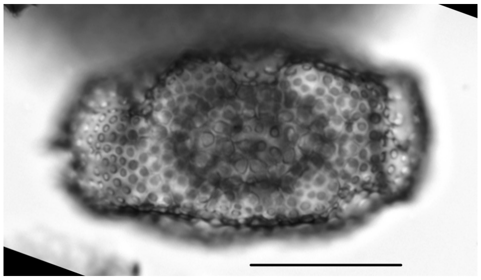

unknown pyloniid sp 14

Pyloniidae

Undescribed

Records: 8

This species has a uniquely narrow and irregular exterior shell with very small pores. Only one specimen has been observed.

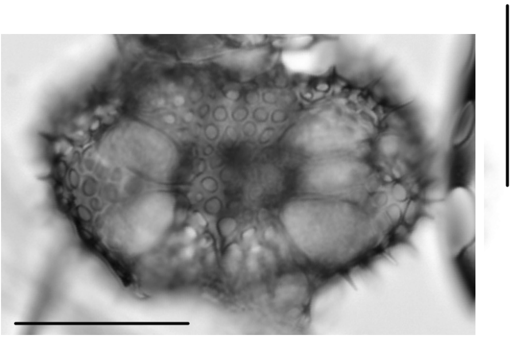

unknown pyloniid sp 15

Pyloniidae

Undescribed

Records: 6

This species is unique for being relatively large, with a wide and irregular exterior shell. Differs from unknown pyloniid sp 7 in that it has a relatively larger inner larnacea.

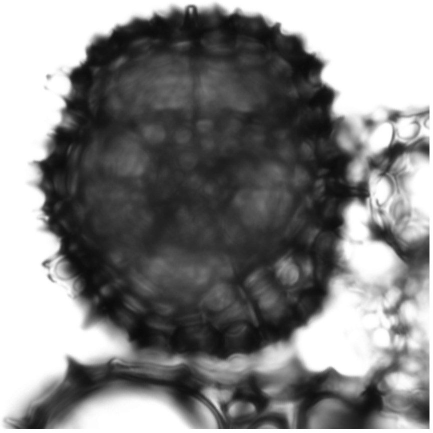

unknown pyloniid sp 16

Pyloniidae

Undescribed

Records: 12

This species has a robust, quadrate, and larged-pored exterior shell, and a wide larnacea shell within.

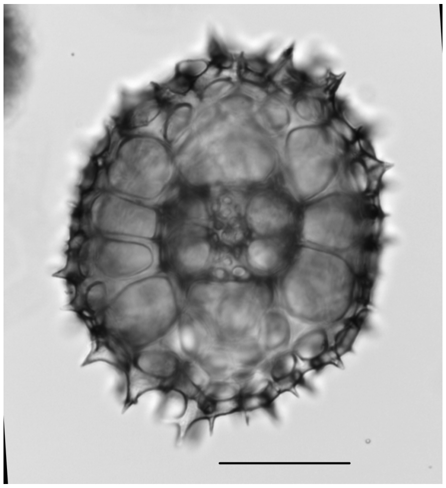

unknown pyloniid sp 3

Pyloniidae

Undescribed

Records: 56

This species differs from unknown pyloniid sp 7 in that its outer shell is oriented 90 degrees from the inner larnacea. It differs from unknown pyloniid sp 1 in that it is smaller, with larger pores and is more heavily silicified.

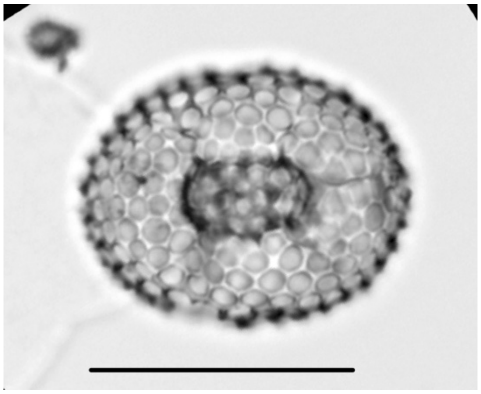

unknown pyloniid sp 5 cf sp 2

Pyloniidae

Undescribed

Records: 18

This species differs from Tholomura pilula in that it has a significantly smaller inner larnacea and lacks prominent spines on shell interior and exterior.

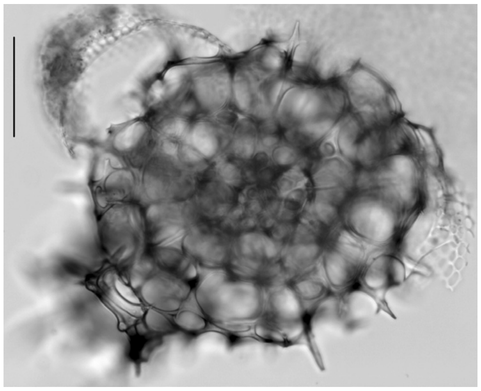

unknown pyloniid sp 6

Pyloniidae

Undescribed

Records: 19

The internal structure of this species is difficult to discern, and the exterior has a thin finely-pored veil. It has spines and irregular, large pores.

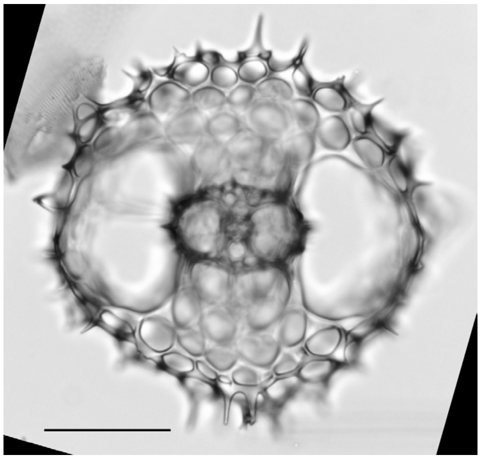

unknown pyloniid sp 7

Pyloniidae

Undescribed

Records: 70

This species differs from unknown pyloniid sp 11 in that it has larger pores on the outer shell, and an array or strong spines. It differs from Tetrapyle circularis in that exterior outline is not as circular, and instead is more vertically elongated.

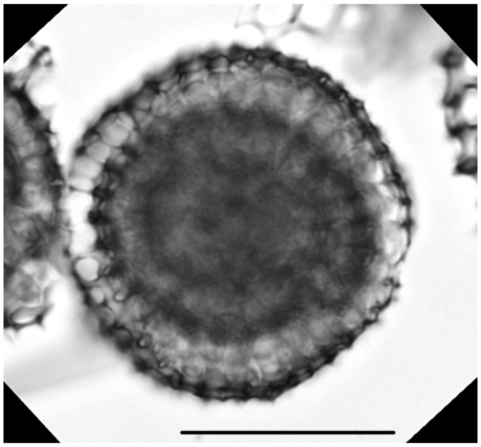

unknown pyloniid sp 9

Pyloniidae

Undescribed

Records: 93

This species is small, with two concentric shells outside of the larnacea.

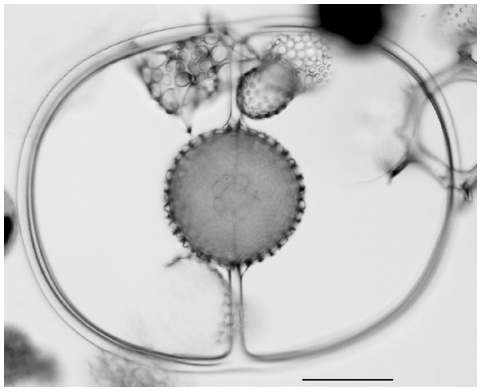

Saturnalis circularis

Saturnalidae

Haeckel, 1887

Records: 23

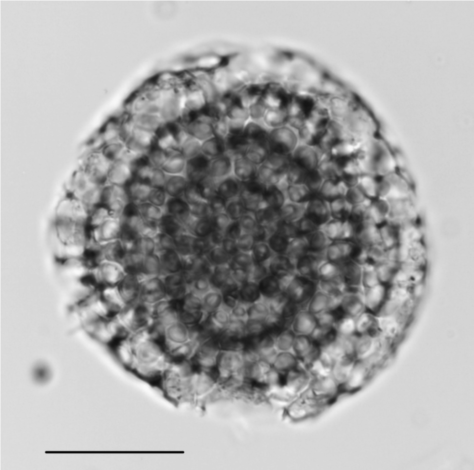

Circodiscus amphitrites
Spongodiscidae (porodiscid)
Zhang & Suzuki, 2017
Records: 6

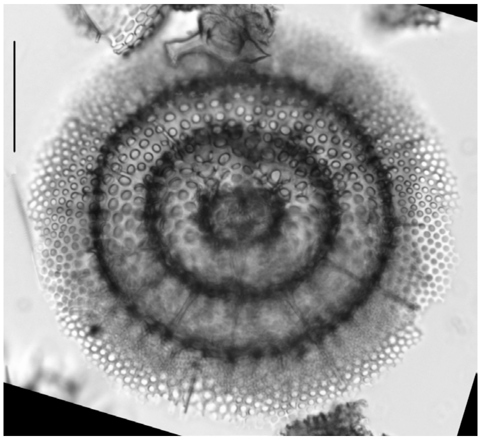

Circodiscus microporus
Spongodiscidae (porodiscid)
(Stohr) Petrushevskaya & Kozlova, 1972
Records: 36

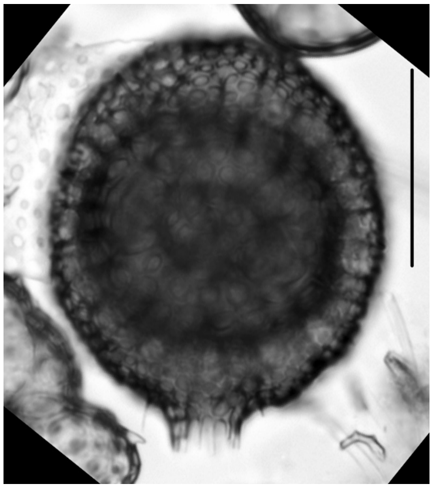

Circodiscus pseudomicroporus
Spongodiscidae (porodiscid)
Zhang & Suzuki, 2017
Records: 12

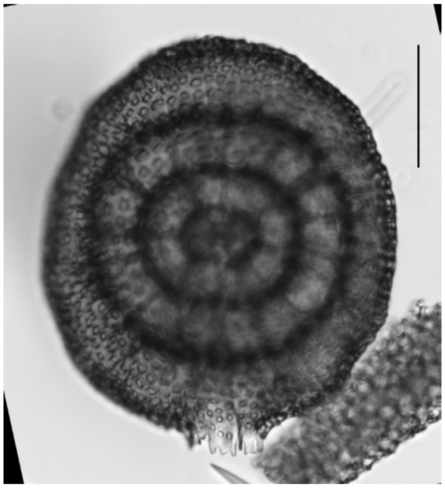

Circodiscus sp 1
Spongodiscidae (porodiscid)
Undescribed
Records: 13

This species differs from Circodiscus microporus in that it has one additional segment/whorl, and a clear termination whereas C. microporus has a more ragged termination. This species also differs from C. microporus in that it has a pylome.

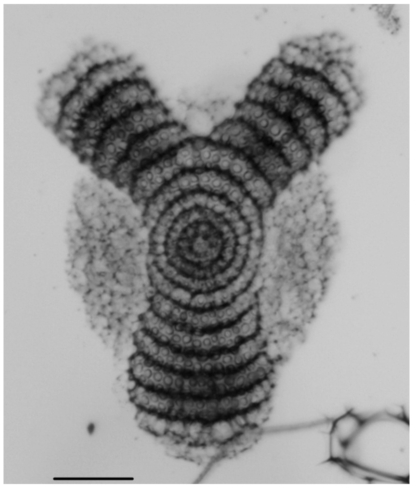

Amphirhopalum ypsilon
Spongodiscidae (spongodiscid)
Haeckel, 1887
Records: 29

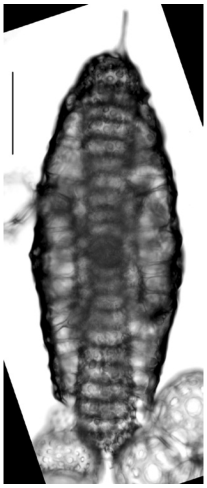

Amphymenium amphistylium
Spongodiscidae (spongodiscid)
Haeckel, 1887
Records: 25

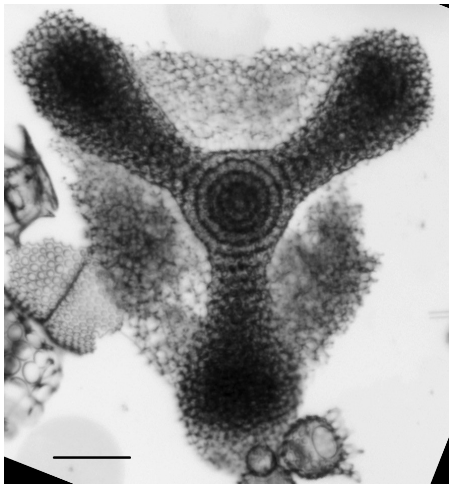

Dictyocoryne profunda
Spongodiscidae (spongodiscid)
Ehrenberg, 1872
Records: 62

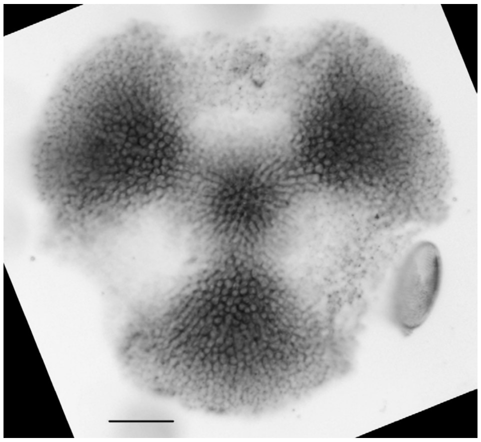

Dictyocoryne truncatum
Spongodiscidae (spongodiscid)
(Ehrenberg) Nigrini & Moore, 1979
Records: 70

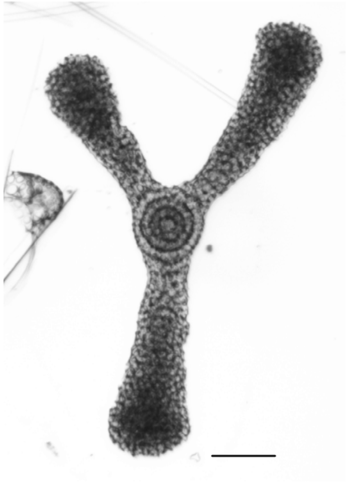

Euchitonia elegans-furcata
Spongodiscidae (spongodiscid)
Ehrenberg, 1860 (genus)
Records: 28
group; listed as valid in Lazarus et al., 2015

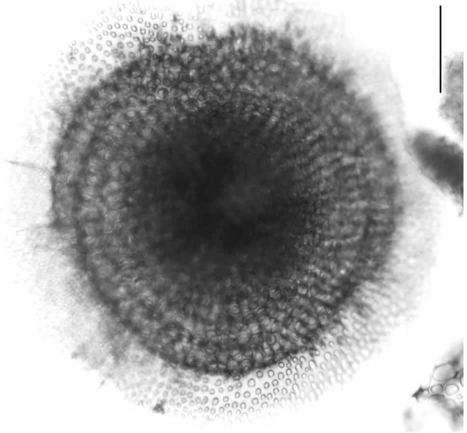

Perichlamydium praetextum
Spongodiscidae (spongodiscid)
Ehrenberg, 1847
Records: 93

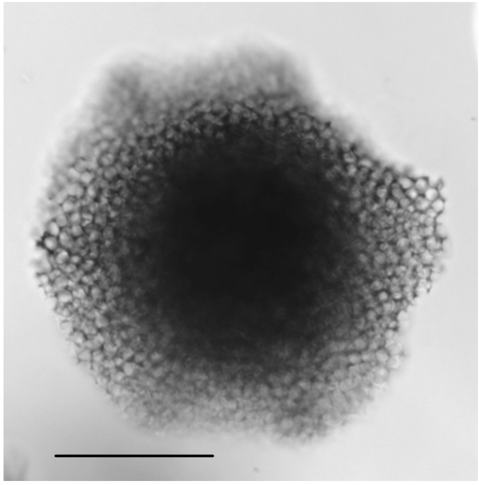

Spongaster pentas
Spongodiscidae (spongodiscid)
Riedel & Sanfilippo, 1970
Records: 20

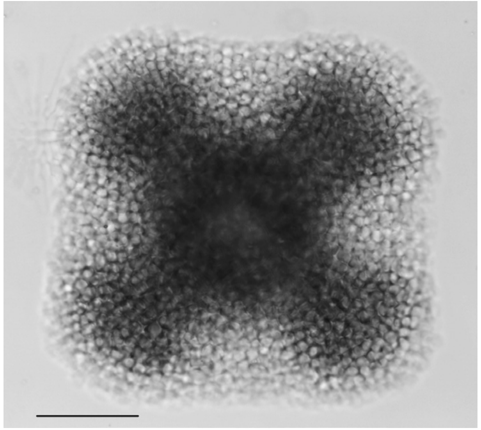

Spongaster tetras tetras
Spongodiscidae (spongodiscid)
Ehrenberg, 1860
Records: 16

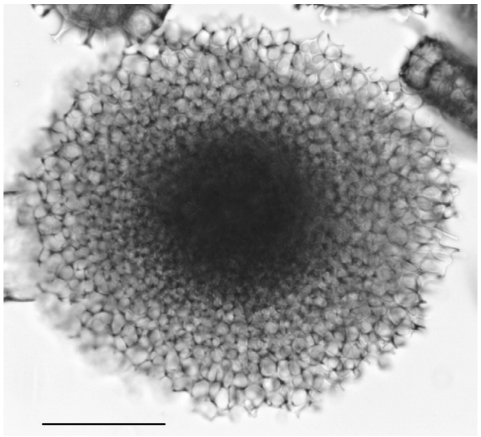

Spongodiscus resurgens
Spongodiscidae (spongodiscid)
Ehrenberg, 1854
Records: 49

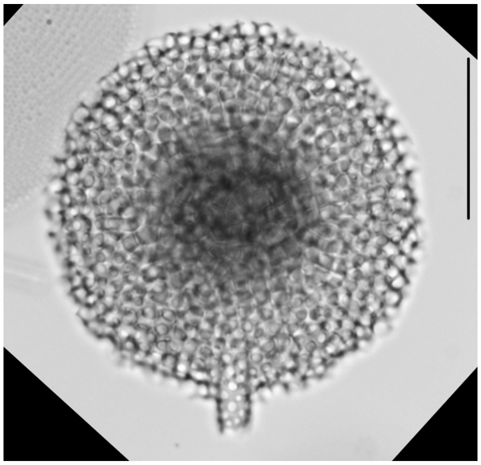

Spongodiscus setosus
Spongodiscidae (spongodiscid)
(Dreyer) Petrushevskaya, 1967
Records: 74

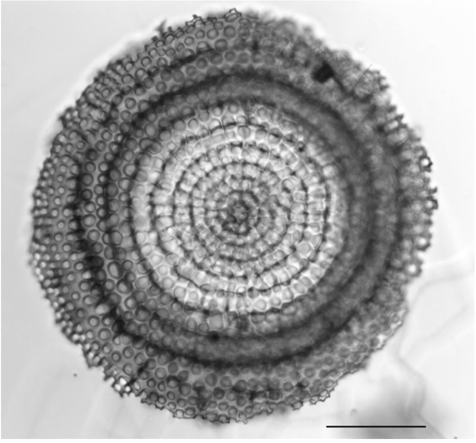

Spongodiscus sp C Kamikuri
Spongodiscidae (spongodiscid)
Kamikuri, 2017
Records: 26

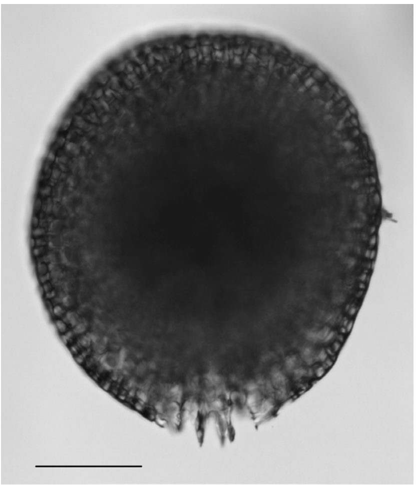

Spongopyle osculosa
Spongodiscidae (spongodiscid)
Dreyer, 1889
Records: 66

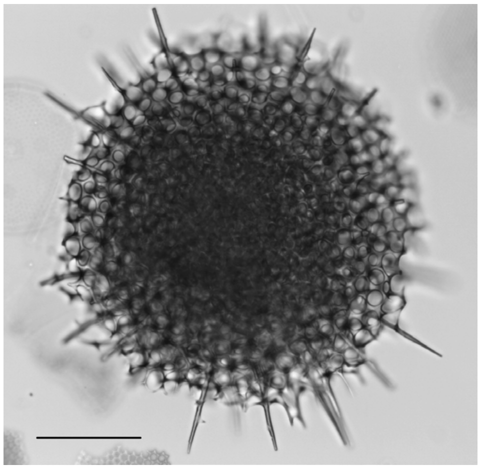

Spongotrochus glacialis
Spongodiscidae (spongodiscid)
Popofsky, 1908
Records: 41

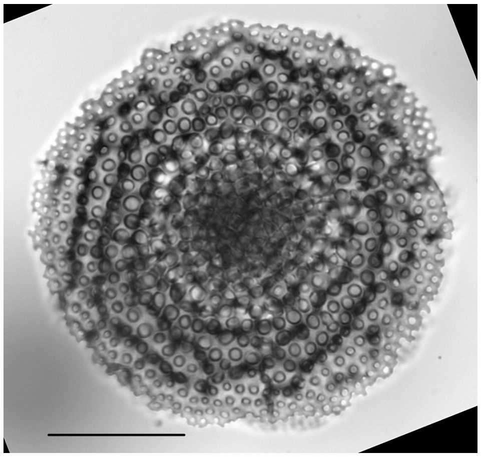

unknown spongodiscid sp 1
Spongodiscidae (spongodiscid)
Undescribed
Records: 61

This species differs from Stylochlamidium venustum and S. asteriscus for having patchy and irregular segments, that do not form full concentric rings around the center of the skeleton.

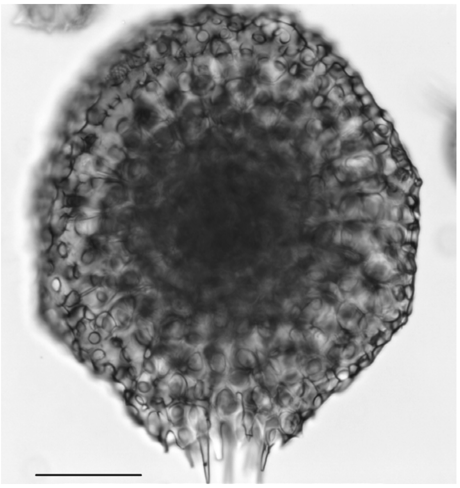

unknown spongodiscid sp 1 cf

Spongodiscidae (spongodiscid)

Undescribed

Records: 59

This species differs from unknown spongodiscid-discid sp 1 in that it is significantly more heavily silicified, has rough external texture, and tends to have a more clear termination at the edge of the outer shell. A spiny pylome is sometimes present.

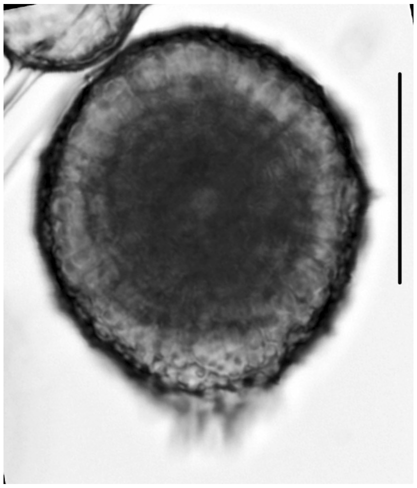

unknown spongodiscid sp 4

Spongodiscidae (spongodiscid)

Undescribed

Records: 17

This species differs from the others in that it has a tightly-coiled interior structure, with a relatively large empty gap separating it from the cortical shell. A spiny pylome is present.

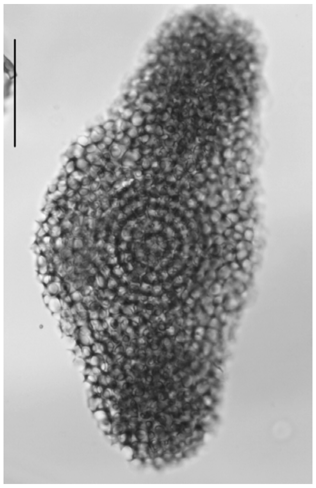

unknown spongodiscid sp 6

Spongodiscidae (spongodiscid)

Undescribed

Records: 12

This species is unique in that it has two long arms, that are oriented not quite at 90 degrees from each other. This differentiates it from those in the Dictyocorne genus.

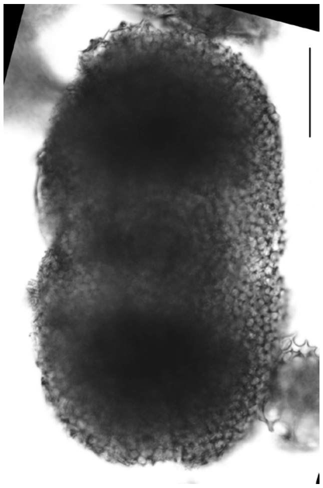

unknown spongodiscid sp 7

Spongodiscidae (spongodiscid)

Undescribed

Records: 8

This species differs from unknown spongodiscid-discid sp 6 in that it is significantly thicker, has arms oriented at 180 degrees from each other, with thick silica accumulations at their ends.

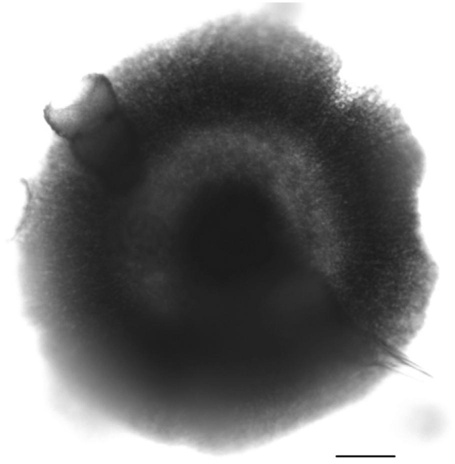

unknown spongodiscid sp 8

Spongodiscidae (spongodiscid)

Undescribed

Records: 5

This species differs from Spongodiscus resur-gens in that it is much larger, and has thick areas around the shell perimeter as well as at the center of the shell.

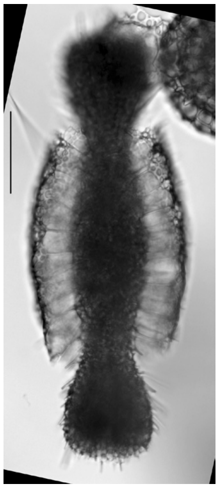

Spongurus cylindricus

Spongodiscidae (spongurid)

Haeckel, 1860

Records: 33

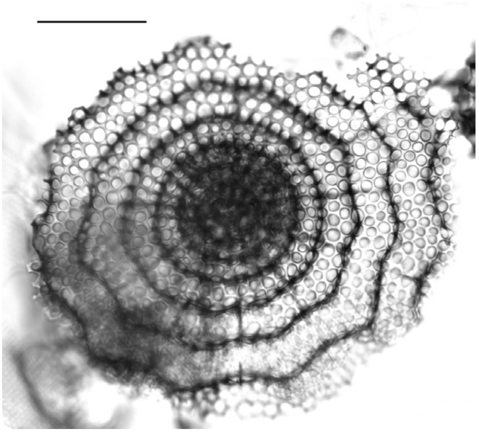

Stylochlamydium venustum-asteriscus

Spongodiscidae (stylodictyid)

Bailey, 1856 & Haeckel, 1887

Records: 40

This group includes specimens that resemble both or either described species.

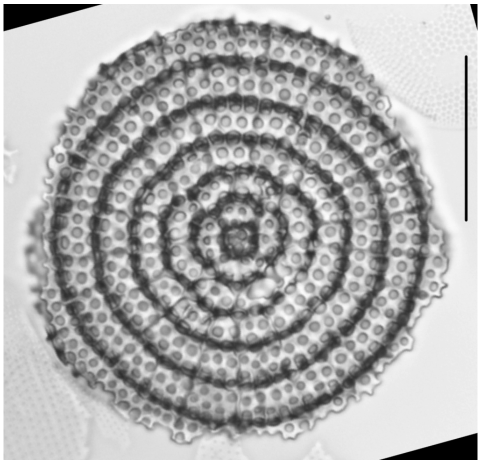

Stylodictya aculeata

Spongodiscidae (stylodictyid)

Jørgensen, 1905

Records: 58

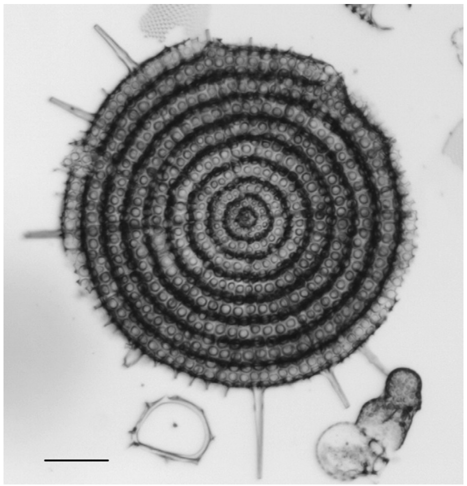

Stylodictya multispina

Spongodiscidae (stylodictyid)

Haeckel, 1860

Records: 63

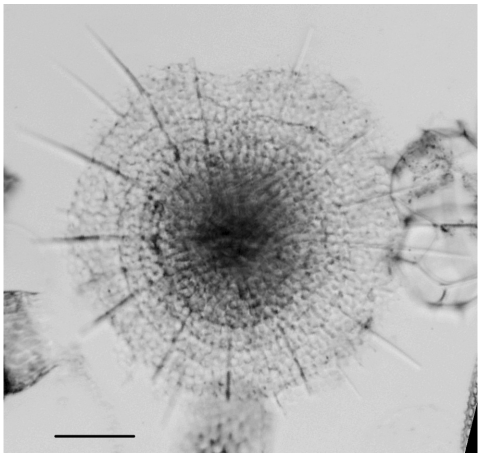

Stylodictya multispina cf

Spongodiscidae (stylodictyid)

Undescribed

Records: 9

This species differs from Stylodictya multispina in that it has more poorly-defined segments, and has a concave region at one part of the shell exterior.

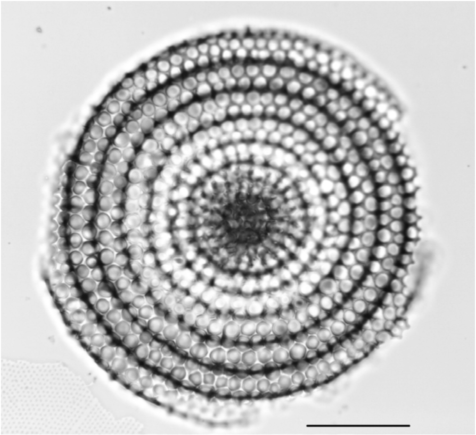

Stylodictya sp 1

Spongodiscidae (stylodictyid)

Undescribed

Records: 46

This species resembles Stylodictya multispina, but lacks long transverse spines.

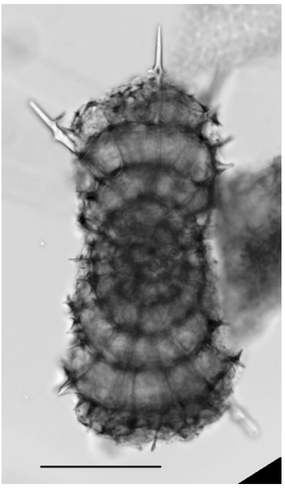

unknown spongodiscid sp 3

Spongodiscidae (stylodictyid)

Undescribed

Records: 13

The overall shape of this species is oval to rectangular, differentiating it from Stylodictya multispina. It has at least six prominent conical spines.

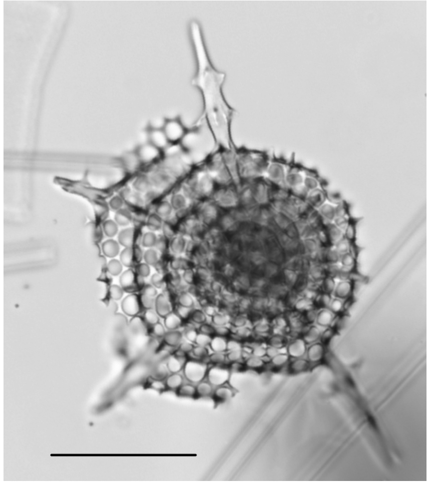

unknown spongodiscid sp 5

Spongodiscidae (stylodictyid)

Undescribed

Records: 40

This species differs from Stylodictya multispina in that it has more massive spines, and whorls that are slightly angular, rather than circular.

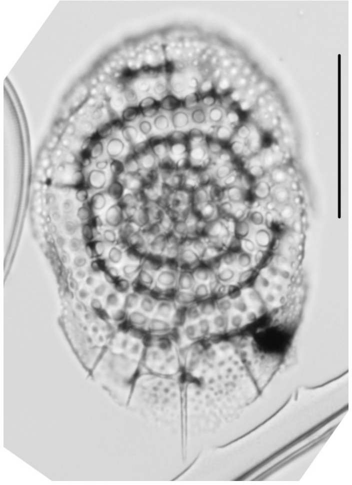

unknown spongodiscid sp 9

Spongodiscidae (stylodictyid)

Undescribed

Records: 35

This species is differentiated from Stylodictya multispina by its overall oval shape, and irregular-shaped whorls. It differs from unknown spongodiscid sp 1 in that it is oval, rather than round.

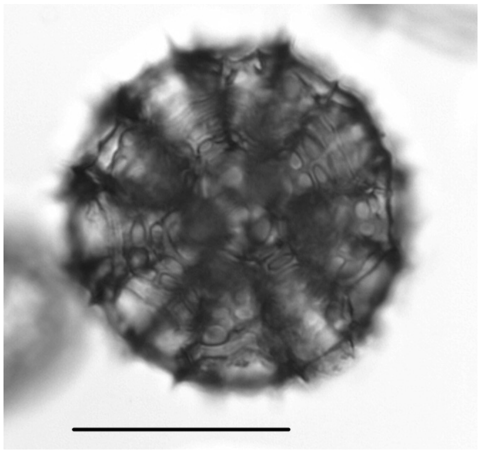

unknown spumellarian sp 1

Spumellaria indetermined

Undescribed

Records: 16

This shape of this species is difficult to discern, despite the observation of four specimens. It appears to be spherical, with large, hexagonal exterior openings, and smaller semi-quadrate pores lining the interior chambers. These chambers form a distinct linear pattern.
